# An accessible property classification framework to predict the solubility of functionalised naphthalenes and rylenes in organic solvents

**DOI:** 10.1039/d6dd00275g

**Published:** 2026-09-21

**Authors:** Connor R. M. MacDonald, Alex S. Loch, Neil G. Berry, Emily R. Draper

**Affiliations:** a School of Chemistry, University of Glasgow Glasgow G12 8QQ UK emily.draper@glasgow.ac.uk; b Department of Chemistry, University of Liverpool Liverpool L69 7ZD UK

## Abstract

The development of functional materials such as multilayer films from supramolecular self-assembling materials is hindered by the current lack of design principles and predictability. Quantitative structure–property relationships have emerged as a useful technique to tie properties of interest to the molecular structure across various applications but have not been widely explored in supramolecular material problems. Herein we describe and share an accessible, visual programming, classification framework which we have applied to the prediction of solubility of amino acid-functionalised napthalenes and rylenes. We show through a proof-of-concept dataset for amino acid-functionalised naphthalenes and rylenes that solubility can be effectively classified with sufficient high-quality training data, and highlight the need for standardised characterisation procedures in the field of supramolecular chemistry if more complex and informative quantitative structure–property relationships are to be developed. Such models could ultimately afford the tailored design of functional materials.

## Abstract 2

We show through a proof-of-concept dataset for naphthalenes and rylenes that solubility can be effectively classified with sufficient high-quality training data, and highlight the need for standardised characterisation procedures.

## Graphical abstract

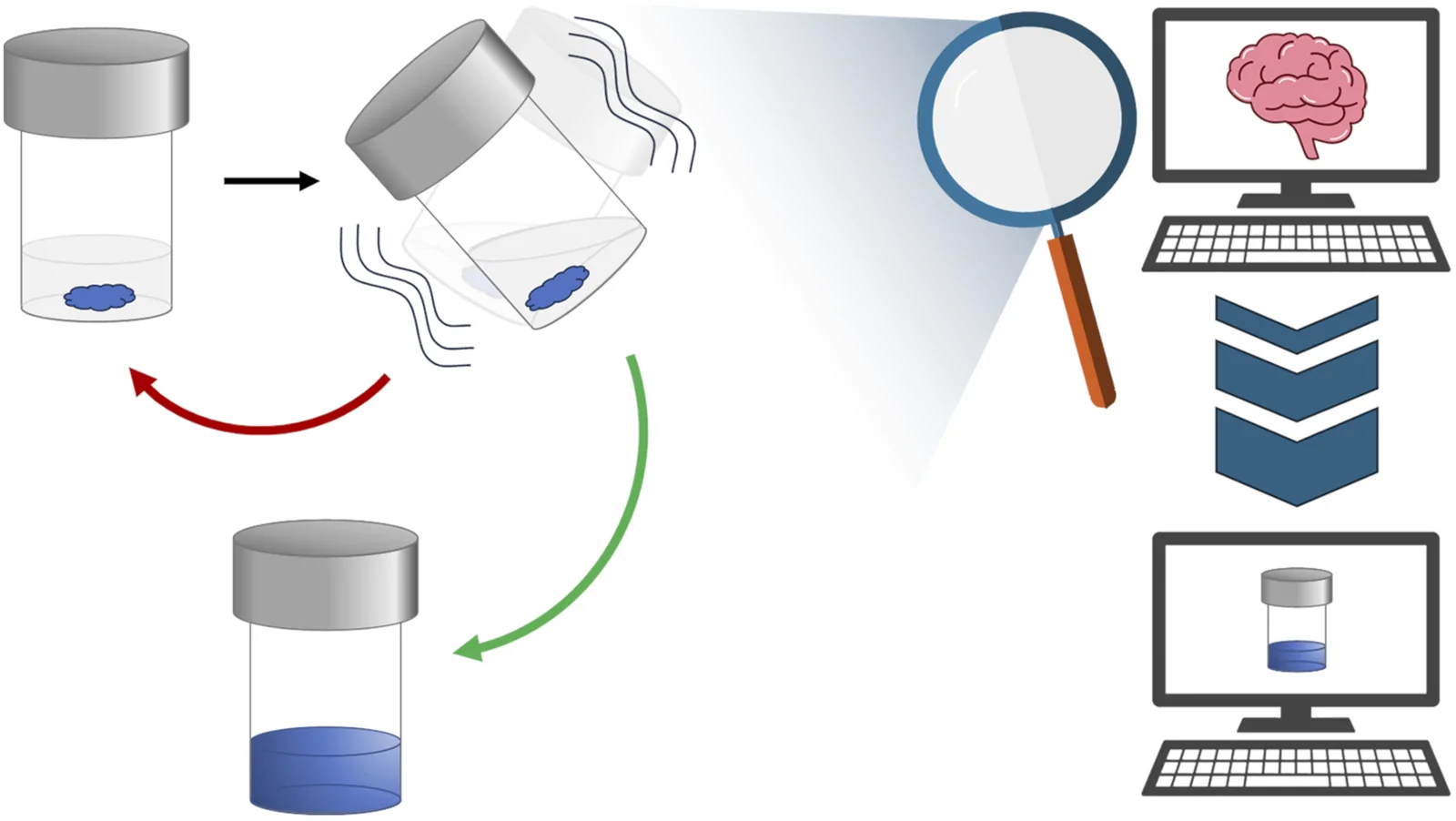

## Introduction

Smart materials are gaining increasing popularity due to their broader range of applications compared to conventional materials. Smart organic films are of particular interest due to their range of responsive properties.^2–6^

The layer-by-layer assembly of multilayer films is a versatile method of material fabrication, allowing functionality that cannot be achieved from a single layer alone. These constructions benefit from simplicity, versatility, and precise controllability, however, mixing between layers can diminish the interfaces of the film if the preceding layers cannot withstand erosion from the solvents used during subsequent depositions.^2^ While films can be chemically altered after deposition to ensure orthogonality to subsequent solvents, changing the chemical structure may diminish the properties of interest. Instead, using chemically orthogonal solvents in each layer can ensure that the deposited material is unaltered, while minimizing interface erosion (Fig. 1).^2^

**Fig. 1:**
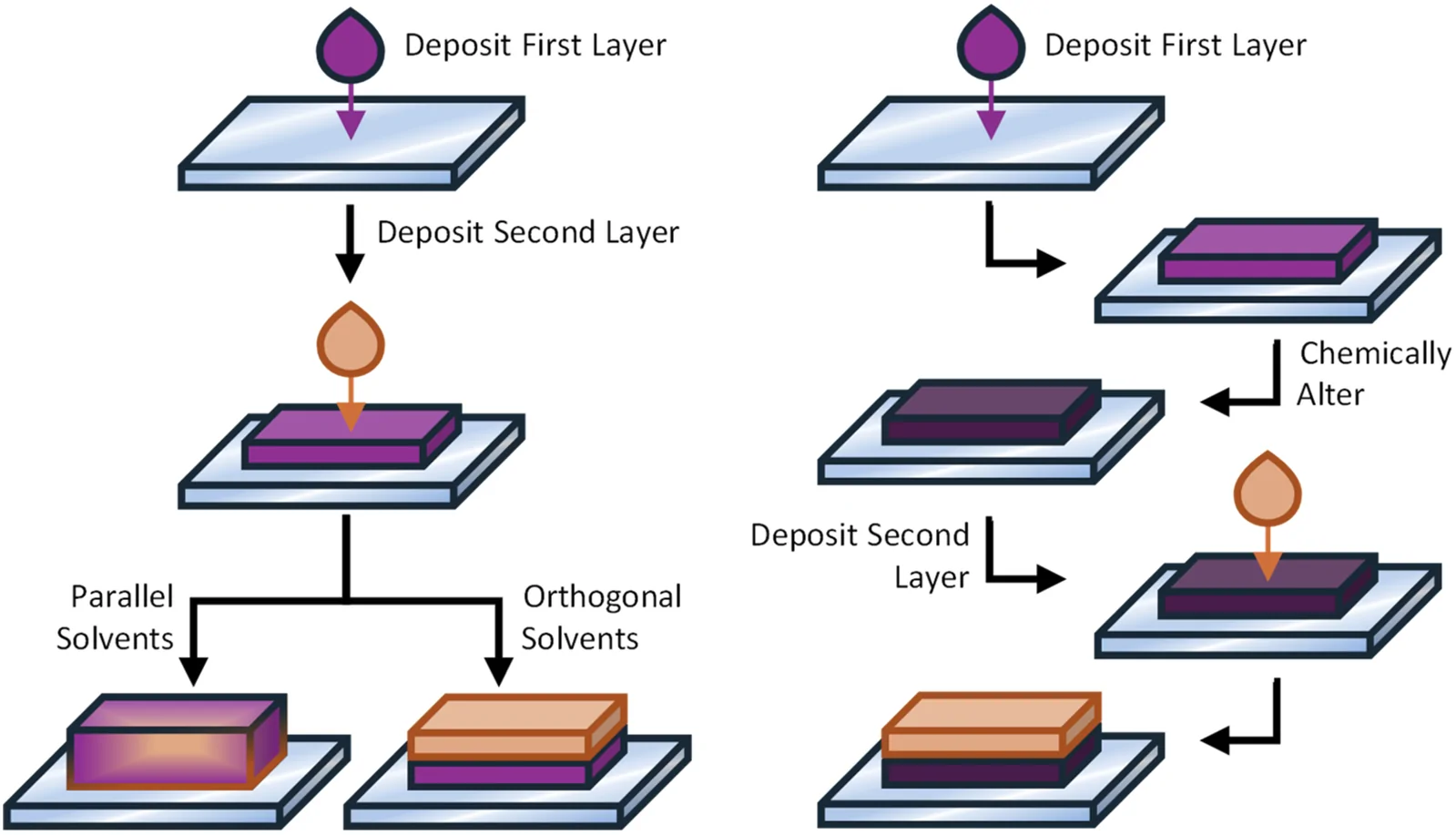
Cartoon depicting the fabrication of solution-processed multilayer films. When using parallel solvent systems, layers can intermix with the preceding layer. To avoid this, the solvents in adjacent layers must be orthogonal, or the first layer must be chemically altered after deposition to resist the solvent of the second layer.

Derivatives of highly aromatic small molecules, such as rylenes, have been shown to afford films with unique properties.^4,7,8^ These properties can be tuned, driven by changes in the hierarchical self-assembly.^9^ Given the influence of solubility and hydrophobic effects between solute and solvent across the scales of assembly, improved predictions of the solubility of self-assembling molecules is a key step to predicting more complex interactions.^10–12^

However, given the innumerable number of structural changes that may be made to tune the molecule's properties, testing the solubility of each candidate can be expense and time consuming. Instead, having access to fast and accurate predictions of solvent compatibility could aid in the design of such materials by identifying candidates with ideal properties. Machine learning is expected to be a promising strategy to improve the discovery and optimisation of supramolecular materials.^13^

Quantitative structure–property relationships have emerged as a useful tool for chemical prediction, linking descriptors of chemical structure to the properties of interest, with a breadth of literature discussing their development and application.^14–16^

While QSPR tools exist for solubility prediction, the data used in such models is limited and is unreproducible in some cases, making the application of such models problematic.^17,18^

Curated solubility datasets, such as BigSolDB 2.0 have emerged to aid the development of QSPRs, however, such datasets are of limited use to the prediction of compounds which share limited chemical similarity to the dataset, such as rylenes.^19^

To overcome this, here we describe an accessible, visual programming QSPR framework for property classification which we have applied to the problem of solubility prediction for rylenes and other similar amino acid-functionalised compounds.

Five QSPR models are reported, providing solubility classification predictions across five organic solvents [acetone, *N*,*N*-dimethylformamide (DMF), dimethyl sulfoxide (DMSO), ethanol, and tetrahydrofuran (THF)] covering a range of properties including hydrogen bond donor and acceptor solvents, protic and aprotic solvents, and solvents of varying polarity. We detail each step of the QSPR modelling including the dataset preparation and processing, model building, internal and external validation, and model visualisation and application.

The method by which solubility is measured, *e.g.*, how the insoluble material is separated from the supernatant, can have a significant impact on the reported solubility.^20^ As such, we have focussed solely on molecules synthesised and tested by ourselves, following a standardised protocol for each case.

We show how the open-source, visual programming platform KNIME^21^ can be used to develop a robust QSPR framework through a consensus of machine learning methods, how each model is optimised using cross-validation techniques, and is assessed by using multiple statistical metrics. The full protocol, from the data collection methods to model building, and final assessment follow guidelines for QSPR models.^22^

## Experimental

### Synthesis of materials investigated

The library of functionalised naphthalenes and rylenes examined here was prepared from previously reported compounds as well as a number of new molecules (Fig. 2). All solubilities were quantified using a UV-vis spectrophotometry approach. The full synthetic and characterisation details for the new molecules are described in the SI (Section 1).

**Fig. 2:**
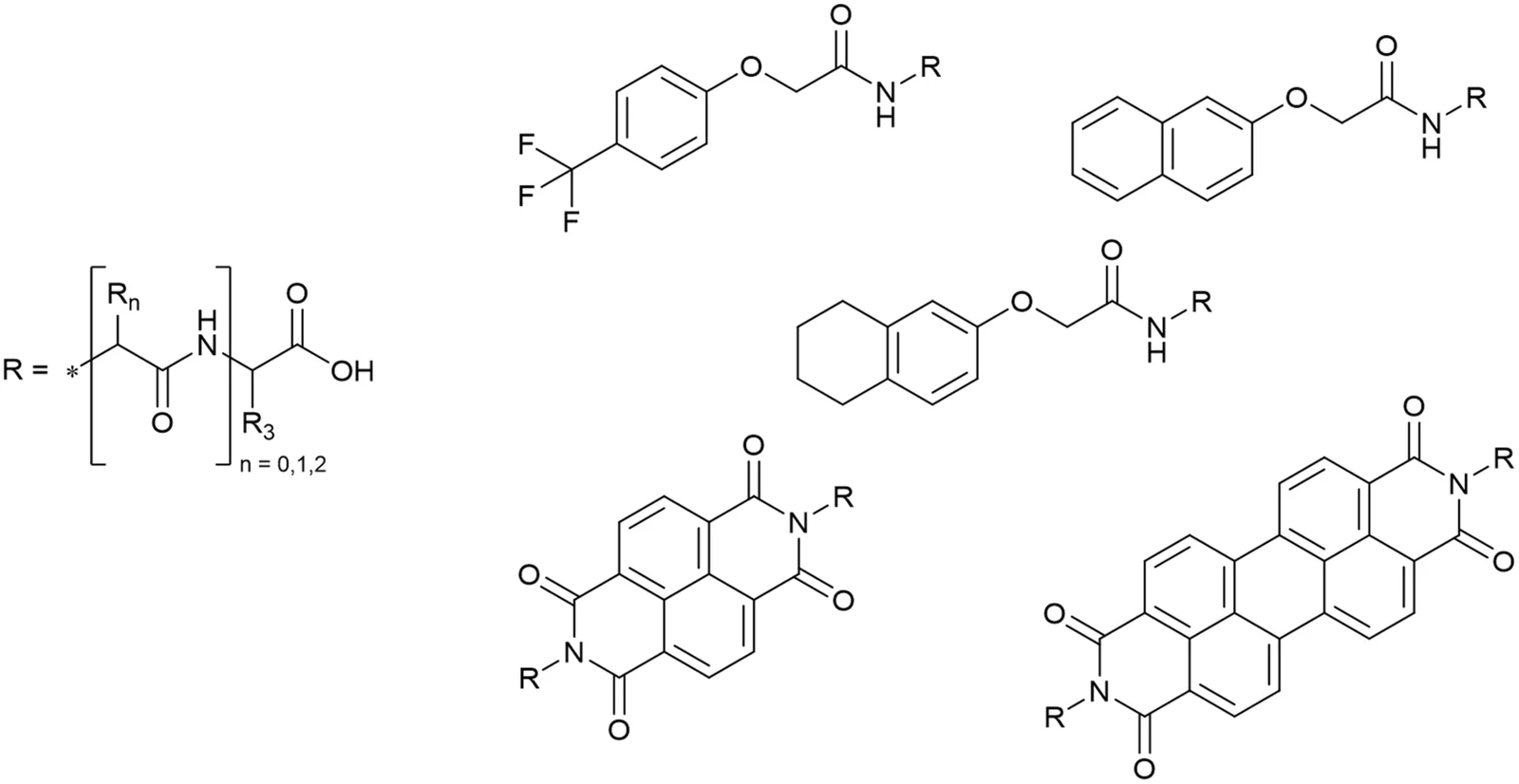
Generic structures of compound library (R_*n*_ = amino acid side chains); see SI for specific structures.

### QSPR

The molecules described above were generated *in silico* and converted to SMILES format using ChemDraw.^23^ Nine physicochemical descriptors and/or molecular fingerprint descriptors were calculated using KNIME (see SI, Section 3).^24^ KNIME was used for both the machine learning methods and model visualisation, with the aid of the RDKit python package.^25,26^

The modelability index (MODI) was calculated using our own scripts in KNIME.^27^ We chose Matthew's correlation co-efficient measure as our metric as it has recently been suggested that the most popular measure of classification models, receiver operating characteristics (ROC) area under the curve (AUC), should be replaced by MCC as the standard metric for assessing binary classification.^28^ The domain of applicability of each model was considered using fingerprint distance based metrics as described by Sahigara *et al.*^29^ The validation library was generated from SMILES representing previously reported structures similar to the training compounds as provided by Reaxys®^30^ (see SI, Section 3).

## Results and discussion

The materials were classified as soluble if their solubility exceeded 10 mg mL^−1^ as determined by the generation and application of UV-visible spectrophotometer calibration curves (see SI, Section 2). Solutions were prepared only at room temperature (∼20 °C) to minimise the influence of changing temperature on the measured solubility. While focusing on room temperature was necessary to limit the scope of data collection, predictions at varying temperatures could be achieved in future by expanding the dataset to include temperature-dependent measurements. The threshold was chosen as it exceeds the typical concentration at which the self-assembly occurs for these families of materials, enabling their application in functional films.^31–33^ Screening for compounds above this threshold would allow the wide chemical space of possible interesting candidates to be narrowed to those with a solubility sufficient to be cast into films. Furthermore, by understanding better solubility across a range of solvents, not only can we better identify useful candidates for multilayered film devices, but we also aid the synthesis process of viable candidates by identifying acceptable solvents to be used to purify the synthetic products. While dichotomising the dependent variable is commonly used to afford simpler models, it is important to highlight that significant information can be lost, reducing the statistical power of the resulting models. Furthermore, the predictions and statistics will be directly affected by the threshold chosen for endpoint dichotomisation. Subsequently, the data must be classified using purposeful thresholds to define classes that can afford meaningful insight. It is also important to consider that data points close to the threshold may introduce noise into the model if experimental error is significant; however, this can be mitigated with replicate analyses to ensure appropriate classifications are assigned. Therefore, future applications of the workflow may be more effective on already categorical properties.^34^

We have compiled datasets consisting of >50 compounds to build the predictive models. While much smaller compared to QSPRs built using databases such as BigSolDB 2.0, in which the naphthalenes and rylenes are poorly represented, the narrower scope of the training data should provide a better assessment of the local chemical space for the families of amino-acid substituted cores shown in Fig. 2.^19,35^ Our dataset represents a useful starting point for the development of solubility predictions of amino acid functionalised napthalenes and rylenes, however, to make more robust and effective predictions in future, the characterisation of larger datasets is required.

We highlight here the necessity for a complete reporting of experimental outcomes in a well-structured manner to aid in appropriate data extraction for future QSPRs. Solubility, for example, suffers from particularly high interlaboratory variance; however, with the application of consistent protocols. For example, while solubility measurements have been reported to have interlaboratory deviations of around 0.6 log units, with standardised methodology and sufficient reported information enabling effective curation, datasets with interlaboratory variances of 0.15 log units have been achieved.^36^ In the context of supramolecular chemistry, there are few conventions for data reporting, and useful metrics for building QSPRs are insufficiently reported or absent altogether.^37^ Furthermore, inequality in data access can limit the availability of information necessary for effective dataset development and curation.^38^ We stress the necessity of transparent, well-documented characterisation data across disciplines to maximise the effectiveness of future QSPR development.

Class distributions varied across the solvents with 47, 83, 91, 44, and 74% of compounds being classified as soluble in acetone, DMF, DMSO, ethanol, and THF; respectively. Stratified partitions of the overall data were taken to prepare (i) training sets with 42–43 compounds; and (ii) test sets with 10 compounds to test the prediction ability of the models. An external validation set with two compounds synthesised after the models were developed was assessed. The complete list of compounds and solubility properties is shown in the SI (Section 1 and Table S3).

### Predictive QSPR modelling

QSPRs tie molecular descriptors, data encoding the molecular structure and properties, to a measured property of interest by finding a relationship between them. This model can be as simple as a linear relationship, or more recently, complex non-linear machine learning models.^39^ Molecular descriptors range in complexity and information content.

While a model may be built using an information dense, three-dimensional (3D) electron density representation of each molecule, such representations can be difficult to calculate and implement. Instead, simpler representations such as 1-dimensional (1D) counts of molecular fragments, or 0D molecular counts such as a count of hydrogen bond donors and acceptors or the molecular mass can be used to represent the molecular structure (Fig. 3). Indeed, simpler descriptors are known to be crucial in predicting the connection between molecular structure and oral bioavailability, as evidenced by their use in Lipinski's rule of five.^41^

**Fig. 3:**
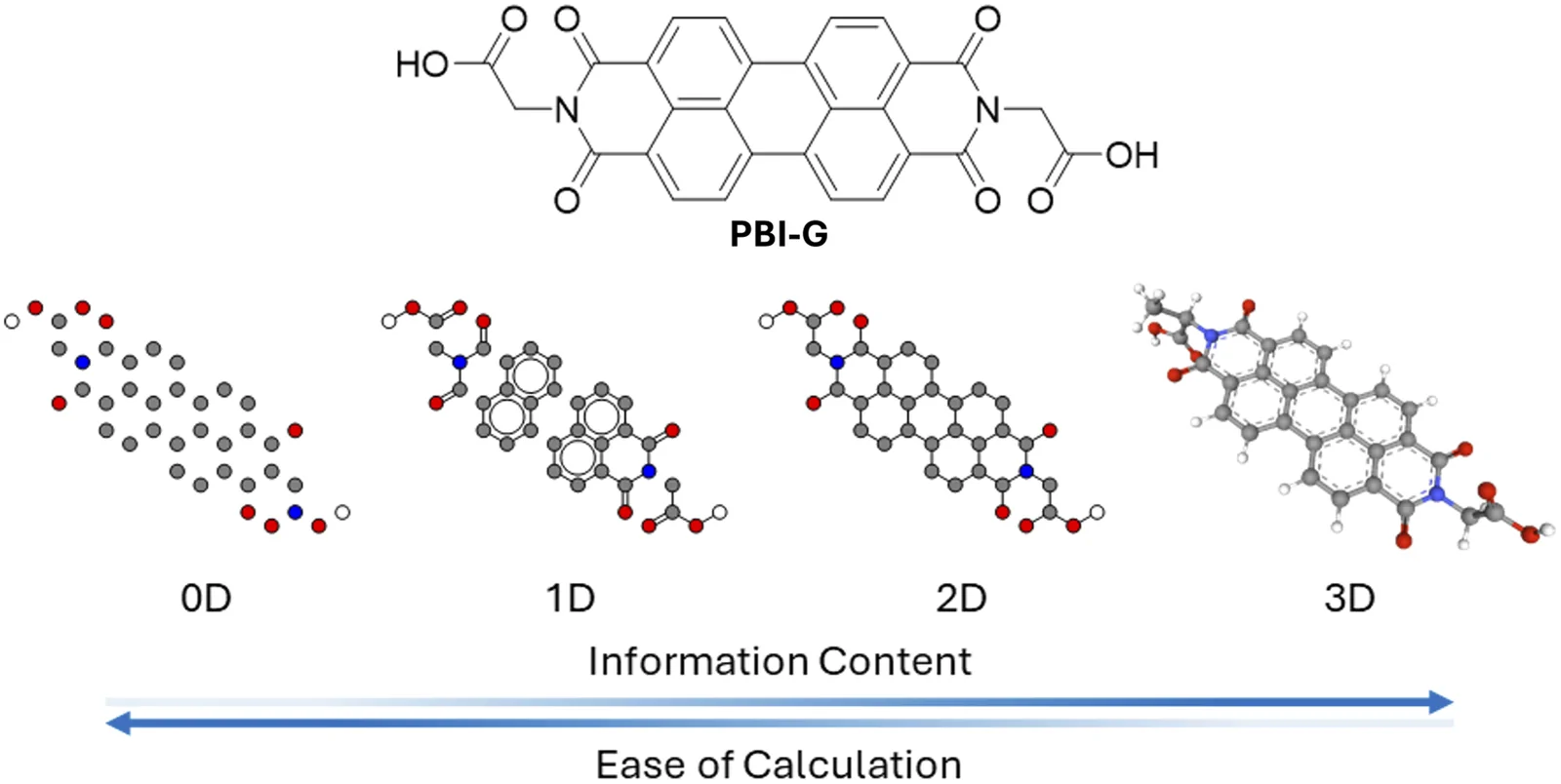
Graphical representation of molecular descriptors of varying dimensionality for PBI-G, and the relationship between information content and ease of calculation.^40^

From our solubility results, no simple relationship could be found between the simplest 0D physicochemical descriptors and solubility (see SI, Section 2, Fig. S4–S8), indicating that developing Lipinski-like rules would be challenging for these molecules.

We also attempted to find a simple relationship using more information-rich Morgan fingerprints descriptors, a simple representation of the presence of different chemical substructures,^42^ but no relationship was found (see SI, Section 2, Fig. S9–S13).

This was expected as the numerous interactions that affect the capacity of the molecules to dissolve across a range of solvents makes it difficult for a single descriptor to capture the nuances of solubility classification. Therefore, the development of QSPR classification models was deemed to be necessary to allow for robust solubility prediction, allowing for a more robust approach to linking the molecular descriptors with solubility.

There are many steps involved in designing a robust and effective QSPR system. An important consideration in building a QSPR model is ensuring that the data is accurate and of high quality. One of the major issues plaguing QSPR datasets is the prevalence of incorrect chemical structure representations.^43^ Therefore, before modelling can even begin, high-quality data must be obtained from well-documented and reliable sources. This data is then processed to obtain property labels and descriptors for each input, which are used in the model's training. Once a model is built, various methods can be used to assess its effectiveness on both the training data used to develop the model and on independent data, ensuring that truly predictive models have been identified.

These effective models can then be probed to identify the key molecular descriptors that visualise how the chemical structure affects the modelled property. Ultimately, such models can be deployed to predict the properties of candidate molecules (Fig. 4).

**Fig. 4:**
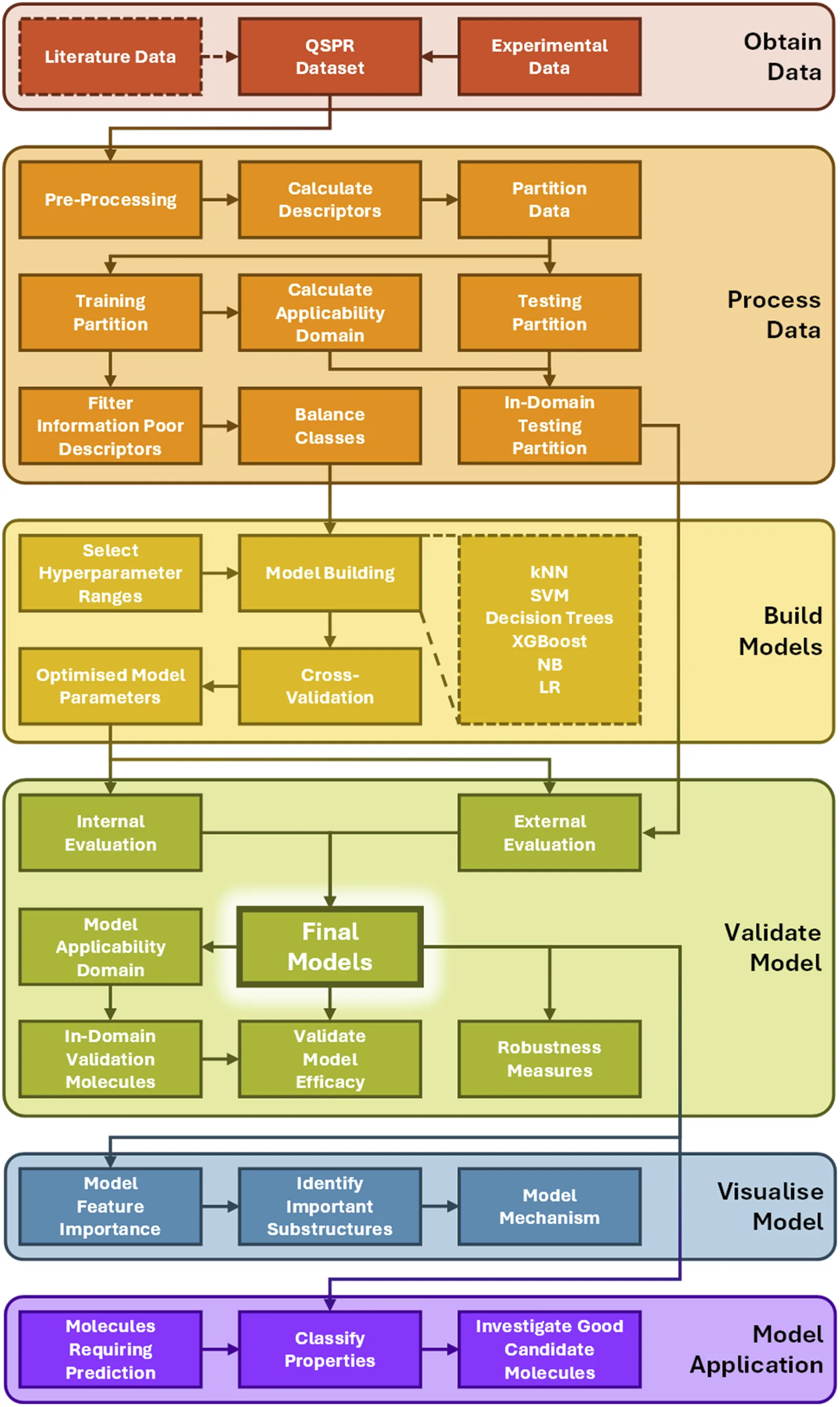
Overall QSPR modelling, testing, and validation workflow.

As part of the QSPR workflow, an assessment of the “modelability” of the training and test set partitions was performed using the modelability index (MODI).^27^ The MODI uses the classification of each compound with the most chemically similar molecule in the dataset to identify feasibility of obtaining predictive QSPR models from a classification dataset; with a MODI statistic of >0.65 being indicative of a modellable classification relationship.

The QSPR models were generated using a variety of machine learning methods: support vector machines (SVM),^44,45^ random forests (RF),^46^*k* nearest neighbours (kNN),^47^ XGBoost (XG),^48^ logistic regression (LR),^49^ naive Bayesian (NB)^49^ and C4.5 ensemble decision trees.^46^ Each modelling method employed molecular fingerprints to capture molecular properties.

Each machine learning technique has its own strengths and weaknesses. While one model may struggle to capture the nuances in a specific area of chemical space, a consensus of models should be better able to make reliable solubility classification predictions. By corroborating predictions with various models, the quality of predictions on unseen candidates can be dramatically improved.^50^ By identifying multiple predictive models, our capacity to rationally design new materials with tailored properties would be significantly improved, as the average of multiple good prediction models should be closer to the actual measurement than any given individual model.

Stratified sampling randomly splits data while ensuring representative class distributions in the test set, which provides better interpolation of the training space. Given its widespread use, ease of implementation, and our focus on within-domain interpolation over extrapolation, we opted to split the data into training and test sets using stratified sampling, ensuring the test set reflects a class distribution comparable to the entire dataset.

Given the limited data, a preliminary investigation of the training set quality was conducted using the modelability index (MODI). While the MODI can indicate whether a dataset is amenable to modelling alone, it is not a good estimator of the resulting model performance (Table 1; see SI).

**Table 1:** MODI of training and test set partitions across 10 random stratified partitions, and the specific MODI values of the best performing model

Model	Training set MODI range	Optimal training set MODI
Acetone	0.44–0.61	0.51
DMF	0.43–0.63	0.63
DMSO	0.43–0.81	0.60
Ethanol	0.46–0.67	0.63
THF	0.52–0.74	0.57

A repeated 5-fold cross-validation approach was used to select the optimal QSPR model for each method based on the largest Matthew's correlation coefficient (MCC). An ideal model has a MCC measure value of 1, with a random model taking a value of 0 and an acceptable value being >0.5. Using a cross-validated approach gives a good estimate of the predictive power of the models. The models generated from each machine learning method with associated statistics are shown in Table S5. Once the optimal model had been selected, we further assessed the model's merits using a range of measures: MCC, Cohen's Kappa (*κ*), and balanced accuracy (Tables 2 and S5).

**Table 2:** Overall quality of predictive performance of optimised QSPR models on the training set. Model is deemed to be good (G) when the following are satisfied: ROC A, balanced accuracy > 0.7, MCC > 0.5, *κ* > 0.4. Models are otherwise deemed to be bad (B)

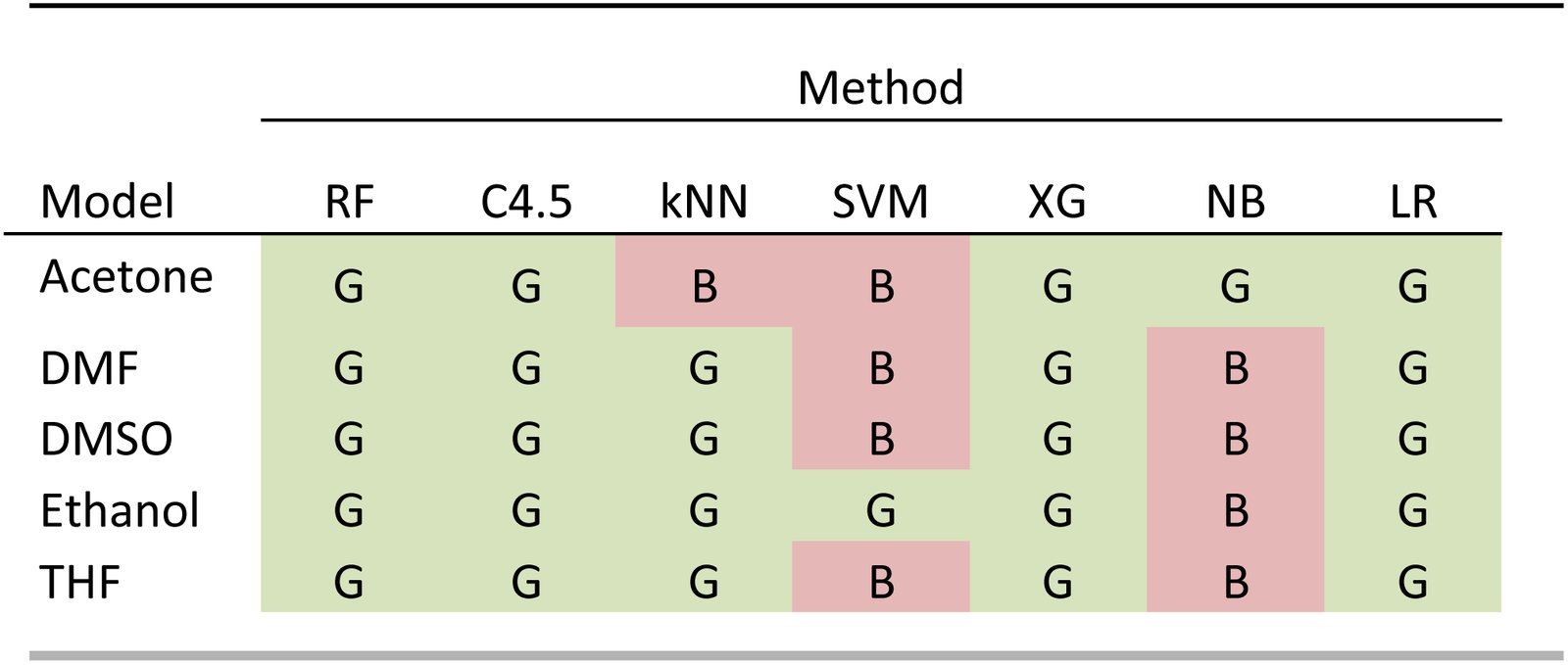

Across the different solvents, the class balance of the data varies markedly. Therefore, it is important that the metrics used are independent of the class distribution. In situations where a model could achieve high overall accuracy simply by favouring the dominant class; balanced accuracy, MCC, and *κ* can be used when class sizes are skewed.

Balanced accuracy is a measure of the number of correctly classified molecules in each class. It can vary between 0 and 1 with an ideal model having a value of 1 and an acceptable value being >0.7. Cohen's Kappa is a statistical measure that assesses the agreement between two raters; in this case, measured classifications are compared to their predicted classifications.

It can vary between −1 and 1 with an ideal model having a value of 1 and an acceptable value being >0.4.^50,51^

Assessing the quality of the model by applying it to the training set alone is insufficient to probe its true predictive performance on unseen data. It is important to assess that the models provide competent predictions for compounds with sufficient chemical similarity to the training set and were not used to train the model. To assess if a candidate molecule has sufficient chemical similarity, distance-based metrics described by Sahigara *et al.*^29^ were used to establish well-defined boundaries, known as “applicability domains” (see SI, Section 3). The Manhattan distance between each of the candidate descriptors and the average value for each descriptor in the training set was calculated. It is expected that molecules which are more similar to the training set will be better modelled by the resulting QSPRs. Therefore, it is important that the appropriate applicability domain is chosen to allow the reliability of predictions to be well-defined. By limiting test predictions to the in-domain candidates for each of the applicability domains of interest, the impact of excluding out-of-domain molecules on predictive performance was assessed for each boundary.

By considering the performance metrics on the models after applying the maxdist and dsz applicability domains, the impact of the applicability domain can be identified. While maxdist performed slightly better on the DMSO models, all other models showed comparable or better performance when employing the dsz applicability domain. In particular, for acetone prediction, only the XGBoost method showed acceptable performance using the maxdist domain, while the dsz domain yielded four predictive models with good metrics (Table 3; see SI, Tables S6–S12).

**Table 3:** Overall quality of predictive performance of optimised QSPR models on the in-domain dsz test set. Model is deemed to be good (G) when the following are satisfied: ROC A, balanced accuracy > 0.7, MCC > 0.5, *κ* > 0.4. Models are otherwise deemed to be bad (B)

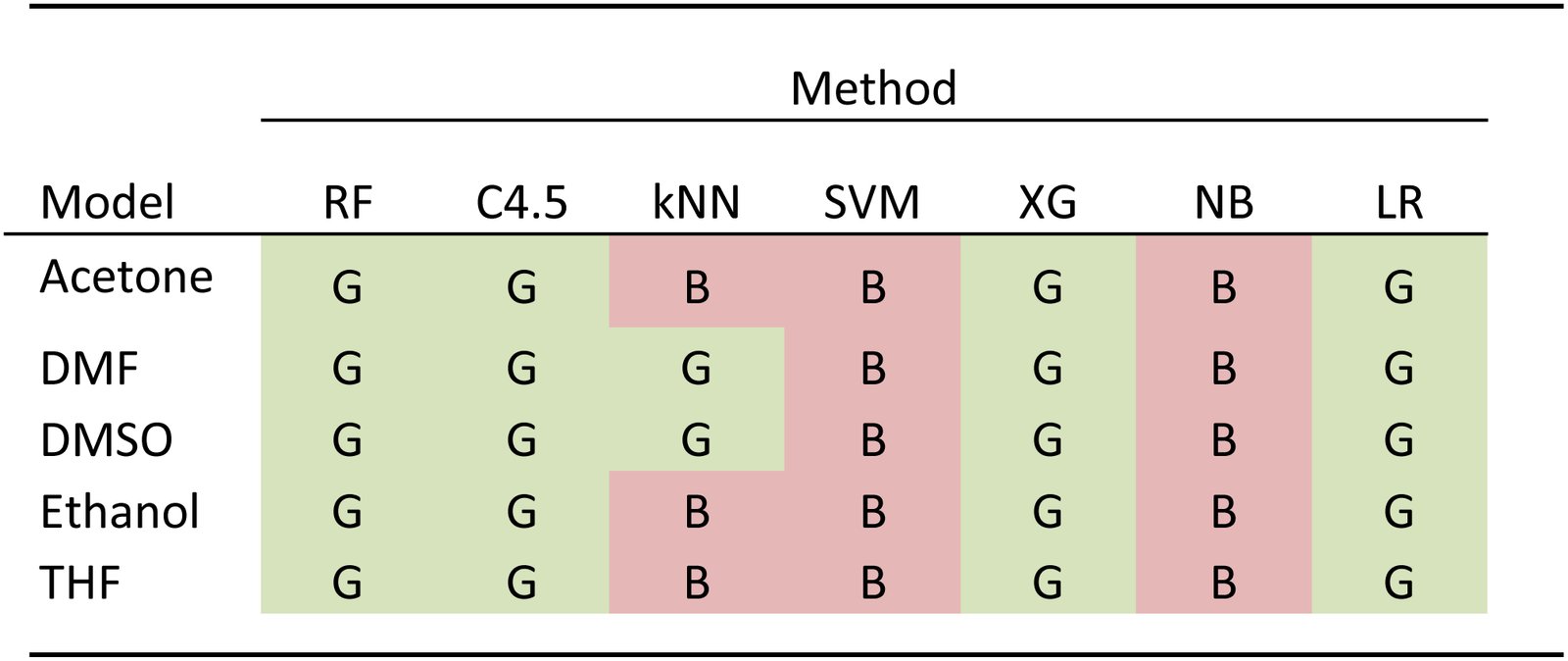

The predictive performance of each model is further identified by the receiver operator characteristic (ROC) curve (see SI, Fig. S14). ROC plots the true positive prediction rate against the false positive rate. An optimal ROC plot is one which has a perfect true positive rate and no false positives at every threshold, with a random model showing equal rates across the curve. The “good” DMF, DMSO, and THF models showed ideal or near-ideal ROC relationships, suggesting a highly predictive model was developed in this proof-of concept study. The acetone and ethanol models, while better than a random model, had poorer ROC curves, suggesting that the predictions from these models would be less reliable (Fig. 5).

**Fig. 5:**
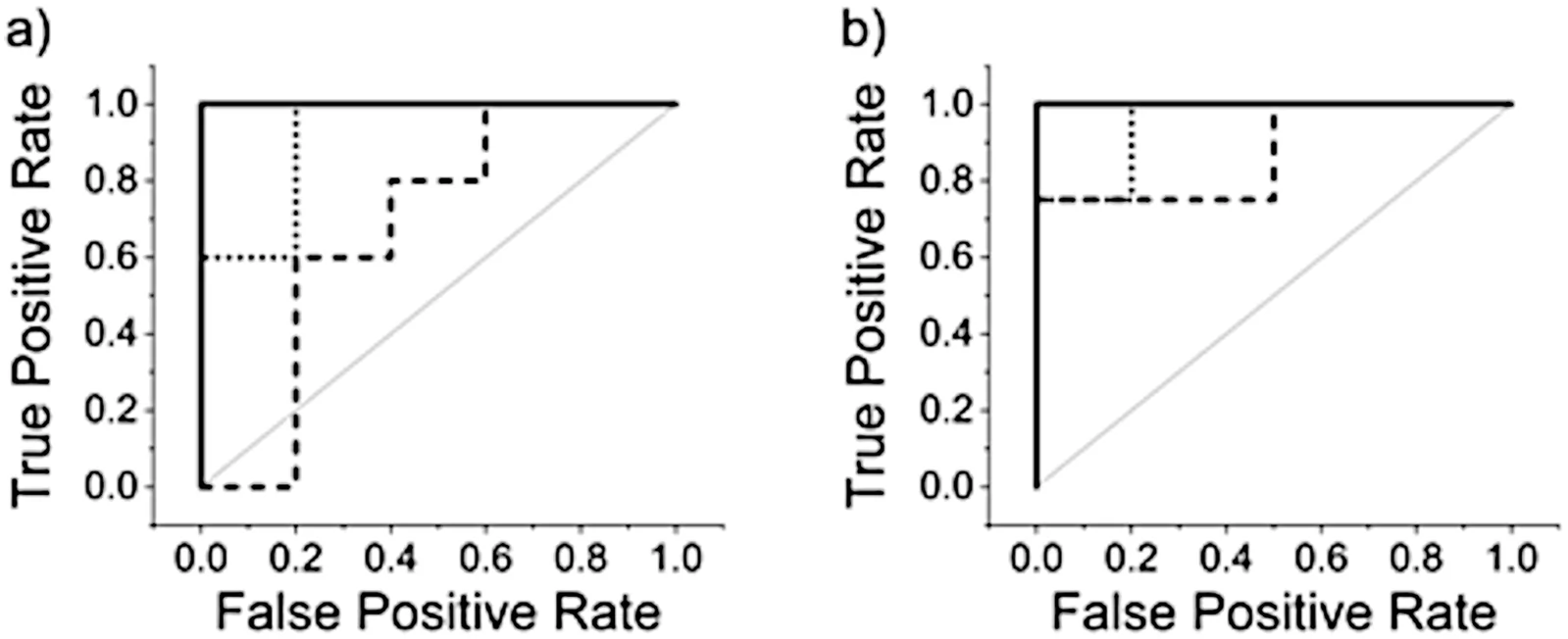
ROC curves for random forest models on (a) maxdist and (b) dsz in-domain test sets. The acetone model is represented by the dashed line, the ethanol by the dotted line; and the DMF, DMSO, and THF models by the solid line. The grey line indicates the ROC performance of a random predictive model.

Thus, the dsz domain was taken as the appropriate domain of applicability for assessing if predicted compounds had sufficient chemical similarity to the model's training set. Given that dsz is generally a more restricted domain of applicability, it was expected that in-domain compounds would have a greater degree of chemical similarity, and thus more predictable, further rationalising this decision.

For the 10 compounds in the acetone, DMF, DMSO, ethanol and THF test sets: between 6 and 9 compounds lay within the applicability domain of each model as defined by the molecular fingerprint descriptors used in the model building. Of these in-domain compounds, 4, 6, 5, 4, and 7 were soluble, and 4, 2, 1, 3, and 2 were insoluble in the respective solvents.

To further investigate the robustness of the optimised models, randomisation tests were conducted in which the target property classification is shuffled for the training, test, and in-domain test set compounds and predicted using the true model. An addition robustness test was performed wherein hyperparameters optimisation was performed on a randomised training set.

In both cases, the performance metrics should be much poorer than those derived from the true data. These metrics identify the statistical significance of the QSPR model.^52^ Indeed, the MCC metrics resulting from the randomisation tests were significantly poorer than that of the models found with true data (Table 4).

**Table 4:** Comparison of MCC scores of the in-domain test set on models built using the true data and models built on randomised data

Model	Method							
	RF		C4.5		XG		LR	
Dataset	True	Randomised	True	Randomised	True	Randomised	True	Randomised
Acetone	0.78	−0.06 ± 0.15	0.78	0.16 ± 0.12	0.78	−0.14 ± 0.10	0.78	0.07 ± 0.08
DMF	1	0.00 ± 0.21	1	−0.11 ± 0.14	1	0.12 ± 0.24	1	−0.19 ± 0.25
DMSO	0.63	0.00 ± 0.15	1	−0.25 ± 0.40	1	−0.07 ± 0.34	0.63	−0.02 ± 0.20
Ethanol	0.55	−0.14 ± 0.11	0.79	0.16 ± 0.12	0.6	0.12 ± 0.12	0.6	−0.19 ± 0.10
THF	1	−0.03 ± 0.17	0.76	−0.05 ± 0.30	0.76	−0.07 ± 0.17	1	0.3 ± 0.16

This increases our confidence in the good prediction metrics random forest, C4.5, XGBoost, and logistic regression models. When applying the dsz applicability domain to the randomised models, all randomised models showed a decrease in performance, however four of these models did not show a significant decrease (DMSO, RF; DMSO, LR; ethanol, XG; THF, C4.5 (see SI, Table S19)). This may indicate poorer generalisability for these models.

Thus, with the 4 models that were demonstrated to perform well in predicting the solubility classification in the test sets across the 5 solvents, we wished to predict the solubility of naphthalenes and rylenes from a large *in silico* library. Select compounds were synthesised and tested to act as a validation set and demonstrate the ability of our approach in successfully identifying both compounds that are soluble and those that are not.

### Virtual library design, generation and screening

Initially, an *in silico* library of dipeptide functionalised rylenes, as shown in Fig. 1, was generated using RDKit^25^ and Python^26^ to combine the SMILES of aromatic cores and amino acid side chains (see SI, Fig. S3). The amino acid side chains studied were limited to glycine, valine, leucine, alanine, phenylalanine, isoleucine, and tyrosine, as they were expected to be the most synthetically accessible. While the synthesis of the dipeptide itself was trivial, the installation of the dipeptide onto the rylenes cores proved to be problematic. Therefore, the literature was screened for naphthalene and rylenes compounds with the general form as shown in Fig. 1 which had reported synthetic pathways as documented in Reaxys®.^30^ This limited the synthesis of validation compounds to those that are expected to be synthetically accessible.

The library in total contained 1207 compounds, each of which had the same set of descriptors calculated as for the training set of molecules. When predicting properties, a compound closer to the training set typically yields more accurate predictions. For candidates within the applicability domain, defined using chemical similarity measures based on Manhattan fingerprint distances, the prediction is more trustworthy.^29^ For the library of 1207 compounds, those molecules which lay outside the model applicability domain were removed, leaving only 41–78 compounds. For each of the in-domain compounds, predictions were made on their solubility using the accepted models. While the *in silico* library of validation candidates was limited to core structures of the QSPR dataset, the resulting model did not capture the breadth of previously reported amino acid derivatives. The narrow applicability domain indicates the utility of these models where there is more confidence in the predictions. Undoubtedly, this could be notably improved by the inclusion of the more diverse derivatives previously reported.

Three in-domain candidate molecules were chosen to be synthesised and tested using the combined likelihood from the four machine learning models. However, two of the compounds could not be accessed using the reported syntheses (see SI, Table S21). In addition, an out-of-domain candidate was synthesised for investigation. Its higher prediction confidence metric across models (derived from the prediction probabilities produced by each model; see SI, Section 3), despite being out-of-domain, illustrates the model's capacity to probe chemical spaces underrepresented in the training data, giving an indication of out-of-domain areas that may provide the desired properties of interest, requiring further dataset expansion. The remaining validation compound predictions agreed with the DMF, DMSO, ethanol, and THF measurements; however, the acetone measurement did not agree with its prediction for the in-domain candidate (Table 5). When considering the consensus of prediction confidence across the four accepted models, the misclassified prediction had the lowest confidence. This indicates that the consensus confidence metric can help identify the candidates that are most likely to be correctly predicted. While the limited validation investigation indicates better predictive power of the DMF, DMSO, and THF models, many more validation compounds would be ideal to assess the predictive performance more generally. Further investigation would also highlight areas of chemical space that warrant further investigation to improve the modelling dataset.

**Table 5:** Validation prediction for each QSPR model with correct prediction highlighted in green, and incorrect predictions highlighted in red. Confidence is the average and standard deviation across the RF, C4.5, XG, and LR models. Predictions with a confidence greater than 40% are deemed to be of high confidence^1^

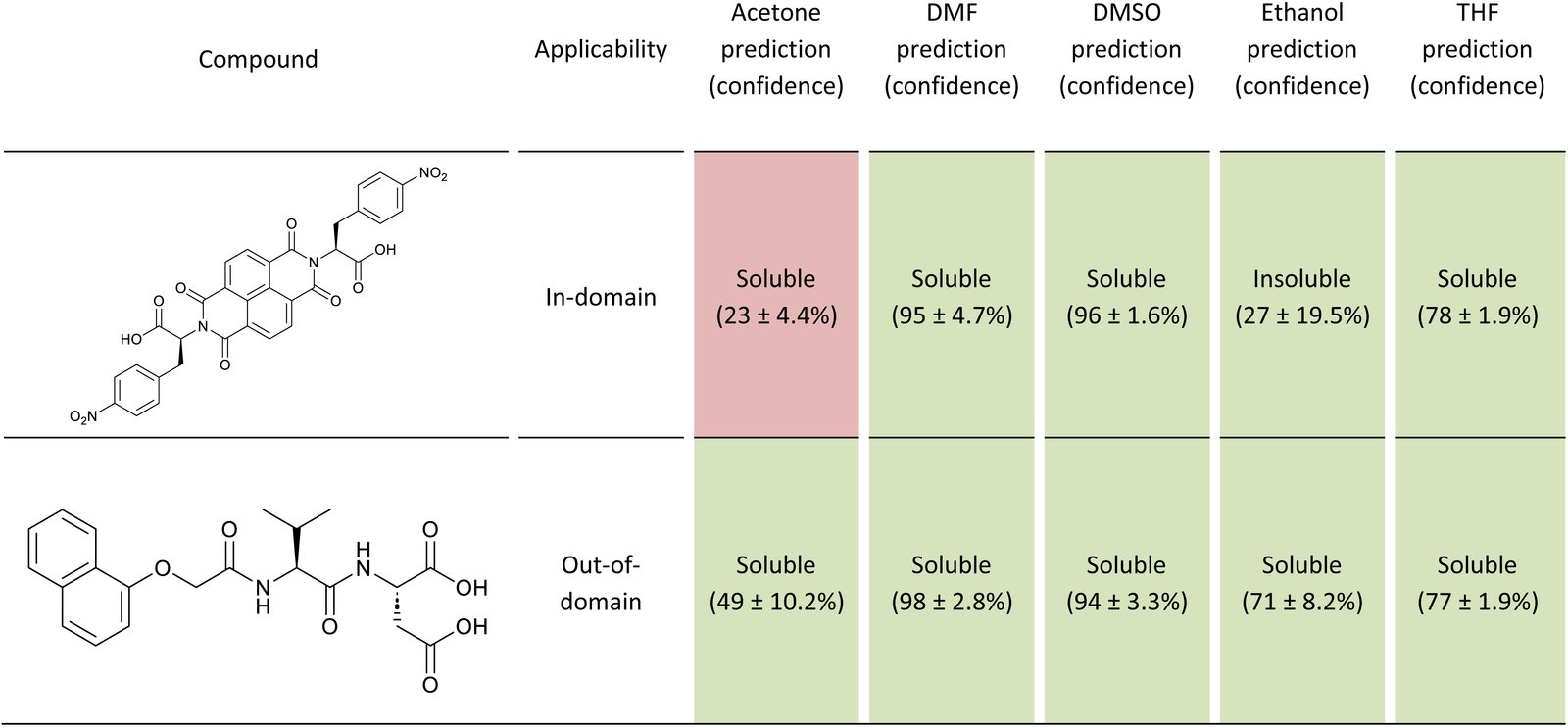

The validation predictions indicate that while predictive QSPR models have been found for DMF, DMSO, and THF, further optimisation is required to improve the prediction of acetone and ethanol solubility for amino acid functionalised rylenes and naphthalenes, highlighted by the lower prediction confidence of the acetone and ethanol predictions, and the incorrect validation prediction in acetone in particular.

Having identified models with predictive ability for selected amino acid-functionalized naphthalenes and rylenes, chemical substructure importance can be calculated to extract useful insights for future investigation and rational design.

The size of the datasets used in this study limited the insights that could be derived from complex feature importance metrics such as Shapley values. However, these metrics suggest that while the inclusion of valine moieties affords little change in the solubility in DMF and DMSO, their presence simultaneously reduces solubility in acetone while increasing solubility in ethanol and THF. Further investigation and experimental validation of such a relationship could offer a pathway to designing molecules with predictable compatibility in orthogonal solvents (see SI, Fig. S15).

Simpler importance metrics highlight the solubility's dependence on the core aromatic structure, the length of the peptide functionalisation, and the aromaticity of amino acid side chains. These models indicate that controlling the aromaticity of the side chains offers the best approach to tuning the solubility of these materials (see SI, Fig. S16–S20). These rational conclusions indicate that meaningful relationships can be established from the Morgan fingerprint and used to find intuitive chemical insight.

## Conclusions

In conclusion, we have demonstrated that the solubility of naphthalene and rylene derivatives can be modelled using local QSPR models. We have shown that investigation of physicochemical descriptions alone is not enough to elucidate the complexity that governs solubility, and that more robust machine learning approaches are required to model how the nuances of the chemical structure impact bulk properties.

The visual framework we have developed provides predictions of solubility and can filter out molecules deemed to be too chemically dissimilar for adequate predictions to be made. If a molecule lies outside of the applicability domain, it does not guarantee that the prediction is incorrect but helps inform the likelihood of a molecule's solubility in the given solvent.

While we have used this framework to investigate the solubility of naphthalenes and rylenes, the workflow has been developed such that it can be adapted to build models for any chemical classification problem or further improved with the addition of further solubility data. This work is an illustrative validation of the approach, and considerably more data is needed to move towards a system with general predictive design ability. While machine learning is expected to be a promising strategy to improve the discovery and optimisation of supramolecular materials, there is currently little effort to unify characterisations across the field. We stress here that development of more complex QSPR models for aiding supramolecular material design would be advantaged by the development of standardised characterisation protocols to allow for high-quality training data preparation from literature review methods.

We invite researchers to use this visual programming tool through which users can predict the solubility of compounds similar to those discussed in this paper, retrain the models with their own solubility data or desired solubility threshold using our data, or tackle another chemical classification problem entirely.

## Author contributions

Connor R. M. MacDonald: methodology, investigation, resources, software, formal analysis, visualisation, writing – original draft, writing – review & editing. Emily R. Draper: conceptualisation, funding acquisition, resources, project administration, supervision, writing – original draft; writing – review & editing. Neil G. Berry: supervision, writing – original draft, writing – review & editing. Alex S. Loch: resources, writing – review & editing.

## Conflicts of interest

There are no conflicts to declare.

## Supplementary Material

DD-005-D6DD00275G-s001

## Data Availability

The classification workflow can be found freely available for use at the following addresses: https://hub.knime.com/2197929m/spaces/Public/Classification%20QSPR, https://github.com/CRMMacDonald/Accessible-QSPR-Supplementary-Files, https://zenodo.org/records/22828403. All characterisation data used to build the models has been supplied in the supplementary information (SI). Supplementary information is available. See DOI: https://doi.org/10.1039/d6dd00275g.
