# Supplementary material for: An accessible property classification framework to predict the solubility of functionalised naphthalenes and rylenes in organic solvents
Source: Digit Discov. 2026 Sep 21;5(11):4894–902. doi: 10.1039/d6dd00275g (PMC13618208; doi:10.1039/d6dd00275g)
Supplement: Supplementary file 2 [file DD-005-D6DD00275G-s001.pdf]

This journal is © The Royal Society of Chemistry 2026
Please do not adjust margins

ELECTRONIC SUPPORTING INFORMATION

An accessible property classification framework to predict the solubility of functionalised naphthalenes and rylenes in organic solvents

Connor R. M. MacDonald,[a] Alex S. Loch,[a] Neil G. Berry,[b] and Emily R. Draper *[a]

[a] School of Chemistry, University of Glasgow, Glasgow, G12 8QQ, UK.
[b] Department of Chemistry, University of Liverpool, Liverpool L69 7ZD, UK.

Contents

Section 1	2
Materials	2
Section 2	15
Solubility Data Collection Methods	15
Solubility Data	16
Solubility Data Summary	39
Section 3	41
QSPR	41
Supplementary Figures	46
Supplementary Tables	63
References	76

Please do not adjust margins

Please do not adjust margins

Section 1

Materials

A number of the compounds used to build the dataset have been previously reported (structures **1** – **34;** Figure S1). [1-18]

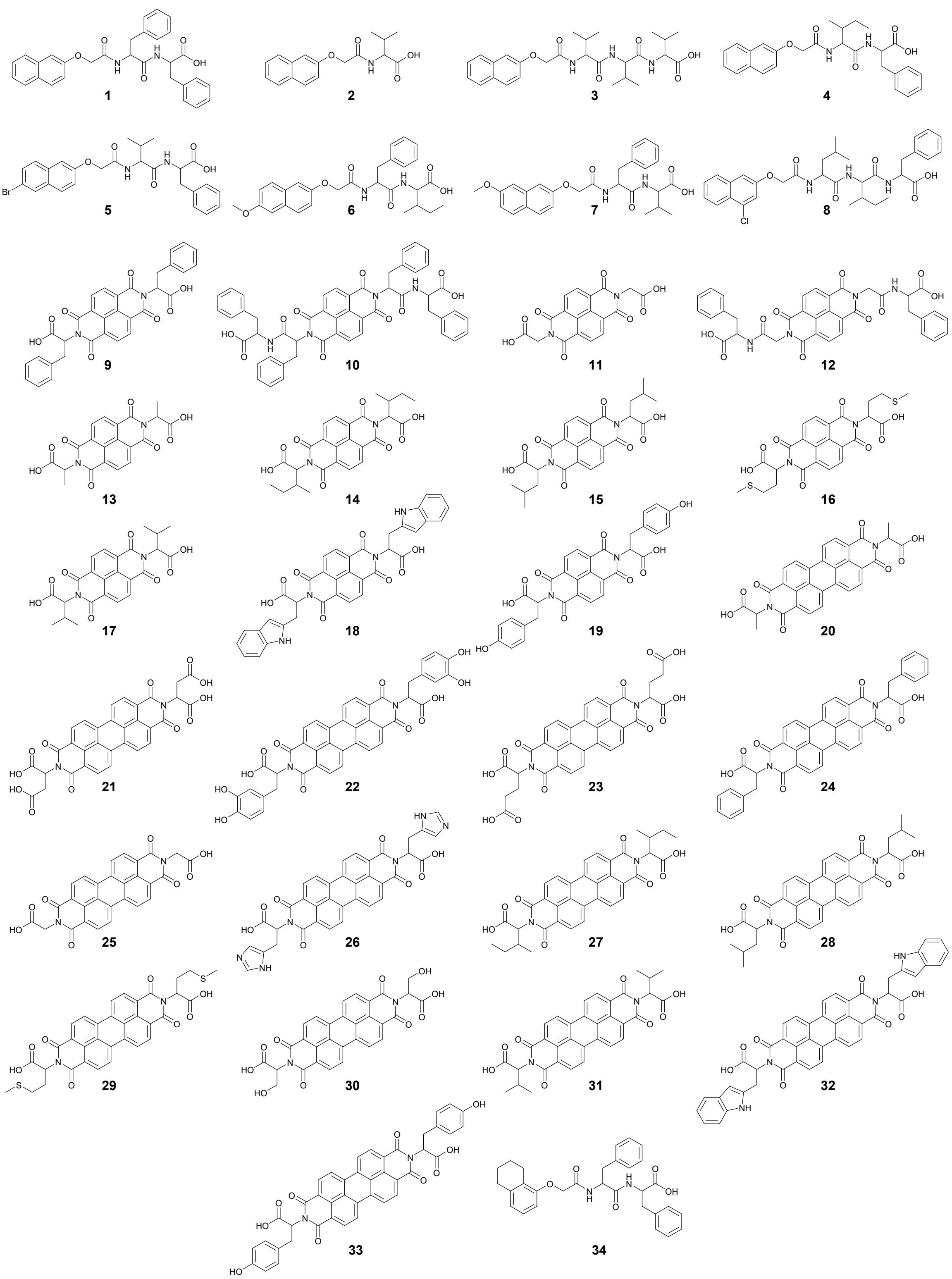

Figure S1. Previously reported compounds **1** – **34**

This journal is © The Royal Society of Chemistry 20xx

Please do not adjust margins

All previously unreported naphthalene and benzotrifluoride derivatives were synthesised using our established routes by coupling the precursor acids with C-protected dipeptides, followed by deprotection of the C-terminus.[3, 19] ^{1}H and ^{13}C NMR spectra were recorded on a Bruker Avance III 400 MHz spectrometer. Chemical shifts (δ) are reported in parts per million (ppm) to the residual solvent peak ($CDCl_3$: 7.26 ppm for ^{1}H and 77.0 ppm for ^{13}C; DMSO-d_6: 2.50 ppm for ^{1}H and 39.52 ppm for ^{13}C). Coupling constants (*J*) are given to the nearest 0.1 Hertz. Peak multiplicities are reported as singlet (s), doublet (d), triplet (t), t (m), and/or broad (b). Peak assignments are reported as: H/C_{Nap} = naphthalene H or C; H/C_{Ar} = aromatic H or C. Amino acids are listed as A: alanine; F: phenylalanine; G: glycine; I: isoleucine; L: leucine; V: valine. High resolution accurate mass measurements (HRMS) were performed on an Agilent 6546 LC/QTOF (quadrupole-time of flight) instrument with an Agilent ESI or APCI source.

2Nap-VLOMe (P1)

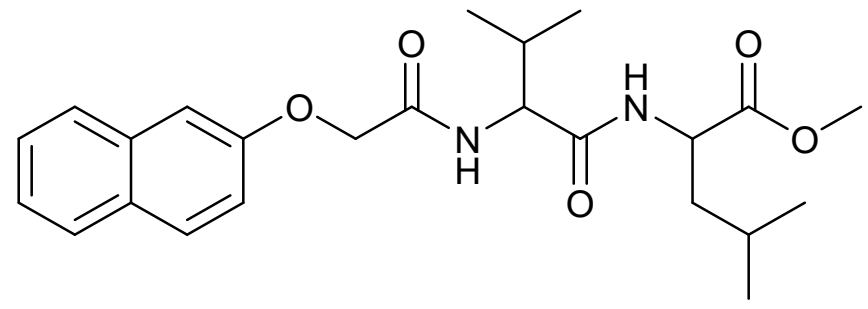

^{1}H NMR (400 MHz, DMSO-d_6) δ: 8.43 (1H, d, *J* 7.3, NH_L), 8.00 (1H, d, *J* 9.0, NH_V), 7.85 (2H, dd, *J* 8.6, 4.0, H_{Nap}), 7.75 (1H, d, *J* 8.2, H_{Nap}), 7.47 (1H, ddd, *J* 8.2, 6.8, 1.3, H_{Nap}), 7.37 (1H, ddd, *J* 8.1, 6.9, 1.3, H_{Nap}), 7.27 (1H, d, *J* 2.6, H_{Nap}), 7.24 (1H, dd, *J* 8.8, 2.6, H_{Nap}), 4.76–4.68 (2H, m, O-CH_2), 4.37–4.25 (2H, m, (NH-CH)$_2$), 3.61 (3H, s, O-CH_3), 2.03 (1H, oct., *J* 6.8, CH_V-CH_{3V}), 1.66–1.43 (3H, m, CH_{2L}-CH_L), 0.88 (6H, t, *J* 7.0, CH_{3V} and CH_{3L}), 0.83 (1H, d, *J* 6.8, CH_{3V}), 0.79 (3H, d, *J* 6.4, CH_{3L}).
^{13}C NMR (101 MHz, DMSO-d_6) δ: 172.7 (C=O-O-CH_3), 170.8 (C=O_V), 167.3 (CH_2-C=O), 155.5 (C_{Nap}-O), 134.0 (C_{Nap}), 129.4 (CH_{Nap}), 128.7 (C_{Nap}), 127.5 (CH_{Nap}), 126.6 (CH_{Nap}), 126.5 (CH_{Nap}), 123.8 (CH_{Nap}), 118.5 (CH_{Nap}), 107.2 (CH_{Nap}), 66.7 (O-CH_2), 56.8 (NH_V-CH_V), 51.7 (O-CH_3), 50.3 (NH_L-CH_L), 39.1 (CH_{2L}), 30.9 (CH_V-CH_{3V}), 24.2 (CH_L-CH_{3L}), 22.7 (CH_{3L}), 21.2 (CH_{3V}), 19.0 (CH_{3L}), 17.8 (CH_{3V}).
HRMS (ESI/QTOF) *m/z*: $[M+H]^+$ calcd for $C_{24}H_{33}N_2O_5$ 429.2384 (100%), 430.2416 (27%); found 429.2393 (100%), 430.2420 (27%).

2Nap-VL (35)

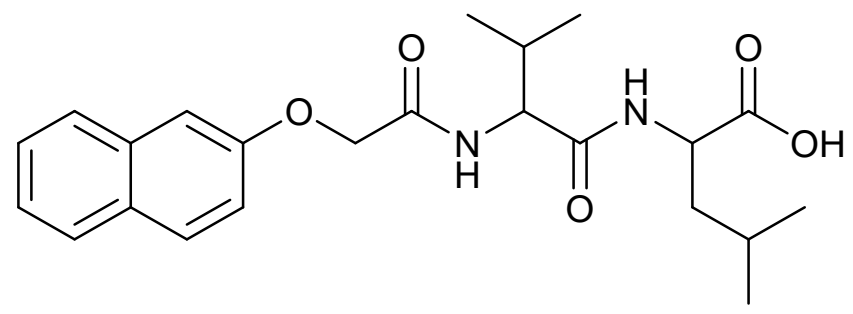

^{1}H NMR (400 MHz, DMSO-d_6) δ: 12.41 (1H, bs, COOH), 8.19 (1H, d, *J* 7.7, NH_L), 7.87 (1H, d, *J* 9.1, NH_V), 7.77–7.72 (2H, m, H_{Nap}), 7.64 (1H, d, *J* 8.3, H_{Nap}), 7.36 (1H, ddd, *J* 8.2, 6.9, 1.3, H_{Nap}), 7.26 (1H, ddd, *J* 8.1, 6.9, 1.2, H_{Nap}), 7.17 (1H, d, *J* 2.6, H_{Nap}), 7.13 (1H, dd, *J* 8.9, 2.6, H_{Nap}), 4.65–4.57 (2H, m, O-CH_2), 4.23 (1H, dd, *J* 9.0, 6.5, NH_V-CH_V), 4.11 (1H, ddd, *J* 9.7, 7.7, 5.4, NH_L-CH_L), 1.93 (1H, oct., J 6.7, CH_V-CH_{3V}), 1.56–1.34 (3H, m, CH_{2V}-CH_V-CH_{3V}), 0.77 (6H, t, *J* 6.4, CH_{3V} and CH_{3L}), 0.72 (3H, d, *J* 6.8, CH_{3V}), 0.69 (3H, d, *J* 6.4, CH_{3L}).
^{13}C NMR (101 MHz, DMSO-d_6) δ: 173.8 (COOH), 170.7 (C=O_V), 167.2 (CH_2-C=O), 155.5 (C_{Nap}-O), 134.0 (C_{Nap}), 129.4 (CH_{Nap}), 128.7 (CNap), 127.5 (CH_{Nap}), 126.6 (CH_{Nap}), 126.5 (CH_{Nap}), 123.8 (CH_{Nap}), 118.5 (CH_{Nap}), 107.3 (CH_{Nap}), 66.7 (O-CH_2), 56.8 (NH_V-CH_V), 50.2 (NH_L-CH_L), 39.5 (CH_{2L}), 31.0 (CH_V-CH_{3V}), 24.2 (CH_L-CH_{3L}), 22.8 (CH_{3L}), 21.2 (CH_{3L}), 19.1 (CH_{3V}), 17.8 (CH_{3V}).
HRMS (ESI/QTOF) *m/z*: $[M+Na]^+$ calcd for $C_{23}H_{30}N_2NaO_5$ 437.2047 (100%), 438.2079 (26%); found 437.2057 (100%), 428.2084 (25%).

2Nap-FLOMe (P2)

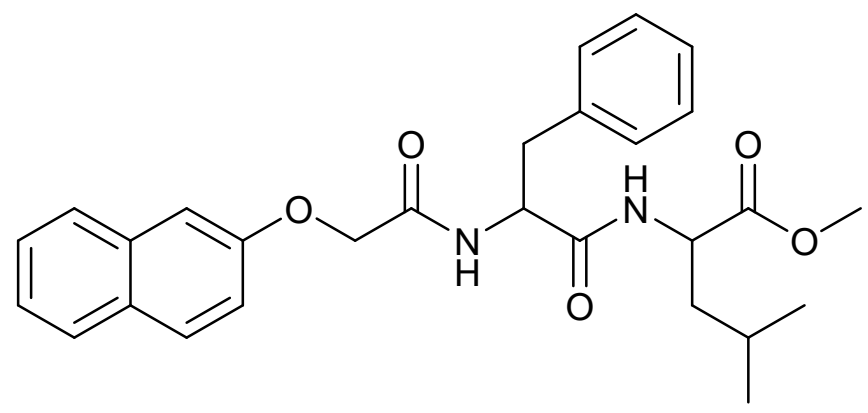

^{1}H NMR (400 MHz, DMSO-d_6) δ: 8.50 (1H, d, *J* 7.7, NH_L), 8.20 (1H, d, *J* 8.5, NH_F), 7.86–7.81 (2H, m, H_{Nap}), 7.72 (1H, d, *J* 8.2, H_{Nap}), 7.46 (1H, ddd, *J* 8.3, 6.8, 1.3, H_{Nap}), 7.36 (1H, ddd, *J* 8.0, 6.8, 1.3, H_{Nap}), 7.26–7.13 (7H, m, H_{Nap} and H_{ArF}), 4.67 (1H, td, *J* 9.4, 4.3, CH_F), 4.57 (2H, s, O-CH_2), 4.32 (1H, ddd, *J* 9.3, 7.6, 5.4, NH_L-CH_L), 3.63 (3H, s, O-CH_3), 3.06 (1H, dd, *J* 13.8, 4.3, 1/2CH_{2F}), 2.89 (1H, dd, *J* 13.8, 9.5, 1/2CH_{2F}), 1.67–1.46 (3H, m, CH_{2L}-CH_L), 0.87 (3H, d, *J* 6.3, CH_{3L}), 0.81 (3H, d, *J* 6.3, CH_{3L}).
^{13}C NMR (101 MHz, DMSO-d_6) δ: 172.7 (C=O_L), 171.0 (C=O_F), 167.3 (CH_2-C=O), 155.5 (C_{Nap}-O), 137.5 (C_{ArF}), 134.0 (C_{Nap}), 129.3 (CH_{Nap}), 129.2 (CH_{ArF}), 128.7 (C_{Nap}), 128.0 (CH_{ArF}), 127.5 (CH_{Nap}), 126.7 (CH_{Nap}), 126.4 (CH_{Nap}), 126.3 (CH_{ArF}), 123.8 (CH_{Nap}), 118.4 (CH_{Nap}), 107.3 (CH_{Nap}), 66.7 (O-CH_2), 53.2 (CH_F), 51.8 (O-CH_3), 50.3 (NH_L-CH_L), 37.4 (CH_{2F}), 24.2 (CH_L-CH_{3L}), 22.7 (CH_{3L}), 21.3 (CH_{3L}).
HRMS (ESI/QTOF) *m/z*: $[M+Na]^+$ calcd for $C_{28}H_{32}N_2NaO_5$ 499.2203 (100%), 500.2236 (32%); found 499.2209 (100%), 500.2241 (34%).

2Nap-FL (36)

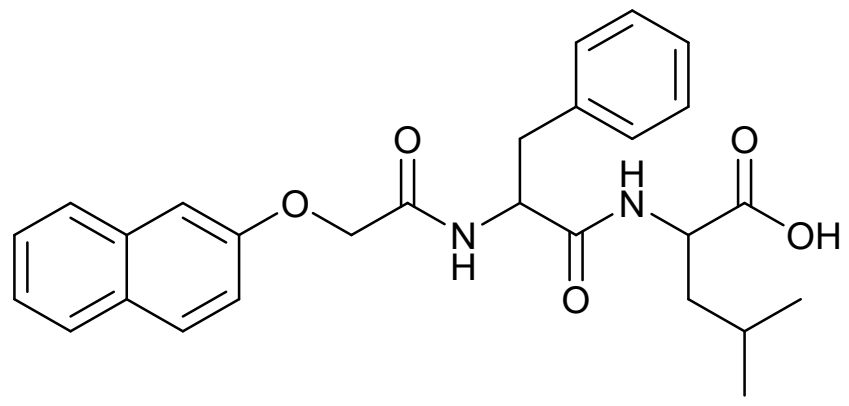

^{1}H NMR (400 MHz, DMSO-d_6) δ: 12.61 (1H, s, COOH), 8.37 (1H, d, *J* 8.0, NH_L), 8.16 (1H, d, *J* 8.5, NH_F), 7.86–7.81 (2H, m, H_{Nap}), 7.72 (1H, d, *J* 8.3, H_{Nap}), 7.46 (1H, ddd, *J* 8.2, 6.8, 1.3, H_{Nap}), 7.36 (1H, ddd, *J* 8.2, 6.9, 1.3, H_{Nap}), 7.27–7.22 (2H, m, H_{ArF}), 7.21–7.12 (5H, m, H_{ArF} and H_{Nap}), 4.67 (1H, td, *J* 9.1, 4.0, CH_F), 4.56 (2H, s, O-CH_2), 4.26 (1H, td, *J* 8.4, 6.0, NH_L-CH_L), 3.07 (1H, dd, *J* 13.9, 4.1, 1/2CH_{2F}), 2.88 (1H, dd, *J* 13.9, 9.6, 1/2CH_{2F}), 1.69–1.47 (3H, m, CH_{2L}-CH_L-CH_{3L}), 0.88 (3H, d, *J* 6.5, CH_{3L}), 0.81 (3H, d, *J* 6.4, CH_{3L}).
^{13}C NMR (101 MHz, DMSO-d_6) δ: 173.9 (COOH), 170.9 ($C=O_F$), 167.3 (CH_2-C=O), 155.5 (C_{Nap}-O), 137.5 (C_{ArF}), 134.0 (C_{Nap}), 129.3 (), 129.2 (), 128.7 (), 128.0 (), 127.5 (), 126.7 (), 126.4 (), 126.2 (), 123.8 (CH_{Nap}), 118.4 (CH_{Nap}), 107.3 (CH_{Nap}), 66.7 (O-CH_2), 53.2 (CH_F), 50.3 (NH_L-CH_L), 39.9 (CH_{2L}), 37.5 (CH_{2F}), 24.2 (CH_L-CH_{3L}), 22.8 (CH_{3L}), 21.3 (CH_{3L}).
HRMS (ESI/QTOF) *m/z*: $[M+Na]^+$ calcd for $C_{27}H_{30}N_2NaO_5$ 485.2065 (100%), 486.2079 (30%); found 485.2056 (100%), 486.2083 (29%).

2Nap-FIOMe (P3)

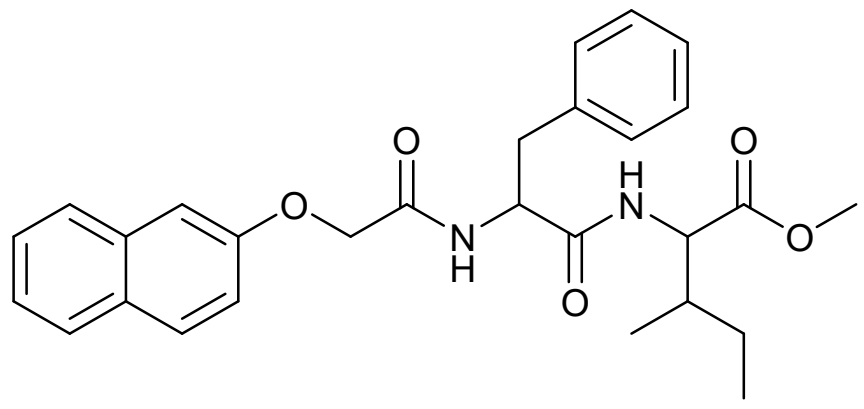

^{1}H NMR (400 MHz, DMSO-d_6) δ: 8.55 (1H, d, *J* 8.0, NH_I), 8.34 (1H, d, *J* 8.4, NH_F), 8.01–7.95 (2H, m, H_{Nap}), 7.87 (1H, d, *J* 8.3, H_{Nap}), 7.61 (1H, ddd, *J* 8.2, 6.8, 1.3, H_{Nap}), 7.51 (1H, ddd, *J* 8.2, 6.9, 1.2, H_{Nap}), 7.41–7.27 (7H, m, H_{Nap} and H_{ArF}), 4.89 (1H, ddd, *J* 9.4, 8.3, 4.4, CH_F), 4.72 (2H, s, O-CH_2), 4.39 (1H, dd, *J* 8.0, 6.4, NH_I-CH_I), 3.78 (3H, s, O-CH_3), 3.17 (1H, dd, *J* 13.9, 4.4, 1/2CH_{2F}), 3.03 (1H, dd, *J* 13.8, 9.5, 1/2CH_{2F}), 1.98–1.86 (1H, m, CH_I-CH_{3I}), 1.60–1.48 (1H, m, 1/2CH_{2I}), 1.37–1.24 (1H, m, 1/2CH_{2I}), 1.00–0.91 (6H, m, CH_{3I}).
^{13}C NMR (101 MHz, DMSO-d_6) δ: 171.7 ($C=O_I$), 171.1 ($C=O_F$), 167.3 (CH_2-C=O), 155.5 (C_{Nap}-O), 137.4 (C_{ArF}), 134.0 (C_{Nap}), 129.3 (CH_{Nap}), 129.2 (CH_{ArF}), 128.7 (C_{Nap}), 127.9 (CH_{ArF}), 127.5 (CH_{Nap}), 126.7 (CH_{Nap}), 126.4 (CH_{Nap}), 126.2 (CH_{ArF}), 123.8 (CH_{Nap}), 118.4 (CH_{Nap}), 107.3 (CH_{Nap}), 66.7 (O-CH_2), 56.4 (NH_I-CH_I), 53.1 (CH_F), 51.6 (O-CH_3), 37.5 (CH_{2F}), 36.3 (CH_I-CH_{3I}), 24.7 (CH_{2I}), 15.3 (CH_I-CH_{3I}), 11.1 (CH_{2I}-CH_{3I}).
HRMS (ESI/QTOF) *m/z*: $[M+Na]^+$ calcd for $C_{28}H_{32}N_2NaO_5$ 499.2203 (100%), 500.2236 (32%); found 499.2203 (100%), 500.2234 (30%).

2Nap-FI (37)

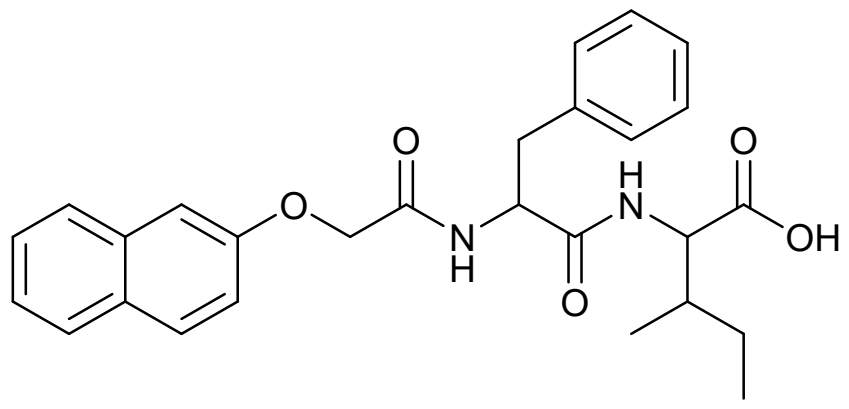

^{1}H NMR (400 MHz, DMSO-d_6) δ: 12.67 (1H, bs, COOH), 8.25 (1H, d, *J* 8.5, NH_I), 8.18 (1H, d, J 8.5, NH_F), 7.85–7.81 (2H, m, H_{Nap}), 7.72 (1H, d, *J* 8.3, H_{Nap}), 7.46 (1H, ddd, *J* 8.2, 6.8, 1.3, H_{Nap}), 7.36 (1H, ddd, *J* 8.1, 6.9, 1.2, H_{Nap}), 7.26–7.22 (2H, m, H_{Nap} and H_{ArF}), 7.20–7.11 (5H, m, H_{Nap} and H_{ArF}), 4.75 (1H, ddd, *J* 9.5, 8.3, 4.1, CH_F), 4.56 (2H, s, O-CH_2), 4.21 (1H, dd, *J* 8.4, 5.9, NH_I-CH_I), 3.04 (1H, dd, *J* 13.9, 4.2, 1/2CH_{2F}), 2.89 (1H, dd, *J* 13.8, 9.5, 1/2CH_{2F}), 1.83–1.73 (1H, m, CH_I-CH_{3I}), 1.46–1.36 (1H, m, 1/2CH_{2I}-CH_{3I}), 1.23–1.11 (1H, m, 1/2CH_{2I}-CH_{3I}), 0.89–0.79 (6H, m, CH_{3I}).
^{13}C NMR (101 MHz, DMSO-d_6) δ: 172.8 ($C=O_I$), 171.0 ($C=O_F$), 167.3 (CH_2-C=O), 155.5 (C_{Nap}-O), 137.5 (C_{ArF}), 134.0 (C_{Nap}), 129.33 (CH_{Nap}), 129.25 (CH_{ArF}), 128.7 (C_{Nap}), 127.9 (CH_{ArF}), 127.5 (CH_{Nap}), 126.8 (CH_{Nap}), 126.4 (CH_{Nap}), 126.2 (CH_{ArF}), 123.8 (CH_{Nap}), 118.4 (CH_{Nap}), 107.3 (CH_{Nap}), 66.7 (O-CH_2), 56.3 (NH_I-CH_I), 53.2 (CH_F), 37.5 (CH_{2F}), 36.4 (CH_I-CH_{3I}), 24.7 (CH_{2I}-CH_{3I}), 15.5 (CH_I-CH_{3I}), 11.3 (CH_{2I}-CH_{3I}).
HRMS (ESI/QTOF) *m/z*: $[M+H]^+$ calcd for $C_{27}H_{31}N_2O_5$ 463.2227 (100%), 464.2260 (30%); found 463.2238 (100%), 464.2268 (30%).

2Nap-VFOMe (P4)

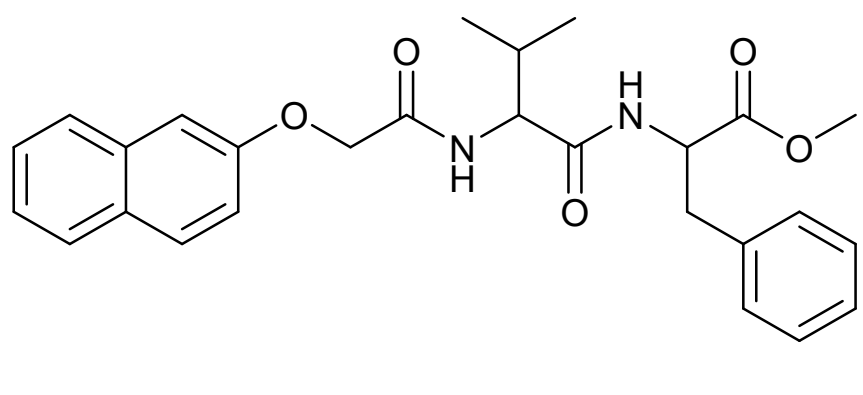

^{1}H NMR (400 MHz, DMSO-d_6) δ: 8.55 (1H, d, J 7.3, NH_F), 7.92–7.81 (3H, m, NH_V and H_{Nap}), 7.75 (1H, d, *J* 8.2, H_{Nap}), 7.46 (1H, ddd, *J* 8.2, 6.8, 1.5, H_{Nap}), 7.36 (1H, ddd, *J* 8.2, 6.8, 1.3, H_{Nap}), 7.29–7.14 (7H, m, H_{Nap} and H_{Ar}), 4.73–4.64 (2H, m, O-CH_2), 4.53–4.45 (1H, m, CH_F), 4.31 (1H, dd, *J* 9.2, 6.6, NH_V-CH_V), 3.56 (3H, s, O-CH_3), 3.02 (1H, dd, *J* 13.9, 6.0, 1/2CH_{2F}), 2.92 (1H, dd, *J* 13.9, 8.9, 1/2CH_{2F}), 1.99 (1H, oct., *J* 6.7, CH_V-CH_{3V}), 0.83 (3H, d, *J* 6.7, CH_{3V}), 0.78 (3H, d, *J* 6.7, CH_{3V}).
^{13}C NMR (101 MHz, DMSO-d_6) δ: 171.7 ($C=O_F$), 170.8 ($C=O_V$), 167.2 (CH_2-C=O), 155.5 (C_{Nap}-O), 137.0 (C_{Ar}), 134.0 (C_{Nap}), 129.4 (CH_{Nap}), 129.0 (CH_{Ar}), 128.7 (C_{Nap}), 128.2 (CH_{Ar}), 127.5 (CH_{Nap}), 126.6 (CH_{Nap}), 126.51 (CH_{Nap} or CH_{Ar}), 126.48 (CH_{Nap} or CH_{Ar}), 123.8 (CH_{Nap}), 118.4 (CH_{Nap}), 107.3 (CH_{Nap}), 66.7 (O-CH_2), 56.7 (NH_V-CH_V), 53.5 (CH_F), 51.7 (O-CH_3), 36.5 (CH_{2F}), 30.9 (CH_V-CH_{3V}), 19.0 (CH_{3V}), 17.7 (CH_{3V}).
HRMS (ESI/QTOF) *m/z*: $[M+H]^+$ calcd for $C_{27}H_{31}N_2O_5$ 463.2227 (100%), 464.2260 (30%); found 463.2228 (100%), 464.2260 (29%).

2Nap-VF (38)

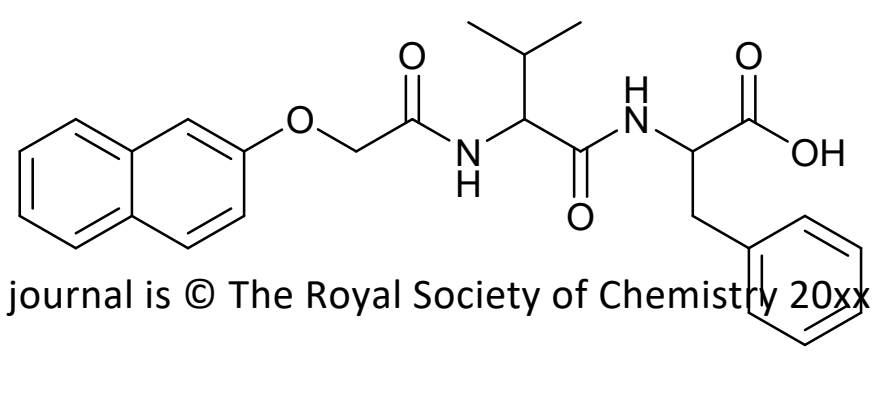

This journal is © The Royal Society of Chemistry 20xx

^{1}H NMR (400 MHz, DMSO-d$_6$) δ: 12.70 (1H, bs, COO$\underline{H}$), 8.41 (1H, d, J 7.7, N$\underline{H}_F$), 7.90 (1H, d, J 9.0, N$\underline{H}_V$), 7.87–7.82 (2H, m, $\underline{H}_{Nap}$), 7.75 (1H, d, J 8.2, $\underline{H}_{Nap}$), 7.46 (1H, ddd, J 8.1, 6.9, 1.2, $\underline{H}_{Nap}$), 7.36 (1H, ddd, J 8.0, 6.8, 1.2, $\underline{H}_{Nap}$), 7.29–7.13 (7H, m, $\underline{H}_{Nap}$ and $\underline{H}_{ArF}$), 4.73–4.64 (2H, m, O-C$\underline{H}_2$), 4.45 (1H, td, J 8.6, 6.2, 1.0, C$\underline{H}_F$), 4.31 (1H, dd, J 9.1, 6.5, NH$_V$-C$\underline{H}_V$), 3.05 (1H, dd, J 13.9, 5.3, 1/2C$\underline{H}_{2F}$), 2.89 (1H, dd, J 13.9, 9.2, 1/2C$\underline{H}_{2F}$), 2.00 (1H, oct., J 6.7, C$\underline{H}_V$-CH$_{3V}$), 0.82 (3H, d, J 6.7, C$\underline{H}_{3V}$), 0.77 (3H, d, J 6.7, C$\underline{H}_{3V}$).
^{13}C NMR (101 MHz, DMSO-d$_6$) δ: 172.7 ($\underline{C}$=O$_F$), 170.6 ($\underline{C}$=O$_V$), 167.2 (CH$_2$-$\underline{C}$=O), 155.5 ($\underline{C}_{Nap}$-O), 137.5 ($\underline{C}_{ArF}$), 134.0 ($\underline{C}_{Nap}$), 129.4 ($\underline{C}$H$_{Nap}$), 129.0 ($\underline{C}$H$_{ArF}$), 128.7 ($\underline{C}_{Nap}$), 128.1 ($\underline{C}$H$_{ArF}$), 127.5 ($\underline{C}$H$_{Nap}$), 126.6 ($\underline{C}$H$_{Nap}$), 126.5 ($\underline{C}$H$_{Nap}$), 126.4 ($\underline{C}$H$_{ArF}$), 123.8 ($\underline{C}$H$_{Nap}$), 118.5 ($\underline{C}$H$_{Nap}$), 107.3 ($\underline{C}$H$_{Nap}$), 66.7 (O-$\underline{C}$H$_2$), 56.8 ($\underline{C}$H$_V$), 53.4 ($\underline{C}$H$_F$), 36.6 ($\underline{C}$H$_{2F}$), 30.9 ($\underline{C}$H$_V$-CH$_{3V}$), 19.1 ($\underline{C}$H$_{3V}$), 17.7 ($\underline{C}$H$_{3V}$).
HRMS (ESI/QTOF) m/z: [M+H]$^+$ calcd for $C_{26}H_{28}N_2O_5$ 449.2071 (100%), 450.2103 (30%); found 449.2075 (100%), 450.2106 (29%).

2Nap-IVOMe (P5)

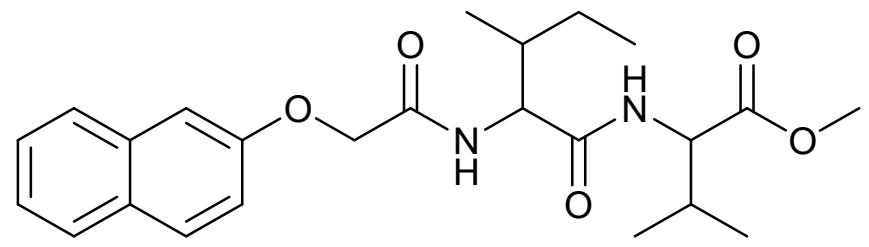

^{1}H NMR (400 MHz, DMSO-d$_6$) δ: 8.31 (1H, d, J 7.7, N$\underline{H}_V$), 8.00 (1H, d, J 9.0, N$\underline{H}_V$), 7.86–7.82 (2H, m, $\underline{H}_{Nap}$), 7.74 (1H, d, J 8.2, $\underline{H}_{Nap}$), 7.46 (1H, ddd, J 8.2, 6.9, 1.3, $\underline{H}_{Nap}$), 7.36 (1H, ddd, J 8.1, 6.9, 1.2, $\underline{H}_{Nap}$), 7.26–7.20 (2H, m, $\underline{H}_{Nap}$), 4.74–4.65 (2H, m, O-C$\underline{H}_2$), 4.42 (1H, dd, J 9.0, 7.6, NH$_I$-C$\underline{H}_I$), 4.14 (1H, dd, J 7.7, 6.4, NH$_V$-C$\underline{H}_V$), 3.61 (3H, s, O-C$\underline{H}_3$), 2.01 (1H, oct., J 6.7, C$\underline{H}_V$-CH$_{3V}$), 1.82–1.70 (1H, m, C$\underline{H}_I$-CH$_{3I}$), 1.48–1.37 (1H, m, 1/2C$\underline{H}_{2I}$-CH$_{3I}$), 1.10–0.98 (1H, m, 1/2C$\underline{H}_{2I}$-CH$_{3I}$), 0.88–0.81 (9H, m, CH$_I$-C$\underline{H}_{3I}$ and C$\underline{H}_{3V}$), 0.78 (3H, t, J 7.5, CH$_{2I}$-C$\underline{H}_{3I}$).
^{13}C NMR (101 MHz, DMSO-d$_6$) δ: 171.7 ($\underline{C}$=O$_V$), 171.2 ($\underline{C}$=O$_I$), 167.2 (CH$_2$-$\underline{C}$=O), 155.5 ($\underline{C}_{Nap}$-O), 134.0 ($\underline{C}_{Nap}$), 129.4 ($\underline{C}$H$_{Nap}$), 128.7 ($\underline{C}_{Nap}$), 127.5 ($\underline{C}$H$_{Nap}$), 126.6 ($\underline{C}$H$_{Nap}$), 126.4 ($\underline{C}$H$_{Nap}$), 123.8 ($\underline{C}$H$_{Nap}$), 118.5 ($\underline{C}$H$_{Nap}$), 107.2 ($\underline{C}$H$_{Nap}$), 66.7 (O-$\underline{C}$H$_2$), 57.5 (NH$_V$-$\underline{C}$H$_V$), 56.0 (NH$_I$-$\underline{C}$H$_I$), 51.5 (O-$\underline{C}$H$_3$), 37.0 ($\underline{C}$H$_I$-CH$_{3I}$), 29.6 ($\underline{C}$H$_V$-CH$_{3V}$), 24.1 ($\underline{C}$H$_{2I}$-CH$_{3I}$), 18.8 ($\underline{C}$H$_{3V}$), 18.3 ($\underline{C}$H$_{3V}$), 15.1 (CH$_I$-$\underline{C}$H$_{3I}$), 10.8 (CH$_{2I}$-$\underline{C}$H$_{3I}$).
HRMS (ESI/QTOF) m/z: [M+Na]$^+$ calcd for $C_{24}H_{32}N_2NaO_5$ 451.2203 (100%), 452.2236 (27%); found 451.2210 (100%), 452.2241 (28%).

2Nap-IV (39)

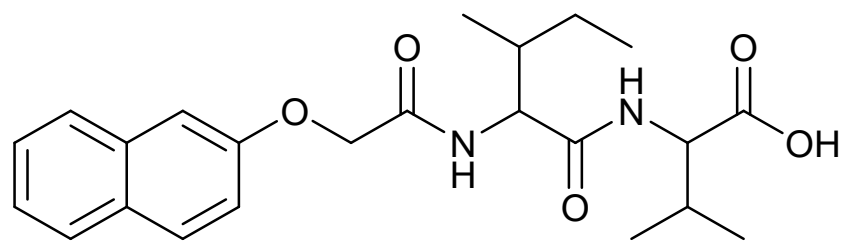

^{1}H NMR (400 MHz, DMSO-d$_6$) δ: 12.55 (1H, s, COO$\underline{H}$), 8.14 (1H, d, J 8.2, N$\underline{H}_V$), 8.00 (1H, d, J 9.0, N$\underline{H}_I$), 7.84 (2H, dd, J 8.8, 2.8, $\underline{H}_{Nap}$), 7.74 (1H, d, J 8.2, $\underline{H}_{Nap}$), 7.46 (1H, t, J 7.5, $\underline{H}_{Nap}$), 7.35 (1H, t, J 7.5, $\underline{H}_{Nap}$), 7.27–7.20 (2H, m, $\underline{H}_{Nap}$), 4.75–4.65 (2H, m, O-C$\underline{H}_2$), 4.42 (1H, t, J 8.3, NH$_I$-C$\underline{H}_I$), 4.11 (1H, dd, J 8.3, 5.9, NH$_V$-C$\underline{H}_V$), 2.02 (1H, oct., J 6.7, C$\underline{H}_V$-CH$_{3V}$), 1.83–1.71 (1H, m, C$\underline{H}_I$-CH$_{3I}$), 1.49–1.37 (1H, m, 1/2C$\underline{H}_{2I}$-CH$_{3I}$), 1.10–0.97 (1H, m, 1/2C$\underline{H}_{2I}$-CH$_{3I}$), 0.86 (9H, t, J 6.5, C$\underline{H}_{3V}$ and CH$_I$-C$\underline{H}_{3I}$), 0.77 (3H, t, J 7.4, CH$_{2I}$-C$\underline{H}_{3I}$).
^{13}C NMR (101 MHz, CDCl$_3$) δ: 172.7 ($\underline{C}$=O$_V$), 171.1 ($\underline{C}$=O$_I$), 167.2 (CH$_2$-$\underline{C}$=O), 155.6 ($\underline{C}_{Nap}$-O), 134.0 ($\underline{C}_{Nap}$), 129.4 ($\underline{C}$H$_{Nap}$), 128.7 ($\underline{C}_{Nap}$), 127.5 ($\underline{C}$H$_{Nap}$), 126.6 ($\underline{C}$H$_{Nap}$), 126.4 ($\underline{C}$H$_{Nap}$), 123.8 ($\underline{C}$H$_{Nap}$), 118.5 ($\underline{C}$H$_{Nap}$), 107.3 ($\underline{C}$H$_{Nap}$), 66.7 (O-$\underline{C}$H$_2$), 57.3 (NH$_V$-$\underline{C}$H$_V$), 56.1 (NH$_I$-$\underline{C}$H$_I$), 37.0 ($\underline{C}$H$_I$-CH$_{3I}$), 29.6 ($\underline{C}$H$_V$-CH$_{3V}$), 24.1 ($\underline{C}$H$_{2I}$-CH$_{3I}$), 19.0 ($\underline{C}$H$_{3V}$), 18.1 ($\underline{C}$H$_{3V}$), 15.2 (CH$_I$-$\underline{C}$H$_{3I}$), 10.9 (CH$_{2I}$-$\underline{C}$H$_{3I}$).
HRMS (ESI/QTOF) m/z: [M+H]$^+$ calcd for $C_{23}H_{31}N_2O_5$ 415.2227 (100%), 416.2260 (26%); found 415.2238 (100%), 416.2265 (25%).

CF$_3$Ph-FLOMe (P6)

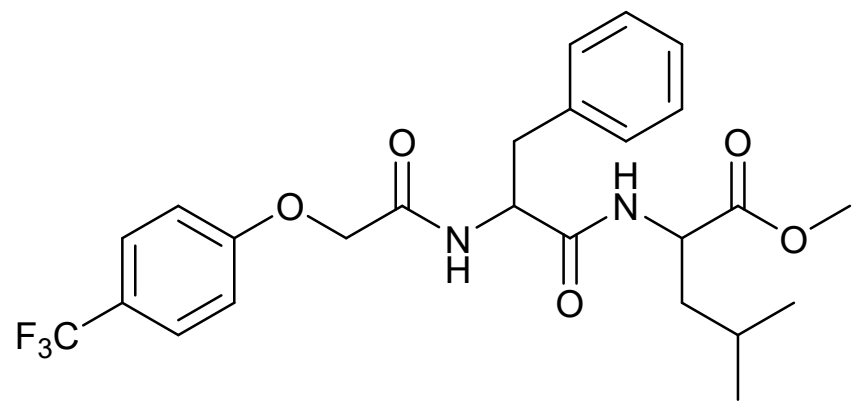

^{1}H NMR (400 MHz, DMSO-d$_6$) δ: 8.38 (1H, d, J 8.1, N$\underline{H}_V$), 8.25 (1H, d, J 8.5, N$\underline{H}_F$), 7.58 (1H, d, J 8.2, $\underline{H}_{Ar}$), 7.26–7.16 (5H, m, $\underline{H}_{ArF}$), 6.97 (2H, d, J 8.2, $\underline{H}_{Ar}$), 4.73 (1H, ddd, J 9.8, 8.4, 4.3, C$\underline{H}_F$), 4.55 (2H, s, O-C$\underline{H}_2$), 4.19 (1H, dd, J 8.1, 6.4, NH$_V$-C$\underline{H}_V$), 3.64 (3H, s, O-C$\underline{H}_3$), 3.02 (1H, dd, J 13.8, 4.3, 1/2C$\underline{H}_{2F}$), 2.84 (1H, dd, J 13.8, 9.8, 1/2C$\underline{H}_{2F}$), 2.05 (1H, oct., J 6.8, C$\underline{H}_V$-CH$_{3V}$), 0.88 (6H, dd, J 9.2, 6.0, C$\underline{H}_{3V}$).
^{13}C NMR (101 MHz, DMSO-d$_6$) δ: 171.8 ($\underline{C}$=O$_V$), 171.2 ($\underline{C}$=O$_F$), 166.8 (CH$_2$-$\underline{C}$=O), 160.5 ($\underline{C}_{Ar}$-O), 137.5 ($\underline{C}_{ArF}$), 129.2 ($\underline{C}$H$_{ArF}$), 128.0 ($\underline{C}$H$_{ArF}$), 126.8 (1C, q, $^3J_{C\text{-}F}$ 3.8, CF$_3$-C$_{Ar}$-C$\underline{H}_{Ar}$), 126.2 ($\underline{C}$H$_{ArF}$), 124.5 (1C, q, $^1J_{C\text{-}F}$ 271, $\underline{C}$F$_3$), 121.5 (1C, q, $^2J_{C\text{-}F}$ 32.2, CF$_3$-$\underline{C}_{Ar}$), 115.0 ($\underline{C}$H$_{Ar}$), 66.5 (O-$\underline{C}$H$_2$), 57.5 (NH$_V$-$\underline{C}$H$_V$), 53.1 ($\underline{C}$H$_F$), 51.7 (O-$\underline{C}$H$_3$), 37.5 ($\underline{C}$H$_{2F}$), 29.9 ($\underline{C}$H$_V$-CH$_{3V}$), 18.9 ($\underline{C}$H$_{3V}$), 18.2 ($\underline{C}$H$_{3V}$).
HRMS (ESI/QTOF) m/z: [M+Na]$^+$ calcd for $C_{24}H_{27}F_3N_2NaO_5$ 503.1764 (100%), 504.1797 (27%); found 503.1777 (100%), 504.1809 (28%).

CF_3Ph-FL (40)

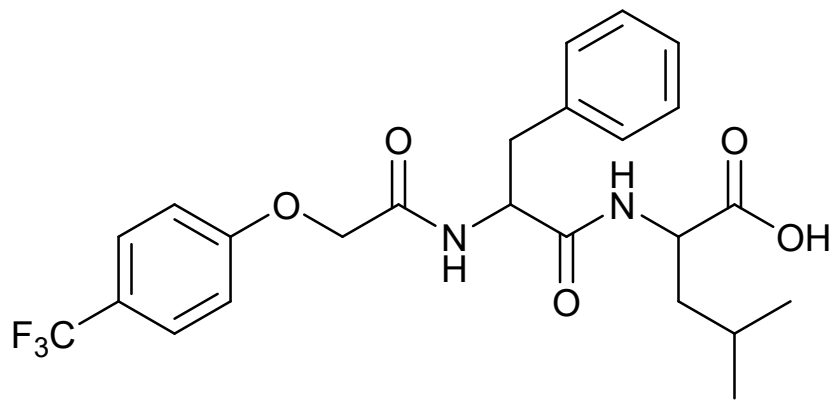

^{1}H NMR (400 MHz, DMSO-d_6) δ: 12.61 (1H, s, COO$\underline{H}$), 8.36 (1H, d, *J* 7.9, N$\underline{H}_L$), 8.23 (1H, d, *J* 8.6, N$\underline{H}_F$), 7.58 (2H, d, *J* 8.6, $\underline{H}_{Ar}$), 7.26−7.16 (5H, m, $\underline{H}_{ArF}$), 6.96 (2H, d, *J* 8.6, $\underline{H}_{Ar}$), 4.65 (1H, td, *J* 9.1, 3.9, C$\underline{H}_F$), 4.55 (2H, s, O-C$\underline{H}_2$), 4.25 (1H, dt, *J* 9.2, 6.0, NH_L-C$\underline{H}_L$), 3.06 (1H, dd, *J* 13.8, 4.0, 1/2C$\underline{H}_{2F}$), 2.83 (1H, dd, *J* 13.9, 10.0, 1/2C$\underline{H}_{2F}$), 1.68−1.49 (3H, m, C$\underline{H}_{2L}$-C$\underline{H}_L$-CH_{3L}), 0.90 (3H, d, *J* 6.4, C$\underline{H}_{3L}$), 0.84 (3H, d, *J* 6.3, C$\underline{H}_{3L}$).
^{13}C NMR (101 MHz, DMSO-d_6) δ: 173.9 ($\underline{C}$=O_L), 170.9 ($\underline{C}$=O_F), 166.8 (CH_2-$\underline{C}$=O), 160.5 ($\underline{C}_{Ar}$-O), 137.6 ($\underline{C}_{ArF}$), 129.2 ($\underline{C}H_{ArF}$), 128.0 ($\underline{C}H_{ArF}$), 126.8 (1C, q, $^3J_{C\text{-}F}$ 3.9, CF_3-C_{Ar}-C$\underline{H}_{Ar}$), 126.2 ($\underline{C}H_{ArF}$), 124.5 (1C, q, $^1J_{C\text{-}F}$ 271.1, $\underline{C}F_3$), 121.7 (1C, q, $^2J_{C\text{-}F}$ 32.3, CF_3-$\underline{C}_{Ar}$), 115.0 ($\underline{C}H_{Ar}$), 66.5 (O-$\underline{C}H_2$), 53.2 ($\underline{C}H_F$), 50.3 (NH_L-C$\underline{H}_L$), 39.9 ($\underline{C}H_{2L}$-CH_L-CH_{3L}), 37.5 ($\underline{C}H_{2F}$), 24.2 ($\underline{C}H_L$-CH_{3L}), 22.8 ($\underline{C}H_{3L}$), 21.3 ($\underline{C}H_{3L}$).
HRMS (ESI/QTOF) *m/z*: $[M+H]^+$ calcd for $C_{24}H_{28}F_3N_2O_5$ 481.1945 (100%), 482.1977 (27%); found 481.1947 (100%), 482.1980 (28%).

CF_3PH-FVOMe (P7)

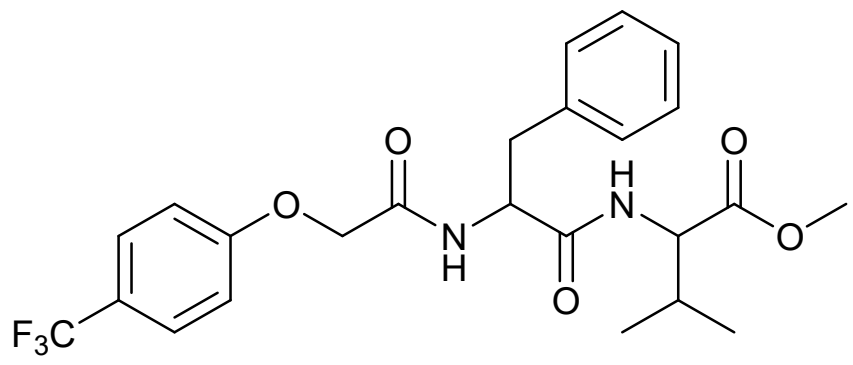

^{1}H NMR (400 MHz, DMSO-d_6) δ: 8.38 (1H, d, *J* 8.1, N$\underline{H}_V$), 8.25 (1H, d, *J* 8.5, N$\underline{H}_F$), 7.58 (1H, d, *J* 8.2, $\underline{H}_{Ar}$), 7.26−7.16 (5H, m, $\underline{H}_{ArF}$), 6.97 (2H, d, *J* 8.2, $\underline{H}_{Ar}$), 4.73 (1H, ddd, *J* 9.8, 8.4, 4.3, C$\underline{H}_F$), 4.55 (2H, s, O-C$\underline{H}_2$), 4.19 (1H, dd, *J* 8.1, 6.4, NH_V-C$\underline{H}_V$), 3.64 (3H, s, O-C$\underline{H}_3$), 3.02 (1H, dd, *J* 13.8, 4.3, 1/2C$\underline{H}_{2F}$), 2.84 (1H, dd, *J* 13.8, 9.8, 1/2C$\underline{H}_{2F}$), 2.05 (1H, oct., *J* 6.8, C$\underline{H}_V$-CH_{3V}), 0.88 (6H, dd, *J* 9.2, 6.0, C$\underline{H}_{3V}$).
^{13}C NMR (101 MHz, DMSO-d_6) δ: 171.8 ($\underline{C}$=O_V), 171.2 ($\underline{C}$=O_F), 166.8 (CH_2-$\underline{C}$=O), 160.5 ($\underline{C}_{Ar}$-O), 137.5 ($\underline{C}_{ArF}$), 129.2 ($\underline{C}H_{ArF}$), 128.0 ($\underline{C}H_{ArF}$), 126.8 (1C, q, $^3J_{C\text{-}F}$ 3.8, CF_3-C_{Ar}-C$\underline{H}_{Ar}$), 126.2 ($\underline{C}H_{ArF}$), 124.5 (1C, q, $^1J_{C\text{-}F}$ 271, $\underline{C}F_3$), 121.5 (1C, q, $^2J_{C\text{-}F}$ 32.2, CF_3-$\underline{C}_{Ar}$), 115.0 ($\underline{C}H_{Ar}$), 66.5 (O-$\underline{C}H_2$), 57.5 (NH_V-$\underline{C}H_V$), 53.1 ($\underline{C}H_F$), 51.7 (O-$\underline{C}H_3$), 37.5 ($\underline{C}H_{2F}$), 29.9 ($\underline{C}H_V$-CH_{3V}), 18.9 ($\underline{C}H_{3V}$), 18.2 ($\underline{C}H_{3V}$).
HRMS (ESI/QTOF) *m/z*: $[M+Na]^+$ calcd for $C_{24}H_{27}F_3N_2NaO_5$ 503.1764 (100%), 504.1797 (27%); found 503.1777 (100%), 504.1809 (28%).

CF_3Ph-FV (41)

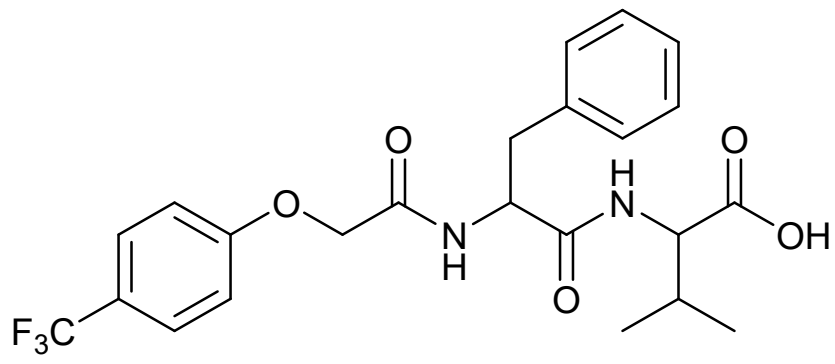

^{1}H NMR (400 MHz, DMSO-d_6) δ: 12.71 (1H, bs, COO$\underline{H}$), 8.25 (2H, dd, *J* 8.5, 4.4, N$\underline{H}_F$ and N$\underline{H}_V$), 7.58 (2H, d, *J* 9.0, $\underline{H}_{Ar}$), 7.27−7.15 (5H, m, $\underline{H}_{ArF}$), 6.95 (2H, d, *J* 8.3, $\underline{H}_{Ar}$), 4.74 (1H, ddd, *J* 10.0, 8.5, 4.0, C$\underline{H}_F$), 4.55 (2H, s, O-C$\underline{H}_2$), 4.17 (1H, dd, *J* 8.5, 5.8, NH_V-C$\underline{H}_V$), 3.04 (1H, dd, *J* 13.8, 4.0, 1/2C$\underline{H}_{2F}$), 2.84 (1H, dd, *J* 13.8, 10.0, 1/2C$\underline{H}_{2F}$), 2.07 (1H, oct., *J* 5.7, C$\underline{H}_V$-CH_{3V}), 0.88 (6H, dd, *J* 6.9, 4.1, C$\underline{H}_{3V}$).
^{13}C NMR (101 MHz, DMSO-d_6) δ: 172.8 ($\underline{C}$=O_V), 171.1 ($\underline{C}$=O_F), 166.8 (CH_2-$\underline{C}$=O), 160.5 ($\underline{C}_{Ar}$-O), 137.5 ($\underline{C}_{ArF}$), 129.3 ($\underline{C}H_{ArF}$), 128.0 ($\underline{C}H_{ArF}$), 126.8 (1C, q, $^3J_{C\text{-}F}$ 3.9, CF_3-C_{Ar}-C$\underline{H}_{Ar}$), 126.2 ($\underline{C}H_{ArF}$), 124.5 (1C, q, $^1J_{C\text{-}F}$ 271.1, $\underline{C}F_3$), 121.6 (1C, q, $^2J_{C\text{-}F}$ 32.1, CF_3-$\underline{C}_{Ar}$), 115.1 ($\underline{C}H_{Ar}$), 66.6 (O-$\underline{C}H_2$), 57.2 (NH_V-$\underline{C}H_V$), 53.2 ($\underline{C}H_F$), 37.5 ($\underline{C}H_{2F}$), 29.9 ($\underline{C}H_V$-CH_{3V}), 19.1 ($\underline{C}H_{3V}$), 18.0 ($\underline{C}H_{3V}$).
HRMS (APCI/QTOF) *m/z*: $[M+H]^+$ calcd for $C_{23}H_{26}F_3N_2O_5$ 467.1788 (100%), 468.1821 (26%); found 467.1800 (100%), 468.1826 (25%).

2Nap-VIOMe (P8)

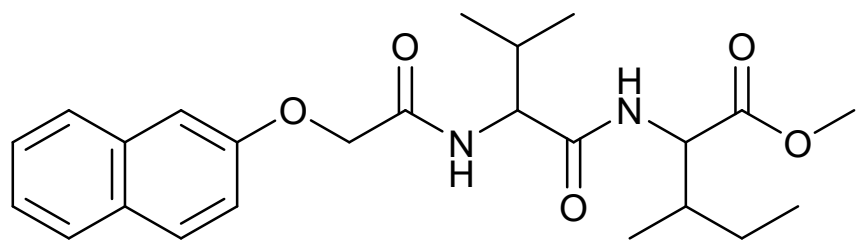

^{1}H NMR (400 MHz, DMSO-d_6) δ: 8.32 (1H, d, *J* 7.5, N$\underline{H}_I$), 7.98 (1H, d, *J* 9.0, N$\underline{H}_V$), 7.85 (1H, d, *J* 4.0, $\underline{H}_{Nap}$), 7.83 (1H, d, *J* 2.8, $\underline{H}_{Nap}$), 7.74 (1H, d, *J* 8.1, $\underline{H}_{Nap}$), 7.46 (1H, t, *J* 7.6, $\underline{H}_{Nap}$), 7.36 (1H, t, *J* 7.6, $\underline{H}_{Nap}$), 7.26 (1H, d, *J* 2.6, $\underline{H}_{Nap}$), 7.23 (1H, dd, *J* 8.8, 2.6, $\underline{H}_{Nap}$), 4.75−4.67 (2H, m, O-C$\underline{H}_2$), 4.41 (1H, dd, *J* 9.0, 6.6, NH_V-C$\underline{H}_V$), 4.18 (1H, dd, *J* 7.4, 6.4, NH_I-C$\underline{H}_I$), 3.61 (3H, s, O-C$\underline{H}_3$), 2.00 (1H, oct., *J* 6.8, C$\underline{H}_V$-CH_{3V}), 1.82−1.70 (1H, m, C$\underline{H}_I$-CH_{3I}), 1.44−1.32 (1H, m, 1/2C$\underline{H}_{2I}$-CH_{3I}), 1.24−1.10 (1H, m, 1/2C$\underline{H}_{2I}$-CH_{3I}), 0.87 (3H, d, *J* 6.8, C$\underline{H}_{3V}$), 0.84−0.77 (9H, m, C$\underline{H}_{3V}$ and C$\underline{H}_{3I}$).
^{13}C NMR (101 MHz, DMSO-d_6) δ: 171.7 ($\underline{C}$=O_I), 171.0 ($\underline{C}$=O_V), 167.3 (CH_2-$\underline{C}$=O), 155.5 (O-$\underline{C}_{Nap}$), 134.0 ($\underline{C}_{Nap}$), 129.4 ($\underline{C}H_{Nap}$), 128.7 ($\underline{C}_{Nap}$), 127.5 ($\underline{C}H_{Nap}$), 126.6 ($\underline{C}H_{Nap}$), 126.5 ($\underline{C}H_{Nap}$), 123.8 ($\underline{C}H_{Nap}$), 118.5 ($\underline{C}H_{Nap}$), 107.3 ($\underline{C}H_{Nap}$), 66.7 (O-$\underline{C}H_2$), 56.6 (NH_V-$\underline{C}H_V$), 56.5 (NH_I-$\underline{C}H_I$), 51.5 (O-$\underline{C}H_3$), 36.0 ($\underline{C}H_I$-CH_{3I}), 31.0 ($\underline{C}H_V$-CH_{3V}), 24.8 ($\underline{C}H_{2I}$-CH_{3I}), 19.0 ($\underline{C}H_{3V}$), 17.9 ($\underline{C}H_{3V}$), 15.3 (CH_I-$\underline{C}H_{3I}$), 11.1 (CH_{2I}-$\underline{C}H_{3I}$).
HRMS (APCI/QTOF) *m/z*: $[M+H]^+$ calcd for $C_{24}H_{33}N_2O_5$ 429.2384 (100%), 430.2416 (27%); found 429.2393 (100%), 430.2420 (27%).

This journal is © The Royal Society of Chemistry 20xx
Please do not adjust margins

2Nap-VI (42)

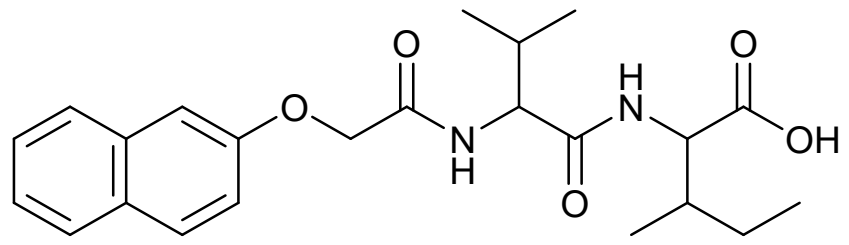

^{1}H NMR (400 MHz, DMSO-d_6) δ: 12.53 (1H, s, COO$\underline{H}$), 8.16 (1H, d, *J* 7.9, N$\underline{H}_{I}$), 7.97 (1H, d, *J* 9.0, N$\underline{H}_{V}$), 7.85 (1H, d, *J* 4.4, $\underline{H}_{Nap}$), 7.83 (1H, d, *J* 3.7, $\underline{H}_{Nap}$), 7.74 (1H, d, *J* 8.3, $\underline{H}_{Nap}$), 7.46 (1H, ddd, *J* 8.3, 6.9, 1.3, $\underline{H}_{Nap}$), 7.36 (1H, ddd, *J* 8.2, 6.9, 1.3, $\underline{H}_{Nap}$), 7.27 (1H, d, *J* 2.6, $\underline{H}_{Nap}$), 7.23 (1H, dd, *J* 8.9, 2.6, $\underline{H}_{Nap}$), 4.75–4.67 (2H, m, O-C$\underline{H}_2$), 4.41 (1H, dd, *J* 9.0, 6.5, NH$_V$-C$\underline{H}_V$), 4.15 (1H, dd, *J* 8.0, 6.0, NH$_I$-C$\underline{H}_I$), 2.01 (1H, oct., *J* 6.7, C$\underline{H}_V$-CH$_{3V}$), 1.81–1.70 (1H, m, C$\underline{H}_I$-CH$_{3I}$), 1.46–1.33 (1H, m, 1/2C$\underline{H}_{2I}$-CH$_{3I}$), 1.24–1.10 (1H, m, 1/2C$\underline{H}_{2I}$-CH$_{3I}$), 0.87 (3H, d, *J* 6.8, C$\underline{H}_{3V}$), 0.84–0.78 (9H, m, C$\underline{H}_{3V}$ and C$\underline{H}_{3I}$).
^{13}C NMR (101 MHz, DMSO-d_6) δ: 172.7 ($\underline{C}$=O$_I$), 170.8 ($\underline{C}$=O$_V$), 167.2 (CH$_2$-$\underline{C}$=O), 155.5 ($\underline{C}_{Nap}$-O), 134.0 ($\underline{C}_{Ar}$), 129.4 ($\underline{C}$H$_{Ar}$), 128.7 ($\underline{C}_{Ar}$), 127.5 ($\underline{C}$H$_{Ar}$), 126.6 ($\underline{C}$H$_{Ar}$), 126.5 ($\underline{C}$H$_{Ar}$), 123.8 ($\underline{C}$H$_{Ar}$), 118.5 ($\underline{C}$H$_{Ar}$), 107.3 ($\underline{C}$H$_{Ar}$), 66.7 (O-$\underline{C}$H$_2$), 56.7 (NH$_V$-$\underline{C}$H$_V$), 56.3 (NH$_I$-$\underline{C}$H$_I$), 36.1 ($\underline{C}$H$_I$-CH$_{3I}$), 31.0 ($\underline{C}$H$_V$-CH$_{3V}$), 24.7 ($\underline{C}$H$_{2I}$-CH$_{3I}$), 19.1 ($\underline{C}$H$_{3V}$), 17.9 ($\underline{C}$H$_{3V}$), 15.5 (CH$_I$-$\underline{C}$H$_{3I}$), 11.2 (CH$_{2I}$-$\underline{C}$H$_{3I}$).
HRMS (APCI/QTOF) *m/z*: [M+H]$^+$ calcd for $C_{23}H_{31}N_2O_5$ 415.2227 (100%), 416.2260 (26%); found 415.2245 (100%), 416.2272 (26%).

All previously unreported asymmetric alkyl / amino acid functionalised naphthalene diimide derivatives were synthesised by coupling the precursor with the alkyl amine using a procedure adapted from previous reporting,[20] followed by the dehydration of the diacid to the monoanhydride, and finally coupling the amino acid as described below.

Naphthelene monoimide diacid (P9)

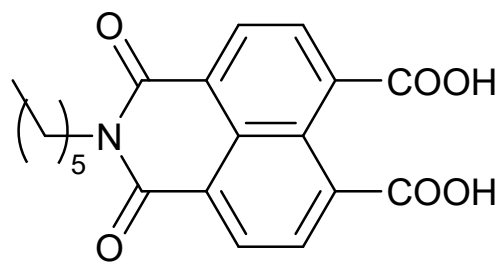

Naphthalenetetracarboxylic acid dianhydride (NTCDA, 7.50 g, 28.0 mmol) was dissolved in a solution of potassium hydroxide (6.2 eq, 9.73 g) in water (1.5 L). The pH was adjusted to 6.3 (target 6.2 to 6.4) with phosphoric acid, *n*-hexylamine (1 eq, 3.70 mL) was added, and the pH was again adjusted to 6.3. The mixture was then heated at reflux for ca. 24 h. After cooling to room temperature, it was filtered (celite or fine-pore filter may be necessary to remove an extremely fine suspension) and acidified to pH 5 with acetic acid. The precipitate was filtered off, washed with water, and dried by freeze drying to give **P9** in sufficient purity as a light brown/cream solid (8.25 g, 80%).

^{1}H NMR (400 MHz, DMSO-d_6) δ: 8.51 (2H, d, *J* 7.5, $\underline{H}_{Ar}$), 8.07 (2H, d, *J* 7.5, $\underline{H}_{Ar}$), 4.03 (2H, t, *J* 7.4, NC$\underline{H}_2$), 1.69-1.58 (2H, m, C$\underline{H}_2$), 1.43-1.23 (6H, m, C$\underline{H}_2$), 0.86 (3H, t, *J* 7.0, C$\underline{H}_3$).
^{13}C NMR (101 MHz, DMSO-d_6) δ: 169.3 and 162.9 ($\underline{C}$=O), 138.8, 130.1, 128.5, 128.5, 125.5, and 123.8 ($\underline{C}_{Ar}$), 39.9, 31.0, 27.4, 26.2, and 22.0 (hexyl $\underline{C}$H$_2$), 13.9 ($\underline{C}$H$_3$).
HRMS (APCI/QTOF): [M+H]$^+$ calcd for $C_{20}H_{20}NO_6$ 370.1285; found 370.1283.

Naphthelene monoimide monoanhydride (P10)

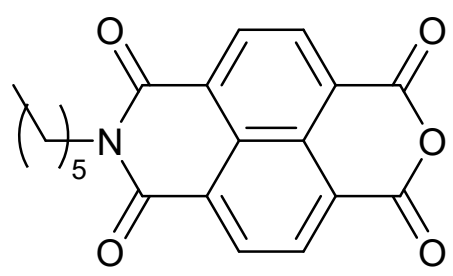

P9 (7.71 g, 20.9 mmol) was heated to reflux in thionyl chloride (150 mL). The reaction foams vigorously and heating had to be applied slowly. After 3 h, the reaction was allowed to cool to ambient temperature and was evaporated to dryness under reduced pressure. The residue was co-evaporated with *iso*-octane several times to afford **P10** as a light brown solid (7.50 g, 100%).

^{1}H NMR (400 MHz, $CDCl_3$) δ: 8.82 (4H, s, $\underline{H}_{Ar}$), 4.22-4.18 (2H, m, NC$\underline{H}_2$), 1.80-1.68 (2H, m, C$\underline{H}_2$), 1.50-1.24 (6H, m, C$\underline{H}_2$), 0.90 (3H, t, *J* 7.1, C$\underline{H}_3$).
^{13}C NMR (100 MHz, $CDCl_3$) δ: 162.2 and 158.9 ($\underline{C}$=O), 133.1, 131.2, 128.9, 127.9, 126.8, and 122.8 ($\underline{C}_{Ar}$), 41.2, 31.45, 28.0, 26.7, and 22.5 ($\underline{C}$H$_2$), 14.0 ($\underline{C}$H$_3$).
HRMS (APCI/QTOF): [M]$^-$ calcd for $C_{20}H_{17}NO_5$ 351.1112; found 351.1116.

Please do not adjust margins

C_6-NDI-A (43)

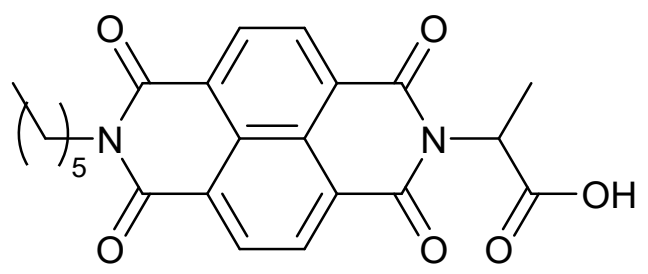

P10 (1.31 g, 3.73 mmol), alanine (1 eq, 332 mg), and imidazole (40 eq, 10.2 g) were mixed in a Schlenk tube and heated to 120 °C under N_2 and exclusion of light for 5 h. The temperature was then reduced to 90 °C, water was added (volume approx. equal to reaction volume), the mixture was stirred for 1 h, then allowed to cool to ambient temperature. It was then filtered through celite. Caution: some grades of celite appear to cause precipitation of the product inside the celite plug. Alternatively, a fine-meshed polymer filter can be used to remove an extremely fine suspension which otherwise passes through regular filter papers. The filtrate was poured into aqueous hydrochloric acid (1 M, 250 mL) and was stirred for 1 h. The precipitate was filtered off and washed with water. It was then stirred in aqueous hydrochloric acid (0.25 M, 250 mL) and heated at 80 °C for 3 hours. After cooling to ambient temperature, the solids were filtered off, placed in a flask with water, and stirred overnight. After another filtration and drying in a vacuum oven, **43** was obtained as brown flakes (1.16 g, 74%). One aliphatic carbon is overlapped by the solvent signal in the carbon NMR.

^{1}H NMR (400 MHz, DMSO-d$_6$) δ: 12.81 (1H, br, COOH), 8.70 (2H, d, *J* 7.6, H_{Ar}), 8.68 (2H, d, *J* 7.6, H_{Ar}), 5.60 (1H, q, *J* 7.0, CH_A), 4.05 (2H, t, *J* 7.5, N-CH_2), 1.70-1.61 (2H, m, CH_2), 1.57 (3H, d, *J* 7.0, CH_{3A}), 1.42–1.25 (6H, m, CH_2), 0.87 (3H, t, *J* 7.0, CH_2-CH_3).
^{13}C NMR (101 MHz, DMSO-d$_6$) δ: 171.1, 162.2, and 161.9 (C=O), 130.8, 130.3, 126.3, 125.9, 125.9, and 125.7 (C_{Ar}), 49.02 (C_A), 30.9, 27.3, 26.2, and 22.0 (CH_2), 14.4 (CH_{3A}), 13.9 (CH_2-CH_3).
HRMS (APCI/QTOF): $[M]^-$ calcd for $C_{23}H_{22}N_2O_6$ 422.1483; found 422.1481.

C_6-NDI-F (44)

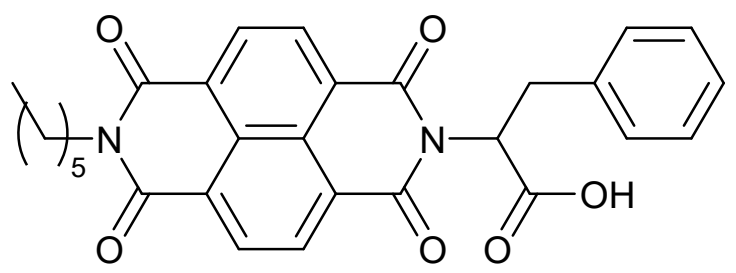

The same procedure was used as for **43,** except that phenylalanine was used as the amino acid.

^{1}H NMR (400 MHz, CDCl$_3$) δ: 8.72 (2H, d, *J* 7.6, H_{Ar}), 8.67 (2H, d, *J* 7.6, H_{Ar}), 7.18–7.02 (5H, m, H_{Ar}), 6.08 (1H, dd, *J* 10.1, 5.6, CH_F), 4.17 (2H, t, *J* 7.6, N-CH_2), 3.70 (1H, dd, *J* 14.3, 5.6, CH_{2F}), 3.52 (1H, dd, *J* 14.3, 10.1, CH_{2F}), 1.78–1.66 (2H, m, CH_2), 1.47–1.26 (6H, m, CH_2), 0.89 (3H, t, *J* 6.9, CH_3).
^{13}C NMR (101 MHz, DMSO-d$_6$) δ: 170.3, 162.3, and 162.1 (C=O), 137.7, 131.1, 130.3, 128.9, 128.1, 126.8, 126.4, 126.1, 125.8, and 124.9 (C_{Ar}), 54.4 (CH_F), 34.2 (CH_{2F}), 31.0, 27.3, 26.2, and 22.0 (CH_2), 13.9 (CH3).
HRMS (APCI/QTOF): $[M+H]^+$ calcd for $C_{29}H_{27}N_2O_6$ 499.1864; found 499.1869.

C_6-NDI-L (45)

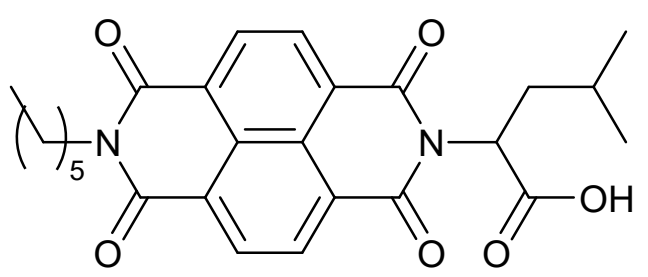

The same procedure was used as for **43,** except that leucine was used as the amino acid.

^{1}H NMR (400 MHz, CDCl$_3$) δ: 8.77 (4H, s, H_{Ar}), 5.83 (1H, dd, *J* 9.1, 5.0, CH_L), 4.20 (2H, t, *J* 7.6, N-CH_2), 2.29–2.20 (1H, m, CH_{2F}), 2.16–2.06 (1H, m, CH_{2F}), 1.80–1.70 (2H, m, CH_2), 1.64–1.51 (1H, m, CH_2), 1.49–1.26 (6H, m, CH_2), 1.01 (3H, d, *J* 6.5, CH_{3L}), 0.94 (3H, d, *J* 6.6, CH_{3L}), 0.90 (3H, t, *J* 7.1, CH_3).
^{13}C NMR (101 MHz, DMSO-d$_6$) δ: 170.9, 162.3, and 162.3 (C=O), 131.1, 130.4, 126.5, 126.1, and 125.5 (C_{Ar}), 51.8 (CH_L), 37.7 (CH_{2F}), 30.9, 27.3, 26.1, 24.9, 22.9, 21.9, 21.9, and 13.9.
HRMS (APCI/QTOF): $[M]^-$ calcd for $C_{26}H_{28}N_2O_6$ 464.1953; found 464.1953.

***tert*-Butyl (*tert*-butoxycarbonyl)glycyl-L-alaninate (P11)**

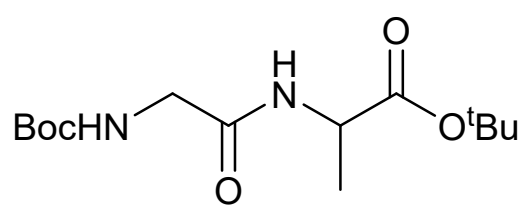

To a solution of N-Boc-glycine (6.16 g, 35.2 mmol) in chloroform (100 mL) was added iso-butyl chloroformate (1 eq, 4.57 mL) followed by N-methylmorpholine (2.2 eq, 8.51 mL). After stirring for 5 minutes, alanine tert-butyl ester hydrochloride (1.05 eq, 6.71 g) was added, and the mixture was stirred overnight. After this time, it was diluted with chloroform, washed in turn with aqueous hydrochloric acid (1 M), water, and brine, dried over anhydrous magnesium sulfate, filtered, and evaporated to dryness under reduced pressure. **P11** was obtained as a pale orange oil and used as is in the next step. A small amount was purified via column chromatography (1:9 ethyl acetate/dichloromethane) to obtain an analytical sample. Rotameric splitting (ca. 85:15) is observed in the proton NMR.

^{1}H NMR (400 MHz, DMSO-d$_6$) δ: 8.04 (1H, d, *J* 7.1, NH), 6.90 (0.85H, t, *J* 6.0, NH), 6.52 (0.15H, bs, NH), 4.15-4.08 (1H, m, CH_A), 3.58 (1H, dd, *J* 16.7, 6.2, CH_2), 3.50 (1H, dd, *J* 16.8, 6.1, CH_2), 1.39 (9H, s, CH_3), 1.37 (9H, s, CH_3), 1.23 (3H, d, *J* 7.28, CH_{3A}).
^{13}C NMR (101 MHz, DMSO-d$_6$) δ: 171.8, 169.1, and 155.7 (C=O), 80.4 and 78.0 (C-CH_3), 48.2 (CH_A), 42.8 (CH_2), 28.2 and 27.6 (CH_3), 17.2 (CH_{3A}).
HRMS (ESI/QTOF) m/z: $[M+Na]^+$ calcd for $C_{14}H_{26}N_2NaO_5$ 325.1734; found 325.1740.

 This journal is © The Royal Society of Chemistry 20xx

Please do not adjust margins

Glycyl-L-alanine trifluoroacetate (P12)

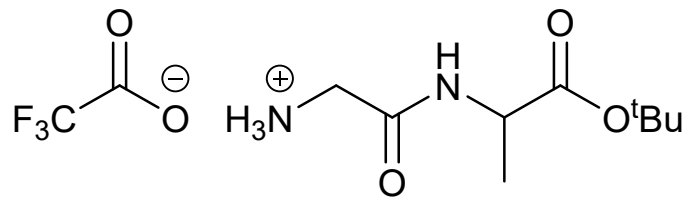

To a solution of **P11** (7.87 g, 26.0 mmol) in chloroform (40 mL) was added trifluoroacetic acid (20 mL, *ca*. 10 eq) and the mixture was stirred overnight. It was then concentrated under reduced pressure and excess diethyl ether was added. The solids were filtered off after 1 h, returned into diethyl ether and stirred for 1 h. Another filtration and drying under vacuum afforded **P12** as a white solid (5.10 g, 75%).

^{1}H NMR (400 MHz, DMSO-d$_6$) δ: 12.75 (bs, COOH), 8.68 (1H, d, *J* 7.23, NH), 8.40–7.74 (3H, bs, NH_3^+), 4.33–4.23 (1H, m, CH_A), 3.58 (2H, s, CH_{2G}), 1.30 (3H, d, *J* 7.28, CH_{3A}).
^{13}C NMR (101 MHz, DMSO-d$_6$) δ: 173.6 and 165.7 (C=O), 158.4 (q, *J* 31.2, F_3C-C=O), 117.2 (q, *J* 299.5, CF_3), 47.8 (CH_A), 40.0 (CH_{2G}), 17.3 (CH_{3A}).
HRMS (ESI/QTOF) m/z: [M-TFA+H]$^+$ calcd for $C_5H_{11}N_2O_3$ 147.0764; found 147.0767.

NDI-GA (46)

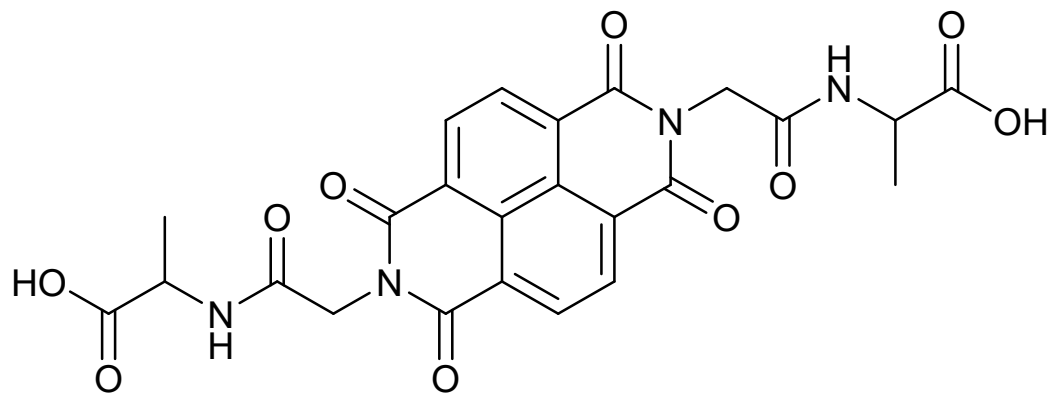

NTCDA (895 mg, 3.34 mmol), **P12** (2.05 eq, 1.78 g), and imidazole (40 eq, 9.10 g) were mixed and stirred at 120 °C in a Schlenk tube under an nitrogen atmosphere in the dark for 5 h. The temperature was reduced to 90 °C, water (ca. 10 mL) was added and stirring at 90 °C continued for 1 h. After this time, the tube was filled with water (ca. 50 mL) and allowed to cool to ambient temperature while stirring overnight. The insolubles were filtered off, the filtrate was poured into aqueous hydrochloric acid (1M, ca. 400 mL), and stirred for 1 h. The precipitate was filtered off, transferred into aqueous hydrochloric acid (0.5 M) and heated at approx. 60 °C overnight. The solids were filtered off, washed with copious amounts of water, and dried in an oven at 60 °C to give **46** as a dark grey solid (862 mg, 49%).
^{1}H NMR (400 MHz, DMSO-d$_6$) δ: 12.6 (2H, bs, COOH), 8.71 (4H, s, H_{Ar}), 8.61 (2H, d, *J* 7.38, NH), 4.76 (2H, d, *J* 16.2, CH_2), 4.70 (2H, d, *J* 16.2, CH_2), 4.29–4.21 (2H, m, CH_A), 1.29 (6H, d, *J* 7.3, CH_3).
^{13}C NMR (101 MHz, DMSO-d$_6$, 80 °C) δ: 173.0, 165.5, and 161.9 (C=O), 130.2, 126.00, and 125.9 (C_{Ar}), 47.3 and 42.2 (CH_{2G} and CH_A), 17.0 (CH_{3A}).
HRMS (ESI/QTOF) m/z: [M+Na]$^+$ calcd for $C_{24}H_{20}N_4NaO_{10}$ 547.1072; found 547.1075.

***tert*-Butyl (*tert*-butoxycarbonyl)glycyl-L-leucinate (P13)**

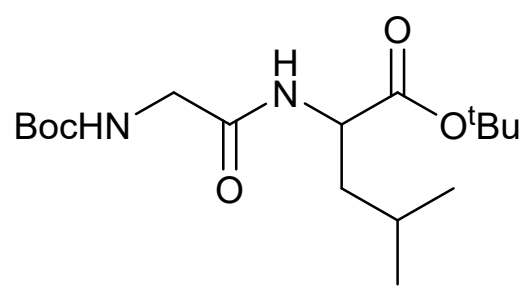

^{1}H NMR (400 MHz, DMSO-d$_6$) δ: 7.95 (0.9H, d, *J* 7.8, NH), 6.91 (0.85H, t, *J* 6.0, NH), 6.58–6.46 (0.15H, bs, NH), 4.22–4.09 (1H, m, NH_L-CH_L), 3.64–3.45 (2H, m, CH_{2G}), 1.68–1.21 (21H, m, CH_3 and CH_{2L}-CH_L-CH_{3L}), 0.88 (3H, d, *J* 6.60, CH_{3L}), 0.83 (3H, d, *J* 6.52, CH_{3L}).
^{13}C NMR (101 MHz, DMSO-d$_6$) δ: 171.7, 169.3, and 155.7 (C=O), 80.5 and 78.0 (C-CH_3), 50.9 (NH_L-CH_L), 42.9 (CH_{2G}), 40.1 (overlapped by DMSO, CH_{2L}), 28.1 and 27.6 (CH_3), 24.2 (CH_L-CH_3), 22.7 and 21.5 (CH_{3L}).
HRMS (APCI/QTOF) m/z: [M+H]$^+$ calcd for $C_{17}H_{33}N_2O_5$ 345.2384; found 345.2387.

Glycyl-L-leucine trifluoroacetate (P14)

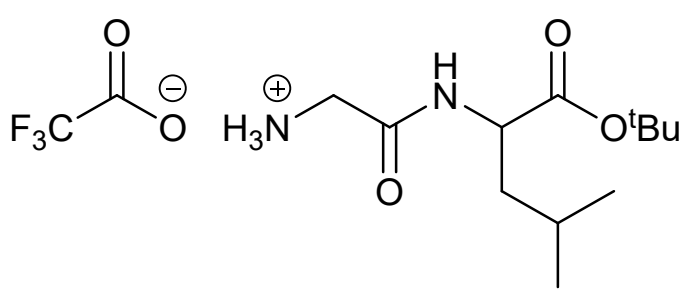

^{1}H NMR (400 MHz, DMSO-d$_6$) δ: 10.92–6.68 (*ca.* 3H, bs, NH_3^+), 8.66 (1H, d, *J* 7.97, NH), 4.30–4.25 (1H, m, NH_L-CH_L), 3.63 (1H, d, *J* 16.2, CH_{2G}), 3.57 (1H, d, *J* 16.2, CH_{2G}), 1.70–1.57 (1H, m, CH_L-CH_{3L}), 1.54–1.51 (2H, m, CH_{2L}), 0.89 (3H, d, *J* 6.54, CH_{3L}), 0.85 (3H, d, *J* 6.47, CH_{3L}).
^{13}C NMR (101 MHz, DMSO-d$_6$) δ: 173.6 and 166.0 (C=O), 158.3 (q, *J* 31.23, F_3C-C=O), 117.2 (q, *J* 299.6, CF_3), 50.5 (NH_L-CH_L), 40.1 (overlapped by DMSO, CH_{2L}), 40.0 (CH_{2G}), 24.2 (CH_L-CH_{3L}), 22.8 and 21.3 (CH_{3L}).
HRMS (ESI/QTOF) m/z: [M-TFA+H]$^+$ calcd for $C_8H_{17}N_2O_3$ 189.1234; found 189.1238.

NDI-GL (47)

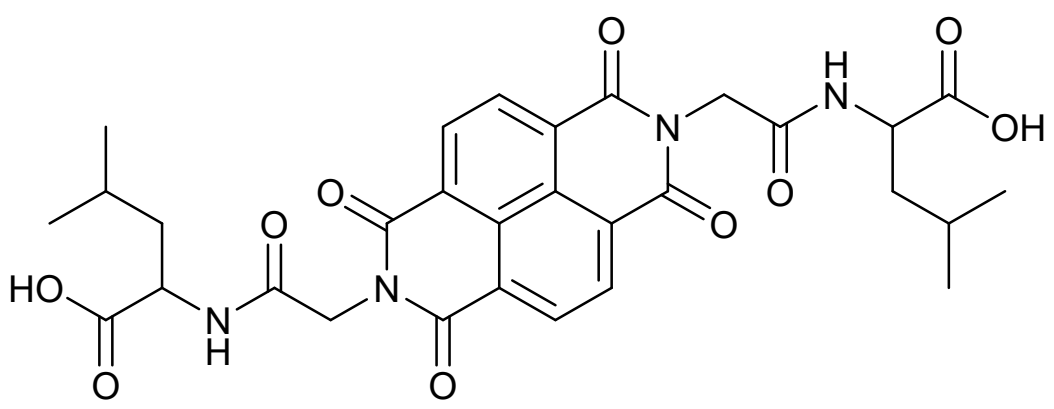

^{1}H NMR (400 MHz, DMSO-d$_6$) δ: 12.62 (2H, bs, COOH), 8.71 (4H, s, H_{Ar}), 8.55 (2H, d, *J* 8.09, NH), 4.75 (2H, d, *J* 16.5, CH_{2G}), 4.71 (2H, d, *J* 16.5, CH_{2G}), 4.31–4.25 (2H, m, NH_L-CH_L), 1.72–1.61 (2H, m, CH_L-CH_{3L}), 1.56–1.48 (4H, m, CH_{2L}), 0.90 (6H, d, *J* 6.56, CH_{3L}), 0.86 (6H, d, *J* 6.5, CH_{3L}).
^{13}C NMR (101 MHz, DMSO-d$_6$) δ: 173.9, 166.3, and 162.4 (C=O), 130.7 and 126.2 (C_{Ar}), 50.4 (NH_L-CH_L), 42.5 (CH_{2G}), 40.2 (CH_{2L}), 24.3 (CH_L-CH_{3L}), 22.9 (CH_{3L}) and 21.4 (CH_{3L}).
HRMS (ESI/QTOF) m/z: [M+Na]$^+$ calcd for $C_{30}H_{32}N_4NaO_{10}$ 631.2011; found 631.2019.

tert-Butyl (_tert_-butoxycarbonyl)leucyl-L-alininate (P15)

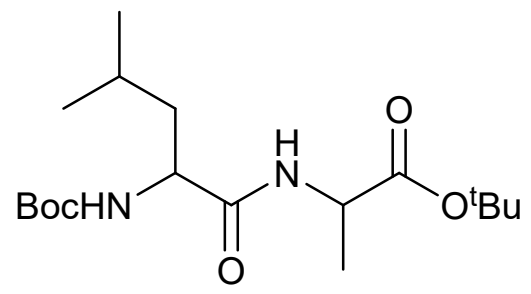

^{1}H NMR (400 MHz, DMSO) δ: 8.07 (1H, d, *J* 7.0, NH), 6.80 (1H, d, *J* 8.6, NH), 4.09 (1H ,p, *J* 7.2, NH$_A$-CH$_A$), 3.98 (1H, td, *J* 8.9, 6.0, NH$_L$-CH$_L$), 1.62 (1H, dt, 13.5, 6.8, CH$_L$-CH$_{3L}$), 1.43 – 1.39 (2H, m, CH$_{2L}$), 1.37 (18H, d, *J* 4.5, CH$_3$), 1.24 (3H, d, *J* 7.3, CH$_{3A}$), 0.86 (6H, dd, *J* 6.6, 5.1, CH$_{3L}$).
^{13}C NMR (101 MHz, DMSO) δ: 172.4, 171.7, and 155.3 (C=O), 80.2 and 77.8 (C-CH$_3$), 52.3 (NH$_L$-CH$_L$), 48.2 (NH$_A$-CH$_A$), 40.9 (CH$_{2L}$), 28.2 and 27.6 (CH$_3$), 24.2 (CH$_L$), 23.1 and 21.4 (CH$_{3L}$), 16.9 (CH$_{3A}$).
HRMS (ESI/QTOF) m/z: [M+H]$^+$ calcd for $C_{18}H_{35}N_2O_5$ 359.2540; found: 359.2550.

NDI-LA (48)

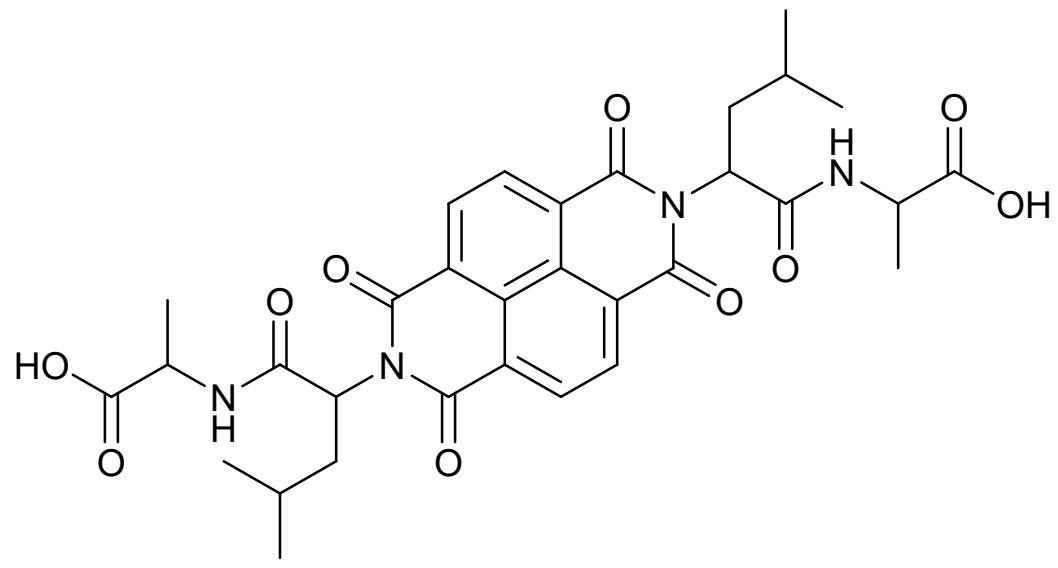

^{1}H NMR (400 MHz, DMSO) δ: 12.49 (s, 2H, COOH), 8.71 (4H, s, H$_{Ar}$), 8.34–8.15 (2H, m, NH), 5.55–5.36 (2H, m, NH$_L$-CH$_L$), 4.30 (2H, p, *J* 7.29, CH$_A$-CH$_{3A}$), 2.27–2.12 (2H, m, CH$_{2L}$), 2.10–1.81 (2H, m, CH$_{2L}$), 1.56–1.43 (2H, m, CH$_L$-CH$_{3L}$), 1.24 (1H, dd, *J* 7.26, 2.37, CH$_{3A}$), 1.08 (5H, d, *J* 7.34, CH$_{3A}$), 0.91 (6H, d, *J* 6.53, CH$_{3L}$), 0.84 (6H, dd, *J* 6.75, 1.77, CH$_{3L}$).
^{13}C NMR (101 MHz, DMSO) δ: 174.3, 168.3, and 162.8 (C=O), 130.5, 126.7, and 126.5 (C$_{Ar}$), 52.5 (NH$_L$-CH$_L$), 47.6 (NH$_A$-CH$_A$), 37.2 (CH$_{2L}$), 24.9 (CH$_L$), 23.1 and 22.1 (CH$_{3L}$), 17.0 (CH$_{3A}$).
HRMS (ESI/QTOF) m/z: [M+H]$^+$ calcd for $C_{32}H_{37}N_4O_{10}$ 637.2504; found: 637.2503.

tert-Butyl (N-_tert_-butoxycarbonyl-_O_-_tert_-butyl)-L-tyrosinyl-L-isoleucinate (P16)

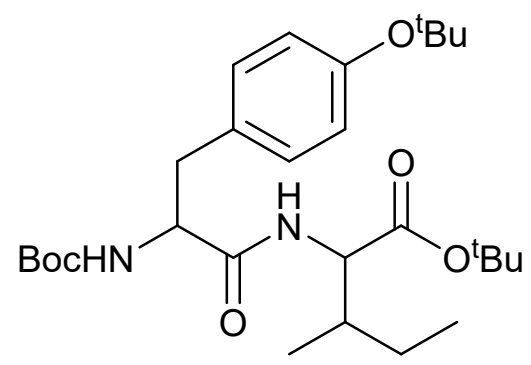

^{1}H NMR (400 MHz, DMSO-d$_6$) δ: 8.04 (0.15H, d, *J* 8.1, NH), 7.93 (0.85H, d, *J* 8.2, NH), 7.22 (0.3H, d, *J* 7.3, H$_{Ar}$), 7.17 (1.7H, d, *J* 8.3, H$_{Ar}$), 6.93 (0.85H, d, *J* 8.79, NH), 6.85 (2H, d, *J* 8.3, H$_{Ar}$), 6.46 (0.15H, d, *J* 7.7, NH), 4.28–4.19 (0.85H, m, CH-NH), 4.19–4.16 (0.15H, m, CH-NH), 4.16–4.07 (1H, m, CH-NH), 2.91 (0.85H, dd, *J* 13.9, 3.7, Ph-CH$_2$), 2.87–2.82 (0.15H, br, Ph-CH$_2$), 2.67 (0.85H, dd, *J* 13.7, 10.7, Ph-CH$_2$), 2.62–2.57 (0.15H, br, Ph-CH$_2$), 1.84–1.71 (1H, m, CH-CH$_3$), 1.46–1.38 (1H, CH-CH$_2$, overlapped by C(CH$_3$)$_3$), 1.40 (9H, s, C(CH$_3$)$_3$), 1.31–1.17 (1H, CH-CH$_2$, overlapped by C(CH$_3$)$_3$), 1.29 (9H, s, C(CH$_3$)$_3$), 1.26 (9H, s, C(CH$_3$)$_3$), 0.87 (3H, t, *J* 7.2, CH$_2$-CH$_3$), 0.86 (3H, d, *J* 6.7, CH-CH$_3$).
^{13}C NMR (100 MHz, DMSO-d$_6$) δ: 171.8, 170.3, and 155.2 (C=O), 153.3, 132.7, 129.6, and 123.2 (C$_{Ar}$), 80.6, 77.9, and 77.5 (C(CH$_3$)$_3$), 56.7 and 55.4 (CH-NH), 36.8 (Ph-CH$_2$), 36.6 (CH-CH$_3$), 28.5, 28.1, and 27.6 (C(CH$_3$)$_3$), 24.8 (CH-CH$_2$), 15.3 (CH-CH$_3$), 11.2 (CH$_2$-CH$_3$).
HRMS (ESI/QTOF) m/z: [M+H]$^+$ calcd for $C_{28}H_{47}N_2O_6$ 507.3429; found: 507.3436.

_L_-Tyrosinyl-_L_-isoleucine hydrochloride (P17)

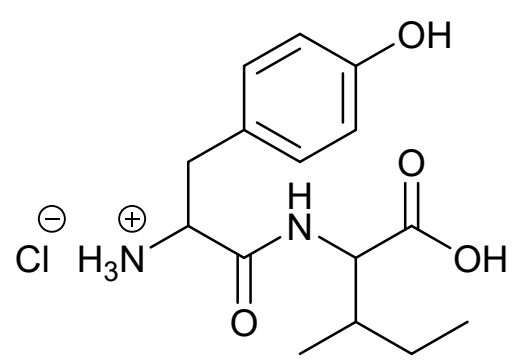

P16 (8.17 g, 16.1 mmol) was dissolved in hydrogen chloride in dioxane (4 M, 40 mL) and stirred overnight. The resulting clear yellow solution was evaporated under reduced pressure. NMR of the residue showed trace remaining *tert*-butyl ester. The crude material was dissolved and stirred in hydrogen chloride in dioxane (4 M, 25 mL) for 3 d. The solvent was evaporated under reduced pressure and the remaining solids stirred in diethyl ether. The solids were then filtered under nitrogen, immediately returned into diethyl ether and stirred for another 2 h. The solid was collected by filtration under nitrogen, then dried in a vacuum oven to afford **P17** (4.32 g, 81%) as a cream solid. Solvent residues from attempts at removing trace dioxane, as well as the dioxane itself, could not be removed from the material even by prolonged exposure to vacuum. The material had an NMR purity of 97.8%, with the balance consisting of dioxane (0.3%), acetonitrile (1.1%), and diethyl ether (0.7%).

^{1}H NMR (400 MHz, DMSO-d$_6$) δ: 13.9–11.8 (1H, br, COOH), 9.40 (1H, br s, OH), 8.71 (1H, d, *J* 8.2, NH), 8.62–7.64 (3H, br s, NH$_3^+$), 7.08 (2H, d, *J* 8.5, H$_{Ar}$), 6.70 (2H, d, *J* 8.5, H$_{Ar}$), 4.22 (1H, dd, *J* 8.2, 5.8, CH-NH), 4.08 (1H, dd, *J* 7.3, 5.5, CH-NH), 3.04 (1H, dd, *J* 14.2, 5.2, Ph-CH$_2$), 2.84 (1H, dd, *J* 14.2, 7.5, Ph-CH$_2$), 1.89–1.75 (1H, m, CH-CH$_3$), 1.52–1.38 (1H, m, CH$_2$-CH$_3$), 1.30–1.14 (1H, m, CH$_2$-CH$_3$), 0.92–0.83 (6H, m, CH-CH$_3$ and CH$_2$-CH$_3$).
^{13}C NMR (100 MHz, DMSO-d$_6$) δ: 172.3 and 168.3 (C=O), 156.6, 130.6, 124.7, and 115.3 (C$_{Ar}$), 56.6 and 53.3 (CH-NH), 36.5 (CH-CH$_3$), 36.0 (Ph-CH$_2$), 24.8 (CH$_2$-CH$_3$), 15.6 (CH-CH$_3$), 11.4 (CH$_2$-CH$_3$).
HRMS (APCI) m/z: [M-HCl+H]$^+$ calcd for $C_{15}H_{23}N_2O_4$ 295.1652; found: 295.1664.

This journal is © The Royal Society of Chemistry 20xx
Please do not adjust margins

NDI-YI (49)

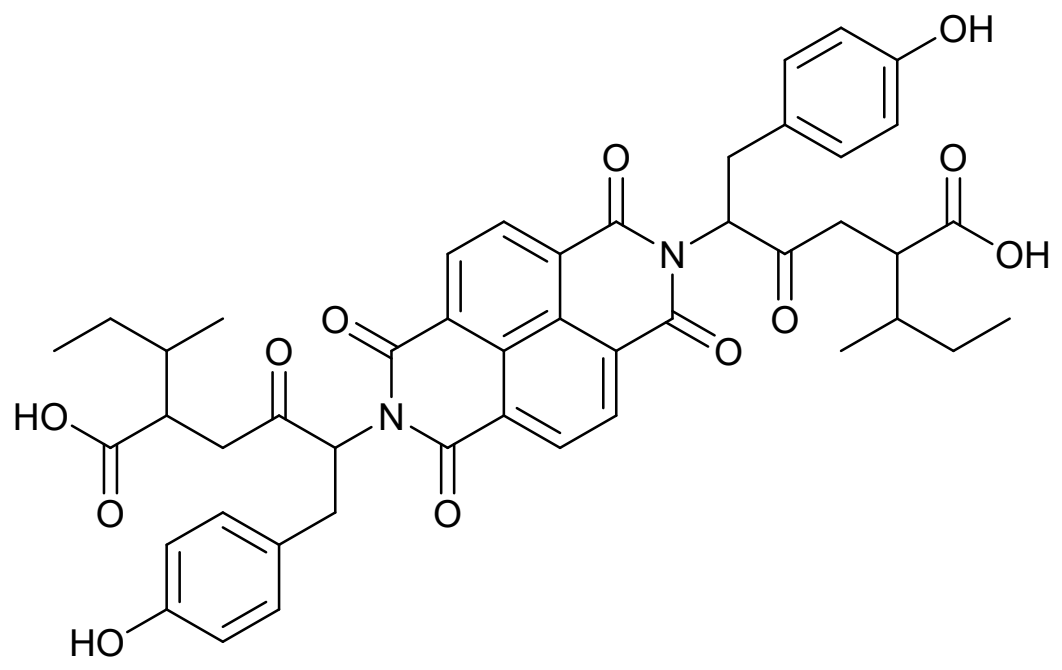

NTCDA (815 mg, 3.04 mmol), **P17** (2.02 g, 6.11 mmol), and imidazole (8.28 g, 73.8 mmol) were stirred at 120 °C in an oil bath under an argon atmosphere in the dark for 5 h. The temperature was then reduced to 90 °C, water was added and stirring at 90 °C continued for a further 1 h. The tube was allowed to cool to ambient temperature while stirring overnight. In contrast to most other NDI syntheses, the product is contained in the solid in the filter rather than in the filtrate. This solid was transferred into hydrochloric acid (0.5 M) and stirred overnight. The solid was filtered and washed with water on the filter. The crude solid was transferred into acetonitrile and stirred for 1 h, filtered, washed with acetonitrile, then dried on a rotary evaporator to give **49** as an orange solid. Another small crop of **49** is obtained as the acetonitrile washings develop a precipitate on standing. Combined yield: 1.21 g (49%). An approx. 85:15 distribution of rotamers is seen in the NMR.

^{1}H NMR (400 MHz, DMSO-d$_6$) δ: 12.54 (2H, s, COOH), 9.00 (2H, s, OH), 8.57 (4H, s, H$_{Ar}$), 8.20 (1.7H, d, *J* 8.4, NH), 8.13 (0.3H, d, *J* 8.9, NH), 6.92–6.85 (4H, m, H$_{ArY}$), 6.47–6.39 (4H, m, H$_{ArY}$), 5.69 (0.3H, dd, *J* 9.8, 5.7, CH-NH), 5.61 (1.7H, dd, *J* 9.1, 5.8, CH-NH), 4.31–4.21 (2H, m, CH-NH), 3.59–3.48 (2H, m, Ph-CH$_2$), 3.21 (0.3H, dd, *J* 14.2, 10.0, Ph-CH$_2$), 3.10 (1.7H, dd, *J* 13.9, 9.4, Ph-CH$_2$), 1.77–1.70 (0.3H, m, Ile-H), 1.70-1.59 (1.7H, m, Ile-H), 1.45-1.33 (0.3H, m, Ile-H), 1.33-1.17 (1.7H, m, Ile-H), 1.16-1.05 (0.3H, m, Ile-H), 1.05-0.90 (1.7H, m, Ile-H), 0.87-0.64 (12H, m, CH$_3$).
^{13}C NMR (100 MHz, DMSO-d$_6$) δ: 173.4, 168.2, and 162.5 (C=O), 155.5, 130.4, 130.1, 130.0, 129.9, 128.2, 128.0, 126.7, 126.4, 126.2, 126.1, and 114.9 (C$_{Ar}$), 56.5 and 55.5 (CH-NH), 36.0, 35.7, 33.5, 24.8, 24.7, 15.5, 10.8, and 10.7 (aliphatic C).
HRMS (ESI/QTOF) m/z: [M-H]$^-$ calcd for $C_{44}H_{43}N_4O_{12}$ 819.2883; found: 819.2888.

PBI-A-1Py (50)

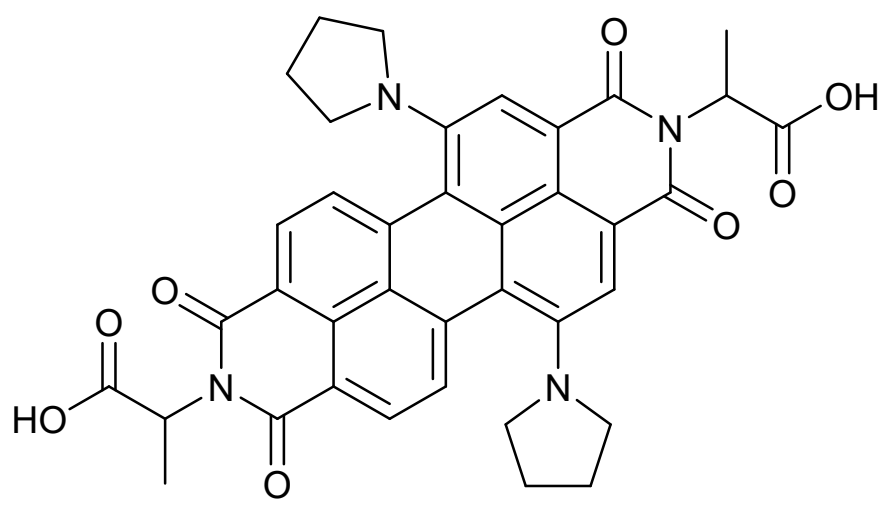

A mixture of **20** (2.0 g, 3.74 mmol) and copper(II) chloride (420 mg, 3.78 mmol) in pyrrolidine (50.0 mL, 599 mmol) was stirred for 2 d in air in which the solution turned from bright purple to bright blue. The solution was diluted with water (100 mL), filtered, and the filtrate allowed to drip into vigorously stirred aqueous hydrochloric acid (1 M, 800 mL). The resulting blue precipitate was collected at the filter, washed with aqueous hydrochloric acid (1 M, 2 × 200 mL), water (2 × 200 mL) and allowed to dry on the filter overnight. The solid was then vigorously stirred in aqueous hydrochloric acid (1 M, 300 mL) overnight. The solid was collected at the filter, washed with water (2 × 200 mL), and again allowed to dry on the filter overnight. The crude solid was sonicated in an acetone:acetonitrile mixture (1:1, 20 mL) for 1 h, collected at the filter, washed with methanol (10 mL), and dried in an oven at 130 °C for 3 days to give **50** as a bright blue solid (1.30 g, 52%).

^{1}H NMR (400 MHz, CDCl$_3$) δ: 8.65 (2H, d, *J* 8.1, H$_{Ar}$), 8.31 (2H, d, *J* 1.7, H$_{Ar}$), 7.75 (2H, d, *J* 8.1, H$_{Ar}$), 5.97–5.90 (1H, m, CH$_A$), 5.7–5.80 (1H, m, CH$_A$), 3.67 (4H, bs, N-CH$_2$), 2.71 (4H, bd, *J* 6.2, N-CH$_2$), 2.17–1.81 (8H, m, N-CH$_2$-CH$_2$), 1.75 (6H, t, *J* 6.2, CH$_{3A}$).
^{13}C NMR (101 MHz, CDCl$_3$) δ: 176.52 (COOH), 176.47 (COOH), 176.2 (COOH), 176.1 (COOH), 163.7 (C=O), 163.2 (C=O), 150.1 (C$_{Ar}$-N), 135.9 (C$_{Ar}$), 130.9 (C$_{Ar}$), 130.6 (CH$_{Ar}$), 128.4 (C$_{Ar}$), 123.2 (CH$_{Ar}$), 122.5 (C$_{Ar}$), 117.8 (CH$_{Ar}$), 117.0 (C$_{Ar}$), 116.8 (C$_{Ar}$), 116.6 (C$_{Ar}$), 52.4 (N-CH$_2$), 52.3 (N-CH$_2$), 49.3 (CH$_A$), 48.8 (CH$_A$), 25.6 (N-CH$_2$-CH$_2$), 14.6 (CH$_{3A}$), 14.5 (CH$_{3A}$).
HRMS (ESI/QTOF) *m/z*: [M−H]$^-$ calcd for $C_{38}H_{31}N_4O_8$ 671.2147; found 671.2146.

PBI-I-1Py (51)

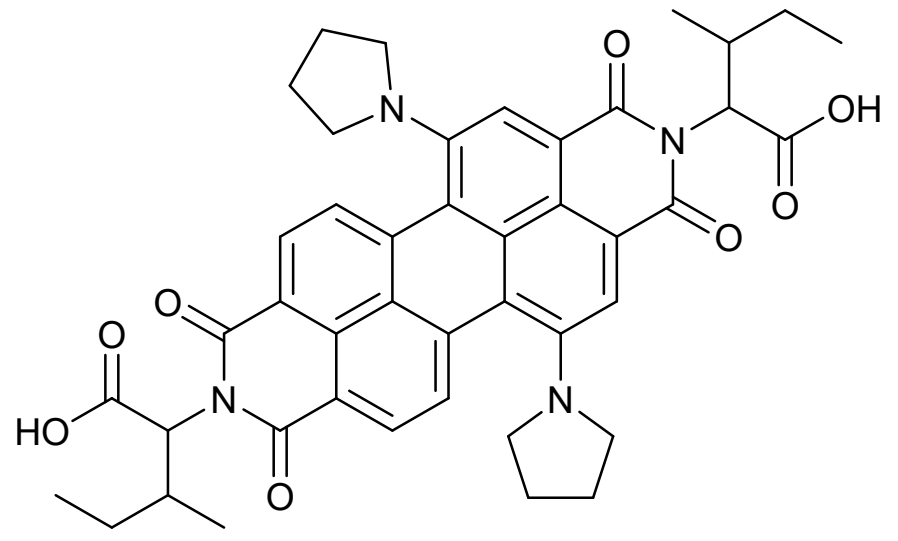

A mixture of **27** (5.0 g, 8.08 mmol) and copper(II) chloride (1.08 g, 9.73 mmol) in pyrrolidine (75.0 mL, 898 mmol) was stirred overnight in air in which the solution turned from bright purple to bright blue. The solution was diluted with water (100 mL), filtered, and the filtrate allowed to drip into vigorously stirred aqueous hydrochloric acid (1 M, 600 mL). The resulting blue precipitate was collected at the filter, washed with aqueous hydrochloric acid (1 M, 2 × 200 mL), water (2 × 200 mL), and allowed to dry on the filter overnight. The solid was then vigorously stirred in aqueous hydrochloric acid (1 M, 300 mL) overnight. The solid was collected at the filter, washed with water (2 × 200 mL), and again allowed to dry on the filter overnight. The crude solid was collected and stirred in an acetonitrile:methanol mixture (10:1, 50 mL) overnight. The solid was collected at the filter, washed with acetonitrile (2 × 20 mL), and dried in an oven at 120 °C for 3 days to give **51** as a bright blue solid (1.98 g, 32%).

^{1}H NMR (400 MHz, $CDCl_3$) *δ*: 8.67 (2H, d, *J* 8.0, $\underline{H}_{Ar}$), 8.34 (2H, d, *J* 1.7, $\underline{H}_{Ar}$), 7.78 (2H, d, *J* 8.1, $\underline{H}_{Ar}$), 5.60 (1H, ddd, *J* 13.8, 9.1, 1.3, N-$C\underline{H}_{I}$), 5.50 (1H, ddd, *J* 15.0, 9.1, 1.8, N-$C\underline{H}_{I}$), 3.70 (4H, bs, N-$C\underline{H}_2$), 2.95–2.57 (6H, m, N-$C\underline{H}_2$ and $C\underline{H}_{I}$-CH_{3I}), 2.20–1.83 (9H, m, N-CH_2-$C\underline{H}_2$ and $C\underline{H}_{2I}$), 1.48–1.25 (5H, m, $C\underline{H}_{2I}$-$C\underline{H}_{3I}$), 1.13–0.98 (4H, m, $C\underline{H}_{2I}$-$C\underline{H}_{3I}$), 0.90–0.77 (6H, m, CH_{2I}-$C\underline{H}_{3I}$).
^{13}C NMR (101 MHz, DMSO-d_6 + 10 µL TFA) *δ*: 177.3 ($\underline{C}$OOH), 177.2 ($\underline{C}$OOH), 177.0 ($\underline{C}$OOH), 176.9 ($\underline{C}$OOH), 164.7 ($\underline{C}$=O), 164.6 ($\underline{C}$=O), 150.0 ($\underline{C}_{Ar}$-N), 136.5 ($\underline{C}_{Ar}$), 132.0 ($\underline{C}H_{Ar}$), 130.7 ($\underline{C}_{Ar}$), 128.8 ($\underline{C}_{Ar}$), 128.0 ($\underline{C}_{Ar}$), 123.6 ($\underline{C}H_{Ar}$), 122.3 ($\underline{C}_{Ar}$), 118.7 ($\underline{C}H_{Ar}$), 117.0 ($\underline{C}_{Ar}$), 115.91 ($\underline{C}_{Ar}$), 115.87 ($\underline{C}_{Ar}$), 58.6 (N-$\underline{C}H_{I}$), 58.5 (N-$\underline{C}H_{I}$), 57.3 (N-$\underline{C}H_{I}$), 57.1 (N-$\underline{C}H_{I}$), 53.8 (N-$\underline{C}H_2$), 33.7 ($\underline{C}H_{I}$-CH_{3I}), 33.6 ($\underline{C}H_{I}$-CH_{3I}), 33.5 ($\underline{C}H_{I}$-CH_{3I}), 28.2 ($\underline{C}H_{2I}$-CH_{3I}), 28.1 ($\underline{C}H_{2I}$-CH_{3I}), 25.8 (N-CH_2-$\underline{C}H_2$), 25.1 ($\underline{C}H_{2I}$-CH_{3I}), 25.0 ($\underline{C}H_{2I}$-CH_{3I}), 17.9 (CH_{I}-$\underline{C}H_{3I}$), 17.8 (CH_{I}-$\underline{C}H_{3I}$), 14.9 (CH_{I}-$\underline{C}H_{3I}$), 14.8 (CH_{I}-$\underline{C}H_{3I}$), 11.12 (CH_{2I}-$\underline{C}H_{3I}$), 11.09 (CH_{2I}-$\underline{C}H_{3I}$), 11.04 (CH_{2I}-$\underline{C}H_{3I}$), 11.02 (CH_{2I}-$\underline{C}H_{3I}$).
HRMS (ESI/QTOF) *m/z*: $[M-H]^-$ calcd for $C_{44}H_{43}N_4O_8$ 755.3086; found 755.3086.

PBI-L-1Py (52)

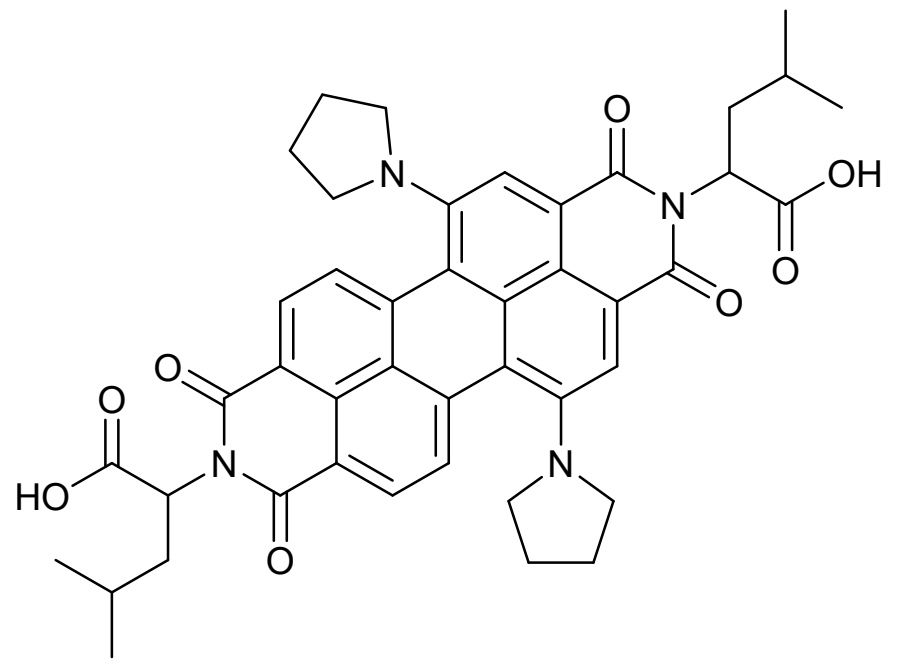

A mixture of **28** (5.0 g, 8.08 mmol) and copper(II) chloride (1.10 g, 9.91 mmol) in pyrrolidine (50.0 mL, 825 mmol) was stirred overnight in air in which the solution turned from bright purple to bright blue. The solution was diluted with water (50 mL), filtered, and the filtrate allowed to drip into vigorously stirred aqueous hydrochloric acid (1 M, 600 mL). The resulting blue precipitate was collected at the filter, and then vigorously stirred in aqueous hydrochloric acid (1 M, 500 mL) overnight. The solid was collected at the filter, washed with water (3 × 500 mL), and allowed to dry on the filter overnight. The crude solid was sonicated in methanol (20 mL) for 1 h, collected at the filter, washed with methanol (10 mL), and dried in an oven at 120 °C for 3 days to give **52** as a bright blue solid (3.49 g, 57%).

^{1}H NMR (400 MHz, $CDCl_3$) *δ*: 8.64 (2H, d, *J* 8.6, $\underline{H}_{Ar}$), 8.32 (2H, d, *J* 1.8, $\underline{H}_{Ar}$), 7.76 (2H, d, *J* 8.1, $\underline{H}_{Ar}$), 5.94 (1H, ddd, *J* 8.5, 5.1, 2.9, N-$C\underline{H}_{L}$), 5.84 (1H, ddd, *J* 8.5, 5.1, 2.9, N-$\underline{C}H_{L}$), 3.68 (4H, bs, N-$C\underline{H}_2$), 2.73 (4H, bs, N-$C\underline{H}_2$), 2.31–2.10 (4H, m, $C\underline{H}_{2L}$), 2.09–1.76 (8H, m, N-CH_2-$C\underline{H}_2$), 1.70–1.53 (2H, m, $C\underline{H}_{L}$-CH_{3L}), 1.03 (6H, dd, *J* 8.8, 6.4, $C\underline{H}_{3L}$), 0.94 (6H, dd, *J* 6.7, 4.7, $C\underline{H}_{3L}$).
^{13}C NMR (101 MHz, $CDCl_3$) *δ*: 176.4 ($\underline{C}$OOH), 176.0 ($\underline{C}$OOH), 164.2 ($\underline{C}$=O), 163.9 ($\underline{C}$=O), 150.3 ($\underline{C}_{Ar}$-N). 136.2 ($\underline{C}_{Ar}$), 131.3 ($\underline{C}_{Ar}$), 131.0 ($\underline{C}H_{Ar}$), 128.8 ($\underline{C}_{Ar}$). 128.5 ($\underline{C}_{Ar}$), 123.4 ($\underline{C}H_{Ar}$), 122.7 ($\underline{C}_{Ar}$), 118.3 ($\underline{C}H_{Ar}$), 117.3 ($\underline{C}_{Ar}$), 117.2 ($\underline{C}_{Ar}$), 117.1 ($\underline{C}_{Ar}$), 52.4 (N-$\underline{C}H_2$-CH_2). 52.2 (N-$\underline{C}H_{L}$), 51.9 (N-$\underline{C}H_{L}$), 38.3 ($\underline{C}H_{2L}$), 38.2 ($\underline{C}H_{2L}$), 25.7 (N-CH_2-$\underline{C}H_2$), 25.6 ($\underline{C}H_{L}$-CH_{3L}), 23.23 ($\underline{C}H_{3L}$), 23.19 ($\underline{C}H_{3L}$), 22.4 ($\underline{C}H_{3L}$), 22.3 ($\underline{C}H_{3L}$).
HRMS (ESI/QTOF) *m/z*: $[M-H]^-$ calcd for $C_{44}H_{43}N_4O_8$ 755.3086; found 755.3085.

This journal is © The Royal Society of Chemistry 20xx

PBI-V-1Py (53)

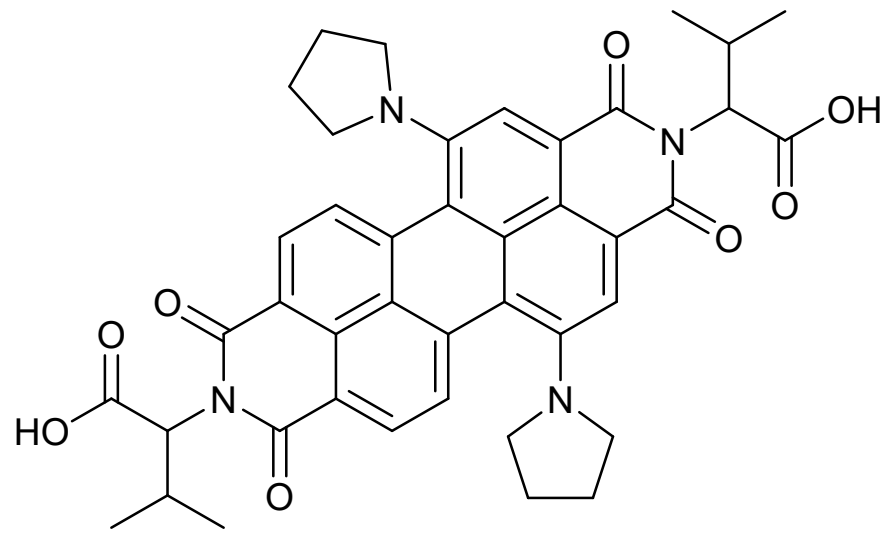

A mixture of **31** (5.40 g, 9.14 mmol) and copper(II) chloride (1.23 g, 11.1 mmol) in pyrrolidine (75.0 mL, 898 mmol) was stirred overnight in air in which the solution turned from bright purple to bright blue. The solution was filtered, and the filtrate allowed to drip into vigorously stirred aqueous hydrochloric acid (1 M, 800 mL). The resulting blue precipitate was collected at the filter, washed with aqueous hydrochloric acid (1 M, 100 mL), water (500 mL) and was allowed to dry on the filter overnight. The solid was collected and vigorously stirred in aqueous hydrochloric acid (1 M, 500 mL) that was heated in an oil bath held at 90 °C for 2 h. The solid was collected at the filter, washed with water (1 L), and allowed to dry on the filter overnight. The crude solid was sonicated in methanol (200 mL) for 1 h, collected at the filter, washed with methanol (50 mL), and dried in an oven at 120 °C for 3 days to give **53** as a bright blue solid (1.35 g, 20%).

^{1}H NMR (400 MHz, $CDCl_3$) δ: 8.66 (2H, d, *J* 8.0, $\underline{H}_{Ar}$), 8.35 (2H, s, $\underline{H}_{Ar}$), 7.78 (2H, d, *J* 8.0, $\underline{H}_{Ar}$), 5.53 (1H, d, *J* 9.0, N-C$\underline{H}_V$), 5.43 (1H, dd, *J* 9.2, 1.4, N-C$\underline{H}_V$), 3.70 (4H, bs, N-C$\underline{H}_2$), 3.10–2.53 (6H, m, C$\underline{H}_V$-CH_{3V} and N-C$\underline{H}_2$), 2.21–1.80 (8H, m, N-CH_2-C$\underline{H}_2$), 1.32 (6H, dd, *J* 6.4, 3.8, C$\underline{H}_{3V}$), 0.85 (6H, t, *J* 6.3, C$\underline{H}_{3V}$).
^{13}C NMR (101 MHz, $CDCl_3$ + 5 uL TFA) δ: 176.9 ($\underline{C}$OOH), 176.5 ($\underline{C}$OOH), 164.6 ($\underline{C}$=O), 164.4 ($\underline{C}$=O), 150.5 ($\underline{C}_{Ar}$-N), 136.7 ($\underline{C}_{Ar}$), 131.7 ($\underline{C}H_{Ar}$), 130.8 ($\underline{C}_{Ar}$), 128.8 ($\underline{C}_{Ar}$), 128.2 ($\underline{C}_{Ar}$), 123.4 ($\underline{C}H_{Ar}$), 122.3 ($\underline{C}_{Ar}$), 118.1 ($\underline{C}H_{Ar}$), 116.6 ($\underline{C}_{Ar}$), 116.5 ($\underline{C}_{Ar}$), 115.8 ($\underline{C}_{Ar}$), 58.8 (N-$\underline{C}H_V$), 58.7 (N-$\underline{C}H_V$), 52.9 (N-$\underline{C}H_2$), 27.6 ($\underline{C}H_V$-CH_{3V}), 27.5 ($\underline{C}H_V$-CH_{3V}), 25.7 (N-CH_2-$\underline{C}H_2$), 21.95 ($\underline{C}H_{3V}$), 21.93 ($\underline{C}H_{3V}$), 19.08 ($\underline{C}H_{3V}$), 19.06 ($\underline{C}H_{3V}$).
HRMS (ESI/QTOF) *m/z*: $[M-H]^-$ calcd for $C_{42}H_{39}N_4O_8$ 727.2777; found 727.2768.

N-Boc-Valyldimethylaspartate (P18)

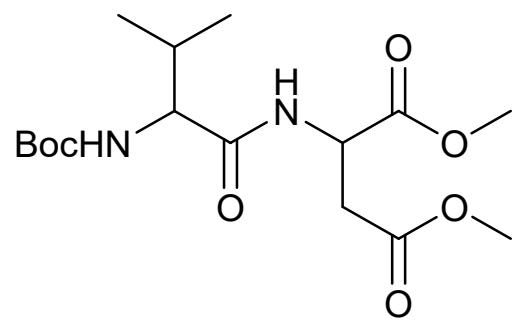

To a solution of N-Boc valine (3.04 g, 15.4 mmol) in chloroform (50 mL) was added iso-butyl chloroformate (2.00 mL, 15.4 mmol) followed by N-methylmorpholine (3.72 mL, 33.9 mmol). After stirring for 10 min, aspartic acid dimethyl ester hydrochloride (3.41 g, 15.7 mmol) was added and the reaction stirred overnight. It was then diluted with chloroform, washed in turn with aqueous hydrogen chloride (1 M), water, and brine. The layers were separated and the organic layer dried over anhydrous magnesium sulphate, filtered, and evaporated under reduced pressure. The crude oil was purified by column chromatography using an ethyl acetate/dichloromethane mixture (5:95) as eluant to afford **P18** as a colourless oil which solidified on standing (2.70 g, 49%). The presence of rotamers (ca. 85:15) is inferred from the proton NMR.
^{1}H NMR (400 MHz, DMSO-d_6) δ: 8.30 (1H, d, *J* 7.7, N$\underline{H}$), 6.66 (0.85H, d, *J* 8.9, N$\underline{H}$), 6.30 (0.15H, d, *J* 7.0, N$\underline{H}$), 4.66–4.61 (1H, m, C$\underline{H}$-NH), 3.81–3.77 (1H, m, C$\underline{H}$-NH), 3.61 (3H, s, O-C$\underline{H}_3$), 3.60 (3H, s, O-C$\underline{H}_3$), 2.82 (1H, dd, *J* 16.5, 5.9, C$\underline{H}_2$), 2.71 (1H, dd, *J* 16.5, 7.3, C$\underline{H}_2$), 1.96–1.87 (1H, C$\underline{H}$(CH_3)$_2$), 1.38 (9H, s, C(C$\underline{H}_3$)$_3$), 0.84 (3H, d, *J* 6.7, CH(C$\underline{H}_3$)$_2$), 0.80 (3H, d, *J* 6.7, CH(C$\underline{H}_3$)$_2$).
^{13}C NMR (100 MHz, DMSO-d_6) δ: 171.3, 171.0, 170.3, and 155.3 ($\underline{C}$=O), 78.0 ($\underline{C}$(CH_3)$_3$), 59.4 ($\underline{C}$H-NH), 52.0 and 51.6 (O-$\underline{C}H_3$), 48.4 ($\underline{C}$H-NH), 35.5 ($\underline{C}H_2$), 30.4 (CH($\underline{C}H_3$)$_2$), 28.1 (C($\underline{C}H_3$)$_3$), 19.0 and 17.9 (CH($\underline{C}H_3$)$_2$).
HRMS (ESI/QTOF) m/z: $[M+Na]^+$ calculated for $C_{16}H_{28}N_2NaO_7$ 383.178872; found: 383.180287.

Valyldimethylaspartate trifluoroacetate (P19)

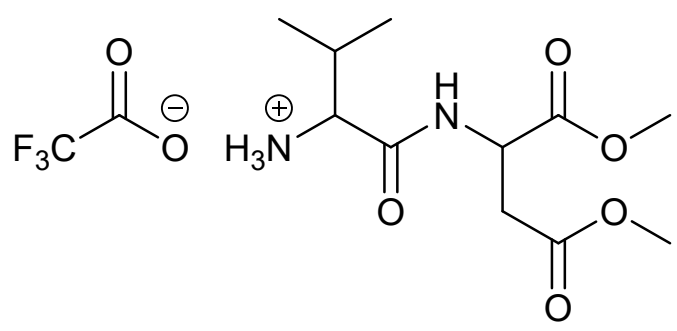

To a solution of **P18** (647 mg, 1.46 mmol) in tetrahydrofuran (15 mL, heating necessary, solution allowed to cool back to ambient temperature before next step) was added a solution of lithium hydroxide (139 mg, 5.84 mmol) in water (15 mL). The solution was stirred for 5 h, then poured into aqueous hydrochloric acid (1 M, 75 mL) and stirred overnight. The solids were filtered off, washed with water on the filter, and dried on a rotary evaporator to give **P19** as a white solid (548 mg, 90%) with *ca.* 0.4% residual tetrahydrofuran (NMR) which could not be removed after drying in a vacuum oven.

^{1}H NMR (400 MHz, DMSO-d_6) δ: 8.90 (1H, d, *J* 7.6, N$\underline{H}$), 8.09 (3H, br s, N$\underline{H}_3^+$), 4.75-4.70 (1H, m, C$\underline{H}^*$), 3.66-3.63 (1H, m, C$\underline{H}^*$), 3.64 (3H, s, OC$\underline{H}_3$), 3.63 (3H, s, OC$\underline{H}_3$), 2.84 (1H, d, *J* 1.0, C$\underline{H}_2$), 2.82 (1H, d, *J* 2.1, C$\underline{H}_2$), 2.14-2.05 (1H, m, C$\underline{H}$(CH_3)$_2$), 0.96 (3H, d, *J* 7.1, CH(C$\underline{H}_3$)$_2$), 0.94 (3H, d, *J* 7.1, CH(C$\underline{H}_3$)$_2$).
^{13}C NMR (100 MHz, DMSO-d_6) δ: 170.56, 170.27, and 167.99 ($\underline{C}$=O), 158.16 (q, *J* 30.5, O$\underline{C}$-CF_3), 117.24 (q, *J* 300.05, $\underline{C}F_3$), 57.10 ($\underline{C}H^*$), 52.26 and 51.77 (O$\underline{C}H_3$), 48.54 ($\underline{C}H^*$), 35.21 ($\underline{C}H_2$), 29.83 ($\underline{C}$H(CH_3)$_2$), 18.02 and 17.39 (CH($\underline{C}H_3$)$_2$).
HRMS (ESI/QTOF) m/z: $[M-TFA+H]^+$ calcd for $C_{11}H_{21}N_2O_5$ 261.144498; found 261.144725.

Please do not adjust margins

1Nap-VD(OMe)$_2$ (P20)

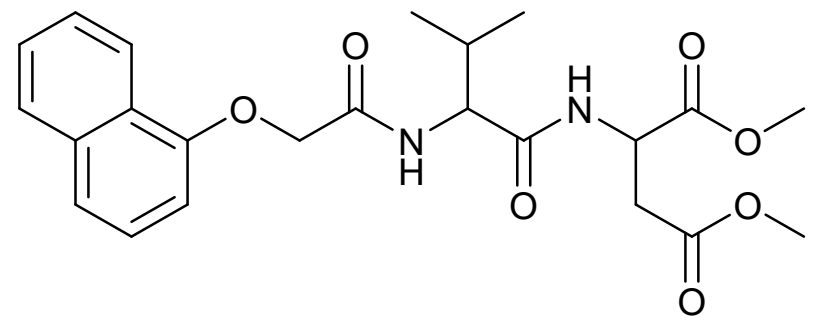

1-Naphthaleneoxyacetic acid (436 mg, 2.16 mmol) was suspended in chloroform (15 mL). *N*-methylmorpholine (522 µL, 4.75 mmol) and *iso*-butyl chloroformate (280 µL, 2.16 mmol) were added and the mixture was stirred for 3 min, upon which all solids dissolved. **P19** (816 mg, 2.18 mmol) was added and the mixture stirred overnight. The resulting mixture was diluted with chloroform, washed in turn with aqueous hydrochloric acid (1 M), water, and brine. The separated organic layer was dried over anhydrous magnesium sulphate, filtered, and evaporated to dryness under reduced pressure. The crude solid was purified over column chromatography using an ethyl acetate/dichloromethane mixture (1:9) as eluent to afford **P20** as an off-white solid (705 mg, 73%).

^{1}H NMR (400 MHz, DMSO-d$_6$) δ: 8.63 (1H, d, *J* 7.7, NH), 8.24–8.19 (1H, m, H$_{Ar}$), 8.00 (1H, d, *J* 9.0, NH), 7.92–7.86 (1H, m, H$_{Ar}$), 7.59–7.48 (3H, m, H$_{Ar}$), 7.40 (1H, t, *J* 8.0, H$_{Ar}$), 6.91 (1H, d, *J* 7.4, H$_{Ar}$), 4.82 (1H, d, *J* 14.6, O-CH$_2$), 4.78 (1H, d, *J* 14.5, O-CH$_2$), 4.69–4.63 (1H, m, CH-NH), 4.34 (1H, dd, *J* 9.0, 6.2, CH-NH), 3.62 (3H, s, O-CH$_3$), 3.60 (3H, s, O-CH$_3$), 2.84 (1H, dd, *J* 16.5, 5.9, CH-CH$_2$), 2.73 (1H, dd, *J* 16.5, 7.5, CH-CH$_2$), 2.09–1.97 (1H, m, CH(CH$_3$)$_2$), 0.87 (3H, d, *J* 6.8, CH(CH$_3$)$_2$), 0.83 (3H, d, *J* 6.8, CH(CH$_3$)$_2$).
^{13}C NMR (100 MHz, DMSO-d$_6$) δ: 170.9, 170.6, 170.3, and 167.3 (C=O), 153.1, 134.1, 127.5, 126.6, 126.0, 125.5, 124.8, 121.4, 120.6, and 105.6 (C$_{Ar}$), 66.7 (O-CH$_2$), 56.7 (CH-NH), 52.1 and 51.7 (O-CH$_3$), 48.5 (CH-NH), 35.3 (CH-CH$_2$), 31.1 (CH(CH$_3$)$_2$), 19.0 and 17.6 (CH(CH$_3$)$_2$). HRMS (ESI/QTOF) m/z: [M+Na]$^+$ calcd for $C_{23}H_{28}NaN_2O_7$ 467.178872; found: 467.180725.

1Nap-VD (54)

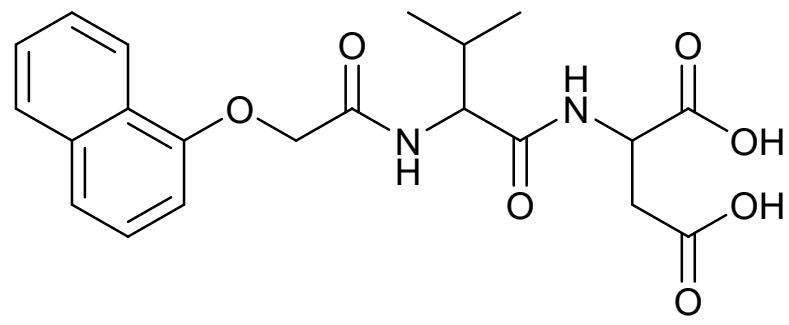

To a stirred solution of **P20** (647 mg, 1.46 mmol) in tetrahydrofuran (15 mL, heating necessary, solution allowed to cool back to ambient temperature before next step) was added a solution of lithium hydroxide (139 mg, 5.84 mmol) in water (15 mL). After 5 h the reaction mixture was poured into aqueous hydrochloric acid (1 M, 75 mL) and stirred overnight. The solids were collected at the filter, washed with water on the filter, and dried on a rotary evaporator to give **54** as a white solid (548 mg, 90%) with *ca.* 0.4% residual tetrahydrofuran (NMR) which could not be removed after drying in a vacuum oven.

^{1}H NMR (400 MHz, DMSO-d$_6$) δ: 12.59 (1H, br, COOH), 8.47 (1H, d, *J* 7.8, NH), 8.25-8.19 (1H, m, H$_{Ar}$), 7.98 (1H, d, *J* 9.1, NH), 7.92-7.86 (1H, m, H$_{Ar}$), 7.58-7.49 (3H, m, H$_{Ar}$), 7.40 (1H, t, *J* 8.0, H$_{Ar}$), 6.92 (1H, dd, *J* 7.6, 0.5, H$_{Ar}$), 4.82 (1H, d, *J* 14.5, OCH$_2$), 4.77 (1H, d, *J* 14.5, OCH$_2$), 4.58-4.52 (1H, m, CH*), 4.37 (1H, dd, *J* 9.1, 5.9, CH*), 2.72 (1H, dd, *J* 16.6, 5.7, CH*CH$_2$), 2.59 (1H, dd, *J* 16.6, 7.4, CH*CH$_2$), 2.11-1.97 (1H, m, CH(CH$_3$)$_2$), 0.87 (3H, d, *J* 6.8, CH(CH$_3$)$_2$), 0.82 (3H, d, *J* 6.8, CH(CH$_3$)$_2$).
^{13}C NMR (101 MHz, DMSO) δ: 172.2, 171.5, 170.4, and 167.2 (C=O), 153.1, 134.1, 127.5, 126.6, 126.0, 125.5, 124.8, 121.4, 120.7, and 105.7 (C$_{Ar}$), 67.0 (O-CH$_2$), 56.6 (CH-NH), 48.7 (CH-NH), 35.8 (CH-CH$_2$), 31.2 (CH(CH$_3$)$_2$), 19.1 and 17.6 (CH(CH$_3$)$_2$). HRMS (ESI/QTOF) m/z: [M+H]$^+$ calcd for $C_{21}H_{25}N_2O_7$ 417.1656; found: 417.1670.

NDI-4-Nitro-Phenylalanine (55)

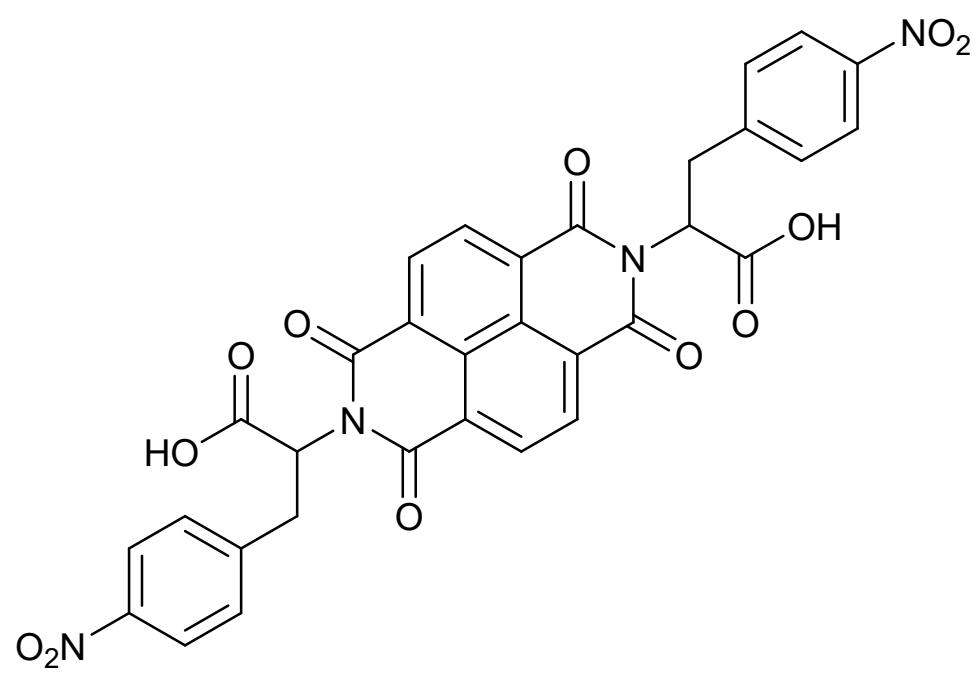

A mixture of naphthalene-1,4,5,8-tetracarboxylic acid dianhydride (NTCDA, 2.79 g, 10.4 mmol), 4-nitrophenylalanine (4.37 g, 20.8 mmol), and imidazole (14.2 g, 208 mmol) was heated to 120 °C in an oil bath under nitrogen and exclusion of light overnight. The temperature was then reduced to 90 °C, the mixture diluted approx. 1:1 with water and stirred for 1 h. After cooling to ambient temperature, the reaction mixture was filtered, then the filtrate poured into aqueous hydrochloric acid (1 M, 100 mL) and stirred for 2 h. The precipitate was filtered off, returned into aqueous hydrochloric acid (0.25 M), and stirred for 1 h. The solids were filtered again, transferred into water (1 L), and stirred overnight. The solid was collected at the filter and freeze-dried to afford **55** as a light brown solid (5.45 g, 80%).

^{1}H NMR (400 MHz, DMSO-d$_6$) δ: 13.15 (2H, br, COOH), 8.66 (4H, s, H$_{Ar}$), 8.03 (4H, d, *J* 8.7, H$_{Ar}$), 7.52 (4H, d, *J* 8.7, H$_{Ar}$), 5.92 (2H, dd, *J* 8.7, 5.7, CH-NH), 3.76 (2H, dd, *J* 14.1, 5.6, Ph-CH$_2$), 3.44 (2H, dd, *J* 14.1, 8.8, Ph-CH$_2$).
^{13}C NMR (101 MHz, DMSO) δ: 169.9 and 162.1 (C=O), 146.4, 146.2, 131.3, 130.4, 126.2, 125.7, and 123.3 (C$_{Ar}$), 54.2 (CH-NH), 34.3 (CH$_2$).

This journal is © The Royal Society of Chemistry 20xx

Please do not adjust margins

Please do not adjust margins

Section 2

Solubility Data Collection Methods

Calibration Curve Procedure: Up to 10 mg of the molecule of choice was dissolved in 2 mL of dimethyl sulfoxide (DMSO) and briefly vortex mixed using a VWR Lab Dancer. 1 mL of this solution was diluted with 1 mL of DMSO to halve the concentration and this dilution procedure was repeated recursively to generate the dilution series. Absorption spectra were obtained using a Cary 60 UV-visible spectrophotometer (Agilent Technologies). Solutions were measured in a 0.1 mm pathlength (l) quartz cuvette (Hellma Analytics or Starna Scientific). The wavelength of maximum absorption (λ_{max}) between 260 and 800 nm for the highest concentration solution in the calibration curve was determined. The λ_{max} absorption was measured across the dilution series and plotted against the associated concentrations to establish a linear Beer-Lambert relationship. The part of the plot where absorption (A) was proportional to concentration (c), the calibration window, was used to calculate the molar absorption coefficient (ε) (Equation S1). For each sample, with the exception of PBI-G **(25)**, the bounds of the calibration curve were sufficient to quantify the absorption at 1 mg mL^{-1}. The quality of the linear fit was quantified using the coefficient of determination (R^2).

The spectra and resulting calibration curve have been provided for each compound, along with key metrics of the calibration window, molar absorption coefficient and coefficient of determination are summarised in Table S1.

$$A = \epsilon c l \quad \text{(Equation S1)}$$

Solubility Classification: Approximately 40 mg portions of each compound were combined with 1.5 mL of acetone, dimethyl formamide (DMF), dimethyl sulfoxide (DMSO), ethanol, or tetrahydrofuran (THF). Each sample was agitated overnight at room temperature (*ca*. 20°C) using an SLS lab basics DigiRoll 6 before filtering through a 0.45 µm PTFE syringe filter (Fisherbrand). Solutions were prepared at a single temperature to minimise the influence of changing temperature on the measured solubility. The filtered solutions were evaporated to dryness under vacuum and reconstituted in DMSO (with the exception of those already solvated in DMSO). Absorption spectra were obtained using a Cary 60 UV-visible spectrophotometer (Agilent Technologies). Solutions were measured in a 0.01 mm or 0.1 mm pathlength quartz cuvette (Hellma Analytics or Starna Scientific). Solubility classification was determined from molar absorption coefficients and dilution history. Compounds were classified as soluble when solubility exceeded 10 mg mL^{-1}. All measurements in the 0.1 mm cuvettes fell withing the calibration window. Many measurements in the 0.01 mm cuvettes fell outside the calibration window, however, as the absorption of 1 mg mL^{-1} in a 0.1 mm cuvette was within the calibration window, the absorbance of the classification threshold of 10 mg mL^{-1} was known for a 0.01 mm cuvette. Therefore, solubility samples in a 0.01 mm cuvette with an absorbance at λ_{max} exceeding the absorbance afforded by 1 mg mL^{-1} in a 0.1 mm cuvette were classified as soluble, and those with an absorbance below were classified as insoluble.

PBI-G **(25)** was the only exception where the calibration window fell below 1 mg mL^{-1}, with classification measurements falling within the calibration window or below. Therefore, PBI-G could be classified as insoluble for each solvent.

The spectra and resulting Beer-Lamber calculations for each compound and solvent has been summarised in Table S2, with the resulting binary classifications provided in Table S3.

This journal is © The Royal Society of Chemistry 20xx

Please do not adjust margins

Solubility Data

Table S1 UV-vis dilution series and calibration curve in DMSO for each training, test, and validation compound. In each case the highest concentration spectra is given in red (▬) and the lowest in purple (▬). Dotted spectra show datapoints that deviate from linearity, indicating saturation at the lower calibration concentration and are subsequently rejected from the calibration curve. The calibration window for a measurement in a 0.1 mm cuvette, molar absorption coefficient, and R^2 goodness-of-fit metrics are reported.

Wavelength (nm)	Concentration (M)	**2Nap-FF (1)**	
		Min Conc. (mg mL^{-1})	0.041
		Max Conc. (mg mL^{-1})	2.625
		Molar Absorption Coefficient (M^{-1} cm^{-1})	5463
		Calibration Linear Fit R^2 (au)	0.99
Wavelength (nm)	Concentration (M)	**2Nap-V (2)**	
		Min Conc. (mg mL^{-1})	0.050
		Max Conc. (mg mL^{-1})	3.300
		Molar Absorption Coefficient (M^{-1} cm^{-1})	5157
		Calibration Linear Fit R^2 (au)	1.00
Wavelength (nm)	Concentration (M)	**2Nap-VVV (3)**	
		Min Conc. (mg mL^{-1})	0.175
		Max Conc. (mg mL^{-1})	2.800
		Molar Absorption Coefficient (M^{-1} cm^{-1})	4853
		Calibration Linear Fit R^2 (au)	1.00
Wavelength (nm)	Concentration (M)	**2Nap-IF (4)**	
		Min Conc. (mg mL^{-1})	0.038
		Max Conc. (mg mL^{-1})	2.430
		Molar Absorption Coefficient (M^{-1} cm^{-1})	5104
		Calibration Linear Fit R^2 (au)	1.00

This journal is © The Royal Society of Chemistry 20xx
Please do not adjust margins

		**6Br2Nap-VF (5)**	
		Min Conc. (mg mL^{-1})	0.065
		Max Conc. (mg mL^{-1})	4.200
		Molar Absorption Coefficient (M^{-1} cm^{-1})	6461
Wavelength (nm)	Concentration (M)	Calibration Linear Fit R^2 (au)	1.00
		**6MeO2Nap-FI (6)**	
		Min Conc. (mg mL^{-1})	0.081
		Max Conc. (mg mL^{-1})	2.600
		Molar Absorption Coefficient (M^{-1} cm^{-1})	6540
Wavelength (nm)	Concentration (M)	Calibration Linear Fit R^2 (au)	1.00
		**7MeO2Nap-FV (7)**	
		Min Conc. (mg mL^{-1})	0.041
		Max Conc. (mg mL^{-1})	2.600
		Molar Absorption Coefficient (M^{-1} cm^{-1})	3071
Wavelength (nm)	Concentration (M)	Calibration Linear Fit R^2 (au)	0.99
		**4ClNap-LIF (8)**	
		Min Conc. (mg mL^{-1})	0.035
		Max Conc. (mg mL^{-1})	2.250
		Molar Absorption Coefficient (M^{-1} cm^{-1})	7498
Wavelength (nm)	Concentration (M)	Calibration Linear Fit R^2 (au)	1.00
		**NDI-F (9)**	
		Min Conc. (mg mL^{-1})	0.040
		Max Conc. (mg mL^{-1})	2.550
		Molar Absorption Coefficient (M^{-1} cm^{-1})	19931
Wavelength (nm)	Concentration (M)	Calibration Linear Fit R^2 (au)	1.00

		**NDI-FF (10)**	
		Min Conc. (mg mL^{-1})	0.189
		Max Conc. (mg mL^{-1})	3.030
		Molar Absorption Coefficient (M^{-1} cm^{-1})	23486
Wavelength (nm)	Concentration (M)	Calibration Linear Fit R^2 (au)	1.00
		**NDI-G (11)**	
		Min Conc. (mg mL^{-1})	0.020
		Max Conc. (mg mL^{-1})	2.550
		Molar Absorption Coefficient (M^{-1} cm^{-1})	19152
Wavelength (nm)	Concentration (M)	Calibration Linear Fit R^2 (au)	1.00
		**NDI-GF (12)**	
		Min Conc. (mg mL^{-1})	0.050
		Max Conc. (mg mL^{-1})	3.230
		Molar Absorption Coefficient (M^{-1} cm^{-1})	16711
Wavelength (nm)	Concentration (M)	Calibration Linear Fit R^2 (au)	1.00
		**NDI-A (13)**	
		Min Conc. (mg mL^{-1})	0.041
		Max Conc. (mg mL^{-1})	2.650
		Molar Absorption Coefficient (M^{-1} cm^{-1})	17616
Wavelength (nm)	Concentration (M)	Calibration Linear Fit R^2 (au)	1.00
		**NDI-I (14)**	
		Min Conc. (mg mL^{-1})	0.080
		Max Conc. (mg mL^{-1})	2.580
		Molar Absorption Coefficient (M^{-1} cm^{-1})	20552
Wavelength (nm)	Concentration (M)	Calibration Linear Fit R^2 (au)	1.00

This journal is © The Royal Society of Chemistry 20xx
Please do not adjust margins
Please do not adjust margins

Wavelength (nm)	Concentration (M)	**NDI-L (15)**	
		Min Conc. (mg mL^{-1})	0.039
		Max Conc. (mg mL^{-1})	2.530
		Molar Absorption Coefficient (M^{-1} cm^{-1})	20230
		Calibration Linear Fit R^2 (au)	1.00
Wavelength (nm)	Concentration (M)	**NDI-M (16)**	
		Min Conc. (mg mL^{-1})	0.020
		Max Conc. (mg mL^{-1})	2.600
		Molar Absorption Coefficient (M^{-1} cm^{-1})	19660
		Calibration Linear Fit R^2 (au)	1.00
Wavelength (nm)	Concentration (M)	**NDI-V (17)**	
		Min Conc. (mg mL^{-1})	0.041
		Max Conc. (mg mL^{-1})	2.650
		Molar Absorption Coefficient (M^{-1} cm^{-1})	20309
		Calibration Linear Fit R^2 (au)	1.00
Wavelength (nm)	Concentration (M)	**NDI-W (18)**	
		Min Conc. (mg mL^{-1})	0.038
		Max Conc. (mg mL^{-1})	2.400
		Molar Absorption Coefficient (M^{-1} cm^{-1})	20751
		Calibration Linear Fit R^2 (au)	1.00
Wavelength (nm)	Concentration (M)	**NDI-Y (19)**	
		Min Conc. (mg mL^{-1})	0.038
		Max Conc. (mg mL^{-1})	2.450
		Molar Absorption Coefficient (M^{-1} cm^{-1})	20930
		Calibration Linear Fit R^2 (au)	1.00

Please do not adjust margins

		**PBI-A (20)**	
		Min Conc. (mg mL^{-1})	0.045
		Max Conc. (mg mL^{-1})	1.450
		Molar Absorption Coefficient (M^{-1} cm^{-1})	41631
Wavelength (nm)	Concentration (M)	Calibration Linear Fit R^2 (au)	1.00
		**PBI-D (21)**	
		Min Conc. (mg mL^{-1})	0.013
		Max Conc. (mg mL^{-1})	1.640
		Molar Absorption Coefficient (M^{-1} cm^{-1})	55454
Wavelength (nm)	Concentration (M)	Calibration Linear Fit R^2 (au)	1.00
		**PBI-DOPA (22)**	
		Min Conc. (mg mL^{-1})	0.044
		Max Conc. (mg mL^{-1})	2.800
		Molar Absorption Coefficient (M^{-1} cm^{-1})	39500
Wavelength (nm)	Concentration (M)	Calibration Linear Fit R^2 (au)	0.99
		**PBI-E (23)**	
		Min Conc. (mg mL^{-1})	0.041
		Max Conc. (mg mL^{-1})	1.300
		Molar Absorption Coefficient (M^{-1} cm^{-1})	47055
Wavelength (nm)	Concentration (M)	Calibration Linear Fit R^2 (au)	0.99
		**PBI-F (24)**	
		Min Conc. (mg mL^{-1})	0.044
		Max Conc. (mg mL^{-1})	1.410
		Molar Absorption Coefficient (M^{-1} cm^{-1})	49572
Wavelength (nm)	Concentration (M)	Calibration Linear Fit R^2 (au)	0.98

This journal is © The Royal Society of Chemistry 20xx

Please do not adjust margins

		**PBI-G (25)**	
		Min Conc. (mg mL^{-1})	0.039
		Max Conc. (mg mL^{-1})	0.150
		Molar Absorption Coefficient (M^{-1} cm^{-1})	23222
Wavelength (nm)	Concentration (M)	Calibration Linear Fit R^2 (au)	0.97
		**PBI-H (26)**	
		Min Conc. (mg mL^{-1})	0.039
		Max Conc. (mg mL^{-1})	1.260
		Molar Absorption Coefficient (M^{-1} cm^{-1})	33619
Wavelength (nm)	Concentration (M)	Calibration Linear Fit R^2 (au)	0.99
		**PBI-I (27)**	
		Min Conc. (mg mL^{-1})	0.042
		Max Conc. (mg mL^{-1})	1.340
		Molar Absorption Coefficient (M^{-1} cm^{-1})	66426
Wavelength (nm)	Concentration (M)	Calibration Linear Fit R^2 (au)	1.00
		**PBI-L (28)**	
		Min Conc. (mg mL^{-1})	0.088
		Max Conc. (mg mL^{-1})	2.830
		Molar Absorption Coefficient (M^{-1} cm^{-1})	24057
Wavelength (nm)	Concentration (M)	Calibration Linear Fit R^2 (au)	0.99
		**PBI-M (29)**	
		Min Conc. (mg mL^{-1})	0.046
		Max Conc. (mg mL^{-1})	1.480
		Molar Absorption Coefficient (M^{-1} cm^{-1})	59885
Wavelength (nm)	Concentration (M)	Calibration Linear Fit R^2 (au)	0.97

This journal is © The Royal Society of Chemistry 20xx

Please do not adjust margins

Wavelength (nm)	Concentration (M)	**PBI-S (30)**	
		Min Conc. (mg mL^{-1})	0.047
		Max Conc. (mg mL^{-1})	1.500
		Molar Absorption Coefficient (M^{-1} cm^{-1})	41343
		Calibration Linear Fit R^2 (au)	1.00
Wavelength (nm)	Concentration (M)	**PBI-V (31)**	
		Min Conc. (mg mL^{-1})	0.043
		Max Conc. (mg mL^{-1})	1.390
		Molar Absorption Coefficient (M^{-1} cm^{-1})	47714
		Calibration Linear Fit R^2 (au)	1.00
Wavelength (nm)	Concentration (M)	**PBI-W (32)**	
		Min Conc. (mg mL^{-1})	0.041
		Max Conc. (mg mL^{-1})	2.650
		Molar Absorption Coefficient (M^{-1} cm^{-1})	40852
		Calibration Linear Fit R^2 (au)	1.00
Wavelength (nm)	Concentration (M)	**PBI-Y (33)**	
		Min Conc. (mg mL^{-1})	0.043
		Max Conc. (mg mL^{-1})	1.390
		Molar Absorption Coefficient (M^{-1} cm^{-1})	42831
		Calibration Linear Fit R^2 (au)	1.00
Wavelength (nm)	Concentration (M)	**ThNap-FF (34)**	
		Min Conc. (mg mL^{-1})	0.019
		Max Conc. (mg mL^{-1})	2.400
		Molar Absorption Coefficient (M^{-1} cm^{-1})	1149
		Calibration Linear Fit R^2 (au)	0.99

This journal is © The Royal Society of Chemistry 20xx
Please do not adjust margins
Please do not adjust margins

Please do not adjust margins

		**2Nap-VL (35)**	
		Min Conc. (mg mL^{-1})	0.042
		Max Conc. (mg mL^{-1})	2.700
		Molar Absorption Coefficient (M^{-1} cm^{-1})	4698
Wavelength (nm)	Concentration (M)	Calibration Linear Fit R^2 (au)	1.00
		**2Nap-FL (36)**	
		Min Conc. (mg mL^{-1})	0.080
		Max Conc. (mg mL^{-1})	2.550
		Molar Absorption Coefficient (M^{-1} cm^{-1})	4636
Wavelength (nm)	Concentration (M)	Calibration Linear Fit R^2 (au)	1.00
		**2Nap-FI (37)**	
		Min Conc. (mg mL^{-1})	0.116
		Max Conc. (mg mL^{-1})	3.700
		Molar Absorption Coefficient (M^{-1} cm^{-1})	4880
Wavelength (nm)	Concentration (M)	Calibration Linear Fit R^2 (au)	1.00
		**2Nap-VF (38)**	
		Min Conc. (mg mL^{-1})	0.040
		Max Conc. (mg mL^{-1})	2.550
		Molar Absorption Coefficient (M^{-1} cm^{-1})	4781
Wavelength (nm)	Concentration (M)	Calibration Linear Fit R^2 (au)	1.00
		**2Nap-IV (39)**	
		Min Conc. (mg mL^{-1})	0.163
		Max Conc. (mg mL^{-1})	2.600
		Molar Absorption Coefficient (M^{-1} cm^{-1})	4624
Wavelength (nm)	Concentration (M)	Calibration Linear Fit R^2 (au)	1.00

This journal is © The Royal Society of Chemistry 20xx

Please do not adjust margins

Wavelength (nm)

Concentration (M)

**CF_3Ph-FL (40)**	
Min Conc. (mg mL^{-1})	0.275
Max Conc. (mg mL^{-1})	2.200
Molar Absorption Coefficient (M^{-1} cm^{-1})	1107
Calibration Linear Fit R^2 (au)	0.99

Wavelength (nm)

Concentration (M)

**CF_3Ph-FV (41)**	
Min Conc. (mg mL^{-1})	0.172
Max Conc. (mg mL^{-1})	2.750
Molar Absorption Coefficient (M^{-1} cm^{-1})	965
Calibration Linear Fit R^2 (au)	0.96

Wavelength (nm)

Concentration (M)

**2Nap-VI (42)**	
Min Conc. (mg mL^{-1})	0.036
Max Conc. (mg mL^{-1})	2.300
Molar Absorption Coefficient (M^{-1} cm^{-1})	4804
Calibration Linear Fit R^2 (au)	1.00

Wavelength (nm)

Concentration (M)

**C_6-NDI-A (43)**	
Min Conc. (mg mL^{-1})	0.021
Max Conc. (mg mL^{-1})	2.750
Molar Absorption Coefficient (M^{-1} cm^{-1})	19357
Calibration Linear Fit R^2 (au)	1.00

Wavelength (nm)

Concentration (M)

**C_6-NDI-F (44)**	
Min Conc. (mg mL^{-1})	0.017
Max Conc. (mg mL^{-1})	2.130
Molar Absorption Coefficient (M^{-1} cm^{-1})	20482
Calibration Linear Fit R^2 (au)	1.00

This journal is © The Royal Society of Chemistry 20xx

Please do not adjust margins

Wavelength (nm)	Concentration (M)	**C_6-NDI-L (45)**	
		Min Conc. (mg mL^{-1})	0.078
		Max Conc. (mg mL^{-1})	2.500
		Molar Absorption Coefficient (M^{-1} cm^{-1})	20710
		Calibration Linear Fit R^2 (au)	1.00
Wavelength (nm)	Concentration (M)	**NDI-GA (46)**	
		Min Conc. (mg mL^{-1})	0.022
		Max Conc. (mg mL^{-1})	2.850
		Molar Absorption Coefficient (M^{-1} cm^{-1})	20959
		Calibration Linear Fit R^2 (au)	1.00
Wavelength (nm)	Concentration (M)	**NDI-GL (47)**	
		Min Conc. (mg mL^{-1})	0.023
		Max Conc. (mg mL^{-1})	2.950
		Molar Absorption Coefficient (M^{-1} cm^{-1})	20864
		Calibration Linear Fit R^2 (au)	1.00
Wavelength (nm)	Concentration (M)	**NDI-LA (48)**	
		Min Conc. (mg mL^{-1})	0.020
		Max Conc. (mg mL^{-1})	2.500
		Molar Absorption Coefficient (M^{-1} cm^{-1})	19712
		Calibration Linear Fit R^2 (au)	1.00
Wavelength (nm)	Concentration (M)	**NDI-YI (49)**	
		Min Conc. (mg mL^{-1})	0.021
		Max Conc. (mg mL^{-1})	2.680
		Molar Absorption Coefficient (M^{-1} cm^{-1})	20715
		Calibration Linear Fit R^2 (au)	1.00

This journal is © The Royal Society of Chemistry 20xx

		**PBI-A 1-Py (50)**	
		Min Conc. (mg mL^{-1})	0.020
		Max Conc. (mg mL^{-1})	2.600
		Molar Absorption Coefficient (M^{-1} cm^{-1})	19524
Wavelength (nm)	Concentration (M)	Calibration Linear Fit R^2 (au)	1.00
		**PBI-I 1-Py (51)**	
		Min Conc. (mg mL^{-1})	0.019
		Max Conc. (mg mL^{-1})	2.450
		Molar Absorption Coefficient (M^{-1} cm^{-1})	23113
Wavelength (nm)	Concentration (M)	Calibration Linear Fit R^2 (au)	1.00
		**PBI-L 1-Py (52)**	
		Min Conc. (mg mL^{-1})	0.038
		Max Conc. (mg mL^{-1})	2.450
		Molar Absorption Coefficient (M^{-1} cm^{-1})	24806
Wavelength (nm)	Concentration (M)	Calibration Linear Fit R^2 (au)	1.00
		**PBI-V 1Py (53)**	
		Min Conc. (mg mL^{-1})	0.018
		Max Conc. (mg mL^{-1})	2.300
		Molar Absorption Coefficient (M^{-1} cm^{-1})	19589
Wavelength (nm)	Concentration (M)	Calibration Linear Fit R^2 (au)	1.00
		**1Nap-VD (54)**	
		Min Conc. (mg mL^{-1})	0.040
		Max Conc. (mg mL^{-1})	2.500
		Molar Absorption Coefficient (M^{-1} cm^{-1})	6536
Wavelength (nm)	Concentration (M)	Calibration Linear Fit R^2 (au)	1.00

This journal is © The Royal Society of Chemistry 20xx
Please do not adjust margins

	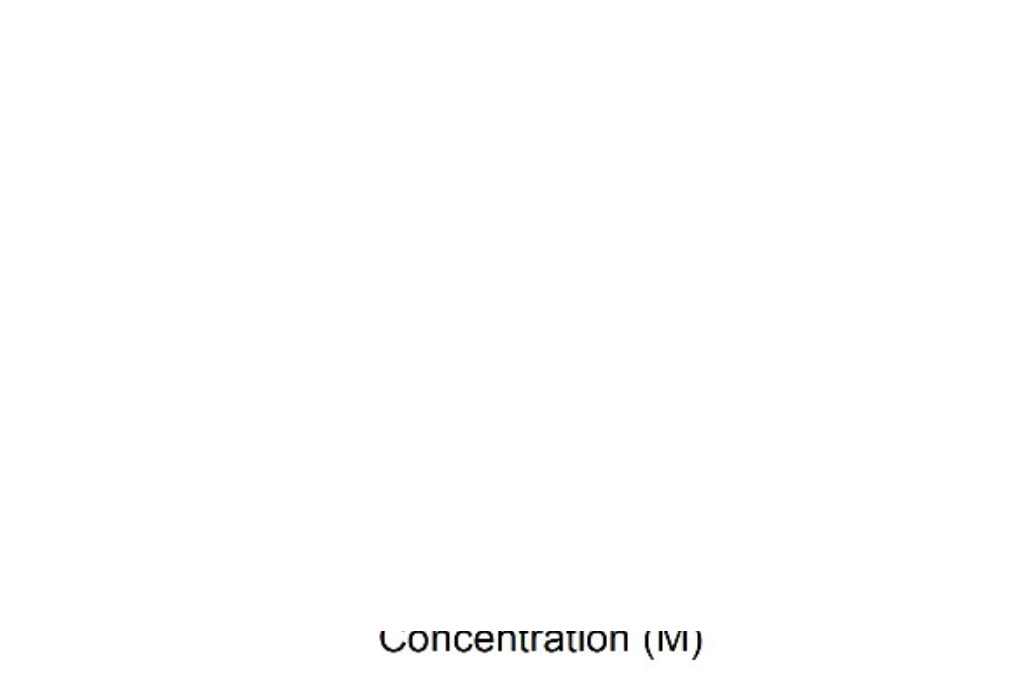	**NDI-4-Nitro-Phenylalanine (55)**	
		Min Conc. (mg mL^{-1})	0.041
		Max Conc. (mg mL^{-1})	2.600
		Molar Absorption Coefficient (M^{-1} cm^{-1})	24648
		Calibration Linear Fit R^2 (au)	1.00

This journal is © The Royal Society of Chemistry 20xx

Table S2 Solubility calculation for each training, test, and validation compound. In each case spectra of the DMSO reconstituted sample is given for each solvent: acetone ( ), DMF ( ), DMSO ( ), ethanol ( ), and THF ( ). Using the calibration curve $\lambda_{max,}$ its associated absorbance, the dilution factor, and a known path length of 0.01 mm, the concentration of each sample can be determine using the Beer-Lambert relationship.

2Nap-FF (1)

Solvent	λ_{max} (nm)	λ_{max} Absorbance (au)	Dilution Factor (au)	Path Length (cm)	Concentration (mg mL^{-1})
Acetone		0.4089	2	0.01	7.4334
DMF		0.38898	1	0.001	35.35638
DMSO	273.5	0.26686	1	0.001	24.25627
Ethanol		0.12824	1	0.001	11.65639
THF		0.1792	1	0.001	16.2884

2Nap-V (2)

Solvent	λ_{max} (nm)	λ_{max} Absorbance (au)	Dilution Factor (au)	Path Length (cm)	Concentration (mg mL^{-1})
Acetone		0.29718	1	0.001	17.36518
DMF		0.09855	4	0.001	23.03437
DMSO	276.0	0.20996	4	0.001	49.07454
Ethanol		0.19817	1	0.001	11.57971
THF		0.51424	1	0.001	30.04869

2Nap-VVV (3)

Solvent	λ_{max} (nm)	λ_{max} Absorbance (au)	Dilution Factor (au)	Path Length (cm)	Concentration (mg mL^{-1})
Acetone		0.08093	1	0.001	8.33164
DMF		0.08851	4	0.001	36.44795
DMSO	273.5	0.06275	4	0.001	25.84012
Ethanol		0.20368	1	0.001	20.96859
THF		0.06536	4	0.001	26.91491

2Nap-IF (4)

Solvent	λ_{max} (nm)	λ_{max} Absorbance (au)	Dilution Factor (au)	Path Length (cm)	Concentration (mg mL^{-1})
Acetone		0.87422	2	0.01	16.15389
DMF		0.24068	1	0.001	22.23649
DMSO	273.5	0.04924	4	0.001	18.19719
Ethanol		0.12824	1	0.001	11.84813
THF		0.44533	1	0.001	41.14416

6Br2Nap-VF (5)

Solvent	λ_{max} (nm)	λ_{max} Absorbance (au)	Dilution Factor (au)	Path Length (cm)	Concentration (mg mL^{-1})
Acetone		0.21927	1	0.001	17.8993
DMF		0.33442	1	0.001	27.29915
DMSO	275.5	0.32799	1	0.001	26.77426
Ethanol		0.07298	1	0.001	5.95745
THF		0.4418	1	0.001	36.06472

Wavelength (nm)

6MeO2Nap-FI (6)					
**Solvent**	**λ_{max} (nm)**	**λ_{max} Absorbance (au)**	**Dilution Factor (au)**	**Path Length (cm)**	**Concentration (mg mL^{-1})**
Acetone	259.0	0.43373	1	0.001	32.66703
DMF		0.37156	1	0.001	27.9846
DMSO		0.47426	1	0.001	35.71961
Ethanol		0.31768	1	0.001	23.92655
THF		0.33669	1	0.001	25.35832

Wavelength (nm)

7MeO2Nap-FV (7)					
**Solvent**	**λ_{max} (nm)**	**λ_{max} Absorbance (au)**	**Dilution Factor (au)**	**Path Length (cm)**	**Concentration (mg mL^{-1})**
Acetone	325.5	0.04202	1	0.001	6.54779
DMF		0.18443	1	0.001	28.73889
DMSO		0.03611	4	0.001	22.50743
Ethanol		0.01844	1	0.001	2.87342
THF		0.18033	1	0.001	28.10001

Wavelength (nm)

4ClNap-LIF (8)					
**Solvent**	**λ_{max} (nm)**	**λ_{max} Absorbance (au)**	**Dilution Factor (au)**	**Path Length (cm)**	**Concentration (mg mL^{-1})**
Acetone	302.0	0.01119	1	0.001	0.88965
DMF		0.21661	1	0.001	17.22133
DMSO		0.08527	4	0.001	27.11718
Ethanol		--	--	--	--
THF		0.03271	1	0.001	2.60057

Wavelength (nm)

NDI-F (9)					
**Solvent**	**λ_{max} (nm)**	**λ_{max} Absorbance (au)**	**Dilution Factor (au)**	**Path Length (cm)**	**Concentration (mg mL^{-1})**
Acetone	380.5	0.35954	4	0.01	4.05904
DMF		0.22339	40	0.01	25.21972
DMSO		0.22847	40	0.01	25.79323
Ethanol		0.10986	10	0.01	3.10067
THF		0.4858	20	0.01	27.42231

Wavelength (nm)

NDI-FF (10)					
**Solvent**	**λ_{max} (nm)**	**λ_{max} Absorbance (au)**	**Dilution Factor (au)**	**Path Length (cm)**	**Concentration (mg mL^{-1})**
Acetone	358.5	0.3002	10	0.01	10.95284
DMF		0.19048	40	0.01	27.79876
DMSO		0.17268	40	0.01	25.20102
Ethanol		0.22891	10	0.01	8.35181
THF		0.39918	10	0.01	14.56414

Please do not adjust margins

Wavelength (nm)

NDI-G (11)					
Solvent	λ_{max} (nm)	λ_{max} Absorbance (au)	Dilution Factor (au)	Path Length (cm)	Concentration (mg mL^{-1})
Acetone	379.5	0.40382	1	0.01	8.06038
DMF		0.87163	1	0.001	17.39801
DMSO		1.43124	1	0.01	28.568
Ethanol		0.00382	1	0.001	0.07625
THF		0.01597	1	0.001	0.31877

Wavelength (nm)
Wavelength (nm)

NDI-GF (12)					
Solvent	λ_{max} (nm)	λ_{max} Absorbance (au)	Dilution Factor (au)	Path Length (cm)	Concentration (mg mL^{-1})
Acetone	379.5	0.11218	0.01	5	2.27112
DMF		0.11426	0.01	40	18.50587
DMSO		0.31172	0.01	40	50.48704
Ethanol		0.09815	0.001	1	3.97416
THF		0.29539	0.001	1	11.96055

Wavelength (nm)

NDI-A (13)					
Solvent	λ_{max} (nm)	λ_{max} Absorbance (au)	Dilution Factor (au)	Path Length (cm)	Concentration (mg mL^{-1})
Acetone	360.0	0.27605	10	0.01	6.4302
DMF		0.10703	80	0.01	19.94491
DMSO		0.11179	80	0.01	20.83193
Ethanol		0.24874	10	0.01	5.79405
THF		0.25932	40	0.01	24.16198

Wavelength (nm)

NDI-I (14)					
Solvent	λ_{max} (nm)	λ_{max} Absorbance (au)	Dilution Factor (au)	Path Length (cm)	Concentration (mg mL^{-1})
Acetone		0.27921	40	0.01	26.8722
DMF		0.27352	40	0.01	26.32457
DMSO		0.26272	40	0.01	25.28514
Ethanol		0.23613	40	0.01	22.72602
THF		0.52167	20	0.01	25.10372

Wavelength (nm)

NDI-L (15)					
Solvent	λ_{max} (nm)	λ_{max} Absorbance (au)	Dilution Factor (au)	Path Length (cm)	Concentration (mg mL^{-1})
Acetone	381.0	0.42369	20	0.01	20.71327
DMF		0.47957	20	0.01	23.44512
DMSO		0.13437	80	0.01	26.27621
Ethanol		0.28852	40	0.01	28.21021
THF		0.53643	20	0.01	26.22488

This journal is © The Royal Society of Chemistry 20xx

NDI-M (16)					
**Solvent**	**λ_{max} (nm)**	**λ_{max} Absorbance (au)**	**Dilution Factor (au)**	**Path Length (cm)**	**Concentration (mg mL^{-1})**
Acetone	381.0	0.23232	0.001	4	25.07874
DMF		0.25811	0.001	4	27.86275
DMSO		0.17051	0.001	4	18.40641
Ethanol		0.84867	0.001	1	22.9033
THF		0.65951	0.001	4	71.19353

NDI-V (17)					
**Solvent**	**λ_{max} (nm)**	**λ_{max} Absorbance (au)**	**Dilution Factor (au)**	**Path Length (cm)**	**Concentration (mg mL^{-1})**
Acetone		0.16797	40	0.01	15.4315
DMF		0.13299	80	0.01	24.43574
DMSO		0.14583	80	0.01	26.79498
Ethanol		0.15862	40	0.01	14.57251
THF		0.25217	40	0.01	23.16701

NDI-W (18)					
**Solvent**	**λ_{max} (nm)**	**λ_{max} Absorbance (au)**	**Dilution Factor (au)**	**Path Length (cm)**	**Concentration (mg mL^{-1})**
Acetone	381.0	0.12717	50	0.01	19.62951
DMF		0.70819	1	0.001	21.86273
DMSO		1.88711	4	0.01	23.303
Ethanol		0.15445	1	0.001	4.76807
THF		0.64836	1	0.001	20.01571

NDI-Y (19)					
**Solvent**	**λ_{max} (nm)**	**λ_{max} Absorbance (au)**	**Dilution Factor (au)**	**Path Length (cm)**	**Concentration (mg mL^{-1})**
Acetone	380.5	0.10804	50	0.01	15.34473
DMF		1.48566	1	0.001	42.20112
DMSO		0.7766	1	0.001	22.05982
Ethanol		0.96227	1	0.001	27.33389
THF		0.61741	1	0.001	17.53792

PBI-A (20)					
**Solvent**	**λ_{max} (nm)**	**λ_{max} Absorbance (au)**	**Dilution Factor (au)**	**Path Length (cm)**	**Concentration (mg mL^{-1})**
Acetone	380.5	0.10633	1	0.01	0.13651
DMF		0.17882	10	0.01	2.29578
DMSO		0.78319	1	0.001	10.05499
Ethanol		-0.00126	1	0.001	0.00
THF		0.012	1	0.001	0.15406

PBI-D (21)

Solvent	λ_{max} (nm)	λ_{max} Absorbance (au)	Dilution Factor (au)	Path Length (cm)	Concentration (mg mL^{-1})
Acetone		0.00306	1	0.001	0.03435
DMF		0.41261	1	0.001	4.63176
DMSO	529.5	1.18889	1	0.001	13.34591
Ethanol		0.00101	1	0.001	0.01134
THF		0.02604	1	0.001	0.29231

PBI-DOPA (22)

Solvent	λ_{max} (nm)	λ_{max} Absorbance (au)	Dilution Factor (au)	Path Length (cm)	Concentration (mg mL^{-1})
Acetone		0.10851	80	0.01	16.49726
DMF		0.17119	80	0.01	26.02677
DMSO	531.5	0.16052	80	0.01	24.40457
Ethanol		0.16079	80	0.01	24.44562
THF		0.20307	80	0.01	30.87363

PBI-E (23)

Solvent	λ_{max} (nm)	λ_{max} Absorbance (au)	Dilution Factor (au)	Path Length (cm)	Concentration (mg mL^{-1})
Acetone		0.0024	1	0.001	0.03318
DMF		1.29903	1	0.001	17.95949
DMSO	529.0	1.43923	1	0.001	19.8978
Ethanol		0.00158	1	0.001	0.02184
THF		0.04742	1	0.001	0.6556

PBI-F (24)

Solvent	λ_{max} (nm)	λ_{max} Absorbance (au)	Dilution Factor (au)	Path Length (cm)	Concentration (mg mL^{-1})
Acetone		0.3283	4	0.01	1.81907
DMF		0.42565	20	0.01	11.79236
DMSO	529.5	0.17662	80	0.01	19.57257
Ethanol		0.52657	1	0.01	0.72941
THF		0.12866	40	0.01	7.12888

PBI-G (25)

Solvent	λ_{max} (nm)	λ_{max} Absorbance (au)	Dilution Factor (au)	Path Length (cm)	Concentration (mg mL^{-1})
Acetone		0.001	1	0.001	0.06168
DMF		0.09863	1	0.01	0.60832
DMSO	524.5	0.04939	4	0.01	1.21849
Ethanol		0.00362	1	0.01	0.02233
THF		0.0011	1	0.01	0.00678

This journal is © The Royal Society of Chemistry 20xx
Please do not adjust margins

PBI-H (26)

Solvent	λ_{max} (nm)	λ_{max} Absorbance (au)	Dilution Factor (au)	Path Length (cm)	Concentration (mg mL^{-1})
Acetone	524.5	0.10633	1	0.001	3.20234
DMF		0.17882	1	0.001	5.38552
DMSO		0.78319	5	0.01	11.79367
Ethanol		-0.00126	1	0.001	0.00
THF		0.012	1	0.001	0.3614

PBI-I (27)

Solvent	λ_{max} (nm)	λ_{max} Absorbance (au)	Dilution Factor (au)	Path Length (cm)	Concentration (mg mL^{-1})
Acetone	531.0	1.19857	1	0.01	1.11625
DMF		0.39181	1	0.001	3.64901
DMSO		0.43931	1	0.001	4.09139
Ethanol		0.48875	1	0.01	0.45518
THF		0.30992	1	0.001	2.88635

PBI-L (28)

Solvent	λ_{max} (nm)	λ_{max} Absorbance (au)	Dilution Factor (au)	Path Length (cm)	Concentration (mg mL^{-1})
Acetone	530.0	0.13594	20	0.01	6.99156
DMF		0.07203	80	0.01	14.81835
DMSO		0.10301	80	0.01	21.19171
Ethanol		0.22969	10	0.01	5.90661
THF		0.15465	80	0.01	31.81533

PBI-M (29)

Solvent	λ_{max} (nm)	λ_{max} Absorbance (au)	Dilution Factor (au)	Path Length (cm)	Concentration (mg mL^{-1})
Acetone	531.0	0.00258	1	0.001	0.02821
DMF		0.06778	1	0.001	0.74102
DMSO		0.20913	1	0.001	2.28637
Ethanol		0.00293	1	0.001	0.03203
THF		0.03869	1	0.01	0.0423

PBI-S (30)

Solvent	λ_{max} (nm)	λ_{max} Absorbance (au)	Dilution Factor (au)	Path Length (cm)	Concentration (mg mL^{-1})
Acetone	529.5	0.01017	1	0.001	0.13148
DMF		0.84519	1	0.001	10.92657
DMSO		0.25515	80	0.01	26.38852
Ethanol		0.00328	1	0.01	0.00424
THF		0.00975	1	0.001	0.12605

PBI-V (31)

Solvent	λ_{max} (nm)	λ_{max} Absorbance (au)	Dilution Factor (au)	Path Length (cm)	Concentration (mg mL^{-1})
Acetone	530.5	0.22859	1	0.01	0.28294
DMF		0.35244	20	0.01	8.7248
DMSO		0.11977	80	0.01	11.85983
Ethanol		0.1625	1	0.001	2.01138
THF		0.4263	1	0.001	5.27662

PBI-W (32)

Solvent	λ_{max} (nm)	λ_{max} Absorbance (au)	Dilution Factor (au)	Path Length (cm)	Concentration (mg mL^{-1})
Acetone	531.0	0.09988	80	0.01	14.95804
DMF		0.18056	80	0.01	27.04069
DMSO		1.04907	1	0.001	19.6386
Ethanol		0.15921	1	0.01	0.29804
THF		0.17264	80	0.01	25.85459

PBI-Y (33)

Solvent	λ_{max} (nm)	λ_{max} Absorbance (au)	Dilution Factor (au)	Path Length (cm)	Concentration (mg mL^{-1})
Acetone	530.5	0.19109	10	0.01	2.63491
DMF		0.21629	80	0.01	23.85911
DMSO		0.19356	80	0.01	21.35175
Ethanol		0.15814	80	0.01	17.44454
THF		0.20435	80	0.01	22.542

ThNap-FF (34)

Solvent	λ_{max} (nm)	λ_{max} Absorbance (au)	Dilution Factor (au)	Path Length (cm)	Concentration (mg mL^{-1})
Acetone	269.5	0.02136	1	0.001	9.30619
DMF		0.0255	4	0.001	44.43969
DMSO		0.01781	4	0.001	31.03807
Ethanol		0.02013	1	0.001	8.7703
THF		0.05312	1	0.001	23.14349

2Nap-VL (35)

Solvent	λ_{max} (nm)	λ_{max} Absorbance (au)	Dilution Factor (au)	Path Length (cm)	Concentration (mg mL^{-1})
Acetone	273.0	0.10434	1	0.001	9.20582
DMF		0.08019	4	0.001	28.30034
DMSO		0.10946	4	0.001	38.6302
Ethanol		0.18429	1	0.001	16.25973
THF		0.09733	4	0.001	34.34933

This journal is © The Royal Society of Chemistry 20xx

2Nap-FL (36)

Solvent	λ_{max} (nm)	λ_{max} Absorbance (au)	Dilution Factor (au)	Path Length (cm)	Concentration (mg mL^{-1})
Acetone	273.5	0.21779	1	0.001	21.72967
DMF		0.07556	4	0.001	30.15555
DMSO		0.06666	4	0.001	26.60361
Ethanol		0.16235	1	0.001	16.19823
THF		0.09253	4	0.001	36.92817

2Nap-FI (37)

Solvent	λ_{max} (nm)	λ_{max} Absorbance (au)	Dilution Factor (au)	Path Length (cm)	Concentration (mg mL^{-1})
Acetone	273.5	0.2131	1	0.001	20.19865
DMF		0.3806	1	0.001	36.07511
DMSO		0.38721	1	0.001	36.70164
Ethanol		0.14058	1	0.001	13.32485
THF		0.35427	1	0.001	33.57942

2Nap-VF (38)

Solvent	λ_{max} (nm)	λ_{max} Absorbance (au)	Dilution Factor (au)	Path Length (cm)	Concentration (mg mL^{-1})
Acetone	273.5	0.07893	1	0.001	7.40466
DMF		0.25567	1	0.001	23.98517
DMSO		0.09589	4	0.001	35.98292
Ethanol		0.13061	1	0.001	12.25292
THF		0.04772	4	0.001	17.90703

2Nap-IV (39)

Solvent	λ_{max} (nm)	λ_{max} Absorbance (au)	Dilution Factor (au)	Path Length (cm)	Concentration (mg mL^{-1})
Acetone	273.0	0.1066	1	0.01	0.95557
DMF		0.32592	1	0.001	29.2158
DMSO		0.15794	1	0.001	14.1579
Ethanol		0.09659	1	0.01	0.86584
THF		0.42525	1	0.001	38.11984

CF_3Ph-FL (40)

Solvent	λ_{max} (nm)	λ_{max} Absorbance (au)	Dilution Factor (au)	Path Length (cm)	Concentration (mg mL^{-1})
Acetone	269.0	0.07979	1	0.001	34.63189
DMF		0.05514	1	0.001	23.93285
DMSO		0.08057	1	0.001	34.97044
Ethanol		0.07357	1	0.001	31.93217
THF		0.07744	1	0.001	33.6119

CF_3Ph-FV (41)

Solvent	λ_{max} (nm)	λ_{max} Absorbance (au)	Dilution Factor (au)	Path Length (cm)	Concentration (mg mL^{-1})
Acetone	268.5	0.02859	1	0.001	13.81978
DMF		0.05605	1	0.001	27.09335
DMSO		0.05435	1	0.001	26.27161
Ethanol		0.06319	1	0.001	30.54467
THF		0.03359	1	0.001	16.23668

2Nap-VI (42)

Solvent	λ_{max} (nm)	λ_{max} Absorbance (au)	Dilution Factor (au)	Path Length (cm)	Concentration (mg mL^{-1})
Acetone	273.5	0.12725	1	0.001	10.97942
DMF		0.0757	4	0.001	26.12627
DMSO		0.05366	4	0.001	18.51963
Ethanol		0.20396	1	0.001	17.59813
THF		0.10679	4	0.001	36.85633

C_6-NDI-A (43)

Solvent	λ_{max} (nm)	λ_{max} Absorbance (au)	Dilution Factor (au)	Path Length (cm)	Concentration (mg mL^{-1})
Acetone	381.5	0.54923	1	0.001	11.98619
DMF		1.29405	1	0.001	28.24087
DMSO		1.49149	1	0.001	32.54973
Ethanol		0.33045	1	0.001	7.21162
THF		1.00809	1	0.001	22.00018

C_6-NDI-F (44)

Solvent	λ_{max} (nm)	λ_{max} Absorbance (au)	Dilution Factor (au)	Path Length (cm)	Concentration (mg mL^{-1})
Acetone	381.0	0.65783	1	0.001	16.01184
DMF		1.15244	1	0.001	28.05085
DMSO		1.01673	1	0.001	24.74761
Ethanol		1.46768	1	0.001	35.72391
THF		0.60874	1	0.001	14.81697

C_6-NDI-L (45)

Solvent	λ_{max} (nm)	λ_{max} Absorbance (au)	Dilution Factor (au)	Path Length (cm)	Concentration (mg mL^{-1})
Acetone	381.5	1.47885	1	0.001	33.17023
DMF		1.01199	1	0.001	22.69868
DMSO		1.74551	1	0.001	39.15134
Ethanol		1.52155	1	0.001	34.12798
THF		1.71097	1	0.001	38.37662

This journal is © The Royal Society of Chemistry 20xx
Please do not adjust margins

Wavelength (nm)

NDI-GA (46)					
**Solvent**	**λ_{max} (nm)**	**λ_{max} Absorbance (au)**	**Dilution Factor (au)**	**Path Length (cm)**	**Concentration (mg mL^{-1})**
Acetone	379.5	0.09086	2	0.01	0.4547
DMF		-	-	-	-
DMSO		0.08906	40	0.01	8.9139
Ethanol		-	-	-	-
THF		-	-	-	-

Wavelength (nm)

NDI-GL (47)					
**Solvent**	**λ_{max} (nm)**	**λ_{max} Absorbance (au)**	**Dilution Factor (au)**	**Path Length (cm)**	**Concentration (mg mL^{-1})**
Acetone	379.5	0.13151	5	0.01	1.91806
DMF		0.71605	1	0.001	20.88708
DMSO		1.86981	1	0.001	54.5421
Ethanol		0.09127	1	0.001	2.66233
THF		0.6518	1	0.001	19.01292

Wavelength (nm)

NDI-LA (48)					
**Solvent**	**λ_{max} (nm)**	**λ_{max} Absorbance (au)**	**Dilution Factor (au)**	**Path Length (cm)**	**Concentration (mg mL^{-1})**
Acetone	382.5	0.05477	200	0.01	35.37933
DMF		0.22527	4	0.001	29.10317
DMSO		0.22527	4	0.001	29.10317
Ethanol		0.30539	4	0.001	39.45406
THF		0.44931	4	0.001	58.04742

Wavelength (nm)

NDI-YI (49)					
**Solvent**	**λ_{max} (nm)**	**λ_{max} Absorbance (au)**	**Dilution Factor (au)**	**Path Length (cm)**	**Concentration (mg mL^{-1})**
Acetone	381.5	0.51179	2	0.01	4.05603
DMF		0.23526	4	0.001	37.28953
DMSO		0.9567	1	0.001	37.91007
Ethanol		0.13995	1	0.001	5.54564
THF		0.91158	1	0.001	36.12216

Wavelength (nm)

PBI-A 1-Py (50)					
**Solvent**	**λ_{max} (nm)**	**λ_{max} Absorbance (au)**	**Dilution Factor (au)**	**Path Length (cm)**	**Concentration (mg mL^{-1})**
Acetone	697.5	0.08082	8	0.01	2.22769
DMF		0.69011	1	0.001	23.77741
DMSO		0.11427	40	0.01	15.74847
Ethanol		0.12081	1	0.001	4.16245
THF		0.51869	1	0.001	17.87121

Please do not adjust margins
Please do not adjust margins

PBI-I 1-Py (51)

Solvent	λ_{max} (nm)	λ_{max} Absorbance (au)	Dilution Factor (au)	Path Length (cm)	Concentration (mg mL^{-1})
Acetone	700.0	0.4605	1	0.001	15.07957
DMF		0.65633	1	0.001	21.49223
DMSO		0.79951	1	0.001	26.18081
Ethanol		0.51332	1	0.001	16.80921
THF		0.80684	1	0.001	26.42084

PBI-L 1-Py (52)

Solvent	λ_{max} (nm)	λ_{max} Absorbance (au)	Dilution Factor (au)	Path Length (cm)	Concentration (mg mL^{-1})
Acetone	695.5	0.06549	100	0.01	19.98176
DMF		0.27796	4	0.001	33.92354
DMSO		0.2166	40	0.01	26.43487
Ethanol		0.25273	1	0.001	7.71109
THF		0.14479	4	0.001	17.67085

PBI-V 1Py (53)

Solvent	λ_{max} (nm)	λ_{max} Absorbance (au)	Dilution Factor (au)	Path Length (cm)	Concentration (mg mL^{-1})
Acetone	697.5	0.35716	1	0.001	12.10153
DMF		0.35237	1	0.001	7.24038
DMSO		0.34067	1	0.001	7.61614
Ethanol		0.03168	1	0.001	0.91598
THF		0.13972	1	0.001	4.44408

1Nap-VD (54)

Solvent	λ_{max} (nm)	λ_{max} Absorbance (au)	Dilution Factor (au)	Path Length (cm)	Concentration (mg mL^{-1})
Acetone	294.0	0.22655	1	0.001	14.43424
DMF		0.54808	1	0.001	34.91997
DMSO		0.58404	1	0.001	37.2111
Ethanol		0.54934	1	0.001	35.00025
THF		0.51001	1	0.001	32.49441

NDI-4-Nitro-Phenylalanine (55)

Solvent	λ_{max} (nm)	λ_{max} Absorbance (au)	Dilution Factor (au)	Path Length (cm)	Concentration (mg mL^{-1})
Acetone	381.0	0.30184	1	0.001	7.9909
DMF		0.32096	4	0.001	33.98832
DMSO		0.35334	4	0.001	37.41723
Ethanol		0.06964	1	0.001	1.84365
THF		0.49769	1	0.001	13.17582

This journal is © The Royal Society of Chemistry 20xx

Solubility Data Summary

Table S3 Solubility classification of training, test, and validation compounds used for model development. Compounds with a solubility greater than or equal to 10 $mgmL^{-1}$ are deemed to be soluble (S), while those with a solubility less than 10 $mgmL^{-1}$ are considered insoluble (I).

Compound	Acetone	DMF	DMSO	Ethanol	THF
**1**	I	S	S	S [b]	S
**2**	S	S	S	S [b]	S
**3**	I [b]	S	S	S	S
**4**	S	S	S	S	S
**5**	S	S	S	I	S
**6**	S	S	S	S	S
**7**	I	S	S	I	S
**8**	I	S	S	[a]	I
**9**	I	S	S	I	S
**10**	S [b]	S	S	I [b]	S
**11**	I [b]	S	S	I	I
**12**	I	S	S	I	S [b]
**13**	I	S	S	I	S
**14**	S	S	S	S	S
**15**	S	S	S	S	S
**16**	S	S	S	S	S
**17**	S	S	S	S	S
**18**	S	S	S	I	S
**19**	S	S	S	S	S
**20**	I	I	S [b]	I	I
**21**	I	I	S	I	I
**22**	S	S	S	S	S
**23**	I	S	S	I	I
**24**	I	S [b]	S	I	I
**25**	I	I	I	I	I
**26**	I	I	S [b]	I	I
**27**	I	I	I	I	I
**28**	I	S	S	I	S
**29**	I	I	I	I	I
**30**	I	S [b]	S	I	I
**31**	I	I [b]	S [b]	I	I
**32**	S	S	S	I	S
**33**	I	S	S	S	S
**34**	I [b]	S	S	I [b]	S
**35**	I [b]	S	S	S	S
**36**	S	S	S	S	S
**37**	S	S	S	S	S
**38**	I	S	S	S [b]	S
**39**	I	S	S	I	S
**40**	S	S	S	S	S
**41**	S	S	S	S	S
**42**	S [b]	S	S	S	S
**43**	S [b]	S	S	I	S
**44**	S	S	S	S	S
**45**	S	S	S	S	S
**46**	I	[a]	I [b]	[a]	[a]
**47**	I	S	S	I	S
**48**	S	S	S	S	S
**49**	I	S	S	I	S
**50**	I	S	S	I	S
**51**	S	S	S	S	S
**52**	S	S	S	I [b]	S
**53**	S	I	I [b]	I	I
**54**	S	S	S	S	S
**55**	I [b]	S	S	I	S

a Insufficient material for solubility measurement. Compound is not included in the model training or testing for this solvent.

b Solubility measurement lies between 7.5 and 12.5 $mgmL^{-1}$, close to the chosen class threshold of 10 $mgmL^{-1}$.

This journal is © The Royal Society of Chemistry 20xx

Please do not adjust margins

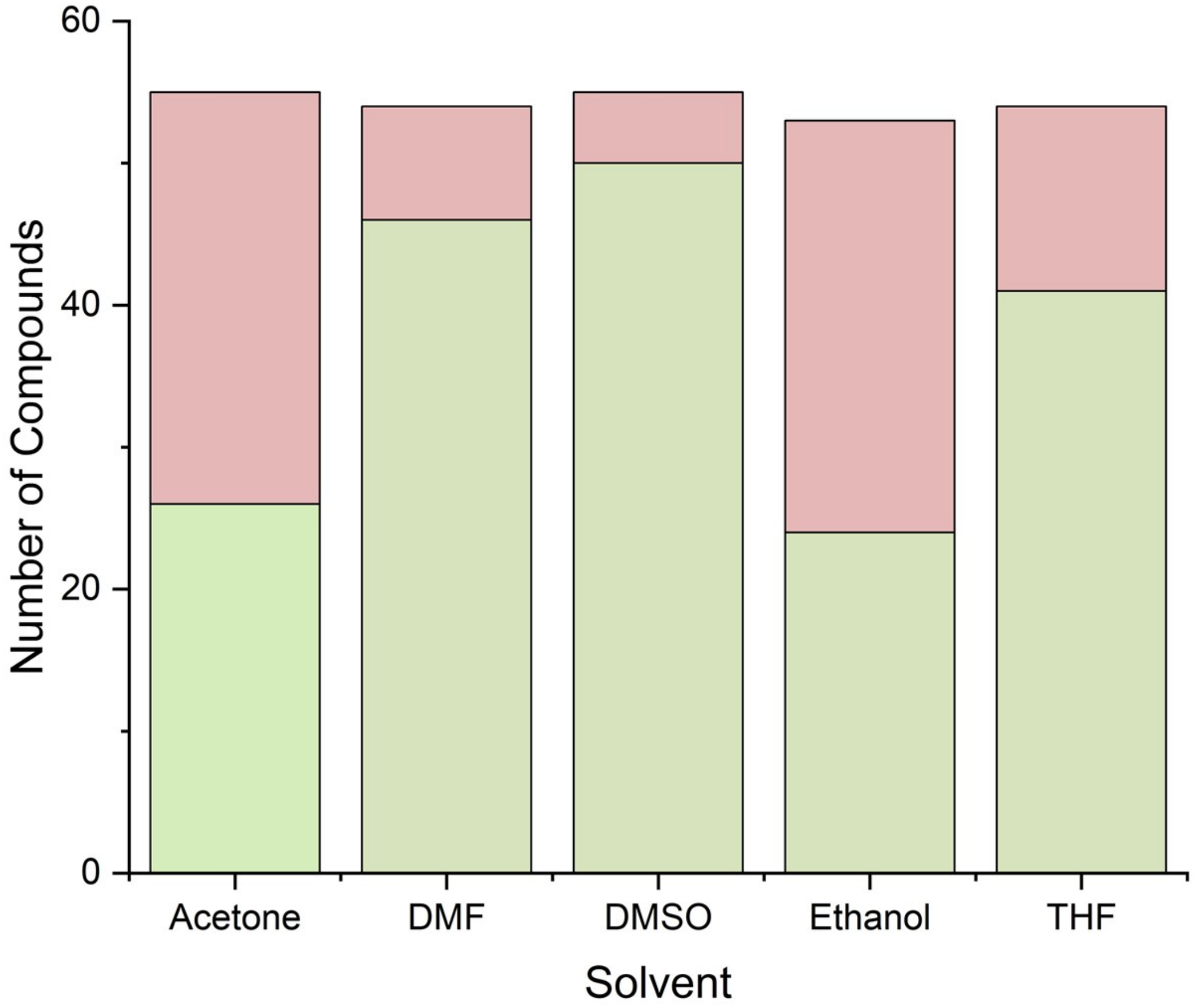

Figure S2 Class distribution of compounds 1 – 52 which comprise the training and test sets across five solvents. The green segment represents the portion of compounds deemed soluble by the above classification procedure, while red represents those deemed insoluble.

This journal is © The Royal Society of Chemistry 20xx

Please do not adjust margins

Please do not adjust margins

Section 3

QSPR

In order to build a QSPR model capable of predicting a property from the chemical structure the model must first interpret the structural features of the compound. Therefore each molecule was generated *in silico* using ChemDraw and converted to SMILES format.[21, 22] From the SMILES representation, molecular descriptors were calculated in KNIME (Version 5.11.0).[23]

Zero-dimensional physicochemical descriptors were calculated to show that no simple model between such properties and solubility could be easily identified (Figure S4 - Figure S8). The physicochemical descriptors SlogP,[24] atomic surface area,[25] atomic polar surface area,[26] rotatable bonds, hydrogen bond donors, hydrogen bond acceptors, number of atoms, and number of rings were calculated. From the histograms of the physicochemical descriptors, there is significant overlap between the soluble and insoluble property distributions, making it difficult to identify which properties have the biggest impact on organic solubility. Therefore, more complex two-dimensional molecular fingerprint descriptors were calculated to better represent each molecule. Molecular fingerprints are descriptors which can encode the structural characteristics of a compound as a vector and have previously been utilised for chemical similarity comparisons and QSPRs.[27] Molecular fingerprints such as the Morgan fingerprint quantify extended connectivity by iteratively describing larger substructures around each atom to capture information about direction connections between atoms as well as the larger structures encoded as an integer. The integer is then converted into a binary integer fingerprint which can be computationally processed. The resulting fingerprint encodes the presence or absence of each unique substructure within the molecule, up to a specified radius.[28] Morgan fingerprints were converted to a list of integer bits 1024 bits long.

The bit integer Morgan fingerprints were used to calculate molecular similarity for the purposes of calculating the modelability index (MODI) and applicability domains.[29, 30] The Manhattan distance was used as a chemical similarity metric as it has been previously determined to be the better metric for calculating chemical similarity in high dimensional space.[31] The Manhattan distance (D) of two fingerprints (**p** and **q**) is calculated by summing the absolute difference between each pair of corresponding coordinates (i) (Equation S2).

$$D(p,q) = \sum_{i=1}^{n} |p_i - q_i| \qquad \text{(Equation S2)}$$

By calculating the Manhattan distance between each pair morgan fingerprints, and determining the lowest distance combination for each compound, the most chemically similar compound could be identified for each training set compound. The number of times the classification (c = 2 for binary classification) of the i-th activity class is the same as nearest neighbour (N_i^{same}) is compare to the number of compounds in that same class (N_i^{total}). The average ratio of these across each class describes the MODI (Equation S3).

$$MODI = \frac{1}{c}\sum_{i=1}^{c} \frac{N_i^{same}}{N_i^{total}} \qquad \text{(Equation S3)}$$

For each class, 20% of molecules are removed from the dataset for independent testing after modelling. After removing the testing partition, the remaining data is used to train the model and define the domain of applicability. While some of the accepted models achieved MODI scores below the indicative threshold, limiting the accepted partitions to those with MODI scores above 0.65 did not yield improved models, showing this metric is just an indication and not predictive of the final performance. Each of the training data fingerprints is used to determine distance based boundaries as described by Sahigara *et al.*[30] Each individual bit is averaged across the training compounds to determine the fingerprint centroid. From this point a number if boundaries are calculated: maxdist, the maximum distance between the centroid and the training fingerprints; 2d and 3d, given by multiplying the average distance (d) between the centroid and the training fingerprints by 2 and 3, respectively; dsz, given by multiplying the average distance (d) and standard deviation (s) between the centroid and the training fingerprints and some constant (z, we have chosen 0.5 as is the typical constant); and p95, the boundary which considers 95% of the training compounds as in-domain. The distance between the training centroid and fingerprints of the test and validation compounds can be calculated and compared against the

This journal is © The Royal Society of Chemistry 20xx

Please do not adjust margins

thresholds to determine if they have sufficient chemical similarity to afford reliable predictions. As discussed in the main text, the dsz applicability domain was chosen as the appropriate boundary.

To maximize the quality of the training data, it is important to screen and remove descriptors of low informational content. These include those with low variance (where the descriptors have near-constant values across samples), and high-correlation (where values across the descriptors match). The column with the most correlated columns is carried forward and all correlated columns are filtered out. This procedure is repeated until no more columns can be identified. Here we applied a low variance threshold of 0.1 and a high-correlation threshold of $r^2 = 0.9$ using Spearman's rho rank correlation to reduce the number of bit fingerprints descriptors from 1024 to between 50 and 56 descriptors of higher informational content.[32]

Following the filtering of information-poor descriptor bits, histograms of each descriptor were investigated for each model. Just as with the physicochemical descriptors, there was significant overlap between the soluble and insoluble property distributions, making it difficult to identify which structural bits have the biggest impact on organic solubility (Figure S9 – Figure S13). With no clear relationship apparent, a more complex machine learning relationship is required to elucidate how the molecular structure governs the solubility across the solvents. Models were initially built using the filtered Morgan fingerprints descriptors only. Due to the poor performance of the acetone and ethanol models a new set of models were built using both the Morgan fingerprints and the physicochemical descriptors. The inclusion of the additional descriptors yielded an improved ethanol solubility model, but did not improve the metrics of the other models. Therefore, we report the metrics for the fingerprint only model for acetone, DMF, DMSO, and THF solubility; and the combined fingerprint / physicochemical descriptors model for ethanol solubility.

Utilising highly imbalanced data classes in machine learning model can mean that the minority class is poorly modelled because it is underrepresented in the training data.[33] To overcome this class balancing techniques can be employed to ensure both classes are well represented and minimise the bias towards the majority class during the machine learning. Synthetic minority oversampling technique was employed over simpler undersampling techniques due to the small size of the dataset.[34] Using the average of a randomly selected data point and its nearest neighbours from the minority class, a new synthetic data point upsamples the minority class. The new synthetic samples are very similar to other samples in the data set which allows the minority class to be augmented without requiring further expanding the dataset.

For each machine learning algorithms to predict successfully predict the properties of interest, their setup must be tuned to establish effective relationships. Hyperparameters are the variables of each machine learning model which can be modified to control the training process of a machine learning models. In order to build robust predictions, we must find the hyperparameters that afford the most effective model. To do this we employed cross-validation, an internal validation technique wherein the predictive power of a model for a given set of hyperparameters can be estimated. The training set is partitioned into a number of folds (in this work we used 5-fold cross validation), one of which is removed from the training procedure, the model is built with the remaining data, and predictions are made for those compounds not part of the model training. This is repeated for each fold of data, the predictions aggregated, and the predictive performance can be estimated using these predictions. We chose Matthew's correlation co-efficient measure as our metric as it has recently been shown that the most popular measure of classification models, receiver operating characteristic's (ROC) area under the curve (AUC), should be replaced by MCC as the standard metric for assessing binary classification.[35] The MCC considers all values of the binary classification confusion matrix (TP, true positive; TN, true negative; FP, false positive; and FN, false negative) to provide a balanced and reliable assessment, especially for imbalanced datasets as defined by (Equation S4. An ideal model has a MCC measure value of 1, with a random model taking a value of 0 and an acceptable value being >0.5.

$$MCC = \frac{TP \cdot TN - FP \cdot FN}{\sqrt{(TP + FP) \cdot (TP + FN) \cdot (TN + FP) \cdot (TN + FN)}} \qquad \text{(Equation S4)}$$

The goal of the cross-validation procedure is to find the combination of hyperparameters which maximises this scoring metric, which should lead to the most predictive model. While there are a number of strategies the search the hyperparameter combinations, Bayesian optimisation being one of the most popular,[36] we employed a simple brute force search of each hyperparameter combination as defined by a start, stop, and step value for each of the most important model parameters (Table S4). While internal assessment allows us to estimate the model's performance, the true predictive power of a model can only be assessed by applying the it to previously unseen data.

After cross-validation, the highest performing hyperparameter combination is taken for each model, and a final model is built using the entirety of the training data. Using the 20% test partition taken before model training, the performance of the model on unseen

Please do not adjust margins

data can be assessed. Across the different solvents, the class balance of the data varies significantly. Therefore, it is important that the metrics used are independent of the class distribution. In situations where a model could achieve high overall accuracy simply by favouring the dominant class; balanced accuracy, MCC, and Cohen's kappa (K) ensure that the performance on each class is equally weighted, making it fairer and more informative when classes are skewed. Balanced accuracy is a measure of the proportion of correctly classified molecules in each class (c = 2 for binary classification) (Equation S5). It can vary between 0 and 1 with an ideal model having a value of 1 and an acceptable value being >0.7.[2]

$$Balanced\ Accuracy = \frac{1}{c}\sum_{i=1}^{c}\frac{TP_i}{TP_i + TN_i} \quad \text{(Equation S5)}$$

Cohen's Kappa is a statistical measure that assesses the agreement between two raters, in this case measured classifications compared to their predicted classifications. It can vary between -1 and 1 with an ideal model having a value of 1 and an acceptable value being > 0.4 (Equation S6).[2]

$$Cohen's\ Kappa = \frac{2 \cdot (TP \cdot TN - FP \cdot FN)}{(TP + FP) \cdot (FP + TN) + (TP + FN) \cdot (FN + TN)} \quad \text{(Equation S6)}$$

Using the thresholds described above, the quality of the models can be classified. The performance of the models on the training set and in-domain test sets are reported (Table S5 – Table S7). The confusion matrices for predictions across each model are also provided (Table S8 - Table S12) Typically, the maxdist was equivalent to the 2d, 3d, and p95 in performance metrics, therefore, as one of the more generous boundaries, the metrics obtained after applying the maxdist domain was compared with the much more pessimistic dsz boundary.

In addition to balanced accuracy, MCC, and K whose thresholds were used to define the performance of models, the receiver operating characteristic curves (ROC) were generated for each model. The graph plots the true positive rate against the false positive rate for every possible classification threshold (The cutoff probability used to decide whether a prediction should be classified as one class or another). It acts as an easy to visualise comparison of model performance which can quantified by calculating the area under the curve (AUC). However, as previously discussed, it has been shown that MCC should replace ROC AUC as the metric for assessing classification model performance should be avoided so was not used to invalidate models, but to help compare their ability (Figure S14).

In any data set, there will be a varying dependence of model performance on dataset composition. The negative effects of training and test set portioning can be significant, especially when modelling with small datasets. Therefore, it was expected that the changing partition of the training data would heavily impact model performance. Therefore, for each solvent model, 10 randomised partitions were investigated. The split was stratified to ensure that solubility classifications in the training set were representative of the entire dataset. The model with the best metrics and fewest out-of-domain compounds was carried forward for further investigation. The selected-partition metrics are a best case and should not be interpreted as robust estimates of generalisation. However, the significant change in predictive quality as the partitions are randomised highlights a significant limitation of the current dataset. Table S13 shows there is a strong dataset dependence on the prediction ability, which could indicate that the limited dataset employed here could result in poorer generalisation on unseen data in the final models. The model performance across each of the randomly stratified datasets is reported in Table S14 - Table S16

To further improve our confidence of the optimised models, a randomisation test was conducted. The MCC values for training, test, and in-domain test set predictions were compared to those derived for random datasets on the optimised model, generated by random shuffling of compound solubility classifications. The statistical significance of QSAR models for each set was evaluated by determining the mean value of MCC (μ) and its standard deviation (σ) for 100 random datasets applied to the optimised models. The Z-score is calculated by comparing the randomised average to the MCC value for the actual dataset (*h*) using (Equation S7. The Z-score can then be compared with the critical values of Z_c at different levels of significance (α) (Table S17 - Table S18).[37] Similarly, MCC values for the test and in-domain test set predictions were compared to those calculated by undertaking the same cross-validation hyperparameter modelling on a randomised training set, and making predictions with the new scrambled models. The statistical significance of QSAR models for each set was evaluated by determining the mean value of MCC (μ) and its standard deviation (σ) for 10 random datasets applied to the optimised randomised models. The Z-score is calculated by comparing the randomised average to the MCC value finor the actual dataset (*h*) using (Equation S7 and Table S19).

This journal is © The Royal Society of Chemistry 20xx

Please do not adjust margins

Please do not adjust margins

$$Z = \frac{h - \mu}{\sigma} \quad \text{(Equation S7)}$$

To further show that the prediction performance of the optimised models outperformed randomised models, a one-tailed one-sample t-test was performed comparing the MCC values for the optimised models with those obtained from the cross-validation hyperparameter modelling on randomised training sets (Table S20). The t-test shows that the improved performance of each model was at least statistically significant, with the highest p-values corresponding to the poorest Z-scoring models.

A key feature of a high-quality model is the inclusion of a mechanistic interpretation of the model, if possible. There are many ways to probe the features underpinning machine learning models. Some, like the information gain metric provided by KNIME's integration of the XGBoost model provide a way to assess the contribution of each descriptor to the model, though relying on a single model may introduce significant bias to the interpretation if the model is particularly biased to include a certain substructural descriptor. While metrics such as Shapley values are a promising method for obtaining even deeper insight, allowing the descriptors impact on the outcome prediction to be elucidated, it was shown to provide limited insights into our models (Figure S15).[38] Shapley values can be highly unstable on randomly drawn datasets, the impact of which is amplified due to the limited quantity of training data.[39] Instead, previous models have used many shallow decision trees to generate metrics of importance.[40] By quantifying how often each descriptor is selected as the optimal variable to partition the data into their appropriate classes, each descriptors importance can be determined. If a descriptor is often selected as the be best split across several trees, it is likely that it is contributing more to the model than those that do not. This can be quantified as the sum of ratios between the frequency of being selected as the optimum partition criteria and how often it was randomly selected to be a candidate across each level of the decision tree (Equation S8).

$$Importance = \frac{\#\, Split_0}{\#\, Candidate_0} + \frac{\#\, Split_1}{\#\, Candidate_1} + \frac{\#\, Split_2}{\#\, Candidate_2} \quad \text{(Equation S8)}$$

While quantifying the descriptors of importance is key to identifying the important structural features governing the properties of interest, identifying the abstract fingerprint bits which contribute to the model does not provide useful information for instructing future chemical design. Instead, the chemical substructure represented by each fingerprint bit of interest should be reverse engineered to elucidate the chemical substructures that influence the desired property. To better visualise the importance of the descriptors we used a previously designed KNIME workflow which combined functionalities in Python[41] and KNIME[23, 41, 42] to highlight the relevant substructure associated with each bit (if present). We have reported the first structure of the 9 most important descriptors for each model (Figure S16 - Figure S20).

As would be expected, descriptors relating to the core structures are some of the most important structures, being one of the most dominant influences of solubility. In addition to this, the peptide link substructure is highlighted often indicating that changing the length of the peptide installed onto the core structure has a large effect on its propensity to dissolve. Lastly, the amino acid side chains are highlighted, particularly those that are highly aromatic, indicating that the presence or absence of aromatic amino acids has a greater effect on the solubility of the compound when compared to their aliphatic counterparts. The ethanol model which included the physicochemical descriptors shows the number of rotatable bonds, atomic surface area, and number of atoms as the most important descriptors, which may concur with the importance of the peptide link for the other models, indicating the molecular size to be one of the most important factors. Similarly, the number of rings is shown to be highly important, which may concur with the importance of the size of the aromatic core, as well as presence of the aromatic functionalities on governing solubility in ethanol.

Having built, optimised, and externally tested each model, the chosen model can be used to predict an independent molecule to validate its predictive ability. An *in silico* library of candidates can be applied to the model using the SMILES representation of each candidate to calculate the same descriptors used to train the model. While such libraries can be manually prepared, even a small number of functionalisation options can yield a large library of candidates. Manually preparing such a dataset inevitably results in errors; therefore, we prepared a script in Python[41] using RDKit[42, 43] to install side chain moieties onto core structures, enabling the automated generation of SMILES for each possible amino acid dipeptide candidate based on previously published code (Figure S3).[44]

This journal is © The Royal Society of Chemistry 20xx

Please do not adjust margins

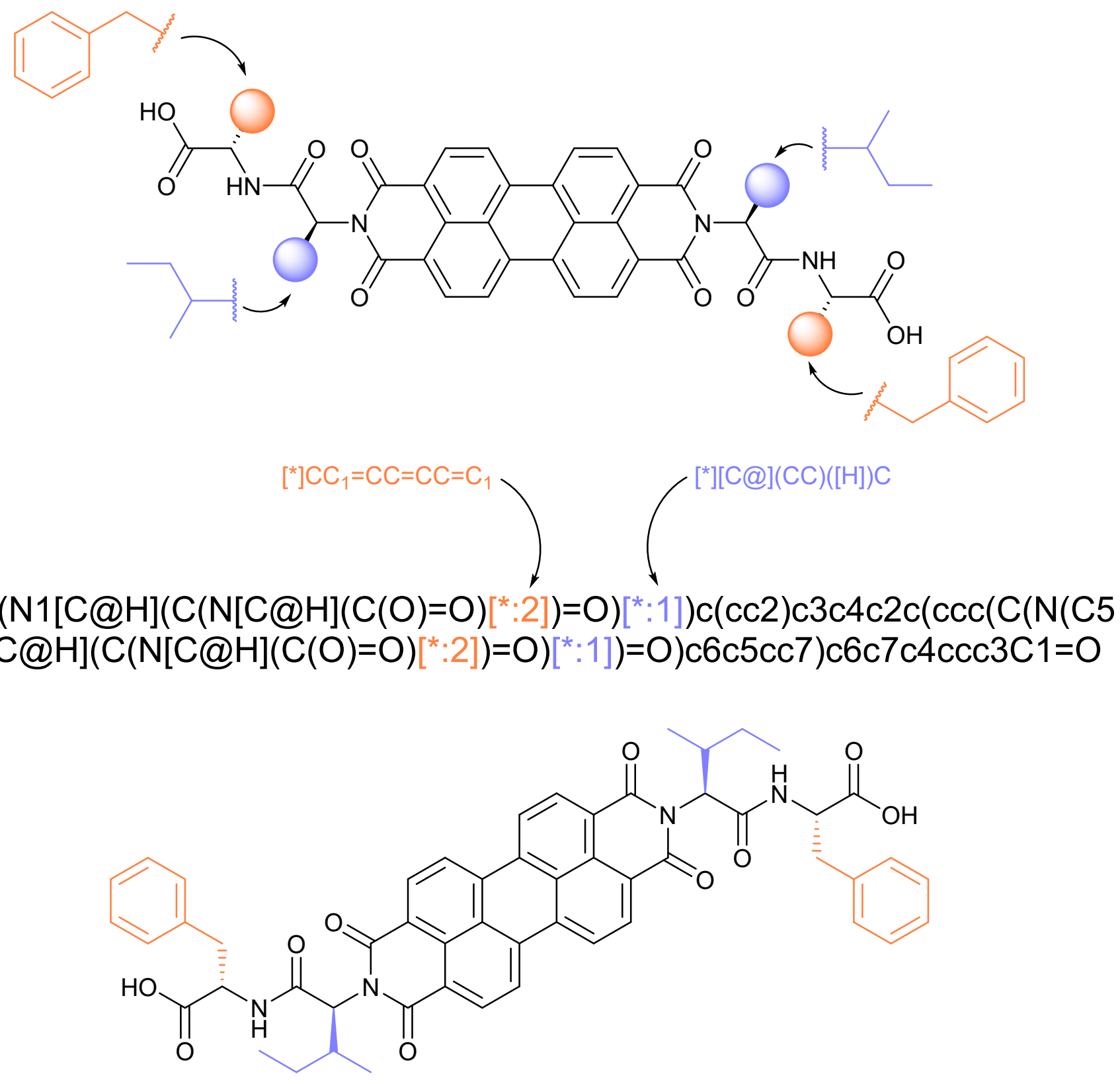

Figure S3. Simplified cartoon of automated smiles generation using SMILES of core structure and side chain substituents.

To provide further confidence in each prediction, each model provides a prediction probability which can be used to calculate prediction confidence and used to inform the likelihood that a prediction will be correct, as defined by the probability value for a given compound P_i (Equation S9). Predictions with a confidence greater than 40% are deemed to be of high confidence.[45]

$$Confidence\ for\ chemical\ i = \left(\frac{|P_i - 0.5|}{0.5}\right) \times 100 \qquad \text{(Equation S9)}$$

The validation predictions indicate that while predictive QSPR models have been found DMF, DMSO, and THF, further optimisation is required to improve the prediction of acetone and ethanol solubility for amino acid functionalised rylenes and naphthalenes (Table S21).

Please do not adjust margins

Supplementary Figures

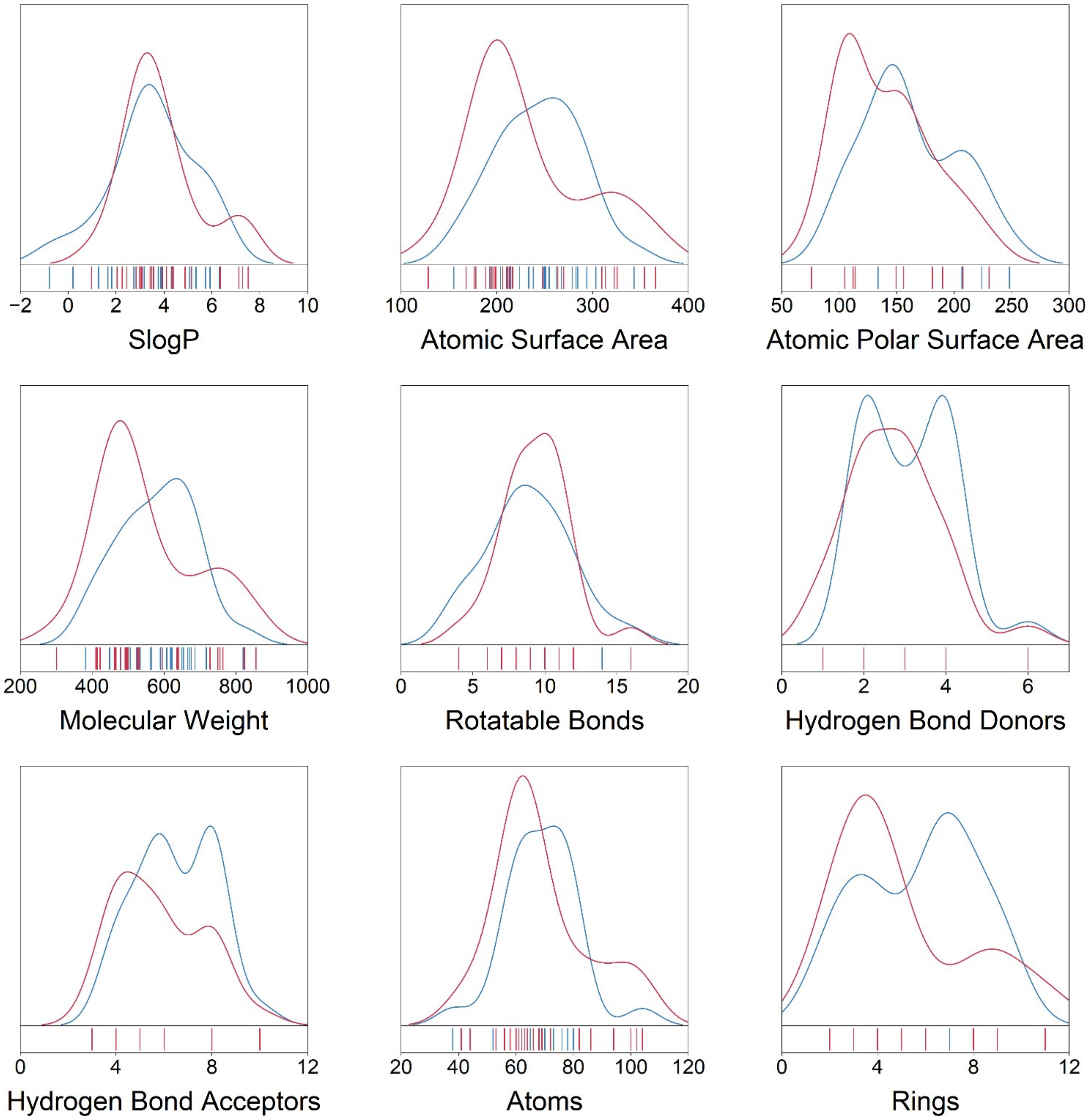

Figure S4. Plot of 9 physicochemical descriptors calculated and their distribution, separated by acetone solubility classification (soluble, red; insoluble, blue) for the training set. The value of each molecule's descriptor is depicted by | marker.

 This journal is © The Royal Society of Chemistry 20xx

Please do not adjust margins

Please do not adjust margins

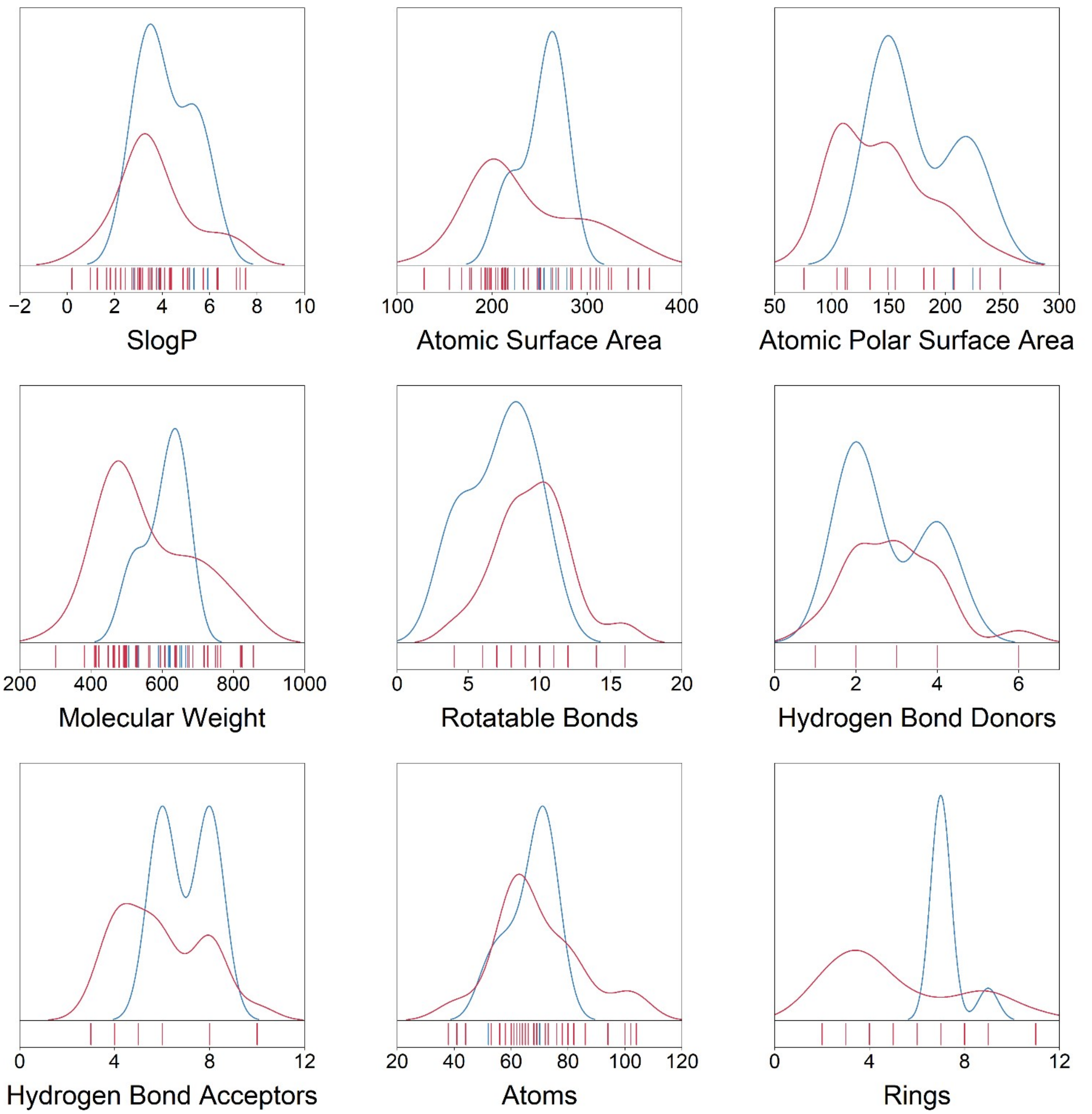

Figure S5. Plot of 9 physicochemical descriptors calculated and their distribution, separated by DMF solubility classification (soluble, red; insoluble, blue) for the training set. The value of each molecule's descriptor is depicted by | marker.

This journal is © The Royal Society of Chemistry 20xx

Please do not adjust margins

Please do not adjust margins

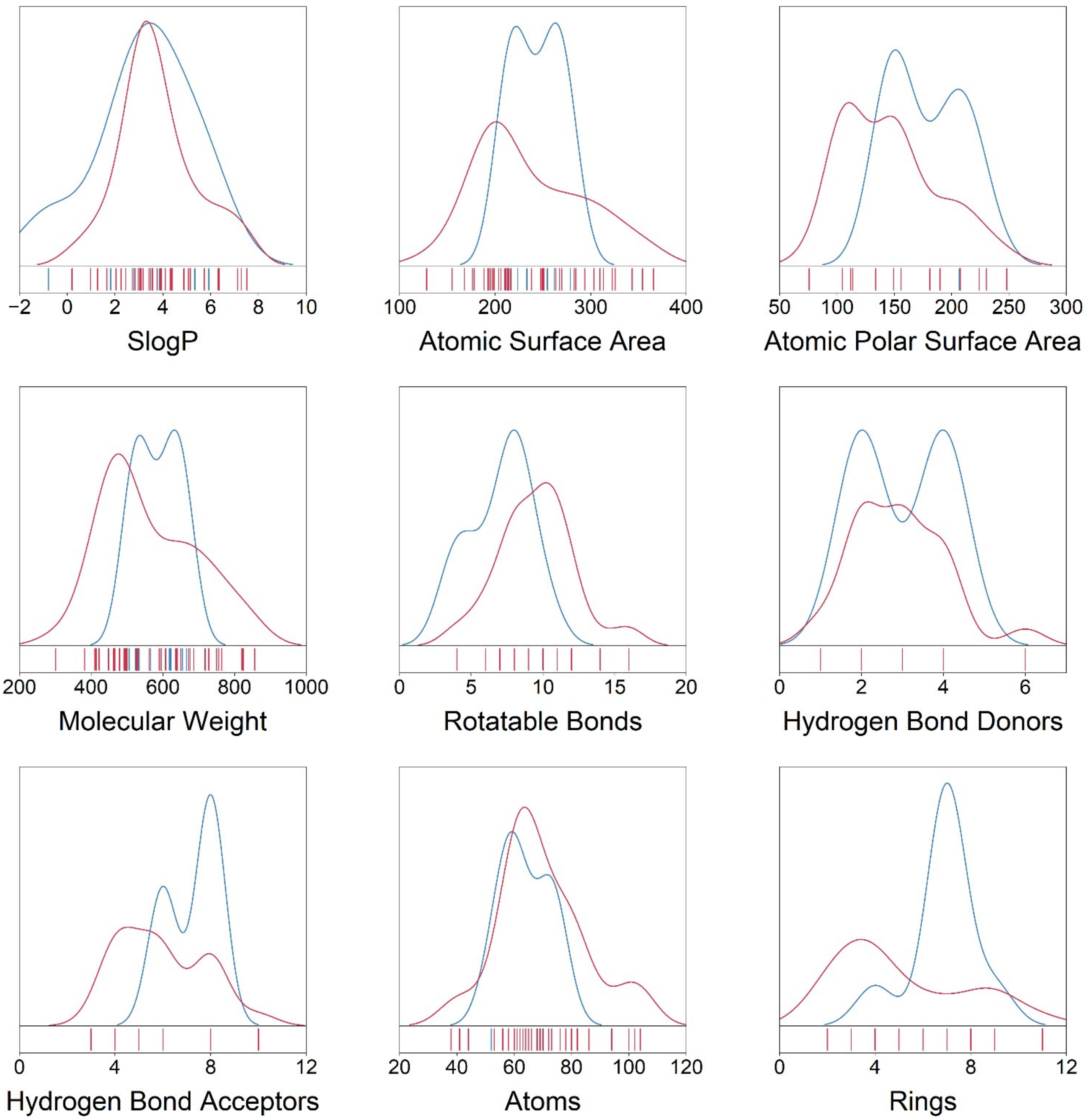

Figure S6. Plot of 9 physicochemical descriptors calculated and their distribution, separated by DMSO solubility classification (soluble, red; insoluble, blue) for the training set. The value of each molecule's descriptor is depicted by | marker.

This journal is © The Royal Society of Chemistry 20xx

Please do not adjust margins

Please do not adjust margins

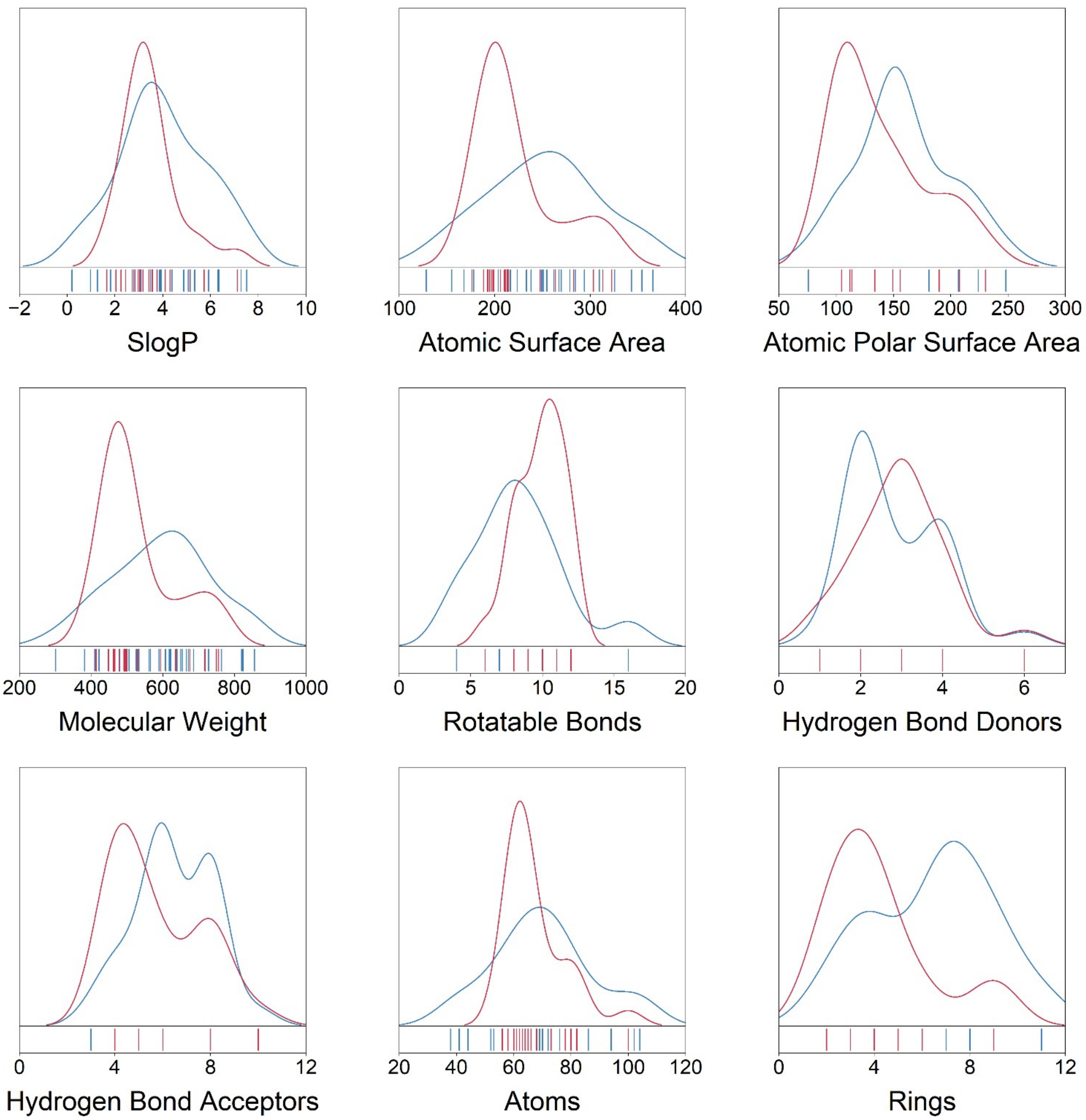

Figure S7. Plot of 9 physicochemical descriptors calculated and their distribution, separated by ethanol solubility classification (soluble, red; insoluble, blue) for the training set. The value of each molecule's descriptor is depicted by | marker.

This journal is © The Royal Society of Chemistry 20xx

Please do not adjust margins

Please do not adjust margins

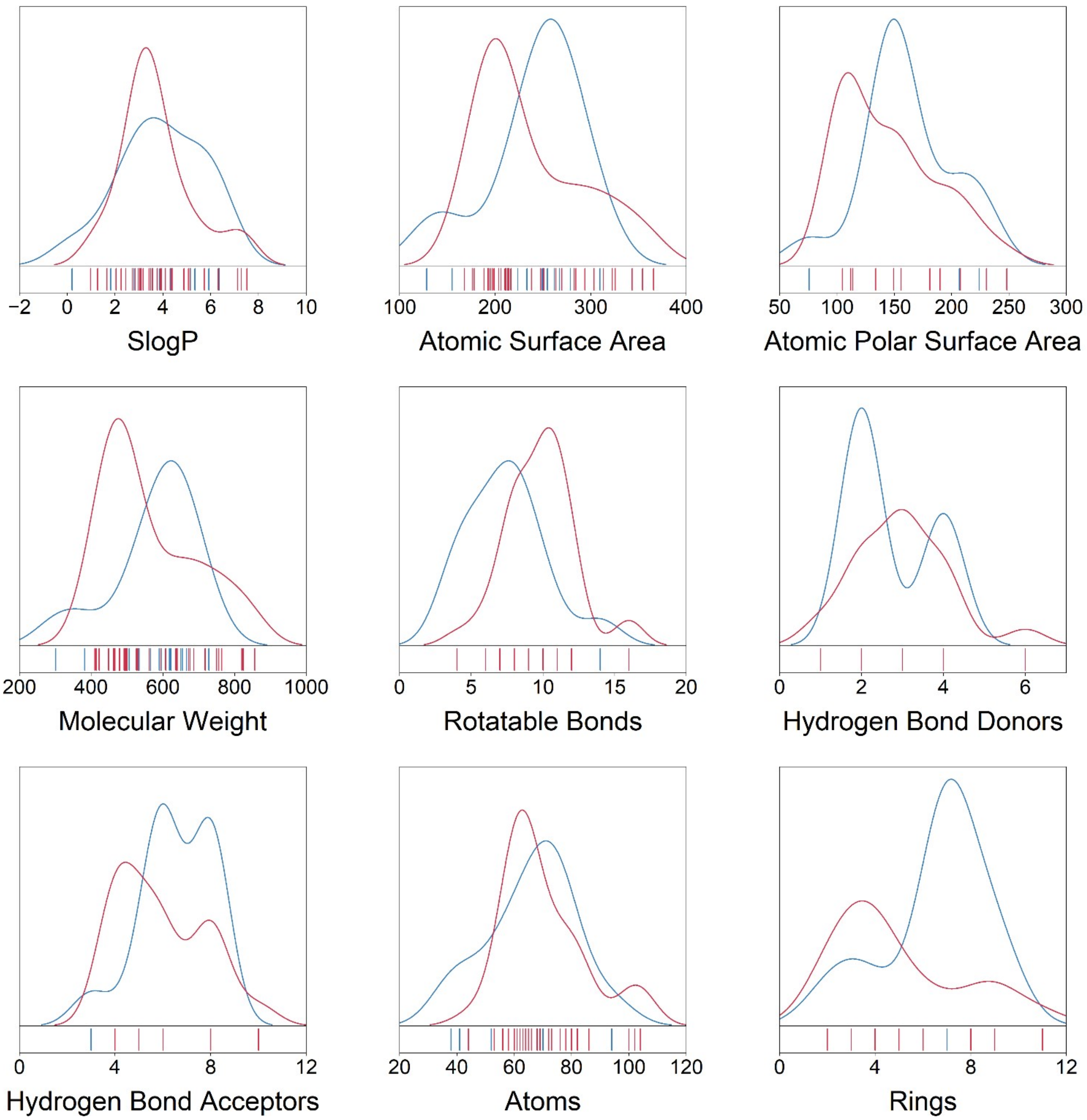

Figure S8. Plot of 9 physicochemical descriptors calculated and their distribution, separated by THF solubility classification (soluble, red; insoluble, blue) for the training set. The value of each molecule's descriptor is depicted by | marker.

 This journal is © The Royal Society of Chemistry 20xx

Please do not adjust margins

Please do not adjust margins

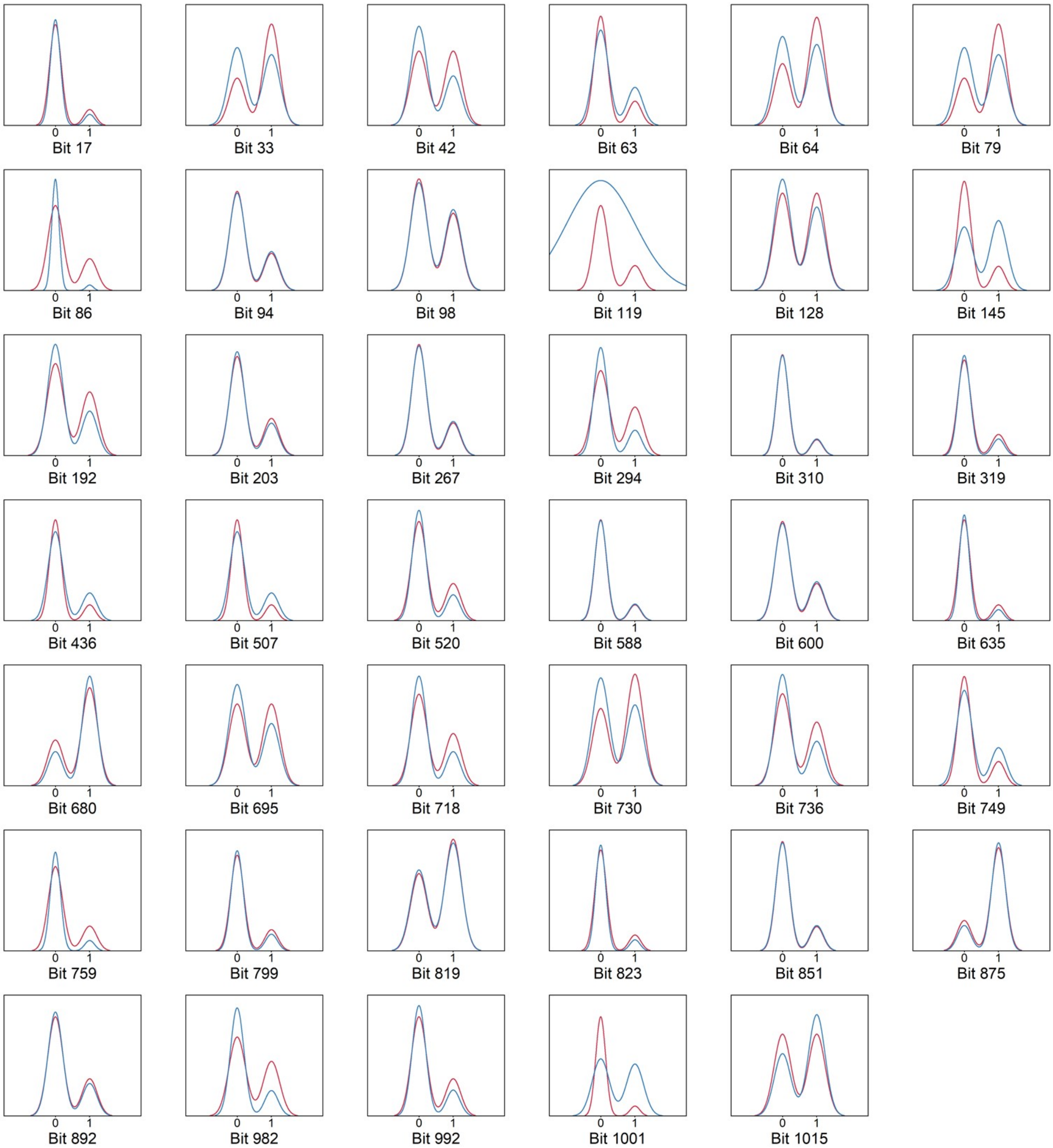

Figure S9. Plot of the 41 Morgan fingerprint descriptors and their distributions, separated by acetone solubility classification (soluble, red; insoluble, blue) for the training set.

Please do not adjust margins

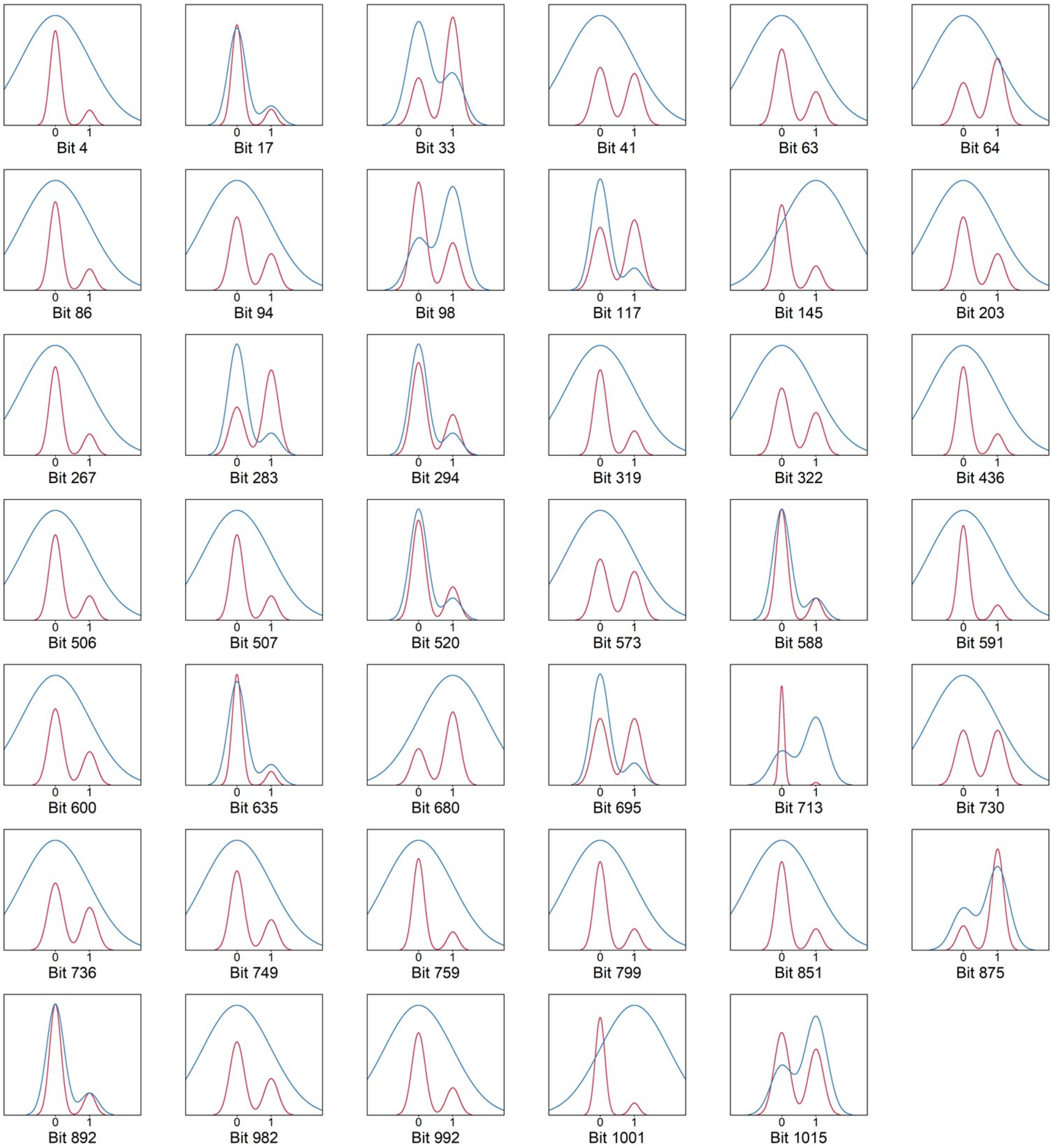

Figure S10. Plot of the 41 Morgan fingerprint descriptors and their distributions, separated by DMF solubility classification (soluble, red; insoluble, blue) for the training set.

 This journal is © The Royal Society of Chemistry 20xx

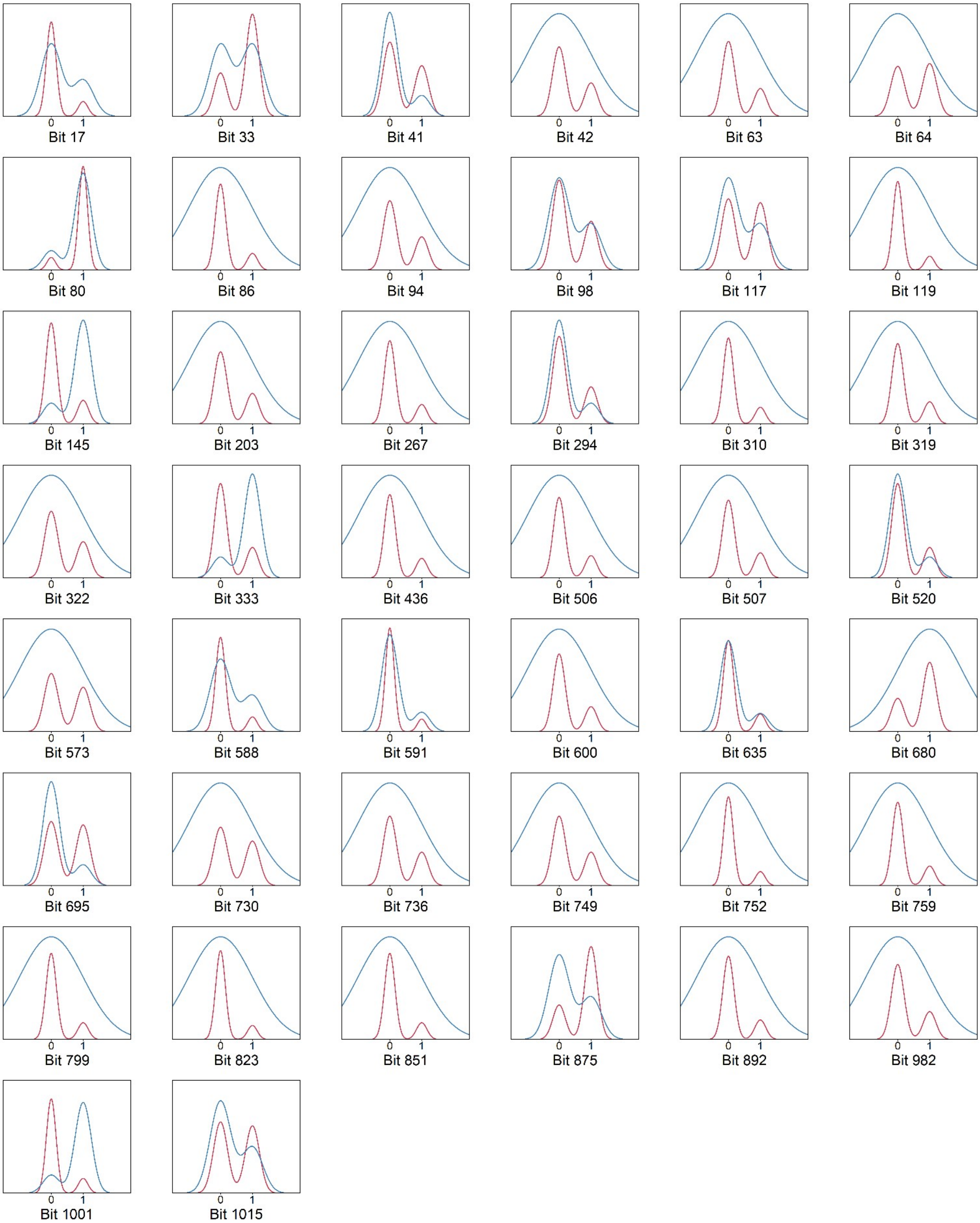

Figure S11. Plot of the 44 Morgan fingerprint descriptors and their distributions, separated by DMSO solubility classification (soluble, red; insoluble, blue) for the training set.

Please do not adjust margins

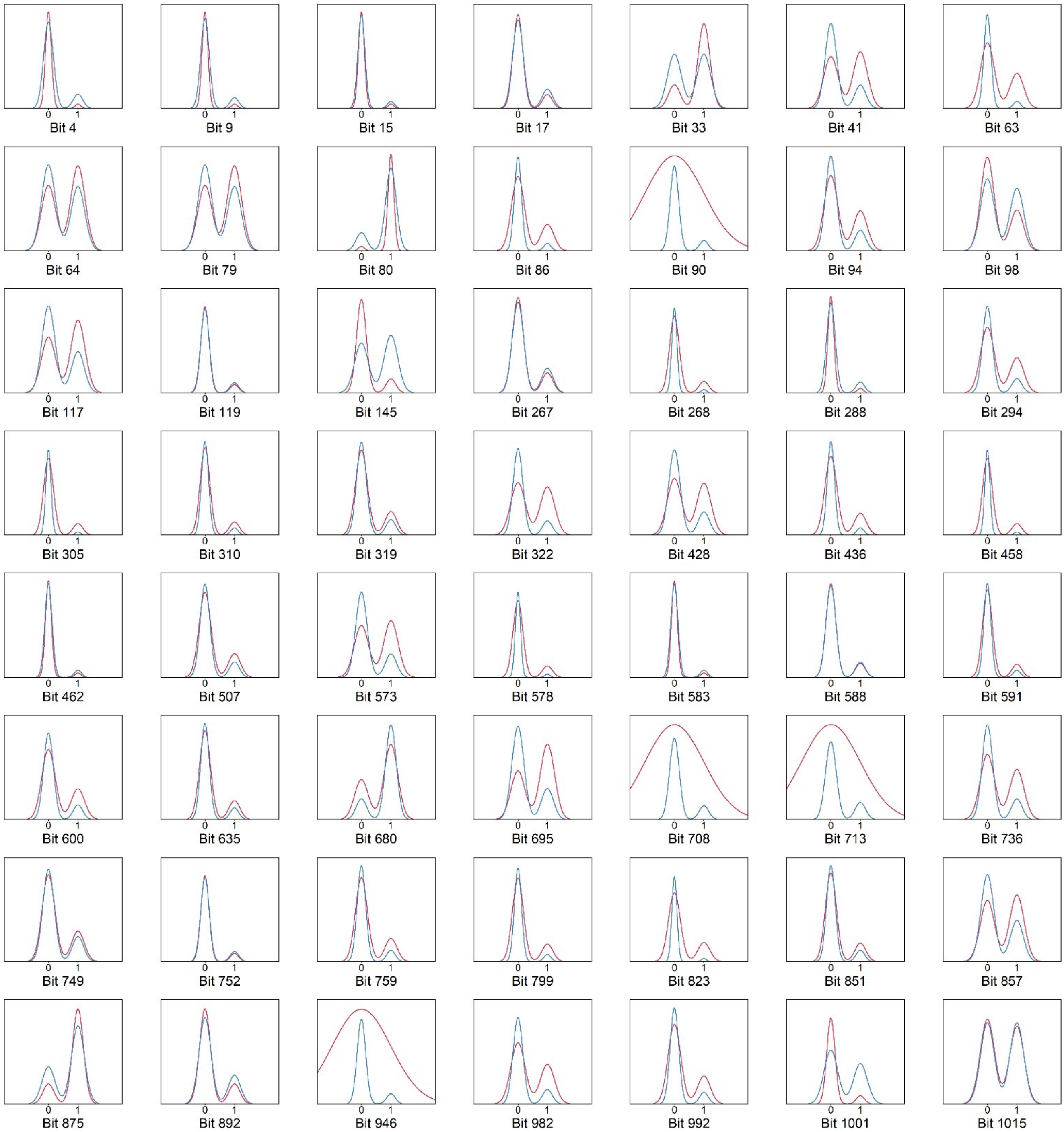

Figure S12. Plot of the 56 Morgan fingerprint descriptors and their distributions, separated by ethanol solubility classification (soluble, red; insoluble, blue) for the training set.

Please do not adjust margins

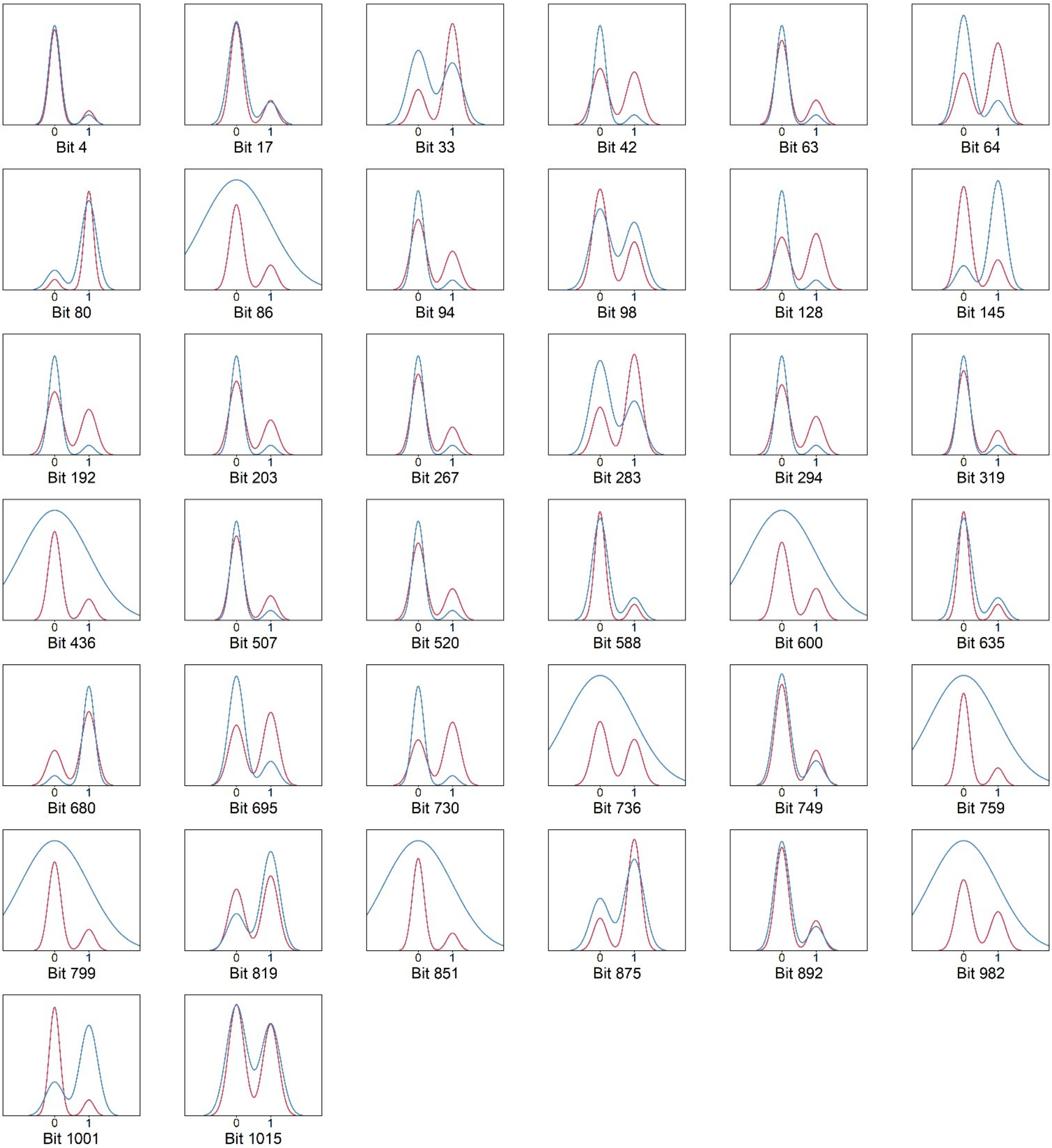

Figure S13. Plot of the 38 Morgan fingerprint descriptors and their distributions, separated by THF solubility classification (soluble, red; insoluble, blue) for the training set.

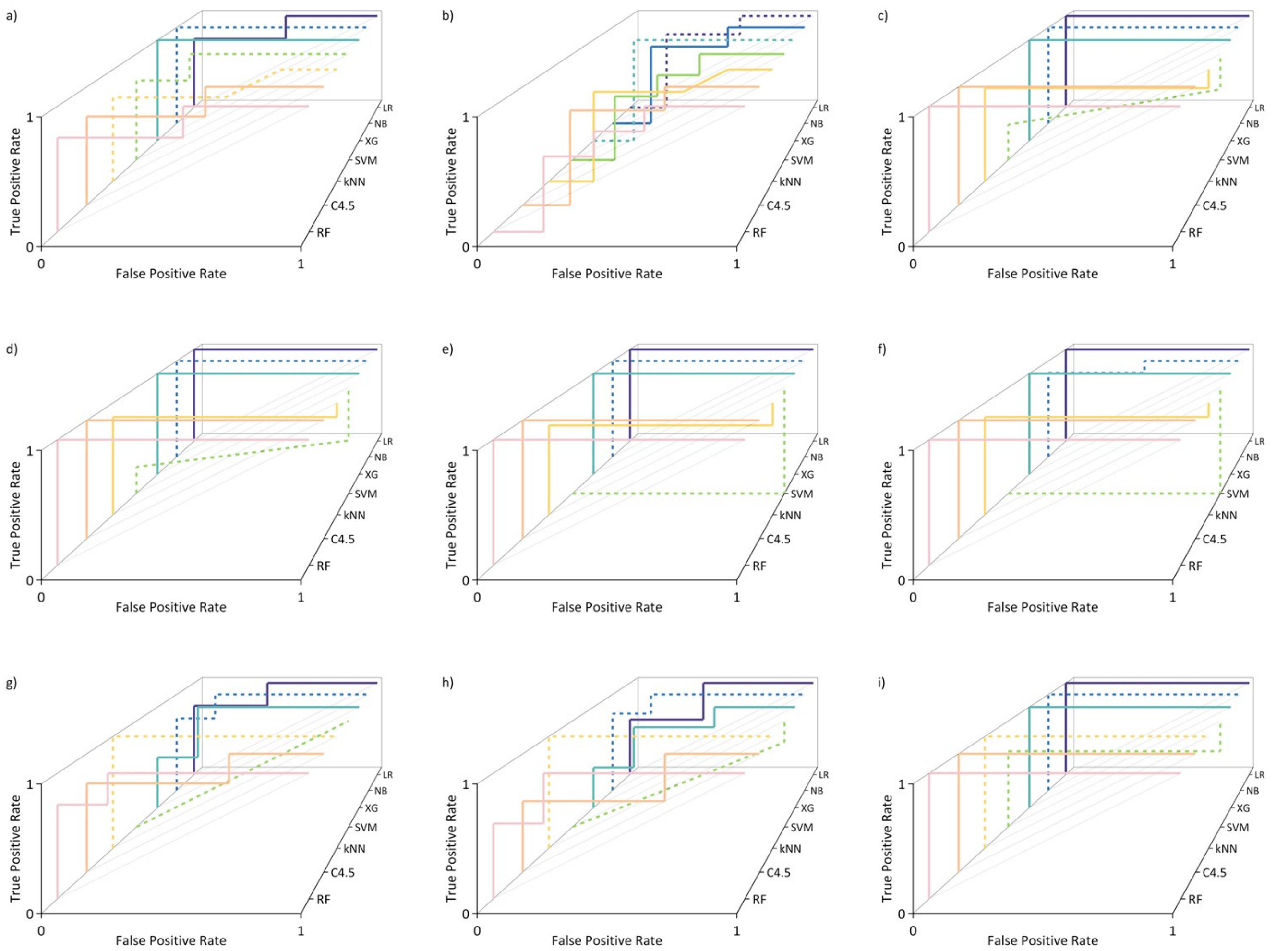

Figure S14. Receiver operator characteristic (ROC) curves for all reported optimised models. Curves with a solid line represent models with good predictive statistics, while curves with a dashed line represents models with bad predictive statistics, as described in Table S3 and S4. Curves have been separated on the basis of solvent model and applicability domain as follows: a) acetone, dsz; b) acetone, maxdist; c) DMF, dsz; d) DMF, maxdist; e) DMSO, dsz; f) DMSO, maxdist; g) ethanol, dsz; h) ethanol, maxdist; i) THF, dsz and maxdist (identical roc curves).

This journal is © The Royal Society of Chemistry 20xx

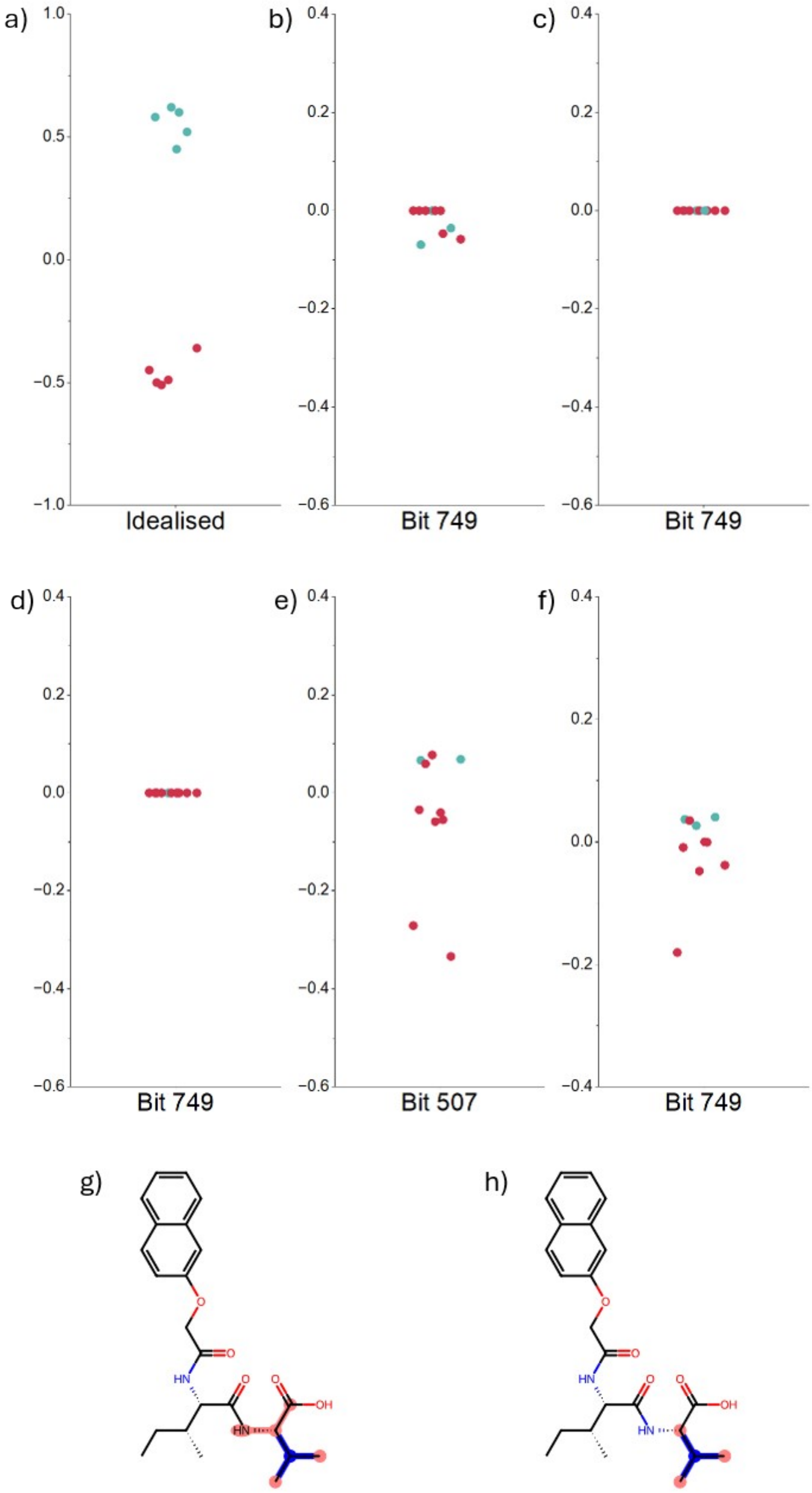

Figure S15. Butterfly plots of Shapley values for test set compound predictions on the optimised models. Green data points indicate a compound for which the descriptor is present, and red data points indicate its absence. a) shows an idealised Shapley value plot that shows a clear relationship between the presence of a descriptor and its classification. b – f) are examples of Shapley value butterfly plots obtained with the reported acetone, DMF, DMSO, ethanol, and THF XGBoost models, respectively. The plots demonstrate the impact of including a valine substructure on the solubility of the resulting compound. While the inclusion of valine moieties affords little change in the solubility in DMF and DMSO, the models suggest that their presence simultaneously reduces solubility in acetone while increasing solubility in ethanol and THF. g) and h) show representative structures with the bit 507 and bit 749 substructure highlighted, respectively.

Please do not adjust margins
Please do not adjust margins

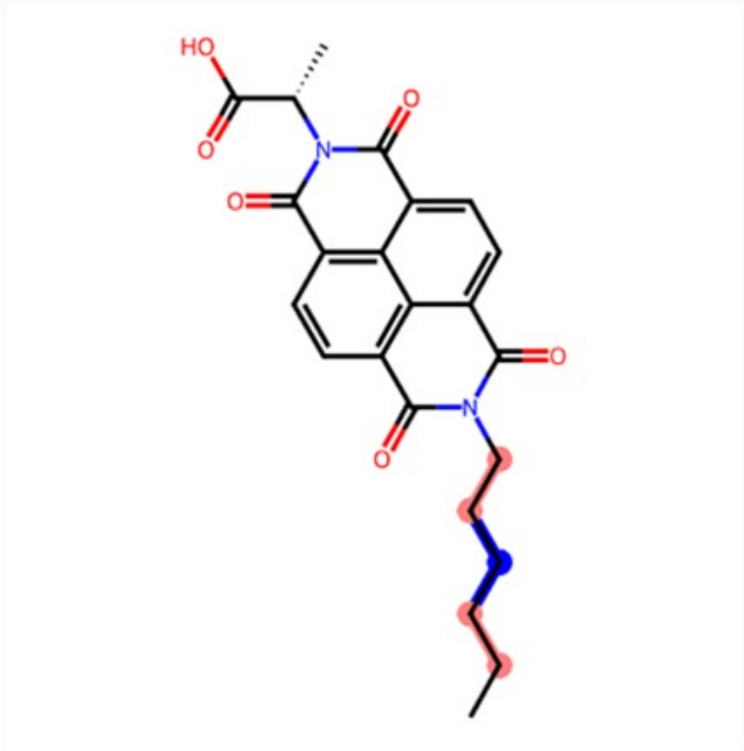

Bit 119 (1.48)

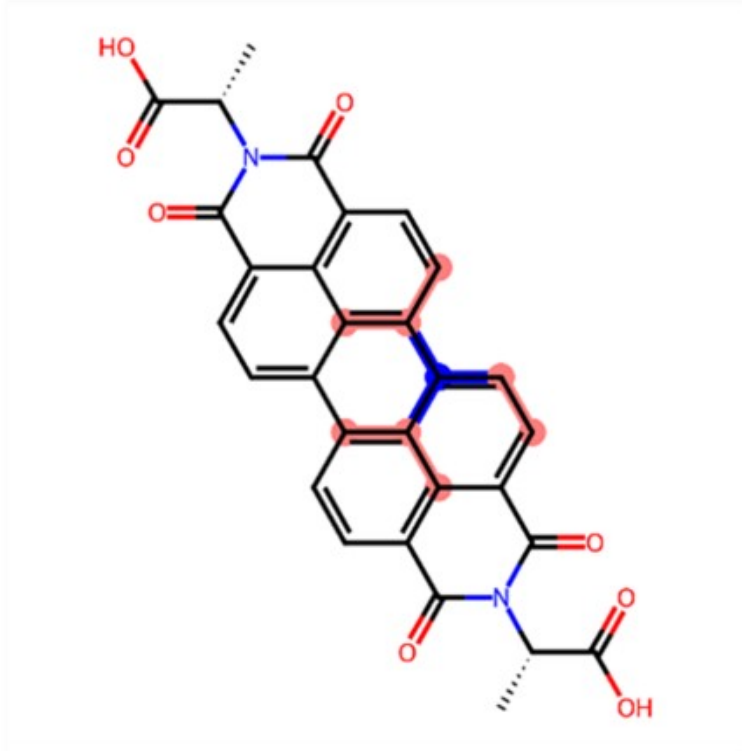

Bit 1001 (1.25)

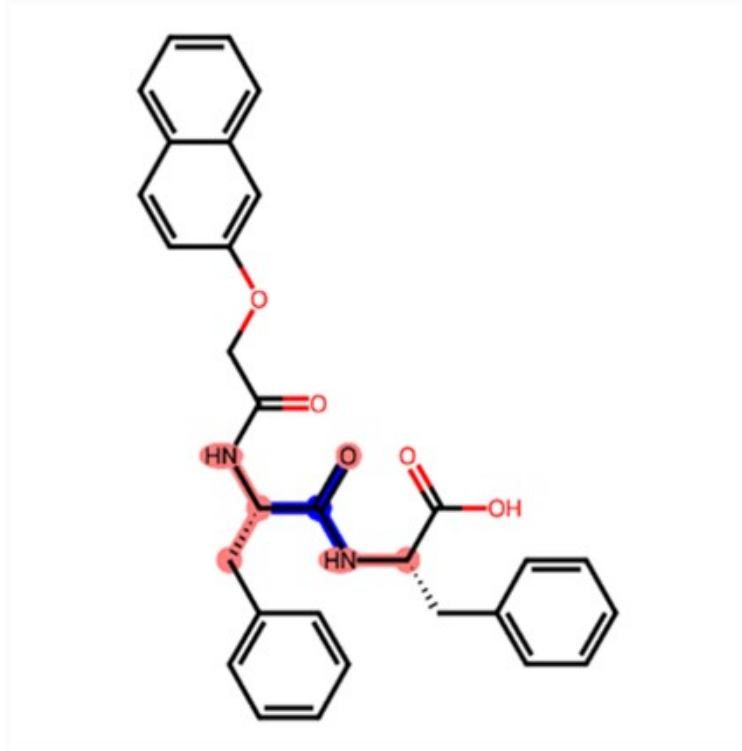

Bit 86 (0.90)

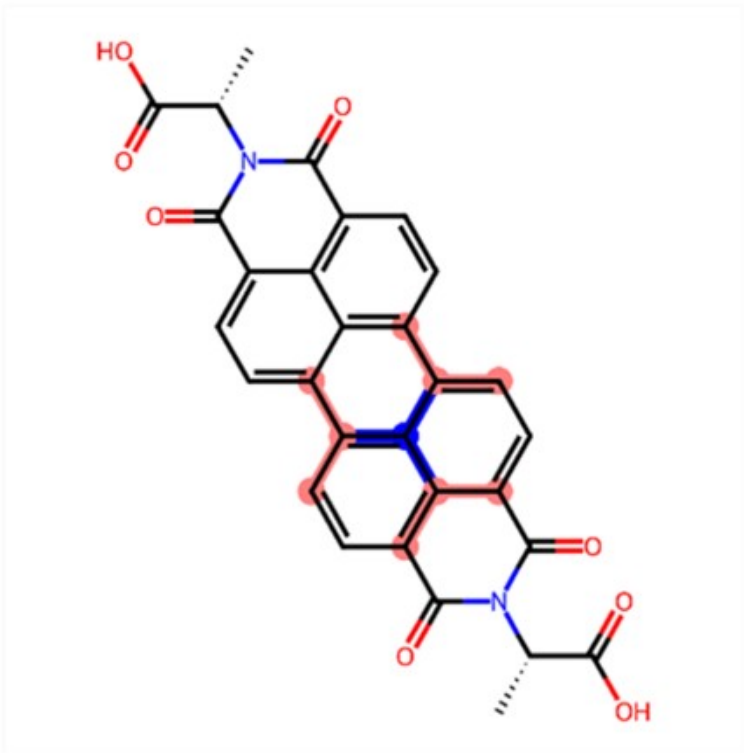

Bit 145 (0.87)

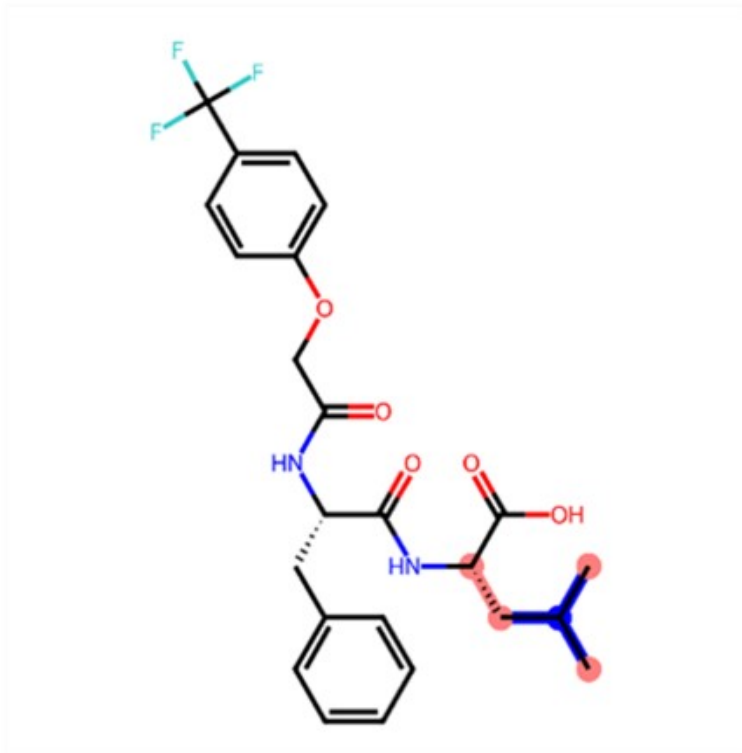

Bit 759 (0.62)

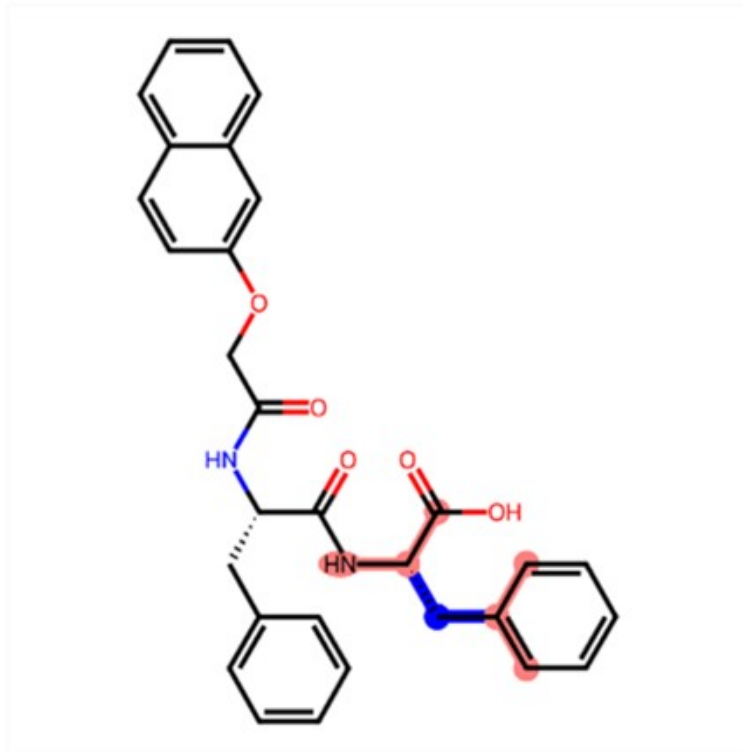

Bit 982 (0.50)

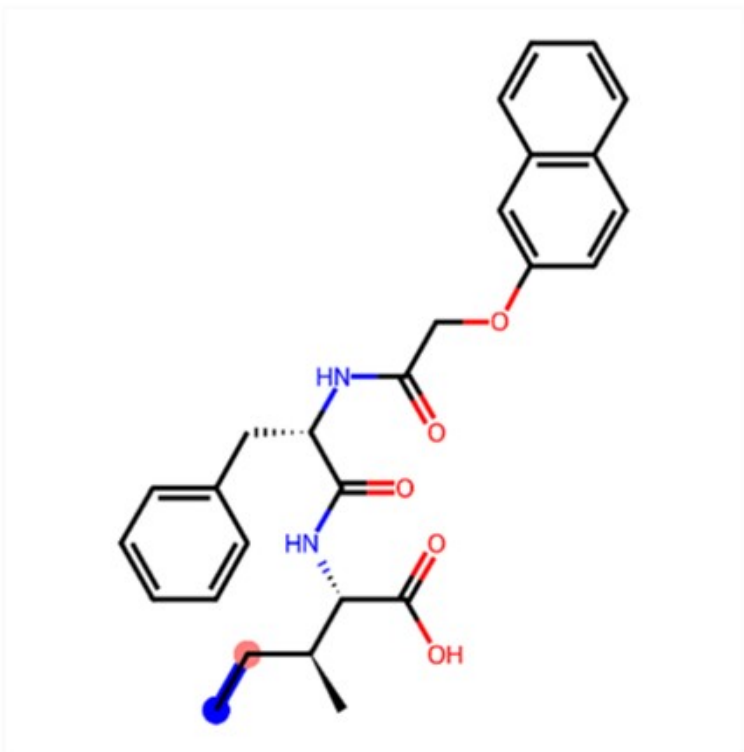

Bit 294 (0.49)

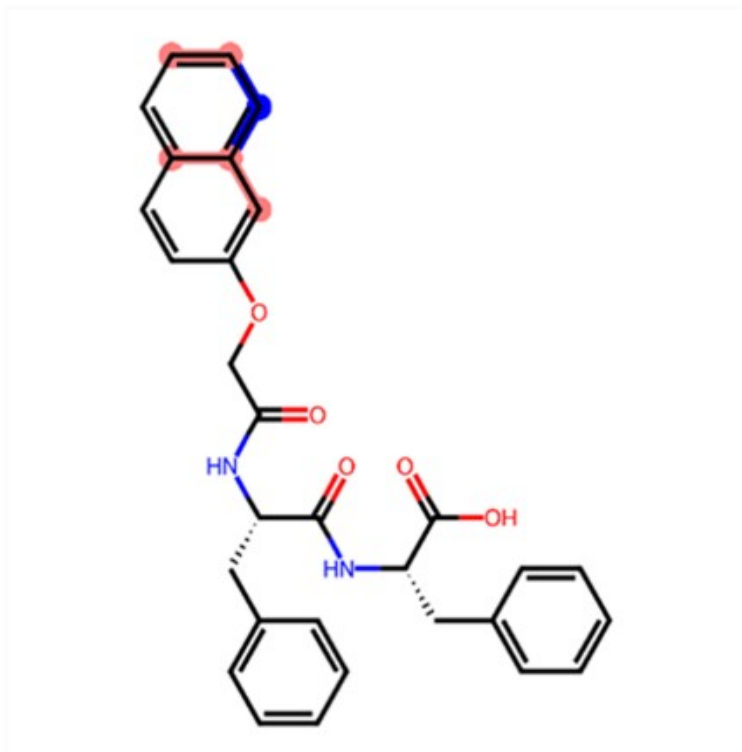

Bit 63 (0.45)

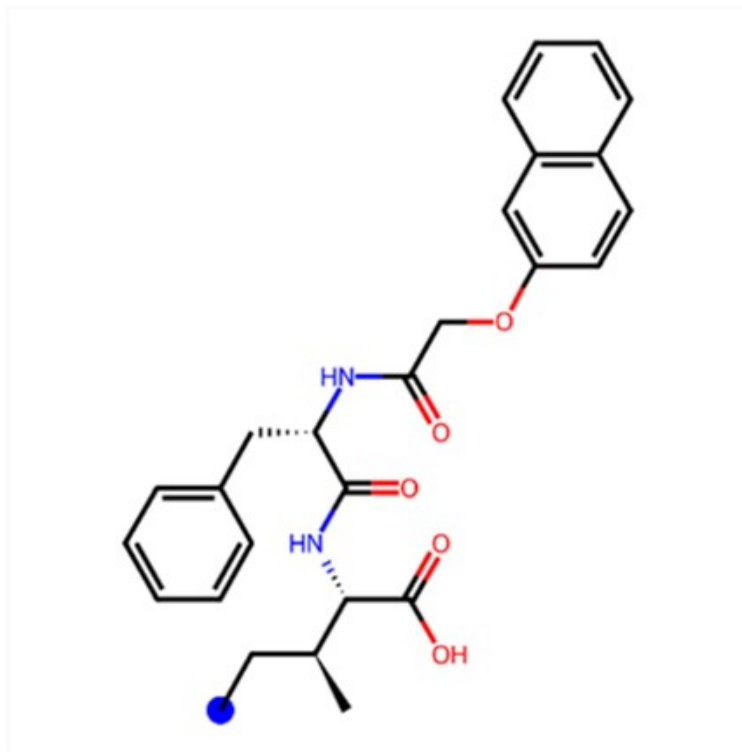

Bit 33 (0.45)

Figure S16. Descriptor feature importance of the 9 most important substructures for the acetone model. The core atom represented by the fingerprint bit of interest is highlighted in blue, with the extended connectivity captured by the structural descriptor highlighted in red. The quantifed importance (Equation 1) of each of these descriptors is also provided.

 This journal is © The Royal Society of Chemistry 20xx

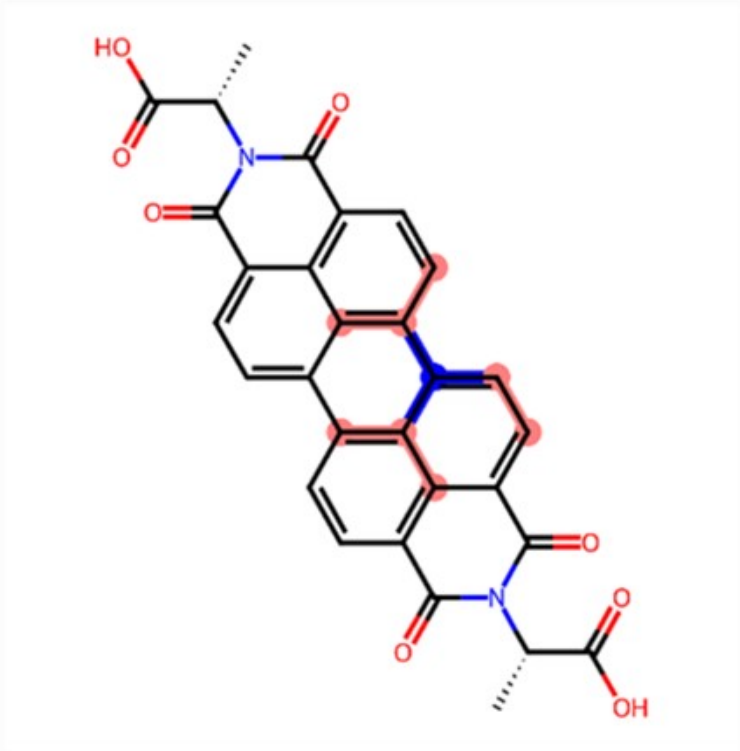

Bit 1001 (1.81)

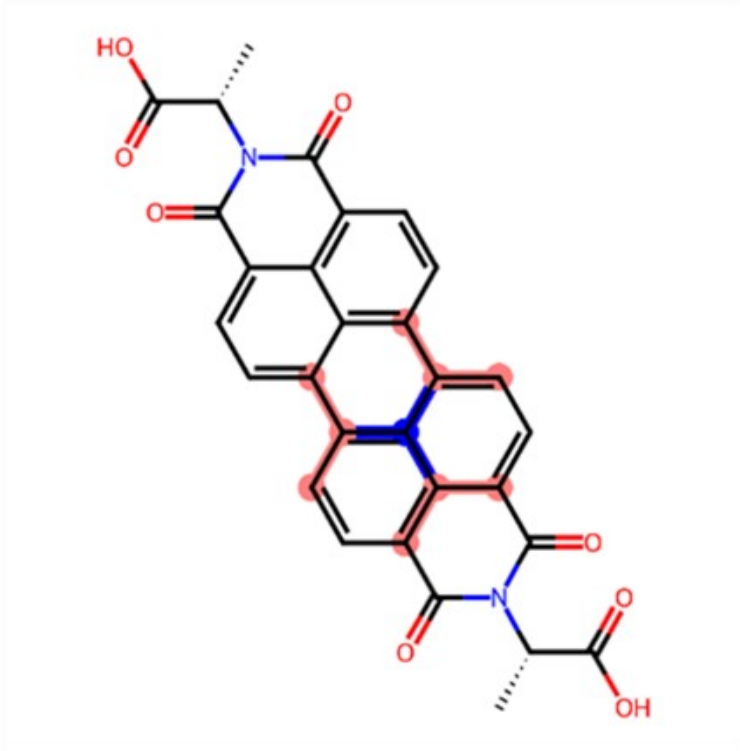

Bit 145 (1.49)

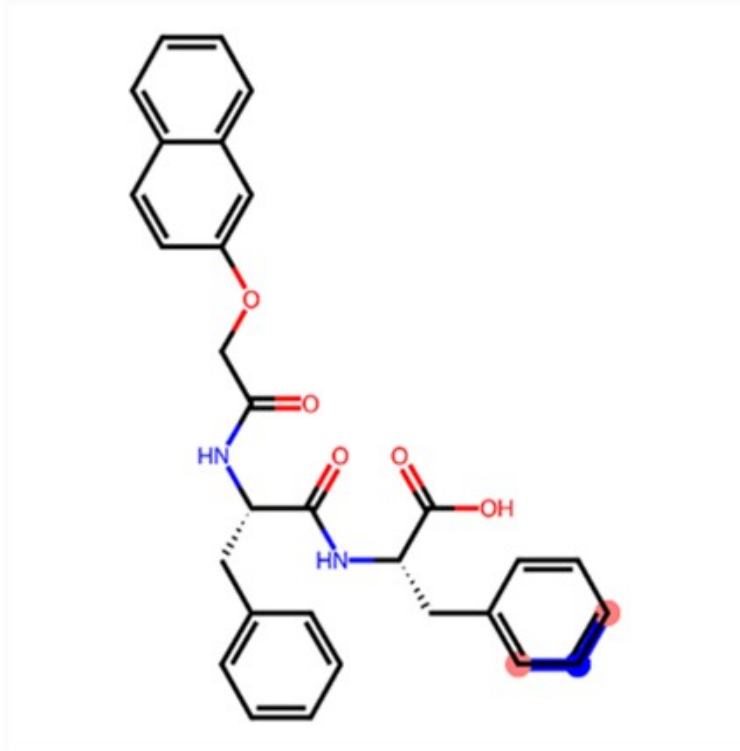

Bit 64 (1.23)

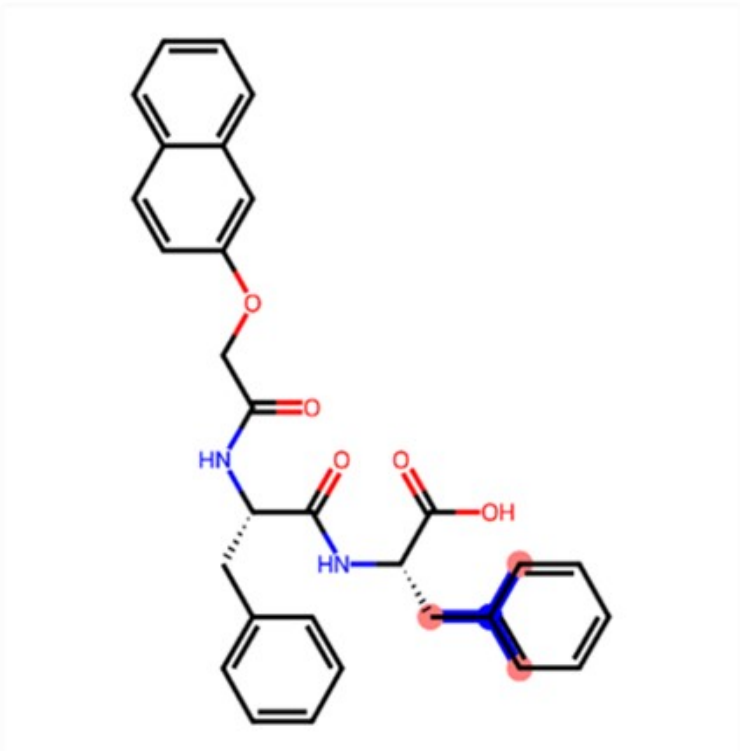

Bit 730 (1.04)

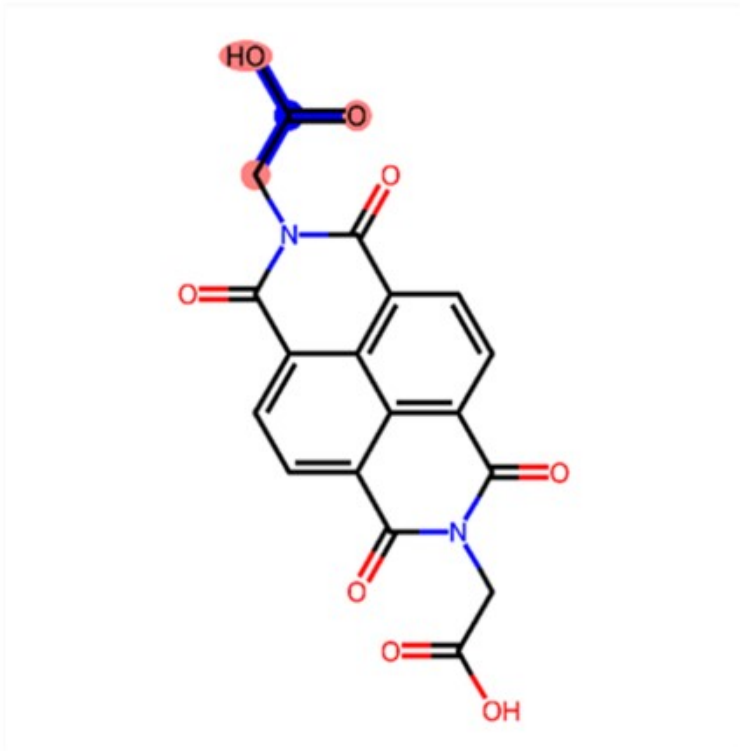

Bit 713 (0.95)

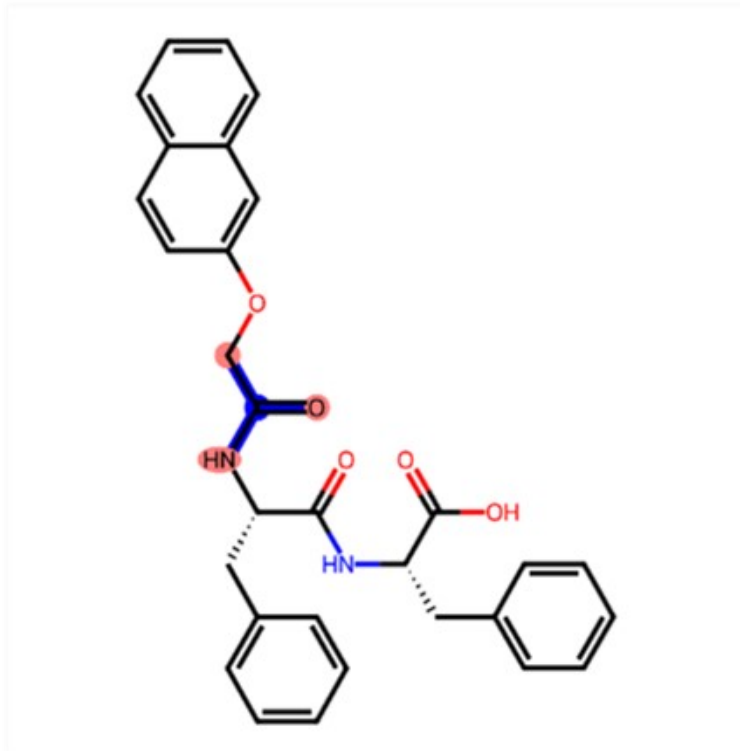

Bit 41 (0.65)

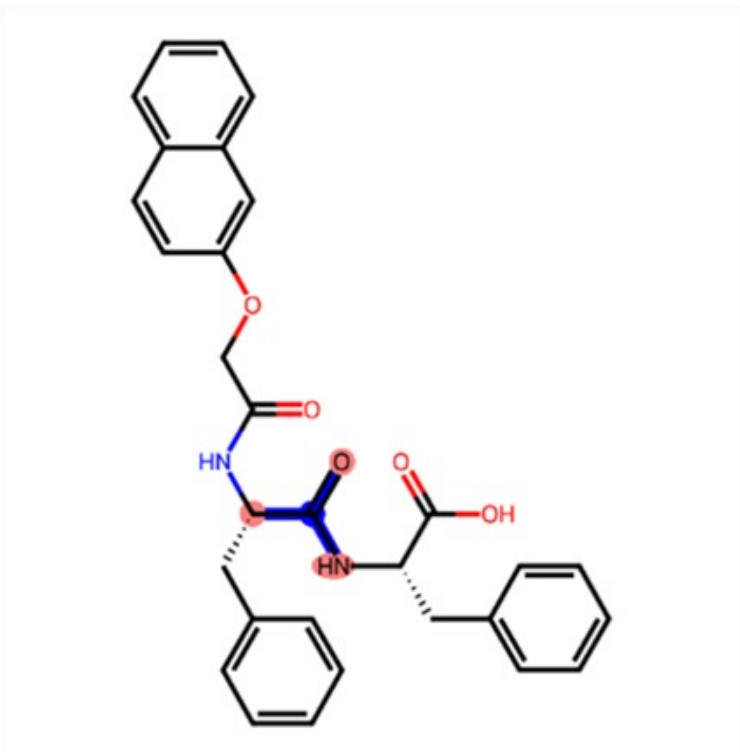

Bit 573 (0.55)

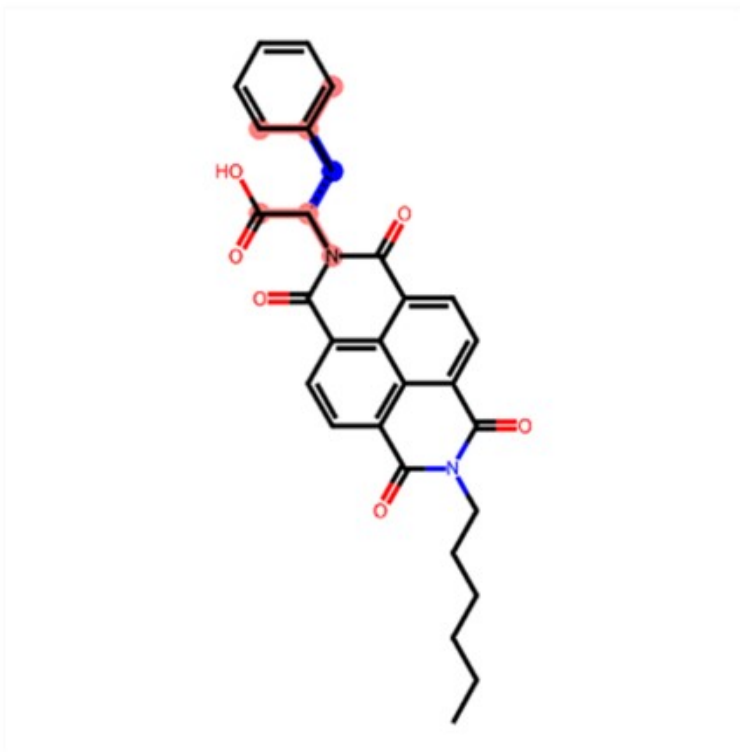

Bit 267 (0.46)

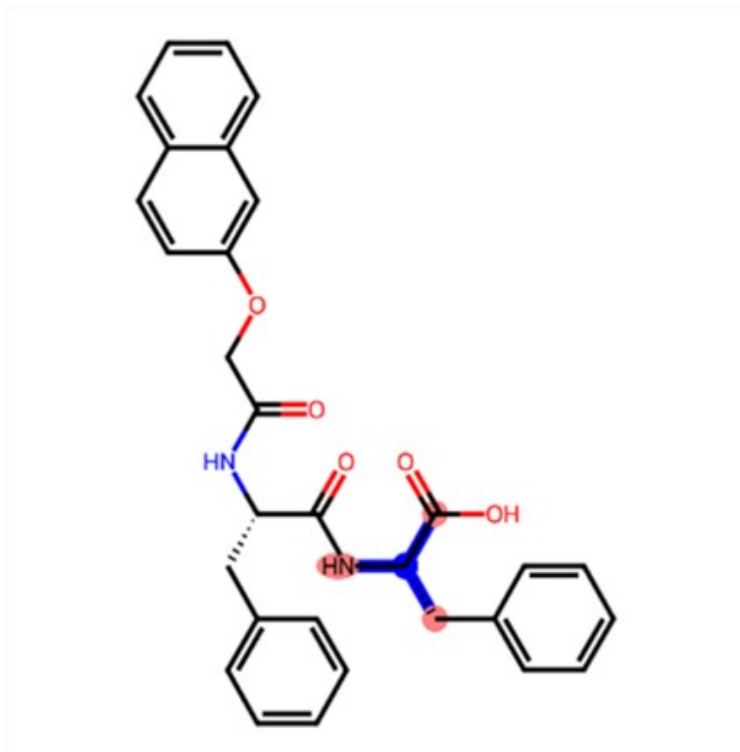

Bit 736 (0.38)

Figure S17. Descriptor feature importance of the 9 most important substructures for the DMF model. The core atom represented by the fingerprint bit of interest is highlighted in blue, with the extended connectivity captured by the structural descriptor highlighted in red. The quantifed importance (Equation 1) of each of these descriptors is also provided.

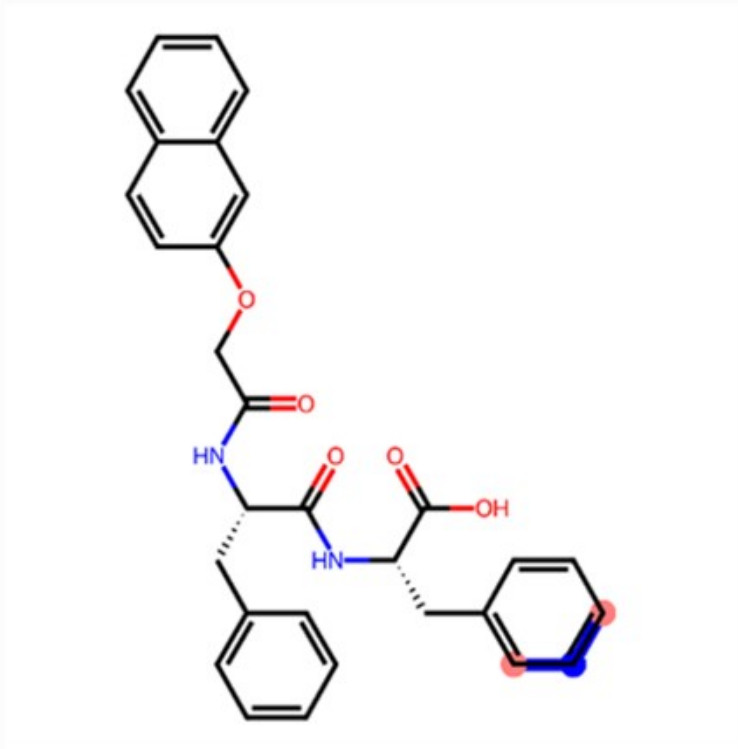

Bit 64 (1.36)

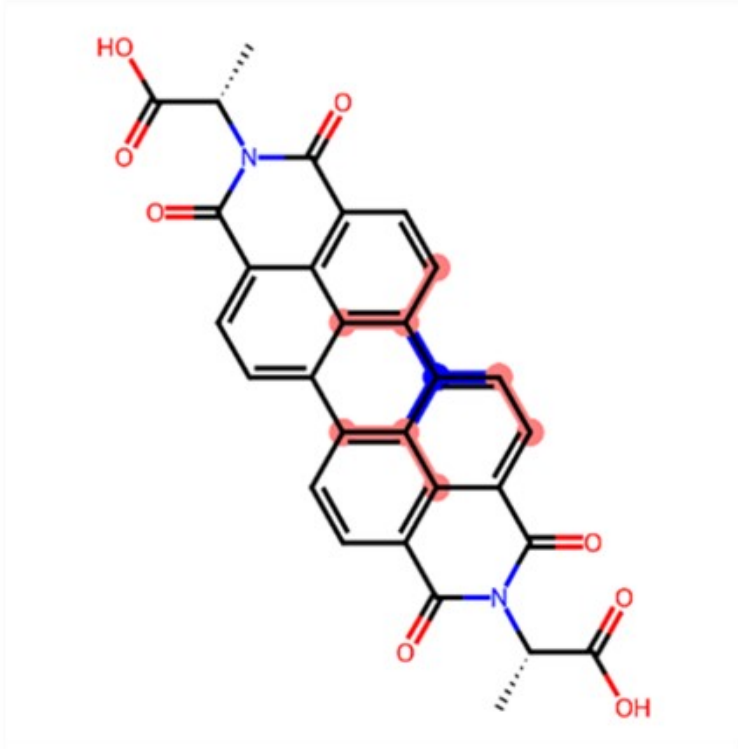

Bit 1001 (1.21)

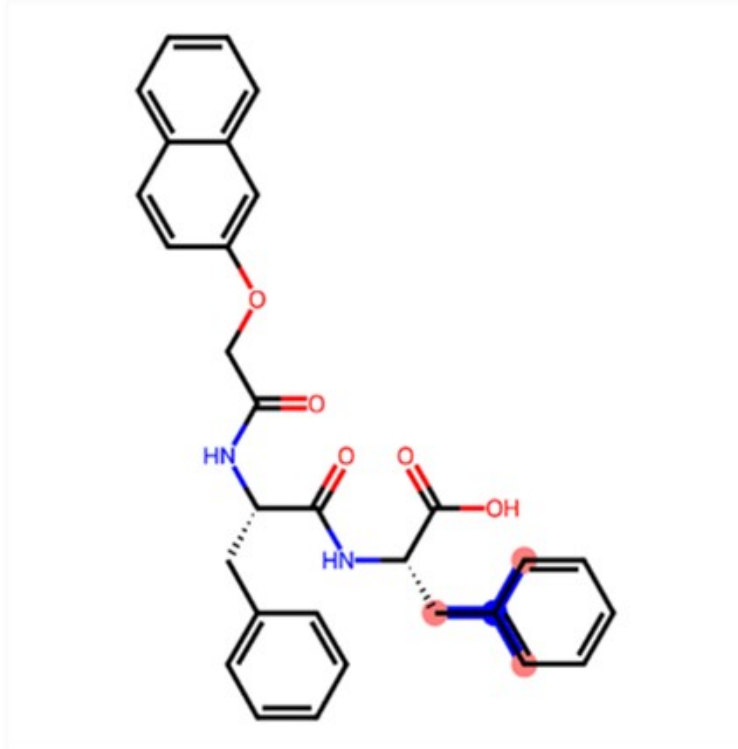

Bit 730 (1.19)

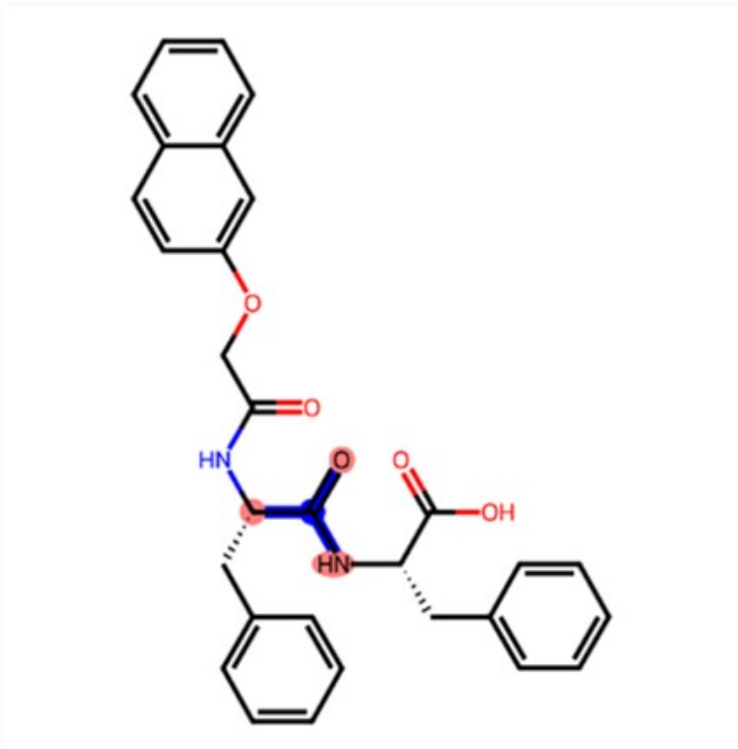

Bit 573 (0.95)

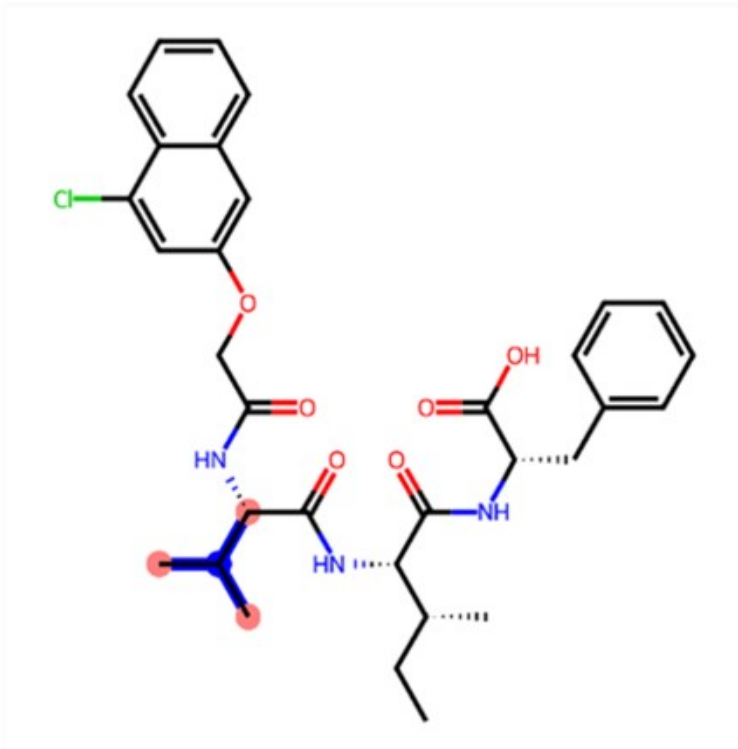

Bit 749 (0.82)

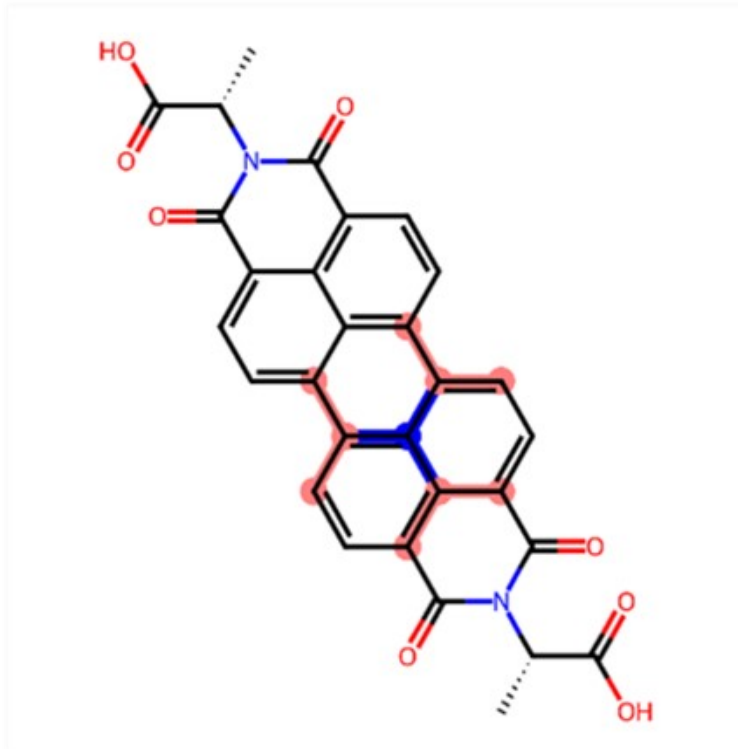

Bit 145 (0.73)

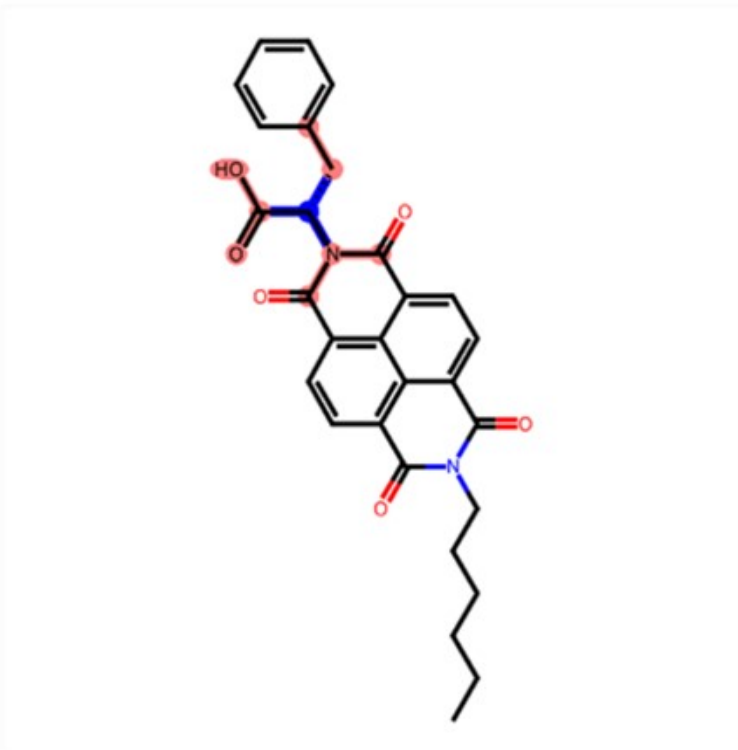

Bit 892 (0.61)

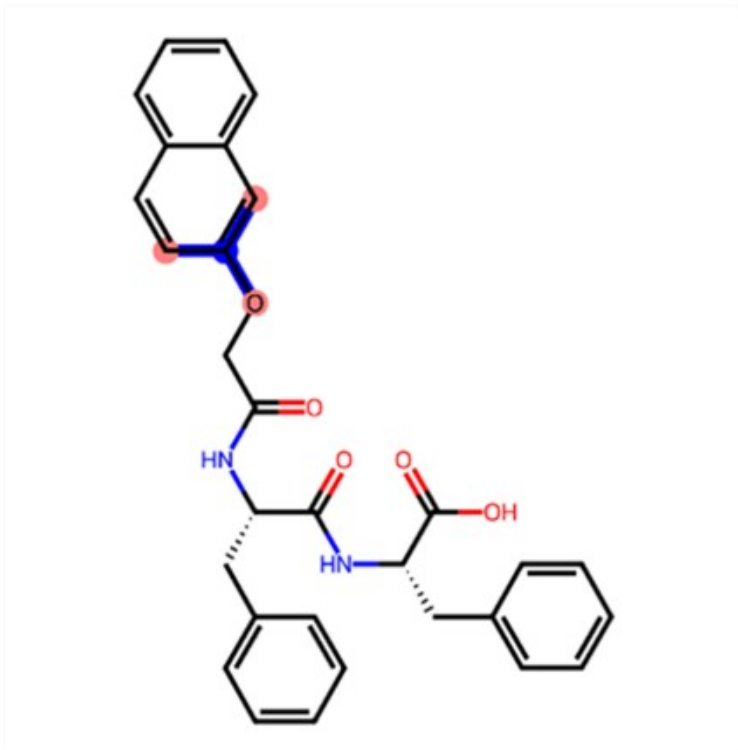

Bit 322 (0.54)

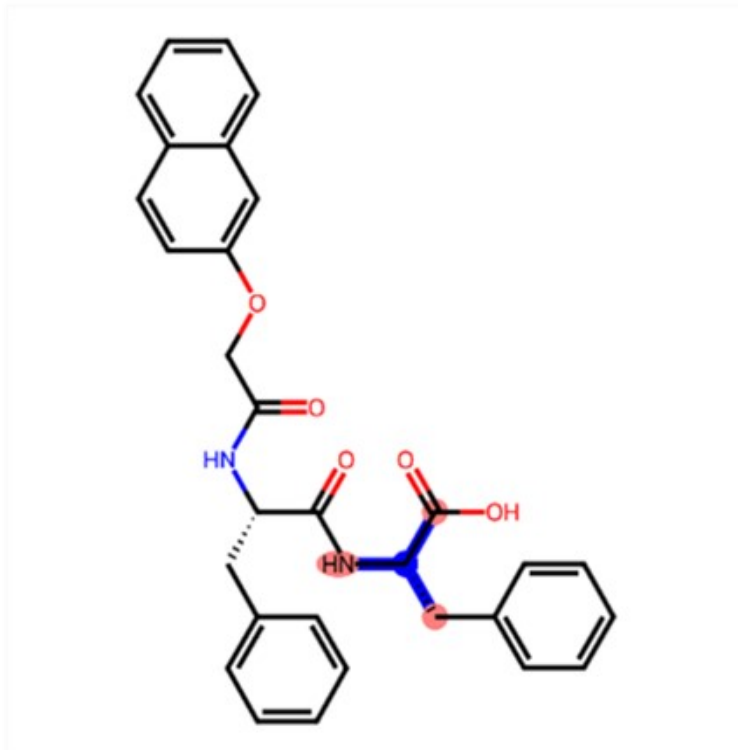

Bit 736 (0.49)

Figure S18. Descriptor feature importance of the 9 most important substructures for the DMSO model. The core atom represented by the fingerprint bit of interest is highlighted in blue, with the extended connectivity captured by the structural descriptor highlighted in red. The quantifed importance (Equation 1) of each of these descriptors is also provided.

This journal is © The Royal Society of Chemistry 20xx

Rotatable Bonds (1.20)

Labute Atomic Surface Area (1.18)

Number of Atoms (1.15)

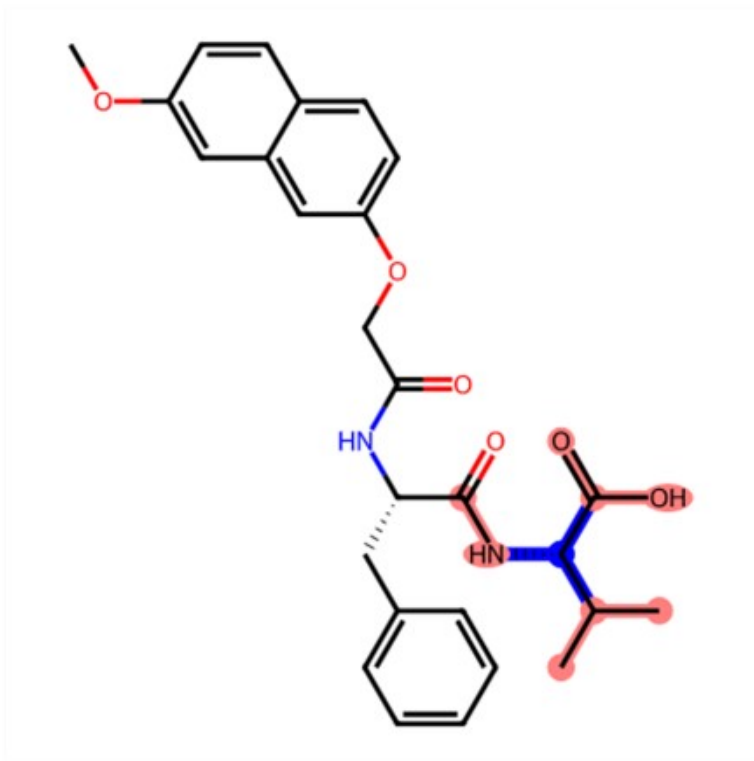

SLogP (1.08)

Bit 752 (0.856)

Number of Rings (0.848)

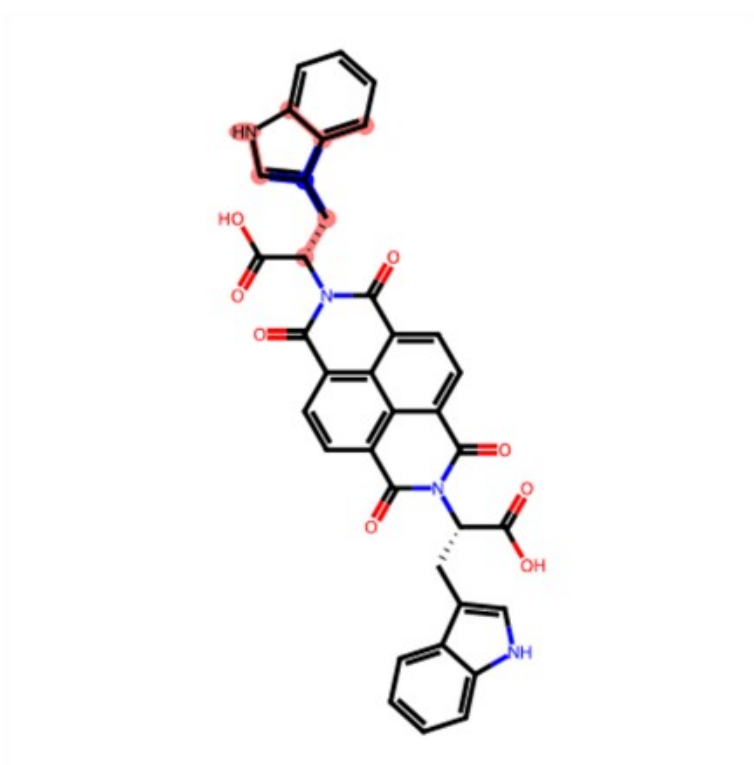

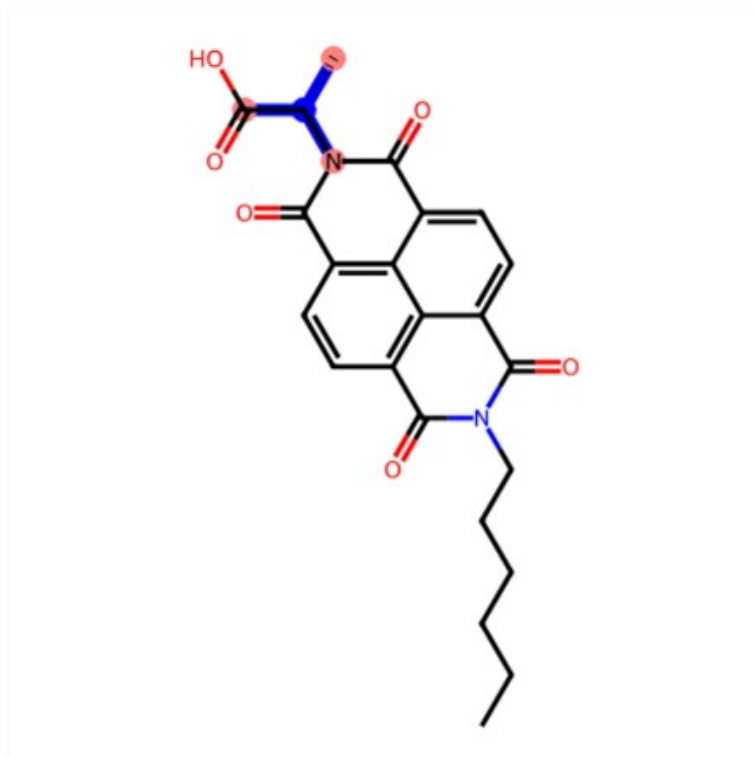

Total Polar Surface Area (0.68)

Bit 946 (0.48)

Bit 708 (0.48)

Figure S19. Descriptor feature importance of the 9 most important substructures for the ethanol model. The core atom represented by the fingerprint bit of interest is highlighted in blue, with the extended connectivity captured by the structural descriptor highlighted in red. The quantifed importance (Equation 1) of each of these descriptors is also provided.

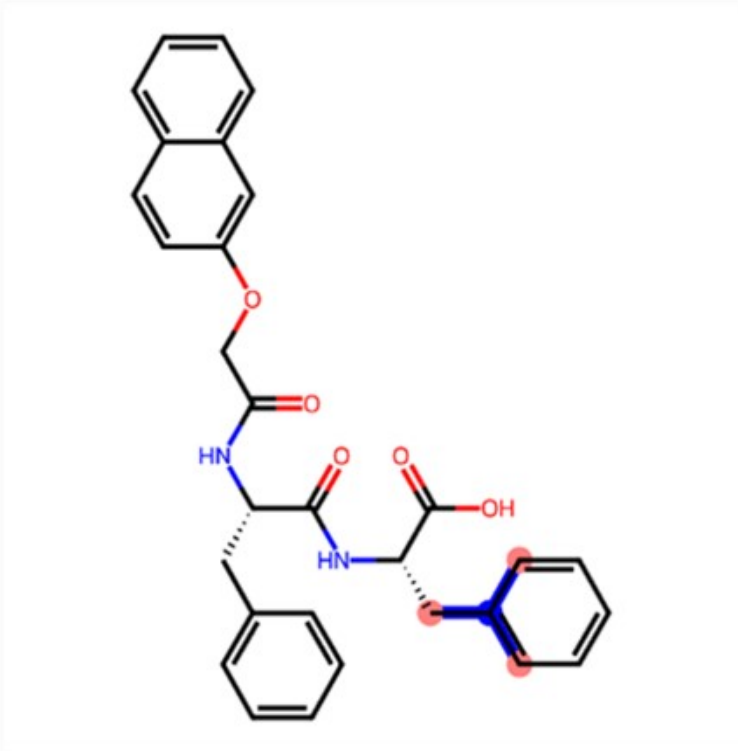

Bit 730 (1.31)

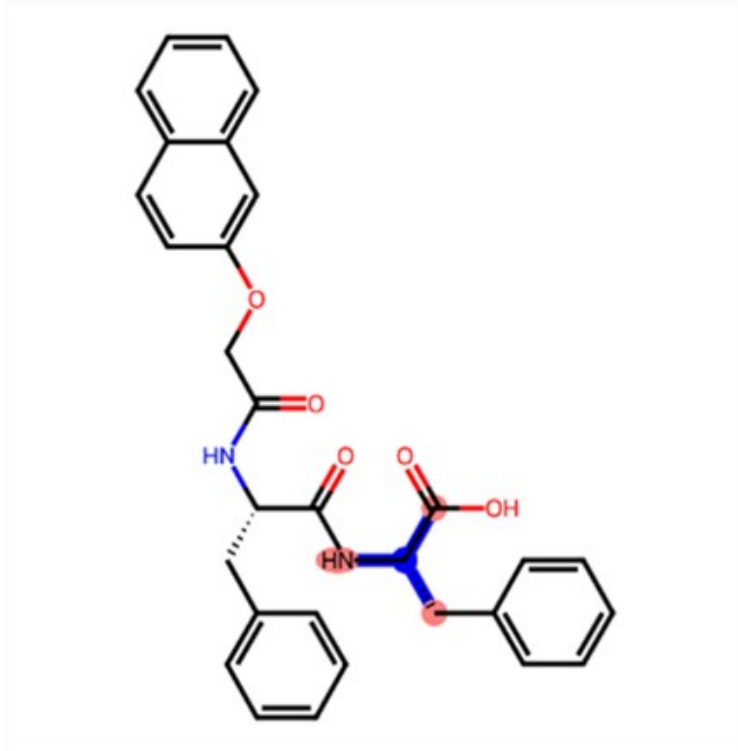

Bit 736 (1.14)

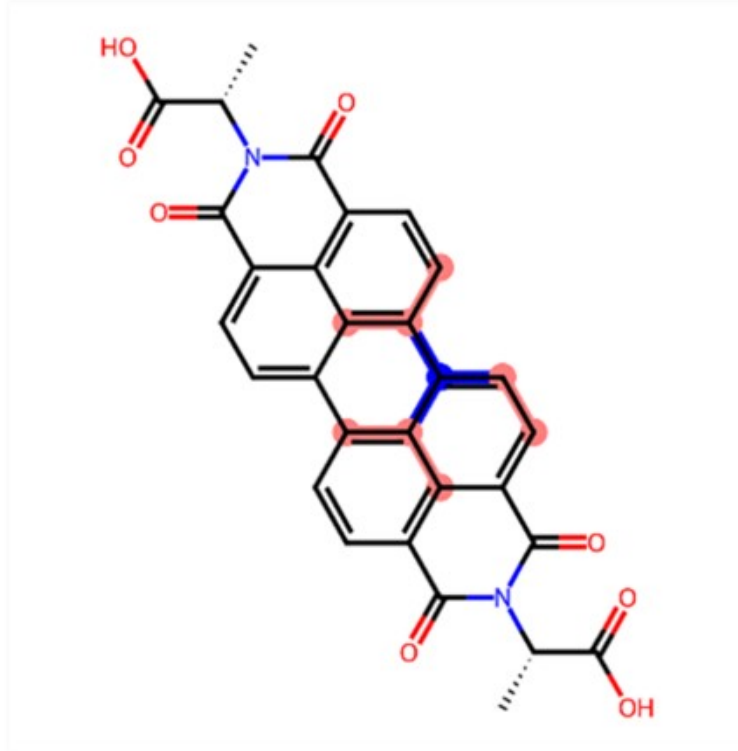

Bit 1001 (1.08)

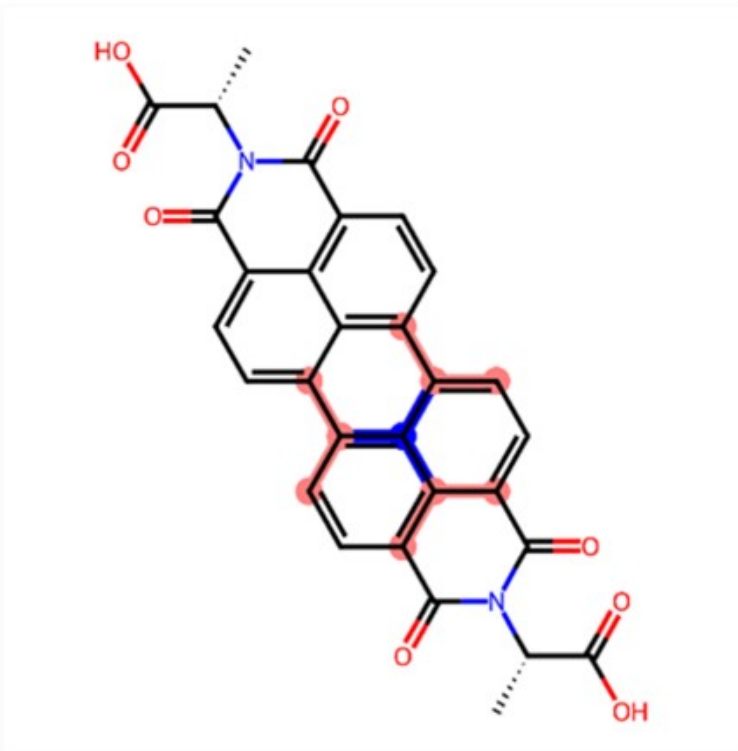

Bit 145 (0.97)

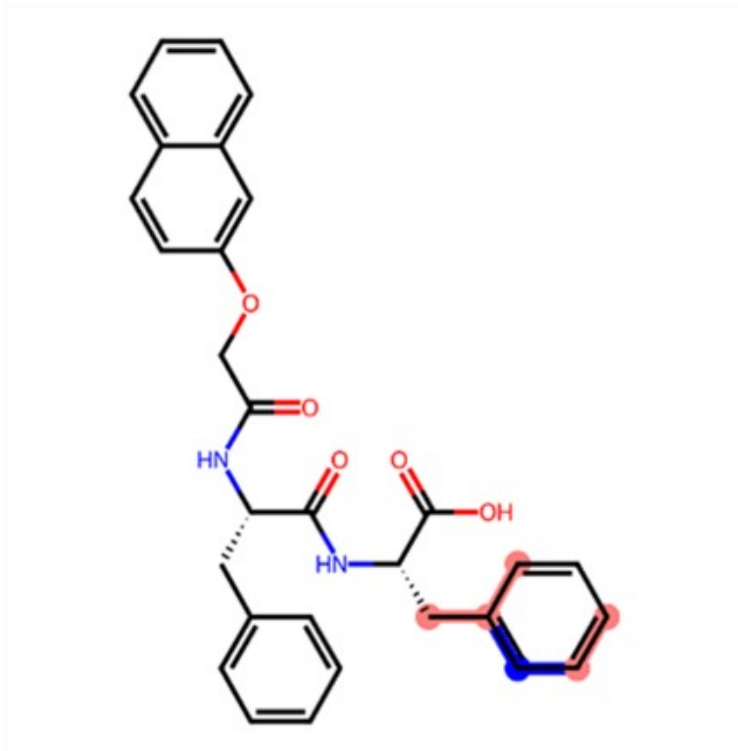

Bit 42 (0.80)

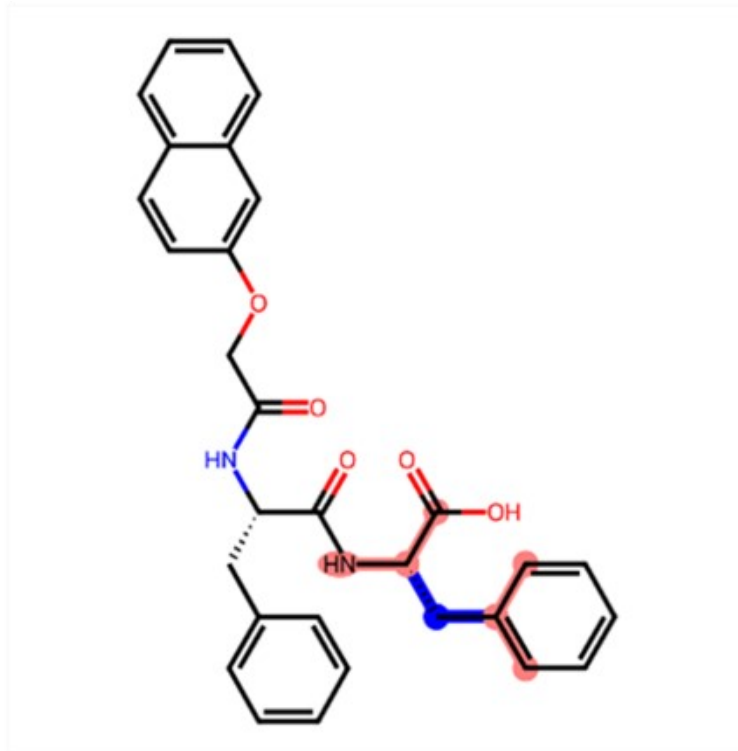

Bit 982 (0.759)

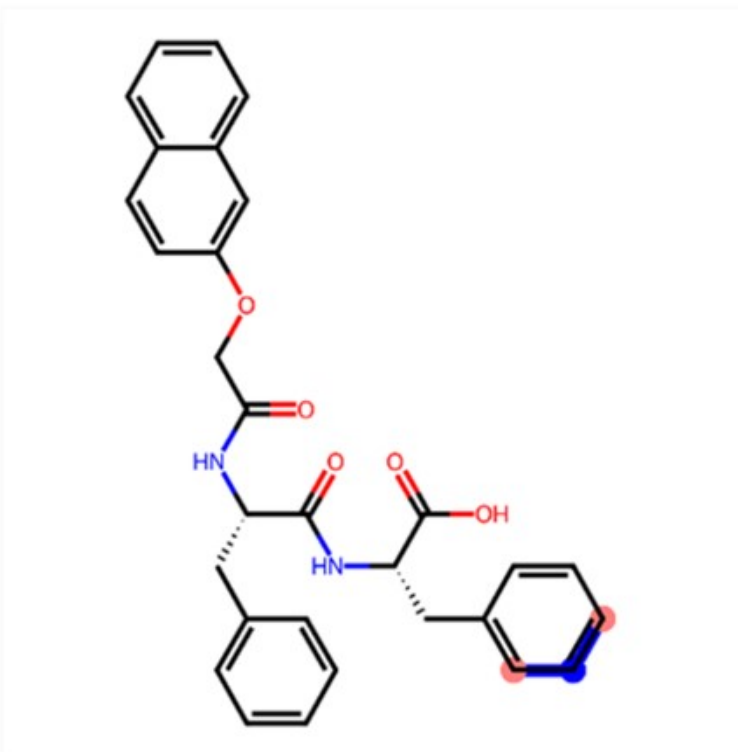

Bit 64 (0.66)

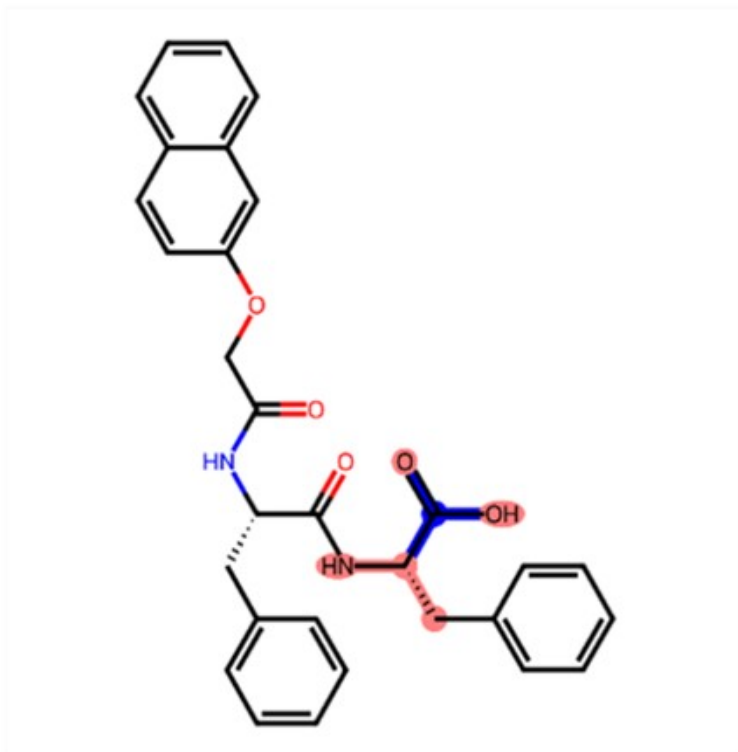

Bit 600 (0.64)

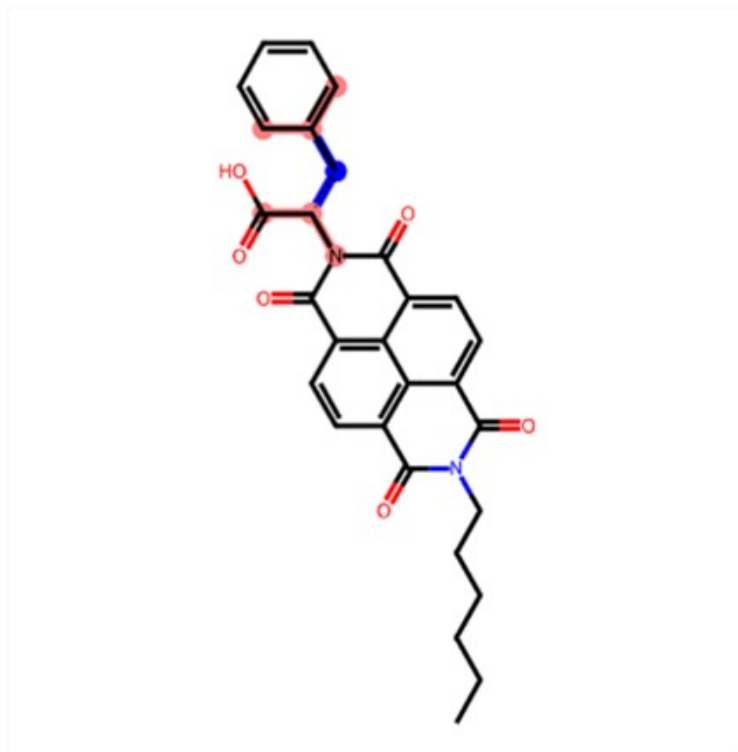

Bit 267 (0.58)

Figure S20. Descriptor feature importance of the 9 most important substructures for the THF model. The core atom represented by the fingerprint bit of interest is highlighted in blue, with the extended connectivity captured by the structural descriptor highlighted in red. The quantified importance (Equation 1) of each of these descriptors is also provided.

This journal is © The Royal Society of Chemistry 20xx

Supplementary Tables

Table S4 Hyperparameter combinations optimised by 5-fold cross-validation procedure.

Model	Parameter	Start	Stop	Step
RF	Depth	10	100	10
	Number of Trees	1000	5000	1000
C4.5	Minimum Node Size	2	10	2
	Number of Trees	1000	5000	1000
	M_{Try}	0.2	0.8	0.2
	Sample Fraction	0.2	0.8	0.2
	Depth	10	100	10
kNN	Number of Neighbours	3	20	1
SVM	σ	-10	10	0.05
XG [a]	Depth	10	100	10
	Rounds	1000	5000	1000
	Learning Rate	0.2	0.8	0.2
LR	Learning Rate	0.1	0.8	0.1
	Variance	0.1	0.8	0.1

a For simplicity, the log10 of the SVM σ hyperparameter input has been reported. Therefore, while all other parameters use a linear interval, the σ interval represents the logarithmic interval of each iteration

Please do not adjust margins

Table S5 Optimisation and performance statistics of the QSPR models developed for the training set

		Resampling results of optimal model	Performance of optimal model on training set				
Model	Method	MCC	ROC AUC	Balanced Accuracy	MCC	K	Overall quality of model
Acetone	RF	0.183	0.973	0.932	0.869	0.861	Good
	C4.5	0.420	0.938	0.861	0.725	0.721	Good
	kNN	0.229	0.705	0.652	0.303	0.303	Bad
	SVM	0.358	0.763	0.500	ND	0.000	Bad
	XG	0.410	0.971	0.907	0.814	0.814	Good
	NB		0.806	0.726	0.504	0.448	Good
	LR	0.228	0.884	0.883	0.768	0.767	Good
DMF	RF	0.946	1.000	0.986	0.913	0.909	Good
	C4.5	0.976	1.000	0.986	0.913	0.909	Good
	kNN	0.798	1.000	0.889	0.577	0.500	Good
	SVM	0.821	0.317	0.500	ND	0.000	Bad
	XG	0.973	1.000	1.000	1.000	1.000	Good
	NB		0.991	0.681	0.273	0.139	Bad
	LR	0.894	1.000	0.986	0.913	0.909	Good
DMSO	RF	0.897	0.982	0.986	0.913	0.909	Good
	C4.5	0.947	0.989	0.986	0.913	0.909	Good
	kNN	0.609	0.998	0.851	0.498	0.397	Good
	SVM	0.679	0.236	0.500	ND	0.000	Bad
	XG	0.897	0.993	0.973	0.842	0.830	Good
	NB		0.975	0.608	0.193	0.071	Bad
	LR	0.849	0.989	0.973	0.842	0.830	Good
Ethanol	RF	0.535	1.000	1.000	1.000	1.000	Good
	C4.5	0.585	0.998	0.978	0.952	0.951	Good
	kNN	0.480	0.849	0.752	0.505	0.505	Good
	SVM	0.495	1.000	1.000	1.000	1.000	Good
	XG	0.522	1.000	1.000	1.000	1.000	Good
	NB		0.810	0.609	0.330	0.196	Bad
	LR	0.486	0.944	0.818	0.655	0.647	Good
THF	RF	0.873	0.984	0.955	0.938	0.937	Good
	C4.5	0.873	0.987	0.955	0.938	0.937	Good
	kNN	0.588	0.916	0.826	0.578	0.540	Good
	SVM	0.701	0.535	0.500	ND	0.000	Bad
	XG	0.871	0.990	0.955	0.938	0.937	Good
	NB		0.905	0.726	0.421	0.301	Bad
	LR	0.683	0.969	0.903	0.773	0.767	Good

ND Not defined

This journal is © The Royal Society of Chemistry 20xx

Please do not adjust margins

Please do not adjust margins

Table S6 Optimisation and performance statistics of the QSPR models developed for the in-domain external test set compounds using the maxdist applicability domain.

		Performance of optimal model on maxdist in-domain test set				
Model	Method	ROC AUC	Balanced Accuracy	MCC	K	Overall quality of model
Acetone	RF	0.680	0.700	0.408	0.400	Bad
	C4.5	0.720	0.700	0.408	0.400	Bad
	kNN	0.700	0.500	0.000	0.000	Bad
	SVM	0.680	0.500	ND	0.000	Bad
	XG	0.800	0.800	0.600	0.600	Good
	NB	0.720	0.400	-0.333	-0.200	Bad
	LR	0.720	0.700	0.408	0.400	Bad
DMF	RF	1.000	1.000	1.000	1.000	Good
	C4.5	1.000	1.000	1.000	1.000	Good
	kNN	0.875	0.875	0.612	0.545	Good
	SVM	0.375	0.500	ND	0.000	Bad
	XG	1.000	1.000	1.000	1.000	Good
	NB	1.000	0.625	0.250	0.118	Bad
	LR	1.000	1.000	1.000	1.000	Good
DMSO	RF	1.000	0.938	0.764	0.737	Good
	C4.5	1.000	1.000	1.000	1.000	Good
	kNN	0.875	0.938	0.764	0.737	Good
	SVM	0.000	0.500	ND	0.000	Bad
	XG	1.000	0.750	0.667	0.615	Good
	NB	0.938	0.625	0.250	0.118	Bad
	LR	1.000	0.938	0.764	0.737	Good
Ethanol	RF	0.920	0.700	0.408	0.400	Good
	C4.5	0.760	0.800	0.655	0.600	Good
	kNN	0.580	0.600	0.333	0.200	Bad
	SVM	0.400	0.500	ND	0.000	Bad
	XG	0.800	0.700	0.500	0.400	Good
	NB	0.96	0.500	ND	0.000	Bad
	LR	0.840	0.700	0.500	0.400	Good
THF	RF	1.000	1.000	1.000	1.000	Good
	C4.5	1.000	0.929	0.756	0.727	Good
	kNN	1.000	0.786	0.478	0.372	Bad
	SVM	0.714	0.500	ND	0.000	Bad
	XG	1.000	0.929	0.756	0.727	Good
	NB	1.000	0.643	0.286	0.151	Bad
	LR	1.000	1.000	1.000	1.000	Good

ND Not defined

Please do not adjust margins

Please do not adjust margins

Table S7 Optimisation and performance statistics of the QSPR models developed for the in-domain external test set compounds using the dsz applicability domain.

		Performance of optimal model on dsz in-domain test set				
Model	Method	ROC AUC	Balanced Accuracy	MCC	K	Overall quality of model
Acetone	RF	0.872	0.875	0.775	0.750	Good
	C4.5	0.875	0.875	0.755	0.750	Good
	kNN	0.844	0.625	0.378	0.250	Bad
	SVM	0.938	0.500	ND	0.000	Bad
	XG	1.000	0.875	0.775	0.750	Good
	NB	1.000	0.500	ND	0	Bad
	LR	0.875	0.875	0.775	0.750	Good
DMF	RF	1.000	1.000	1.000	1.000	Good
	C4.5	1.000	1.000	1.000	1.000	Good
	kNN	0.833	0.833	0.577	0.500	Good
	SVM	0.500	0.500	ND	0.000	Bad
	XG	1.000	1.000	1.000	1.000	Good
	NB	1.000	0.500	ND	0.000	Bad
	LR	1.000	1.000	1.000	1.000	Good
DMSO	RF	1.000	0.900	0.632	0.571	Good
	C4.5	1.000	1.000	1.000	1.000	Good
	kNN	0.800	0.900	0.632	0.571	Good
	SVM	0.000	0.500	ND	0.000	Bad
	XG	1.000	1.000	1.000	1.000	Good
	NB	1.000	0.500	ND	0.000	Bad
	LR	1.000	0.900	0.632	0.571	Good
Ethanol	RF	0.950	0.775	0.550	0.550	Good
	C4.5	0.850	0.875	0.791	0.769	Good
	kNN	0.575	0.625	0.395	0.270	Bad
	SVM	0.400	0.500	ND	0.000	Bad
	XG	0.900	0.750	0.598	0.526	Good
	NB	0.960	0.500	ND	0.000	Bad
	LR	0.900	0.750	0.598	0.526	Good
THF	RF	1.000	1.000	1.000	1.000	Good
	C4.5	1.000	0.929	0.756	0.727	Good
	kNN	1.000	0.786	0.478	0.372	Bad
	SVM	0.714	0.500	ND	0.000	Bad
	XG	1.000	0.929	0.756	0.727	Good
	NB	1.000	0.643	0.286	0.151	Bad
	LR	1.000	1.000	1.000	1.000	Good

ND Not defined

This journal is © The Royal Society of Chemistry 20xx

Please do not adjust margins

Please do not adjust margins

Table S8 Confusion Matrices for Acetone model predictions of the training set and in-domain maxdist and dsz sets.

		Training		Maxdist		dsz	
		Soluble	Insolbule	Soluble	Insolbule	Soluble	Insolbule
RF	Soluble	19	0	3	1	3	0
	Insoluble	3	21	2	4	1	4
C4.5	Soluble	18	2	3	1	3	0
	Insoluble	4	19	2	4	1	4
kNN	Soluble	14	7	1	1	1	0
	Insoluble	8	14	4	4	3	4
SVM	Soluble	22	21	5	5	4	4
	Insoluble	0	0	0	0	0	0
XG	Soluble	20	2	4	1	3	0
	Insoluble	2	19	1	4	1	4
NB	Soluble	11	1	0	1	0	0
	Insoluble	11	20	5	4	4	4
LR	Soluble	20	3	3	1	3	0
	Insoluble	2	18	2	4	1	4

Table S9 Confusion Matrices for DMF model predictions of the training set and in-domain maxdist and dsz sets.

		Training		Maxdist		dsz	
		Soluble	Insolbule	Soluble	Insolbule	Soluble	Insolbule
RF	Soluble	35	0	8	0	6	0
	Insoluble	1	6	0	2	0	2
C4.5	Soluble	35	0	8	0	6	0
	Insoluble	1	6	0	2	0	2
kNN	Soluble	28	0	6	0	4	0
	Insoluble	8	6	2	2	2	2
SVM	Soluble	36	6	8	2	6	2
	Insoluble	0	0	0	0	0	0
XG	Soluble	36	0	8	0	6	0
	Insoluble	0	6	0	2	0	2
NB	Soluble	13	0	2	0	0	0
	Insoluble	23	6	6	2	6	2
LR	Soluble	35	0	8	0	6	0
	Insoluble	1	6	0	2	0	2

Table S10 Confusion Matrices for DMSO model predictions of the training set and in-domain maxdist and dsz sets.

		Training		Maxdist		dsz	
		Soluble	Insolbule	Soluble	Insolbule	Soluble	Insolbule
RF	Soluble	36	0	7	0	4	0
	Insoluble	1	6	1	2	1	1
C4.5	Soluble	36	0	8	0	5	0
	Insoluble	1	6	0	2	0	1
kNN	Soluble	26	0	7	0	4	0
	Insoluble	11	6	1	2	1	1
SVM	Soluble	37	6	8	2	5	1
	Insoluble	0	0	0	0	0	0
XG	Soluble	35	0	8	1	5	0
	Insoluble	2	6	0	1	0	1
NB	Soluble	8	0	2	0	0	0
	Insoluble	29	6	6	2	5	1
LR	Soluble	35	0	7	0	4	0
	Insoluble	2	6	1	2	1	1

Please do not adjust margins

Please do not adjust margins

Table S11 Confusion Matrices for Ethanol model predictions of the training set and in-domain maxdist and dsz sets.

		Training		Maxdist		dsz	
		Soluble	Insolbule	Soluble	Insolbule	Soluble	Insolbule
RF	Soluble	18	0	3	1	3	1
	Insoluble	0	23	2	4	1	4
C4.5	Soluble	18	1	3	0	3	0
	Insoluble	0	22	2	5	1	5
kNN	Soluble	13	5	1	0	1	0
	Insoluble	5	18	4	5	3	5
SVM	Soluble	18	0	0	0	0	0
	Insoluble	0	23	5	5	4	5
XG	Soluble	18	0	2	0	2	0
	Insoluble	0	23	3	5	2	5
NB	Soluble	18	18	5	5	4	5
	Insoluble	0	5	0	0	0	0
LR	Soluble	13	2	2	0	2	0
	Insoluble	5	21	3	5	2	5

Table S12 Confusion Matrices for THF model predictions of the training set and in-domain maxdist and dsz sets.

		Training		Maxdist		dsz	
		Soluble	Insolbule	Soluble	Insolbule	Soluble	Insolbule
RF	Soluble	31	1	7	0	7	0
	Insoluble	0	10	0	2	0	2
C4.5	Soluble	31	1	6	0	6	0
	Insoluble	0	10	1	2	1	2
kNN	Soluble	23	1	4	0	4	0
	Insoluble	8	10	3	2	3	2
SVM	Soluble	31	11	7	2	7	2
	Insoluble	0	0	0	0	0	0
XG	Soluble	31	1	6	0	6	0
	Insoluble	0	10	1	2	1	2
NB	Soluble	14	0	2	0	2	0
	Insoluble	17	11	5	2	5	2
LR	Soluble	28	1	7	0	7	0
	Insoluble	3	10	0	2	0	2

This journal is © The Royal Society of Chemistry 20xx

Please do not adjust margins

Please do not adjust margins

Table S13 Summary of the number of models which provided good predictive statistics for their respective training set and in-domain (maxdist and dsz) test set compounds over 10 random stratified partitions. The number of in-domain compounds for each partition is also tabulated.

		Solvent				
Partition	Partition Number	Acetone	DMF	DMSO	Ethanol	THF
Training	1	5	5	5	5	5
	2	6	5	4	4	4
	3	3	4	4	6	3
	4	4	6	5	5	5
	5	5	4	4	6	5
	6	6	5	5	4	5
	7	6	5	4	7	5
	8	6	5	4	7	5
	9	6	5	5	3	4
	10	6	5	5	6	5
Maxdist	1	4	5	4	1	5
	2	2	5	4	0	4
	3	0	5	4	0	1
	4	0	6	5	0	1
	5	1	4	4	3	4
	6	4	5	5	5	5
	7	0	5	5	0	6
	8	0	5	5	0	1
	9	0	5	3	6	4
	10	0	5	5	0	0
Dsz	1	4	5	4	1	5
	2	0	5	0	0	3
	3	3	5	4	0	0
	4	0	5	5	1	0
	5	4	4	4	5	4
	6	4	5	5	3	3
	7	5	5	5	0	5
	8	2	4	5	0	1
	9	0	5	0	2	5
	10	5	5	5	2	5
Dsz Out-of-Domain Compounds	1	4	2	4	1	4
	2	5	5	1	1	5
	3	6	5	6	2	3
	4	2	5	6	1	5
	5	2	3	6	1	1
	6	4	3	6	5	4
	7	4	3	4	4	3
	8	6	5	4	4	5
	9	2	6	3	3	4
	10	5	1	5	3	4

Please do not adjust margins

Please do not adjust margins

Table S14 Summary of the model statistics for the training set compounds over 10 random stratified partitions.

		AUC ROC	Balanced Accuracy	MCC	Cohen's Kappa
Acetone	RF	0.99 ± 0.007	0.948 ± 0.023	0.898 ± 0.045	0.893 ± 0.047
	C4.5	0.957 ± 0.028	0.882 ± 0.052	0.769 ± 0.104	0.763 ± 0.103
	kNN	0.729 ± 0.05	0.744 ± 0.06	0.496 ± 0.119	0.488 ± 0.121
	SVM	0.924 ± 0.109	0.826 ± 0.217	0.654 ± 0.434	0.653 ± 0.434
	XG	0.977 ± 0.009	0.912 ± 0.034	0.826 ± 0.069	0.823 ± 0.069
	NB	0.844 ± 0.036	0.72 ± 0.052	0.487 ± 0.06	0.441 ± 0.104
	LR	0.921 ± 0.031	0.862 ± 0.038	0.727 ± 0.076	0.725 ± 0.076
DMF	RF	0.996 ± 0.006	0.987 ± 0.004	0.922 ± 0.026	0.918 ± 0.027
	C4.5	0.996 ± 0.006	0.986 ± 0.006	0.925 ± 0.027	0.91 ± 0.038
	kNN	0.982 ± 0.021	0.883 ± 0.019	0.567 ± 0.038	0.487 ± 0.048
	SVM	0.332 ± 0.09	0.5 ± 0	0 ± 0	0 ± 0
	XG	0.998 ± 0.003	0.987 ± 0.01	0.925 ± 0.056	0.92 ± 0.06
	NB	0.989 ± 0.007	0.672 ± 0.026	0.264 ± 0.029	0.132 ± 0.026
	LR	0.997 ± 0.006	0.983 ± 0.008	0.9 ± 0.046	0.894 ± 0.05
DMSO	RF	0.99 ± 0.009	0.984 ± 0.012	0.903 ± 0.064	0.897 ± 0.07
	C4.5	0.99 ± 0.007	0.982 ± 0.012	0.896 ± 0.067	0.889 ± 0.073
	kNN	0.984 ± 0.019	0.876 ± 0.028	0.55 ± 0.057	0.464 ± 0.074
	SVM	0.362 ± 0.12	0.5 ± 0	0 ± 0	0 ± 0
	XG	0.992 ± 0.006	0.974 ± 0.004	0.849 ± 0.021	0.838 ± 0.024
	NB	0.965 ± 0.016	0.643 ± 0.03	0.23 ± 0.032	0.094 ± 0.04
	LR	0.989 ± 0.008	0.969 ± 0.006	0.824 ± 0.027	0.809 ± 0.032
Ethanol	RF	1 ± 0	0.991 ± 0.011	0.981 ± 0.024	0.98 ± 0.024
	C4.5	0.97 ± 0.052	0.914 ± 0.096	0.823 ± 0.189	0.819 ± 0.191
	kNN	0.79 ± 0.085	0.733 ± 0.063	0.466 ± 0.123	0.463 ± 0.121
	SVM	1 ± 0	0.997 ± 0.008	0.995 ± 0.015	0.995 ± 0.015
	XG	0.998 ± 0.003	0.978 ± 0.017	0.956 ± 0.034	0.956 ± 0.035
	NB	0.824 ± 0.029	0.705 ± 0.048	0.472 ± 0.088	0.384 ± 0.095
	LR	0.894 ± 0.066	0.777 ± 0.064	0.555 ± 0.129	0.548 ± 0.125
THF	RF	0.997 ± 0.005	0.968 ± 0.017	0.936 ± 0.036	0.934 ± 0.039
	C4.5	0.998 ± 0.004	0.974 ± 0.016	0.946 ± 0.018	0.945 ± 0.018
	kNN	0.929 ± 0.059	0.842 ± 0.069	0.625 ± 0.127	0.594 ± 0.136
	SVM	0.539 ± 0.134	0.5 ± 0	0 ± 0	0 ± 0
	XG	0.996 ± 0.004	0.971 ± 0.017	0.927 ± 0.046	0.923 ± 0.05
	NB	0.894 ± 0.028	0.692 ± 0.019	0.358 ± 0.032	0.261 ± 0.031
	LR	0.944 ± 0.089	0.907 ± 0.045	0.785 ± 0.097	0.779 ± 0.099

This journal is © The Royal Society of Chemistry 20xx

Please do not adjust margins

Please do not adjust margins

Table S15 Summary of the model statistics for the test compounds within the maxdist domain over 10 random stratified partitions.

		AUC ROC	Balanced Accuracy	MCC	Cohen's Kappa
Acetone	RF	0.617 ± 0.117	0.615 ± 0.138	0.236 ± 0.283	0.22 ± 0.289
	C4.5	0.656 ± 0.136	0.603 ± 0.143	0.216 ± 0.299	0.205 ± 0.287
	kNN	0.68 ± 0.14	0.585 ± 0.084	0.177 ± 0.175	0.17 ± 0.168
	SVM	0.548 ± 0.124	0.53 ± 0.078	0.074 ± 0.213	0.058 ± 0.159
	XG	0.653 ± 0.134	0.665 ± 0.105	0.335 ± 0.208	0.27 ± 0.21
	NB	0.51 ± 0.168	0.463 ± 0.127	-0.1 ± 0.288	-0.035 ± 0.263
	LR	0.625 ± 0.147	0.603 ± 0.136	0.21 ± 0.275	0.205 ± 0.272
DMF	RF	0.991 ± 0.028	0.994 ± 0.019	0.976 ± 0.071	0.977 ± 0.07
	C4.5	0.991 ± 0.028	0.938 ± 0.097	0.886 ± 0.143	0.87 ± 0.163
	kNN	0.899 ± 0.071	0.874 ± 0.038	0.619 ± 0.083	0.557 ± 0.112
	SVM	0.231 ± 0.19	0.5 ± 0	0 ± 0	0 ± 0
	XG	0.991 ± 0.02	0.913 ± 0.109	0.853 ± 0.151	0.832 ± 0.173
	NB	0.978 ± 0.028	0.664 ± 0.069	0.302 ± 0.09	0.175 ± 0.096
	LR	0.991 ± 0.028	0.969 ± 0.042	0.89 ± 0.141	0.876 ± 0.161
DMSO	RF	0.938 ± 0.079	0.831 ± 0.125	0.65 ± 0.221	0.609 ± 0.228
	C4.5	0.937 ± 0.079	0.844 ± 0.138	0.697 ± 0.261	0.661 ± 0.276
	kNN	0.861 ± 0.105	0.797 ± 0.135	0.541 ± 0.262	0.481 ± 0.282
	SVM	0.264 ± 0.204	0.5 ± 0	0 ± 0	0 ± 0
	XG	0.938 ± 0.097	0.881 ± 0.11	0.776 ± 0.198	0.738 ± 0.235
	NB	0.897 ± 0.105	0.647 ± 0.056	0.276 ± 0.074	0.2 ± 0.13
	LR	0.963 ± 0.064	0.894 ± 0.101	0.77 ± 0.201	0.731 ± 0.239
Ethanol	RF	0.712 ± 0.173	0.61 ± 0.145	0.228 ± 0.303	0.22 ± 0.289
	C4.5	0.692 ± 0.157	0.66 ± 0.136	0.348 ± 0.283	0.32 ± 0.271
	kNN	0.578 ± 0.184	0.55 ± 0.136	0.109 ± 0.311	0.1 ± 0.272
	SVM	0.552 ± 0.102	0.49 ± 0.03	-0.033 ± 0.1	-0.02 ± 0.06
	XG	0.704 ± 0.172	0.63 ± 0.162	0.272 ± 0.337	0.26 ± 0.323
	NB	0.732 ± 0.179	0.61 ± 0.13	0.257 ± 0.274	0.22 ± 0.26
	LR	0.656 ± 0.178	0.56 ± 0.143	0.152 ± 0.316	0.12 ± 0.286
THF	RF	1.86 ± 2.955	0.798 ± 0.125	0.579 ± 0.242	0.562 ± 0.237
	C4.5	0.836 ± 0.149	0.805 ± 0.111	0.595 ± 0.204	0.574 ± 0.195
	kNN	0.84 ± 0.127	0.735 ± 0.072	0.482 ± 0.101	0.426 ± 0.092
	SVM	0.426 ± 0.15	0.5 ± 0	0 ± 0	0 ± 0
	XG	0.823 ± 0.15	0.738 ± 0.125	0.458 ± 0.226	0.451 ± 0.221
	NB	0.902 ± 0.105	0.66 ± 0.078	0.321 ± 0.143	0.221 ± 0.118
	LR	0.862 ± 0.16	0.786 ± 0.132	0.542 ± 0.248	0.519 ± 0.247

Please do not adjust margins

Please do not adjust margins

Table S16 Summary of the model statistics for the test compounds within the dsz domain over 10 random stratified partitions.

		AUC ROC	Balanced Accuracy	MCC	Cohen's Kappa
Acetone	RF	0.745 ± 0.204	0.7 ± 0.187	0.422 ± 0.379	0.376 ± 0.373
	C4.5	0.749 ± 0.251	0.713 ± 0.168	0.447 ± 0.34	0.401 ± 0.336
	kNN	0.699 ± 0.233	0.633 ± 0.188	0.254 ± 0.356	0.199 ± 0.316
	SVM	0.543 ± 0.298	0.527 ± 0.19	0.078 ± 0.357	0.053 ± 0.292
	XG	0.76 ± 0.212	0.753 ± 0.187	0.518 ± 0.376	0.478 ± 0.382
	NB	0.481 ± 0.305	0.464 ± 0.225	-0.063 ± 0.437	-0.056 ± 0.416
	LR	0.642 ± 0.246	0.714 ± 0.195	0.468 ± 0.377	0.417 ± 0.372
DMF	RF	0.989 ± 0.032	0.993 ± 0.021	0.976 ± 0.073	0.973 ± 0.082
	C4.5	0.989 ± 0.032	0.933 ± 0.097	0.866 ± 0.173	0.847 ± 0.197
	kNN	0.911 ± 0.099	0.839 ± 0.098	0.652 ± 0.164	0.592 ± 0.2
	SVM	0.302 ± 0.263	0.5 ± 0	0 ± 0	0 ± 0
	XG	0.98 ± 0.041	0.908 ± 0.108	0.832 ± 0.175	0.806 ± 0.204
	NB	0.963 ± 0.066	0.534 ± 0.056	0.086 ± 0.14	0.048 ± 0.089
	LR	0.989 ± 0.032	0.966 ± 0.056	0.915 ± 0.131	0.904 ± 0.15
DMSO	RF	0.9 ± 0.144	0.826 ± 0.161	0.655 ± 0.309	0.615 ± 0.317
	C4.5	0.9 ± 0.144	0.836 ± 0.168	0.692 ± 0.326	0.658 ± 0.337
	kNN	0.827 ± 0.165	0.756 ± 0.187	0.515 ± 0.359	0.458 ± 0.369
	SVM	0.29 ± 0.221	0.5 ± 0	0 ± 0	0 ± 0
	XG	0.915 ± 0.121	0.886 ± 0.121	0.783 ± 0.231	0.743 ± 0.276
	NB	0.92 ± 0.145	0.507 ± 0.021	0.019 ± 0.057	0.007 ± 0.021
	LR	0.93 ± 0.125	0.889 ± 0.121	0.778 ± 0.233	0.752 ± 0.273
Ethanol	RF	0.761 ± 0.179	0.624 ± 0.176	0.273 ± 0.367	0.245 ± 0.35
	C4.5	0.742 ± 0.152	0.679 ± 0.16	0.39 ± 0.33	0.359 ± 0.32
	kNN	0.539 ± 0.161	0.51 ± 0.099	0.032 ± 0.284	0.023 ± 0.195
	SVM	0.606 ± 0.148	0.5 ± 0	0 ± 0	0 ± 0
	XG	0.767 ± 0.195	0.669 ± 0.172	0.369 ± 0.361	0.339 ± 0.346
	NB	0.723 ± 0.183	0.618 ± 0.106	0.267 ± 0.244	0.228 ± 0.225
	LR	0.632 ± 0.178	0.516 ± 0.151	0.059 ± 0.364	0.029 ± 0.306
THF	RF	0.881 ± 0.171	0.819 ± 0.132	0.625 ± 0.249	0.585 ± 0.264
	C4.5	0.87 ± 0.174	0.822 ± 0.134	0.627 ± 0.251	0.588 ± 0.266
	kNN	0.86 ± 0.138	0.763 ± 0.072	0.51 ± 0.155	0.445 ± 0.155
	SVM	0.716 ± 0.192	0.5 ± 0	0 ± 0	0 ± 0
	XG	0.897 ± 0.131	0.729 ± 0.181	0.444 ± 0.349	0.425 ± 0.338
	NB	0.906 ± 0.131	0.526 ± 0.119	0.054 ± 0.278	0.01 ± 0.211
	LR	0.881 ± 0.171	0.79 ± 0.138	0.567 ± 0.26	0.524 ± 0.273

This journal is © The Royal Society of Chemistry 20xx

Please do not adjust margins

Please do not adjust margins

Table S17 Crtitcal Z-scores (Z_c) and their corresponding levels of significance (α)

Z_c	α
1.28	0.10
1.64	0.05
2.33	0.01
3.10	0.001

Table S18 Z-scores for "good" models on the randomised test and in-domain test sets. Scores above the 0.05 confidence threshold are highlighted in green, and scores below are highlighted in red.

Model	Partition	Method			
		RF	C4.5	XG	LR
Acetone	Test	0.941	1.115	1.592	1.306
	Dsz	2.132	1.789	2.219	1.719
DMF	Test	2.804	2.691	3.477	3.25
	Dsz	2.810	2.674	2.407	2.232
DMSO	Test	2.137	3.250	1.520	2.020
	Dsz	1.520	2.054	2.280	1.484
Ethanol	Test	1.660	2.058	1.497	1.359
	Dsz	1.533	2.309	1.717	1.716
THF	Test	2.303	1.466	1.497	2.398
	Dsz	2.581	1.741	2.071	3.284

Table S19 Z-scores for "good" models on the test and in-domain test sets applied to models built with randomised training data. Scores above the 0.05 confidence threshold are highlighted in green, and scores below are highlighted in red.

Model	Partition	Method			
		RF	C4.5	XG	LR
Acetone	Test	1.471	0.849	2.653	1.605
	Dsz	2.144	1.759	2.902	2.471
DMF	Test	2.250	3.109	2.663	2.696
	Dsz	2.202	2.982	1.805	2.375
DMSO	Test	1.752	2.297	2.017	2.511
	Dsz	1.624	1.959	1.821	1.446
Ethanol	Test	1.551	1.333	1.132	2.203
	Dsz	2.030	1.792	1.385	2.444
THF	Test	2.138	0.997	2.610	1.634
	Dsz	2.457	1.473	2.008	1.717

Please do not adjust margins

Table S20 One-tailed one-sample t-test p-values for "good" models on the training, test, and in-domain test sets applied to models built with randomised training data. Scores below the 0.05 p-value threshold are highlighted in green.

		Method			
Model	Partition	RF	C4.5	XG	LR
Acetone	Test	1.127×10^{-3}	1.255×10^{-2}	7.560×10^{-6}	3.348×10^{-4}
	Dsz	1.009×10^{-4}	1.747×10^{-4}	3.629×10^{-6}	3.744×10^{-5}
DMF	Test	2.790×10^{-5}	2.058×10^{-6}	7.331×10^{-6}	6.627×10^{-6}
	Dsz	3.297×10^{-5}	9.686×10^{-6}	6.955×10^{-4}	4.974×10^{-5}
DMSO	Test	1.802×10^{-4}	2.369×10^{-5}	6.403×10^{-5}	3.352×10^{-5}
	Dsz	6.218×10^{-4}	1.855×10^{-4}	6.611×10^{-4}	2.324×10^{-3}
Ethanol	Test	4.227×10^{-4}	1.124×10^{-3}	2.967×10^{-3}	3.284×10^{-5}
	Dsz	6.126×10^{-5}	1.529×10^{-4}	8.806×10^{-4}	1.452×10^{-5}
THF	Test	4.116×10^{-5}	5.825×10^{-3}	8.609×10^{-6}	2.932×10^{-4}
	Dsz	1.396×10^{-5}	5.938×10^{-4}	6.652×10^{-5}	2.084×10^{-4}

This journal is © The Royal Society of Chemistry 20xx

Table S21 Validation predictions and their prediction confidences. The consensus values across the four methods is also provided.

		Individual Methods				Overall	
Compound	Solvent	RF Prediction (Confidence)	C 4.5 Prediction (Confidence)	XG Prediction (Confidence)	LR Prediction (Confidence)	Prediction (Confidence)	Measurement
	Acetone	Soluble (24%)	Soluble (20%)	Soluble (28%)	Soluble (18%)	Soluble (23 ± 4.4%)	Insoluble
	DMF	Soluble (94%)	Soluble (98%)	Soluble (98%)	Soluble (88%)	Soluble (95 ± 4.7%)	Soluble
	DMSO	Soluble (96%)	Soluble (98%)	Soluble (96%)	Soluble (94%)	Insoluble (96 ± 1.6%)	Soluble
	Ethanol	Insoluble (16%)	Insoluble (26%)	Insoluble (54%)	Insoluble (10%)	Insoluble (27 ± 19.5%)	Insoluble
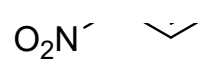	THF	Soluble (76%)	Soluble (80%)	Soluble (76%)	Soluble (78%)	Soluble (78 ± 1.9%)	Soluble
	Acetone	Soluble (62%)	Soluble (50%)	Soluble (38%)	Soluble (44%)	Soluble (49 ± 10.2%)	Soluble
	DMF	Soluble (100%)	Soluble (100%)	Soluble (94%)	Soluble (98%)	Soluble (98 ± 2.8%)	Soluble
	DMSO	Soluble (94%)	Soluble (98%)	Soluble (94%)	Soluble (90%)	Soluble (94 ± 3.3%)	Soluble
	Ethanol	Soluble (70%)	Soluble (72%)	Soluble (80%)	Soluble (60%)	Soluble (71 ± 8.2%)	Soluble
	THF	Soluble (98%)	Soluble (98%)	Soluble (96%)	Soluble (94%)	Soluble (77 ± 1.9%)	Soluble
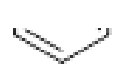 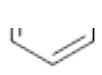		Synthetically Inaccessible using reported methods[46, 47]					

Please do not adjust margins

References

1. L. Chen, S. Revel, K. Morris and D. J. Adams, *Chemical Communications*, 2010, **46**, 4267–4269.
2. J. K. Gupta, D. J. Adams and N. G. Berry, *Chemical Science*, 2016, **7**, 4713–4719.
3. J. D. Simpson, L. Thomson, C. M. Woodley, C. M. Wallace, B. Dietrich, A. S. Loch, D. J. Adams and N. G. Berry, *Advanced Materials*, 2025, **37**, 2415031.
4. E. L. Bowley, S. Bianco, F. H. Stewart, C. M. Wallace, R. E. Ginesi, A. S. Loch, M. Rosenthal, A. J. Smith and D. J. Adams, *ChemSystemsChem*, 2025, **7**, e202400073.
5. C. Patterson, B. Dietrich, C. Wilson, A. R. Mount and D. J. Adams, *Soft Matter*, 2022, **18**, 1064–1070.
6. R. I. Randle, L. Cavalcanti, S. Sproules and E. R. Draper, *Materials Advances*, 2022, **3**, 3326–3331.
7. P. Pengo, G. D. Pantoş, S. Otto and J. K. M. Sanders, *The Journal of Organic Chemistry*, 2006, **71**, 7063–7066.
8. N. R. Murray, T. J. D. McCabe, M. Reid and E. R. Draper, *Journal of Materials Chemistry C*, 2024, **12**, 12483–12490.
9. E. R. Draper, L. J. Archibald, M. C. Nolan, R. Schweins, M. A. Zwijnenburg, S. Sproules and D. J. Adams, *Chemistry – A European Journal*, 2018, **24**, 4006–4010.
10. E. Kavery, N. Nagarajan, G. Paramaguru and R. Renganathan, *Spectrochimica Acta Part A: Molecular and Biomolecular Spectroscopy*, 2015, **146**, 13–23.
11. E. R. Draper, B. J. Greeves, M. Barrow, R. Schweins, M. A. Zwijnenburg and D. J. Adams, *Chem*, 2017, **2**, 716–731.
12. R. F. Araújo, C. J. R. Silva, M. C. Paiva, M. M. Franco and M. F. Proença, *RSC Advances*, 2013, **3**, 24535–24542.
13. Y. Xie, C. Liu, L. Cheng, Y. Fan, H. Li, W. Liu, L. Zhu, X. Li, K. Deng, Q. Zeng and S. Han, *Chinese Chemical Letters*, 2022, **33**, 4649–4654.
14. J. G. Egan, G. Brodie, D. McDowall, A. J. Smith, C. J. C. Edwards-Gayle and E. R. Draper, *Materials Advances*, 2021, **2**, 5248–5253.
15. E. Kozma, G. Grisci, W. Mróz, M. Catellani, A. Eckstein-Andicsovà, K. Pagano and F. Galeotti, *Dyes and Pigments*, 2016, **125**, 201–209.
16. S. Panja, B. Dietrich, A. Trabold, A. Zydel, A. Qadir and D. J. Adams, *Chemical Communications*, 2021, **57**, 7898–7901.
17. A. M. Fuentes-Caparrós, K. McAulay, S. E. Rogers, R. M. Dalgliesh and D. J. Adams, *Journal*, 2019, **24**.
18. Z. Xu, J. Doutch, N. Mahmoudi, S. Sproules and E. R. Draper, *Journal of Materials Chemistry C*, 2026, **14**, 205–211.
19. L. Chen, S. Revel, K. Morris, L. C. Serpell and D. J. Adams, *Langmuir*, 2010, **26**, 13466–13471.
20. E. Buncel, N. L. Mailloux, R. S. Brown, P. M. Kazmaier and J. Dust, *Tetrahedron Letters*, 2001, **42**, 3559–3562.
21. Chemdraw Version 23.0.1.10, https://revvitysignals.com/products/research/chemdraw, (accessed 21 April 2024).
22. D. Weininger, *Journal of Chemical Information and Computer Sciences*, 1988, **28**, 31–36.
23. M. R. Berthold, N. Cebron, F. Dill, T. R. Gabriel, T. Kötter, T. Meinl, P. Ohl, C. Sieb, K. Thiel and B. Wiswedel, Berlin, Heidelberg, 2008.
24. S. A. Wildman and G. M. Crippen, *Journal of Chemical Information and Computer Sciences*, 1999, **39**, 868–873.
25. P. Labute, *Journal of Molecular Graphics and Modelling*, 2000, **18**, 464–477.
26. P. Ertl, B. Rohde and P. Selzer, *Journal of Medicinal Chemistry*, 2000, **43**, 3714–3717.
27. A. Capecchi, D. Probst and J.-L. Reymond, *Journal of Cheminformatics*, 2020, **12**, 43.
28. D. Rogers and M. Hahn, *Journal of Chemical Information and Modeling*, 2010, **50**, 742–754.
29. A. Golbraikh, E. Muratov, D. Fourches and A. Tropsha, *J Chem Inf Model*, 2014, **54**, 1–4.
30. F. Sahigara, K. Mansouri, D. Ballabio, A. Mauri, V. Consonni and R. Todeschini, *Molecules*, 2012, **17**, 4791–4810.
31. C. C. Aggarwal, A. Hinneburg and D. A. Keim, Berlin, Heidelberg, 2001.
32. C. Spearman, *International Journal of Epidemiology*, 2010, **39**, 1137–1150.
33. L. Wang, M. Han, X. Li, N. Zhang and H. Cheng, *IEEE Access*, 2021, **9**, 64606–64628.
34. N. V. Chawla, K. W. Bowyer, L. O. Hall and W. P. Kegelmeyer, *Journal of artificial intelligence research*, 2002, **16**, 321–357.
35. D. Chicco and G. Jurman, *BioData Mining*, 2023, **16**, 4.
36. J. Bergstra, R. Bardenet, Y. Bengio and B. Kégl, *Advances in neural information processing systems*, 2011, **24**.
37. M. Shen, A. LeTiran, Y. Xiao, A. Golbraikh, H. Kohn and A. Tropsha, *J Med Chem*, 2002, **45**, 2811–2823.
38. S. M. Lundberg and S.-I. Lee, *Advances in neural information processing systems*, 2017, **30**.
39. Y. Kwon, M. A. Rivas and J. Zou, 2021.
40. S. L. Brunton and J. N. Kutz, *Data-driven science and engineering: machine learning, dynamical systems, and control*, Cambridge University Press, Cambridge, United Kingdom, New York, NY, Second edn., 2022.
41. G. v. Rossum and F. L. Drake, *The Python Language Reference Manual*, Network Theory Ltd., 2011.
42. G. Landrum, RDKit: Open-source cheminformatics, https://www.rdkit.org, DOI: 10.5281/zenodo.7828379).
43. G. Landrum, P. Tosco, B. Kelley, R. Rodriguez, D. Cosgrove, Sriniker, R. Vianello, Gedeck, N. Schneider, G. Jones, E. Kawashima, D. Nealschneider, A. Dalke, B. Cole, M. Swain, S. Turk, A. Savelev, A. Vaucher and M. Wójcikowski, RDKit Release_2024.03.1, DOI: https://doi.org/10.5281/zenodo.10893044).
44. D. Huang, Add R-groups to RDKIT Molecules, https://oloren-ai.github.io/blog/add_rgroup.html, (accessed 10 July 2023, 2023).
45. W. Tong, Q. Xie, H. Hong, L. Shi, H. Fang and R. Perkins, *Environ Health Perspect*, 2004, **112**, 1249–1254.
46. S. Roy Chowdhury, S. Mondal and P. K. Iyer, *ACS Biomaterials Science & Engineering*, 2018, **4**, 4076–4083.
47. CN118184679, 2024.

This journal is © The Royal Society of Chemistry 20xx

Please do not adjust margins
